# Pitchfork and Gprasp2 Target Smoothened to the Primary Cilium for Hedgehog Pathway Activation

**DOI:** 10.1371/journal.pone.0149477

**Published:** 2016-02-22

**Authors:** Bomi Jung, Daniela Padula, Ingo Burtscher, Cedric Landerer, Dominik Lutter, Fabian Theis, Ana C. Messias, Arie Geerlof, Michael Sattler, Elisabeth Kremmer, Karsten Boldt, Marius Ueffing, Heiko Lickert

**Affiliations:** 1 Institute of Diabetes and Regeneration Research, Helmholtz Zentrum München, München-Neuherberg, Germany; 2 Institute of Stem Cell Research, Helmholtz Zentrum München, München-Neuherberg, Germany; 3 Institute of Computational Biology, Helmholtz Zentrum München, München-Neuherberg, Germany; 4 Institute of Diabetes and Adipositas, Helmholtz Zentrum München, München-Neuherberg, Germany; 5 Institute of Structural Biology, Helmholtz Zentrum München, München-Neuherberg, Germany; 6 Institute of Molecular Immunology, Helmholtz Zentrum München, München-Neuherberg, Germany; 7 Department of Protein Science, Helmholtz Zentrum München, München-Neuherberg, Germany; 8 German Center for Diabetes Research (DZD), 85764 München-Neuherberg, Germany; 9 Center for Integrated Protein Science Munich at Biomolecular NMR Spectroscopy, Department Chemie, Technische Universität München, 85747 Garching, Germany; 10 Centre of Ophthalmology, Institute for Ophthalmology Research, University of Tuebingen, 72076 Tuebingen, Germany; 11 Department of Ecology & Evolutionary Biology, University of Tennessee, Knoxville TN 37996, United States of America; Indiana University School of Medicine, UNITED STATES

## Abstract

The seven-transmembrane receptor Smoothened (Smo) activates all Hedgehog (Hh) signaling by translocation into the primary cilia (PC), but how this is regulated is not well understood. Here we show that Pitchfork (Pifo) and the G protein-coupled receptor associated sorting protein 2 (Gprasp2) are essential components of an Hh induced ciliary targeting complex able to regulate Smo translocation to the PC. Depletion of Pifo or Gprasp2 leads to failure of Smo translocation to the PC and lack of Hh target gene activation. Together, our results identify a novel protein complex that is regulated by Hh signaling and required for Smo ciliary trafficking and Hh pathway activation.

## Introduction

The primary cilium (PC) is a microtubule-based organelle that emanates from the apical plasma membrane where it is anchored by the mother centriole of the centrosome, called basal body (BB) [1, 2]. PC assembly and disassembly are tightly linked to cell-cycle exit and reentry, respectively [3–6]. Almost all interphase and quiescent cells in the mammalian body posses a PC that is essential to receive and integrate extracellular signals [7–9]. As such it is not surprising that ciliary dysfunction leads to a variety of human syndromes collectively referred to as ciliopathies, e.g. Bardet-Biedl syndrome (BBS), Meckel-Gruber syndrome (MKS) or Ellis-van Creveld (EVC) syndrome [10–12]. Among the wide spectrum of phenotypes associated with ciliopathies, some abnormalities are attributed to defective Hedgehog (Hh) signaling, i.e. polydactyly, neural tube patterning defects, ataxia due to cerebellar hypoplasia and craniofacial defects [8, 13–15]. How PC integrate environmental signals to control tissue- and cell-type specific functions is still not well defined.

Vertebrate Hh proteins, Sonic (Shh), Indian (Ihh) and Desert hedgehog (Dhh), regulate diverse aspects of embryonic development and tissue homeostasis, while dysregulated signaling is associated with several types of tumors [16–18]. In vertebrates the PC acts as the key-signaling hub for the Hh pathway [19]. In the absence of Hh signaling, the twelve-transmembrane receptor (12TMR) Patched1 (Ptch1) localizes to the PC and suppresses the activity of the 7TMR Smo [20]. Upon Hh stimulation Smo translocation to the PC orchestrates all downstream signaling [21, 22]. This culminates in the activation of Hh target genes, such as Ptch1 and Gli1 by the GLI-Kruppel family member 1 and 2 (Gli1 and 2) of transcription factors [16–19]. Although most of the vertebrate Hh signaling components localize to the PC [23, 24] and efficient signaling transduction requires this organelle [13], regulation of Smo translocation into the PC remains enigmatic.

Several 7TM G-protein coupled receptors (7TM-GPCRs), such as melanin-concentrating hormone receptor 1 (Mchr1), somatostatin receptor type 3 (Sstr3) and Dopamine receptor 1 (D1), are localized to neuronal cilia, which likely involves receptor trafficking by BBS proteins [7]. Interestingly, the ciliary localization of 7TM-GPCRs including Smo depends on a conserved hydrophobic and basic residue motif in the carboxy-terminal tail [22, 25–27]. Moreover, loss of the retrograde microtuble (MT)-motor protein Dynein results in Smo enrichment in the PC, suggesting that Smo trafficking in and out of the PC is regulated by MT-dependent transport [28, 29]. This idea is further supported by the function of β-arrestins in mediating Smo transport into the PC via the anterograde Kinesin MT-motor protein Kif3A [30]. However, mouse knockout studies for either β-arrestin 1 or 2 did not reveal any obvious Hh phenotype [31, 32], suggesting functional redundancy or additional proteins involved in Smo trafficking to the PC.

During evolution, the PC was adapted for Hh signaling and many pathway components localize to the PC [19, 33]. We recently discovered Pitchfork (Pifo) which encodes long (28 kDa) and short (23 kDa) protein isoforms in a microarray-based screen to identify Spemann/Mangold organizer genes [5, 34]. Pifo appears with chordates and is specifically expressed in regions of embryonic organizer activities, such as the mouse node, the floor plate of the neural tube and the limb bud [5]. All of these tissues depend on Hh signaling for embryonic and tissue patterning [17, 18, 35]. Our recent analysis indicated that Pifo specifically regulates PC disassembly, but not assembly, by regulating the activity of AuroraA kinase at the BB and PC [5]. Pifo interacts and co-localizes with α, β, γ-Tubulin, MT-dependent motor proteins, small GTPases involved in vesicular trafficking and is dynamically transported along ciliary axonemal MTs. Taken together, these results suggest to us that Pifo might be required for the delivery of Hh components to the PC.

## Results

### Identification of a heterotrimeric Smo-Gprasp2-Pifo complex

**Fig 1:**
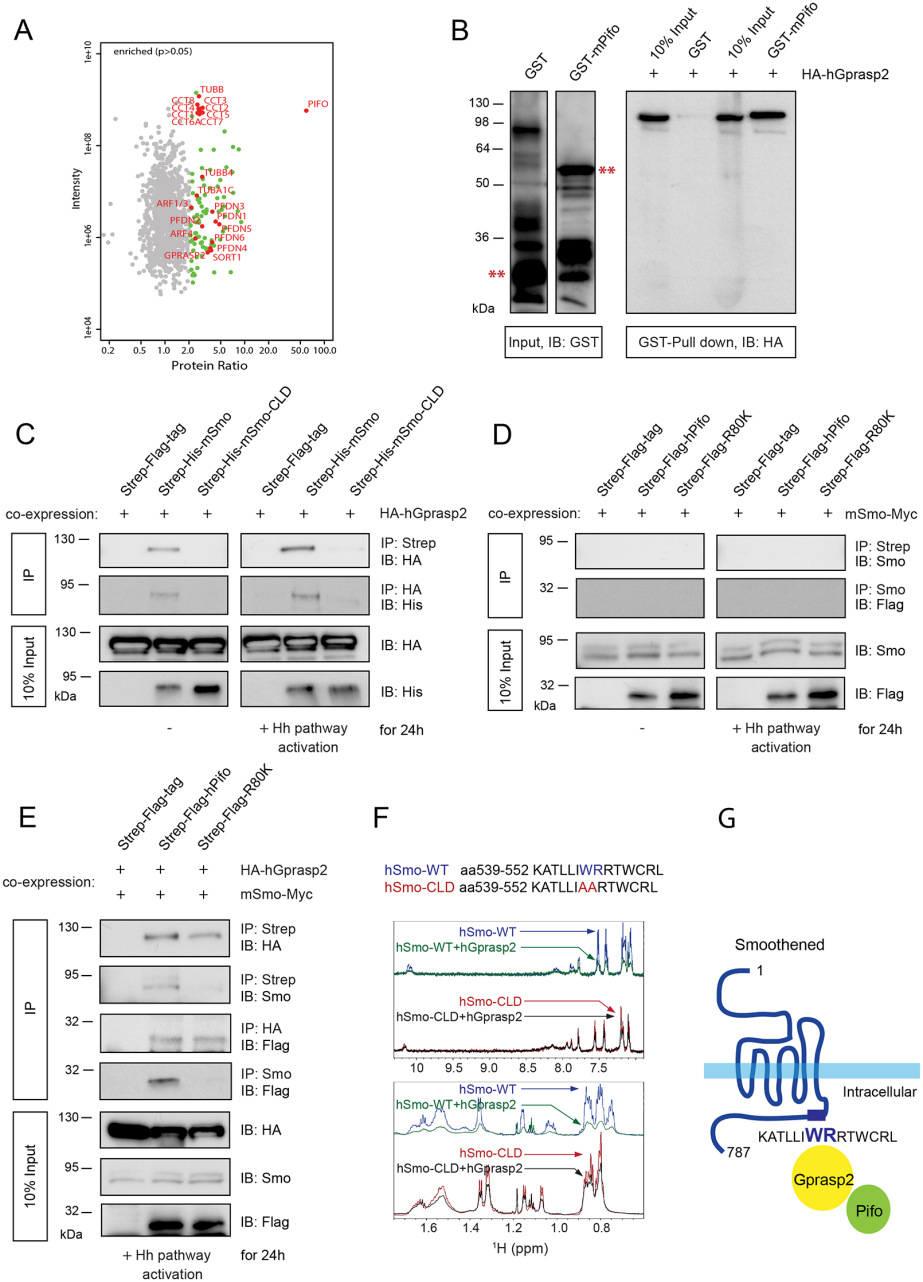
Smo, Gprasp2, and Pifo form a multimeric complex upon Shh signaling. (**A**) Scatter plot of Pifo interactome. Green dots represent significant enriched (p>0.05) Pifo-interaction partners with some highlighted in red. X-axis shows the log2 ratios and Y-axis represents the log2 intensity of each protein. (**B**) Confirmation of protein interaction between Pifo and Gprasp2 in GST pull-down assay. GST and GST-mPifo fusion proteins (left panels, **) were subjected to pull-down *in vitro* translated HA-tagged hGPRASP2 (right panel, GST pull-down). (**C**, **D**, **E**) Determination of Pifo-Gprasp2-Smo multimeric complex formation in co-immunoprecipitation (co-IP). HEK293T cells transiently co-transfected with the indicated expression plasmids were treated with or without SAG (IP: Strep) or Shh (IP: HA and IP: Smo) and the cell lysates were then subjected to immunoprecipitation. Note that input (10%) and protein complexes were detected by immunoblotting with the indicated antibodies (B, C, E, F). (**F**) Confirmation of direct PPI between hSMO and hGPRASP2 by NMR. Zoomed views of aromatic region (top) and the aliphatic region (bottom) of ^1^H NMR spectra of the hSMO-WT and hSMO-CLD peptides were acquired before (blue and red lines) and after addition of hGPRASP2 (green and black lines). (**G**) Schematic illustration of the Smo ciliary targeting complex.

To investigate the function of Pifo we established the Pifo protein-protein interactome (PPI) in primary ciliated cells using quantitative stable isotope labeling by amino acids in cell culture (SILAC). Affinity purification in combination with mass spectronomy of native protein complexes identified novel Pifo interaction partners (Fig 1A and S1 Table), such as α- and β-Tubulin isoforms, the prefoldin chaperon complex (PFDN1-6) and the cytosolic type II chaperonin complex (CCT1-8) facilitating the assembly and transport of axonemal MTs [36], the small GTPase ADP-ribosylation factor 4 (ARF4) involved in the formation of transport carriers that target the 7TMR Rhodopsin to the photoreceptor PC [37], the neuronal anterograde Trk receptor sorting protein Sortilin1 (SORT1) [38], as well as the G protein-coupled receptor (GPCR) trafficking protein (Gprasp2). Gprasp2 interacts with huntingtin [39], which associates with MT motor proteins and is likely to be involved in intracellular vesicular trafficking [40]. The activity of 7TM-GPCRs is regulated by β-arrestins, facilitated by GPCR kinases (Grks) [41] and postendocytic sorting of 7TM-GPCRs into the lysosome depends on Gprasp1 [42]. Activation of the GPCR-like 7TMR Smo causes increased Grk2-dependent phosphorylation and association with β-arrestin 2 [43] that in turn, mediates Kif3a motor protein-driven anterograde transport of Smo into the PC for Hh pathway activation [30]. The translocation of Smo to the PC upon Hh induction depends on a conserved hydrophobic and basic residue ciliary targeting motif (CTM) that is conserved in several 7TMRs, including Rhodopsin [22]. Strikingly, Gprasp1 and 2 bind to the exact same CTM in several 7TM-GPCRs [44]. Thus the results of the PPI suggested that Pifo and Gprasp2 might traffic the 7TMR Smo into the PC.

To test this idea, we first confirmed that human GPRASP2 (hGPRASP2) and mouse Pifo (mPifo) interact in GST-pull down assays (Fig 1B). Co-immunoprecipitation (co-IP) studies in ciliated HEK293T cells in the absence or presence of the Hh pathway agonist SAG or Shh [45] confirmed this *in vitro* observation and further revealed that mouse Smo (mSmo), but not ciliary localization defective Smo (mSmo-CLD) [22], efficiently precipitated over-expressed hGPRASP2 in a CTM-dependent fashion (Fig 1C). NMR studies further demonstrated that a peptide from human SMO (hSMO-WT, aa 539–552) directly interacts with hGPRASP2 in a CTM-dependent manner (Fig 1F). Addition of hGPRASP2 to hSMO-WT peptide in a 1:25 molar ratio (hGPRASP2:hSMO-WT) causes severe line broadening and decrease in intensity of the proton NMR spectra of the free peptide, consistent with the formation of higher molecular weight complexes between hGPRASP2 and hSMO-WT peptide. In contrast, an analogous addition of hGPRASP2 to hSMO-CLD peptide containing ciliary localization defective Smo leads to only marginal line broadening and intensity decrease of the peptide NMR spectra, indicative of a much weaker interaction. In contrast to Gprasp2, co-IP studies showed that human PIFO (hPIFO) and a pathogenic isoform (hPIFO R80K) [5] did not precipitate Smo under these conditions (Fig 1D). While co-transfection of hGPRASP2 resulted in an interaction between mSmo and hPIFO, no interaction was seen between mSmo and hPIFO R80K (Fig 1E) suggesting that a heterotrimeric ciliary targeting complex can be formed in which Gprasp2 bridges between Smo and Pifo (Fig 1G).

### Shh induces the formation of a Smo-Gprasp2-Pifo ciliary targeting complex

**Fig 2:**
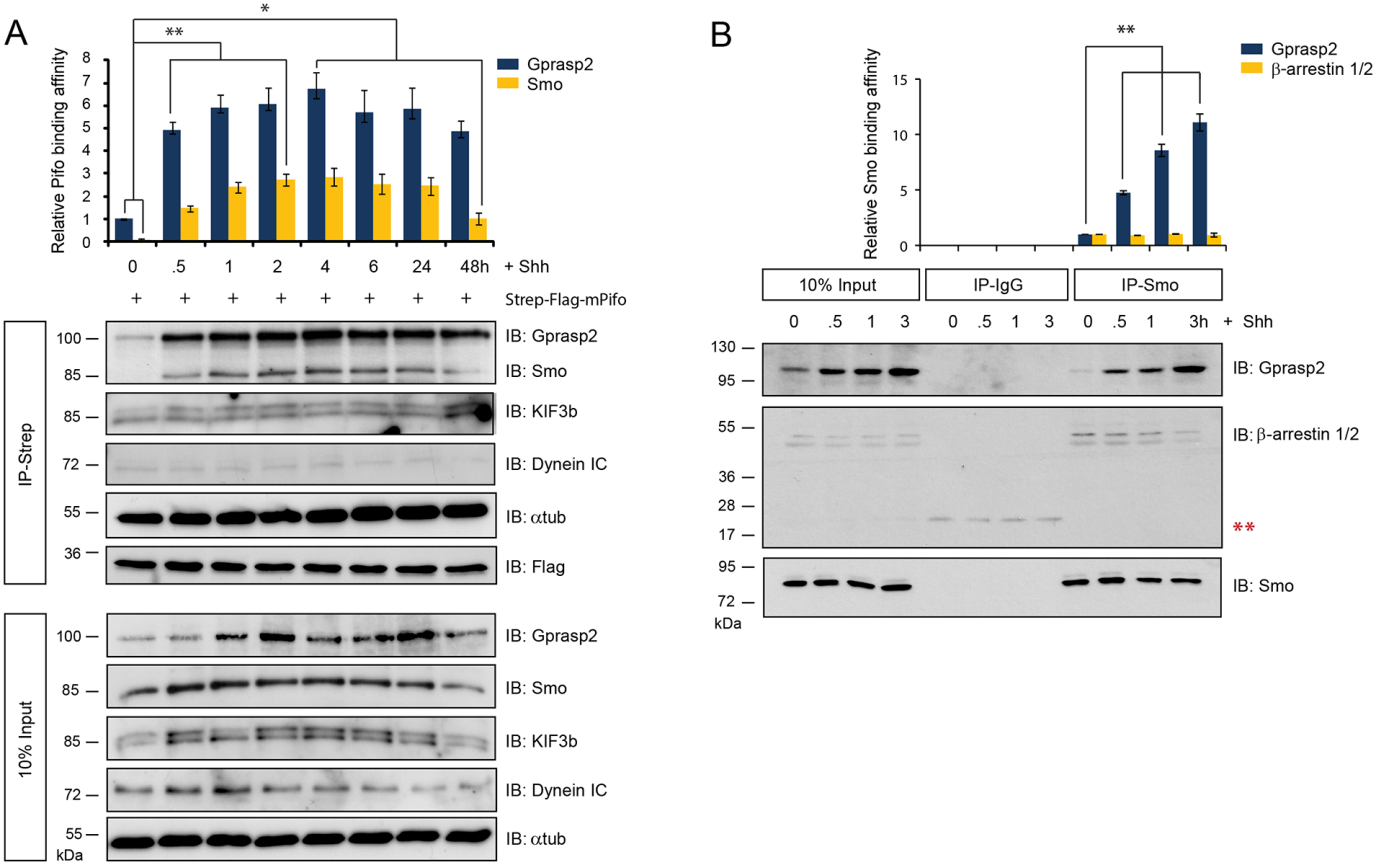
Shh signaling induces Smo ciliary targeting complex formation. (**A**) Kinetics of Smo-Gprasp2-Pifo multimeric complex formation upon Shh treatment. Strep-Tactin sepharose-coupled mPifo was subjected to time-resolved affinity precipitations of native protein complexes formed upon Shh stimulation in PLCs. (**B**) Time-resolved endogenous co-IP of the Gprasp2-Smo ciliary targeting complex upon Shh stimulation in PLCs. Lysates from cells exposed to Shh for different time periods were subjected to immunoprecipitation with Anti-Smo antibody-conjugated beads. Immunoprecipitation with isotype-specific IgG antibodies served as negative control (** indicates non-specific bands). After immunoprecipitation, subsequent immunoblotting with the indicated antibodies determined dynamic changes in protein interactions (A, B). β-arrestin 1 and 2, well-known Smo interacting proteins, were used as a positive control. All error bars indicate the mean ± SD of three independent experiments. Data were analyzed using a two tailed unpaired *t*-test (* = p<0.1, ** = p<0.01).

We next tested whether the Smo-Gprasp2-Pifo interaction requires Shh signaling. For this purpose, we performed affinity-precipitation experiments using immobilized mPifo and lysates of ciliated mouse primary limb bud cultures (PLCs) in which we followed multiprotein complex formation in a time-resolved manner upon Shh induction (Fig 2A). In the absence of Shh, mPifo interacts with endogenous α-Tubulin, the anterograde Kif3b and retrograde Dynein IC motor protein subunits and Gprasp2 (Fig 2A; IP-Strep; 0 h). Upon Shh stimulation, the affinity of Smo and Gprasp2 to Pifo increased markedly within the first 30 min (Fig 2A; IP-Strep; 0.5 h), while total levels remained stable (Fig 2A and 2B; 10% Input). Multimeric complex formation reached peak levels within 1–4 h and then declined by 48 h of ligand exposure (Fig 2A; IP-Strep; 0.5 to 48 h). Next, we compared the Shh-dependent endogenous interactions of Smo with Gprasp2 and the Smo ciliary sorting protein β-arrestin 1 and 2 [30] in PLCs. While both endogenous β-arrestin 1 and 2 and Gprasp2 were co-IPed with Smo, Gprasp2 showed increased affinity upon Shh stimulation (Fig 2B). These results indicate that Shh induction initiates the formation of a Smo ciliary transport complex by increasing the affinity of Smo to Gprasp2-Pifo-Kif3b.

### Pifo is required for Shh target gene activation and Smo ciliary translocation

**Fig 3:**
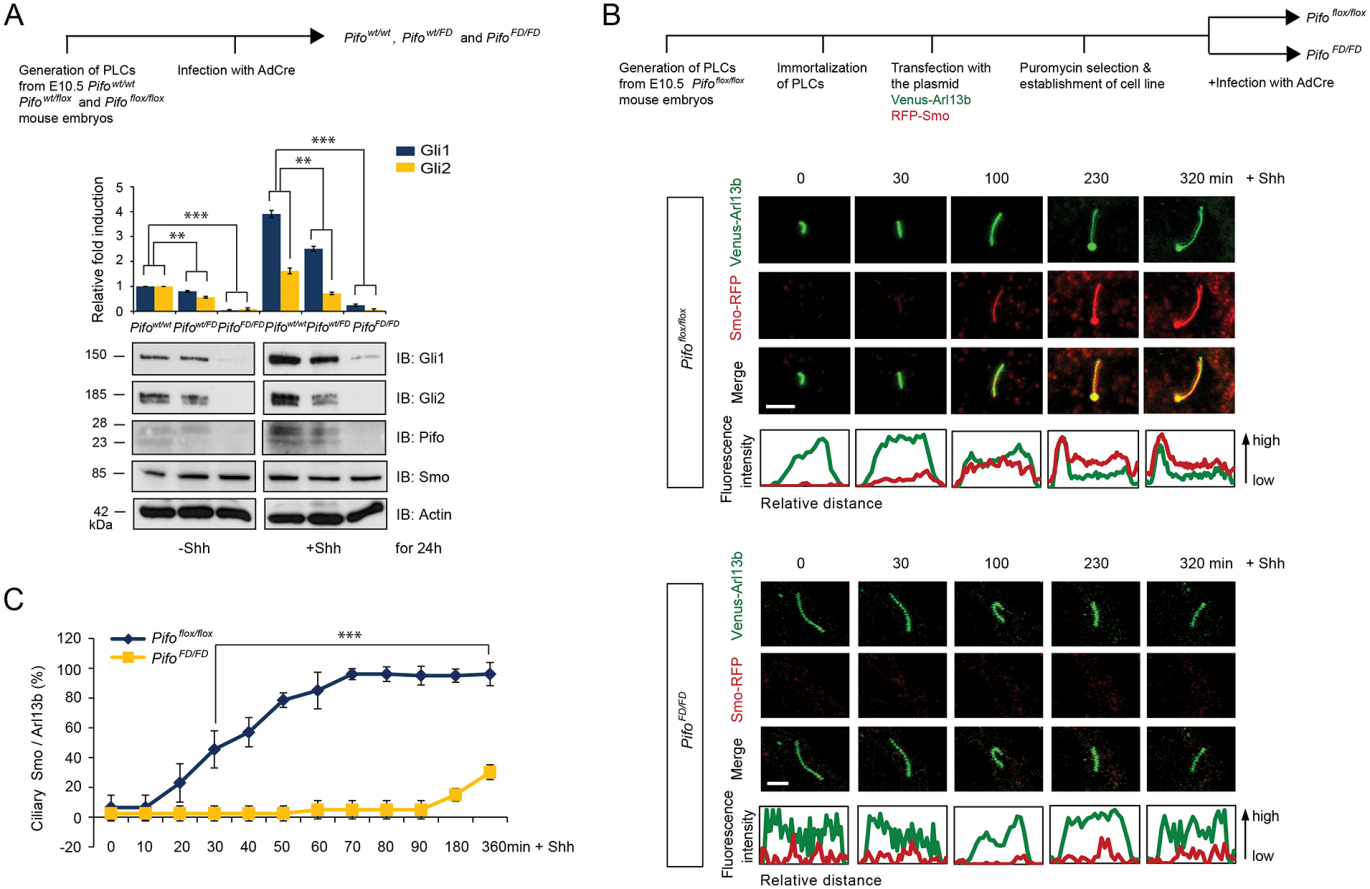
Pifo is necessary for Shh target gene activation and translocation of Smo into the PC. (**A**) The levels of Gli1 and Gli2 protein in *Pifo*^*wt/wt*^, *Pifo*^*wt/FD*^, and *Pifo*^*FD/FD*^ PLCs treated with or without Shh. Graphs represent quantification of immunoblot data that show the mean fold change of protein normalized to Actin levels. (**B**) Immortalization and generation of stably transfected *Pifo*^*flox/flox*^ PLCs to analyze the requirement of Pifo in Smo ciliary translocation by quantitative live-cell imaging. Selected still images (**B**) and quantification (**B**, **C**) of confocal time-lapse movies of *Pifo*^*flox/flox*^ PLCs and *Pifo*^*FD/FD*^ PLCs stably expressing Venus-tagged Arl13b and RFP-tagged Smo. Scale bar = 5 μm. >100 cilia per condition were analyzed. All error bars indicate the mean ± SD of three independent experiments. Data were analyzed using a two tailed unpaired *t*-test (** = p<0.01, *** = p<0.001)

Next we checked whether depletion of Pifo and Gprasp2 results in a lack of Smo translocation to the PC and failure of Shh target gene induction. Recently, we reported that Pifo is necessary for PC disassembly, but not assembly [5]. As structural defects of PC can interfere with Shh signaling [13] we used an inducible approach to specifically delete Pifo after cell-cycle exit and PC assembly. For this purpose we generated a conditional Pifo allele and derived wild type (*Pifo*^*wt/wt*^), heterozygous (*Pifo*^*wt/flox*^) and homozygous floxed (*Pifo*^*flox/flox*^) PLCs for conditional adenovirus Cre recombinase (AdCre)-mediated gene deletion (*Pifo*^*FD/FD*^) (S1 and S2 Figs). The conditional knock-out (CKO) strategy allowed us to rapidly and completely delete Pifo in PLCs, which we confirmed on the DNA, mRNA and protein level (S2 and S3 Figs). Furthermore, detailed immunocytochemistry combined with laser scanning microscopy (LSM) revealed equal number and normal structure of PC in *Pifo*^*flox/flox*^ and *Pifo*^*FD/FD*^ PLCs after cell-cycle exit (S3 Fig), suggesting that Pifo is not required to build and maintain PC [5]. Interestingly, deletion of one or two copies of Pifo (*Pifo*^*wt/FD*^ and *Pifo*^*FD/FD*^) in primary ciliated PLCs revealed that Shh-induced Gli1 and Gli2 mRNA and protein expression requires Pifo in a concentration-dependent manner (Fig 3A and S3 Fig). To further analyze the requirement for Pifo in Shh-induced Smo translocation to the PC, we developed a live-cell imaging system using LSM. Therefore, we first immortalized *Pifo*^*flox/flox*^ then stably transfected PLCs with a Venus fluorescent protein-tagged Arl13b small GTPase marker protein for the PC (Venus-Arl13b) and a Red fluorescent protein-tagged version of the 7TMR Smo (RFP-Smo; Fig 3B). Quantitative live-cell imaging of primary ciliated cells revealed that Pifo is necessary for the rapid translocation of RFP-Smo into the PC upon Shh stimulation (Fig 3B and 3C).

**Fig 4:**
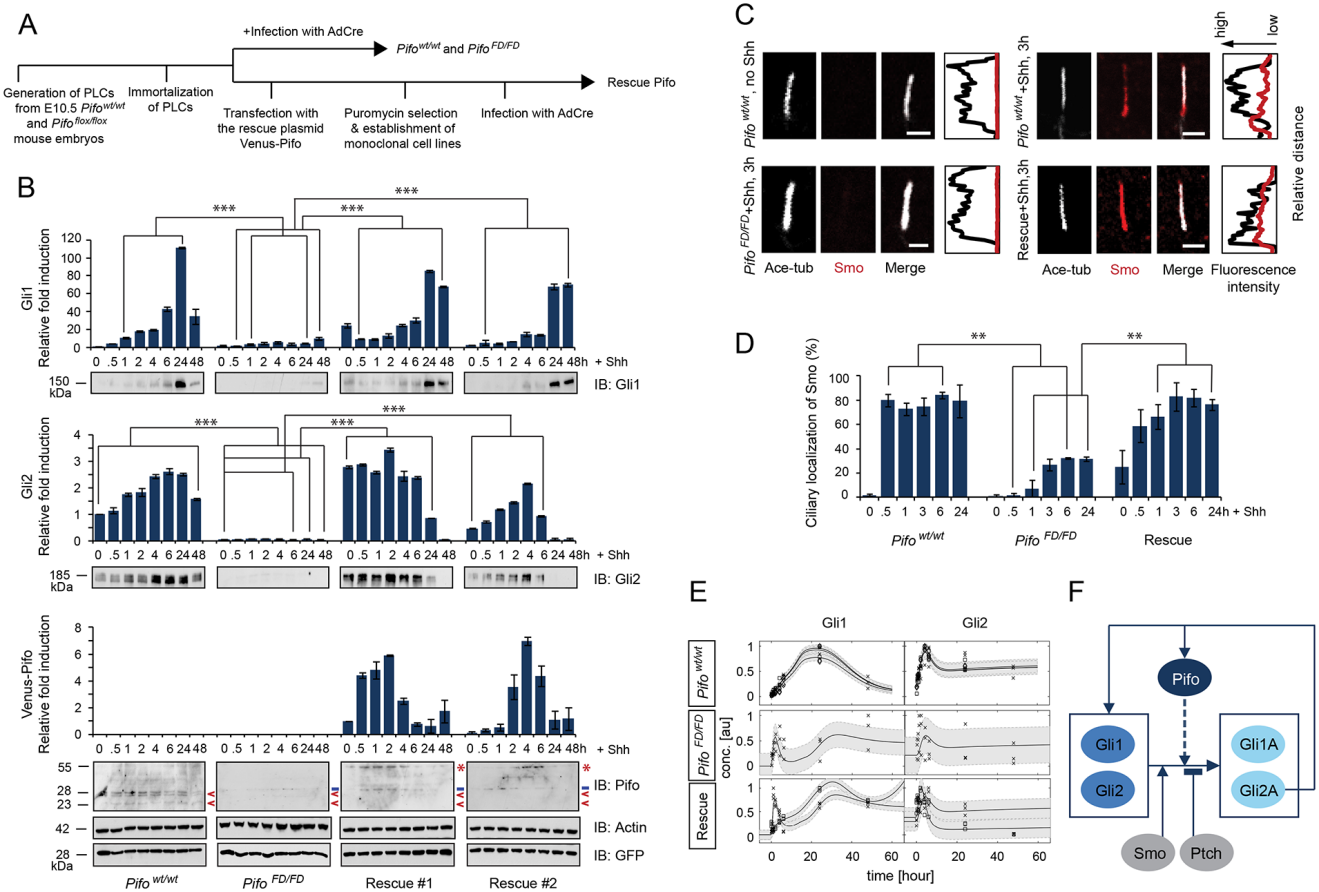
Pifo is necessary and sufficient for ciliary localization of Smo and Shh target gene activation. (**A**) Experimental scheme for generating Pifo conditional knock-out and Venus-Pifo rescued cell lines. (**B**) Induction kinetics of Gli1 and Gli2 protein in *Pifo*^*wt/wt*^, *Pifo*^*FD/FD*^, and two independent Venus-Pifo rescued PLC cell lines upon Shh stimulation. Note that expression levels of the endogenous (**<**) and Venus-tagged Pifo (*) were detected by α-Pifo (- indicates non-specific bands). Protein loading was controlled by Actin levels. Transduction efficiency of AdCre was evaluated by α-GFP. Mean ± SD of three independent experiments. Representative confocal image (**C**) and quantification (**D**) of ciliary Smo in *Pifo*^*wt/wt*^, *Pifo*^*FD/FD*^, and rescued PLCs. Scale bar = 2 μm. >100 cilia per condition were analyzed. All error bars indicate the mean ± SD of three independent experiments. Data were analyzed using a two tailed unpaired *t*-test (** = p<0.01, *** = p<0.001). (**E**) The fitted Pifo+ model dynamics (solid line) of Gli1 and 2 for the three different conditions (*Pifo*^*wt/wt*^, *Pifo*^*FD/FD*^ and Rescue) relative to Shh activation (t = 0). Black circles, boxes and crosses denote experimental data in replicates, arbitrary units. Gray areas denote the confidence intervals for the parameter estimation. (**F**) Scheme showing hypothesized influence of Pifo on Shh target genes, Gli1 and 2, (dotted line) used for the mathematical model. Smo and Pifo support activation of Gli1 and 2, but Ptch inhibits the activation. Activated Gli1 and 2 (Gli1A and Gli2A) support both their own and Pifo expression levels.

To analyze the detailed kinetics of the Pifo requirement for Gli1 and Gli2 activation, we generated immortalized *Pifo*^*wt/wt*^ and *Pifo*^*FD/FD*^ PLCs and rescued Pifo deficiency in *Pifo*^*FD/FD*^ PLCs by different levels of stable Venus-Pifo expression (Fig 4A and 4B). Activation of both Gli1 and Gli2 are almost completely abolished in *Pifo*^*FD/FD*^ PLCs (Fig 4B). Importantly, rescue of the Pifo levels in two independent PLC lines demonstrates that Gli1 and Gli2 activation depends on absolute levels and timing of Venus-Pifo accumulation (Fig 4B; Rescue #1 and #2). We further confirmed that failure of Shh target gene induction was caused by insufficient Smo translocation to the PC. Whereas 70–80% of *Pifo*^*wt/wt*^ PLCs show efficient Smo translocation to the PC within 30 min of ligand exposure, *Pifo*^*FD/FD*^ PLCs with normally formed PC barely responded to SAG induced pathway activation, which can be sufficiently rescued by stable Venus-Pifo expression (Fig 4C and 4D). Together, these results demonstrate that Pifo is necessary and sufficient for Smo translocation to the PC and Gli1 and Gli2-mediated Shh pathway activation.

### Kinetic modeling supports Pifo mediated enhancement of Shh signaling

To test the influence of Pifo on the Shh signaling pathway we performed in-silico modeling using ordinary differential equations (ODEs). By comparing two different model versions, we found evidence that Pifo functions as an enhancer on Shh signaling. The core for both models consists of a regulatory feedback loop where Gli 1 and 2 proteins promote the transcriptional activation of Gli1 and 2 genes. Smo functions as an activator for Gli1 and 2 proteins whereas Ptch inhibits these. One version of our model includes Pifo as an additional activator for Gli1 and 2 proteins (Pifo+), whereas the other version lacks this interaction (Pifo-) (Fig 4F, dotted line). Both models parameters were simultaneously fitted to three different data sets based on three conditions, *Pifo*^*wt/wt*^, *Pifo*^*FD/FD*^, and rescue data. The Pifo+ model was able to consistently replicate the given protein kinetics with high precision (Fig 4E and S1 Supporting Information). In contrast the Pifo- model was not able to replicate the dynamics of the data (S1 Supporting Information). The models were compared using Akaike Information Criterion (AIC) scores (Pifo+: -408.65; Pifo-: -160.76). In conclusion, mathematical modeling clearly supports an autoregulatory feedback mechanism of the Shh pathway by enhancing target gene activation.

### Gprasp2 is required for Shh target gene activation and Smo ciliary translocation

**Fig 5:**
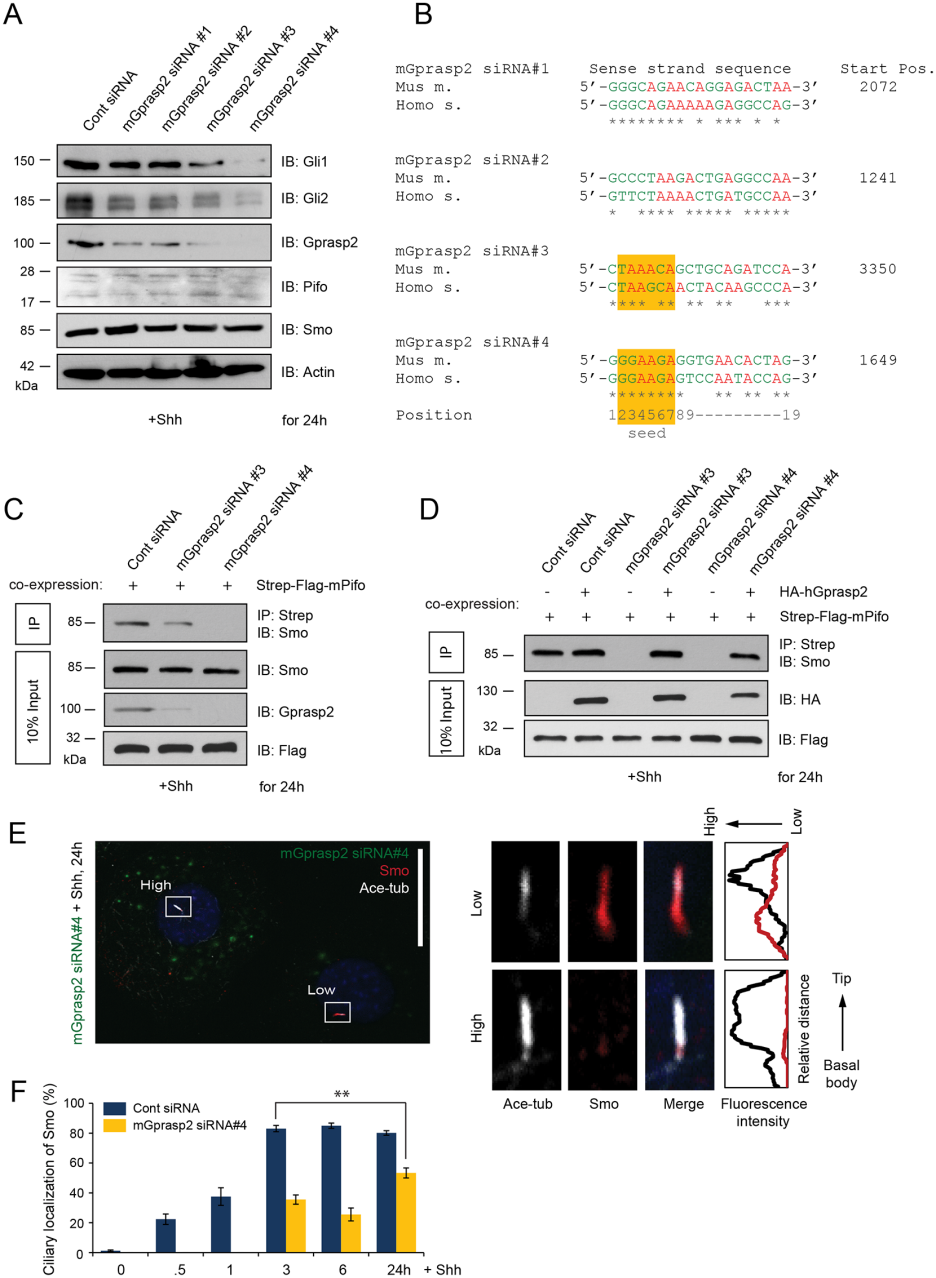
Gprasp2 is required for ciliary localization of Smo and Shh target gene activation. (**A**) Shh-induced protein levels after 48h of siRNA-mediated knock-down of Gprasp2 in PLCs. (**B**) Mouse Gprasp2 siRNA target sequences with Multiple species ClustalW alignment to human and mouse Gprasp2 and indicated siRNA seed region position. Determination of Smo-Pifo complex formation after depletion of mGprasp2 (**C**) and rescue of mGprasp2 depletion by introducing hGPRASP2 (**D**). 48h post-transfection with the indicated siRNA duplexes or co-transfection with the indicated siRNA duplexes and HA-tagged hGPRASP2, PLCs were stimulated with Shh and the lysates were subsequently subjected to Strep-Tactin sepharose-coupled mPifo. Input (10%) and endogenous Smo protein complexes were determined by immunoblotting with the indicated antibodies. Representative confocal image (**E**) and quantification (**E**, **F**) of endogenous ciliary Smo after 48 h of siRNA-mediated depletion of mGprasp2 in PLCs. Note that levels of Alexa Fluor 488 (high and low) correlates with different Gprasp2 knock-down efficiency. Scale bar = 25 μm. >100 cilia per condition were analyzed. All error bars indicate the mean ± SD of three independent experiments. Data were analyzed using a two tailed unpaired *t*-test (** = p<0.01).

Next we investigated Gprasp2 function in Shh signaling using RNAi in PLCs (Fig 5A and 5B). First, we confirmed that PC assembly and structure was not affected (S3 Fig) after efficient knock-down of Gprasp2 (Fig 5A; siRNA#4). Following 24 h Shh treatment, Gli1 and Gli2, but not Pifo and Smo activation, required Gprasp2 in a concentration-dependent fashion (Fig 5A). Knock-down of Gprasp2 in PLCs stimulated with Shh for 24 h prevented affinity precipitation of endogenous Smo by immobilized mPifo (Fig 5C), consistent with the idea that Gprasp2 bridges a heterotrimeric Smo-Gprasp2-Pifo complex. Over-expression of hGPRASP2 that is targeted by siRNA#4 and siRNA#3 rescued the protein-protein interactions (Fig 5D). Finally, we show that high levels of Gprasp2 knock-down correlates with a profound reduction of Smo and Pifo translocation to the PC using static (Fig 5E and 5F and S4 Fig) and live-cell imaging (S5 Fig). Taken together, these results indicate that the Smo-Gprasp2-Pifo ciliary targeting complex is necessary for pathway activation.

### Pifo and Gprasp2 are rapidly induced by Hh signaling for pathway activation

**Fig 6:**
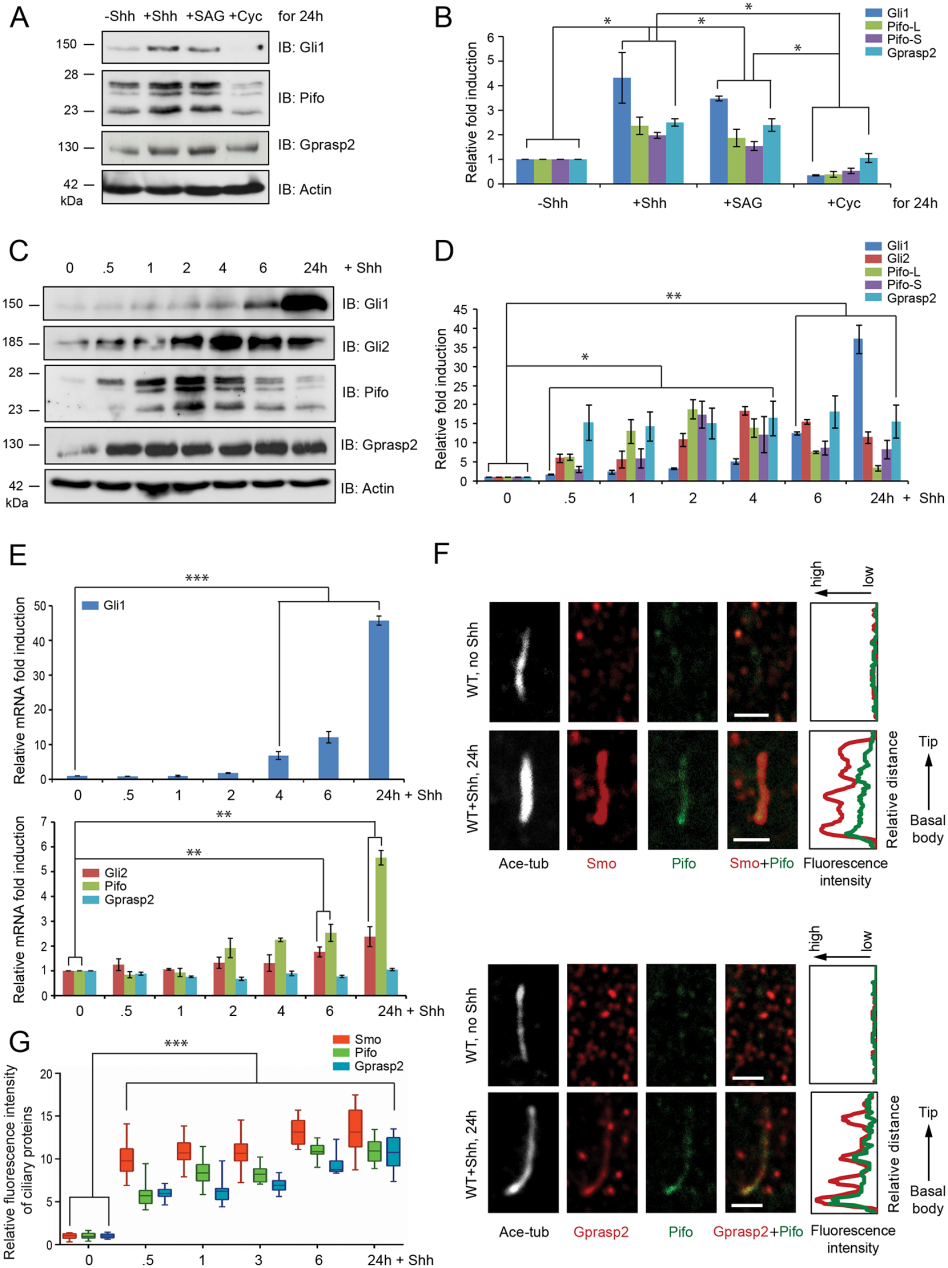
Rapid Shh induction of Pifo, Gprasp2 and Gli2 is regulated on transcriptional and post-translational level. (**A**) The levels of Gli1, Pifo and Gprasp2 protein after treatment with Shh, SAG, or Cyc in PLCs. Graphs (**B**, **D**) represent quantification of immunoblot data that show the mean fold change of protein expression normalized to Actin levels. Characterization of induction kinetics of Gli1, Gli2, Pifo, and Gprasp2 proteins (**C**) and mRNAs (**E**) in response to Shh stimulation in PLCs. Representative confocal images for co-localization analysis of ciliary Pifo and Smo or Gprasp2 (**F**) and quantification (**G**) of ciliary accumulation in response to Shh stimulation. >100 cilia per condition were analyzed. All error bars indicate the mean ± SD of three independent experiments. Data were analyzed using a two tailed unpaired *t*-test (B, D, E) or a two way ANOVA (G) (* = p<0.1, ** = p<0.01, *** = p<0.001)

Finally, we examined whether Pifo and Gprasp2 differential regulation and PC localization is orchestrated by Shh signaling. For this purpose, we compared the induction kinetics of Pifo and Gprasp2 with the well-known Shh target gene Gli1 in PLCs. Both Shh ligand and the Hh pathway agonist SAG treatment for 24 h induced Gli1, Pifo and Gprasp2 proteins, whereas the Hh pathway antagonist Cyclopamine (Cyc) blocked their activation (Fig 6A and 6B). Next, we compared the induction kinetics of Pifo and Gprasp2 to Gli1 and Gli2 in a time-resolved fashion upon Shh stimulation. This revealed that Gli2, Pifo and Gprasp2 showed a faster response to Shh compared to Gli1 (Fig 6C and 6D). To further understand this regulation, we investigated the dynamic transcriptional activation of these genes using quantitative PCR (qPCR). This revealed that Gli1, Gli2 and Pifo are induced to different degrees by Shh signaling, whereas Gprasp2 was not (Fig 6E). The comparison of the protein and mRNA induction kinetics clearly indicates that Gli2, Gprasp2 and Pifo quickly accumulate through post-transcriptional mechanisms (Fig 6C and 6E). In contrast to Smo, which is not present at the PC before Hh signaling (Figs 3 and 5), both Gprasp2 and Pifo were detectable at low levels, which increased upon Hh signaling (Fig 6F and 6G). As total Smo remains stable over time (Fig 2) the rapid translocation (<30 min) of Smo into the PC can only be caused by post-translational modifications and increased affinity of Smo ciliary targeting components. Together, these data indicate that Pifo and Gprasp2 are components of an Hh-induced ciliary targeting complex that is regulated on transcriptional and post-translational level to facilitate signaling at the PC.

## Discussion

**Fig 7:**
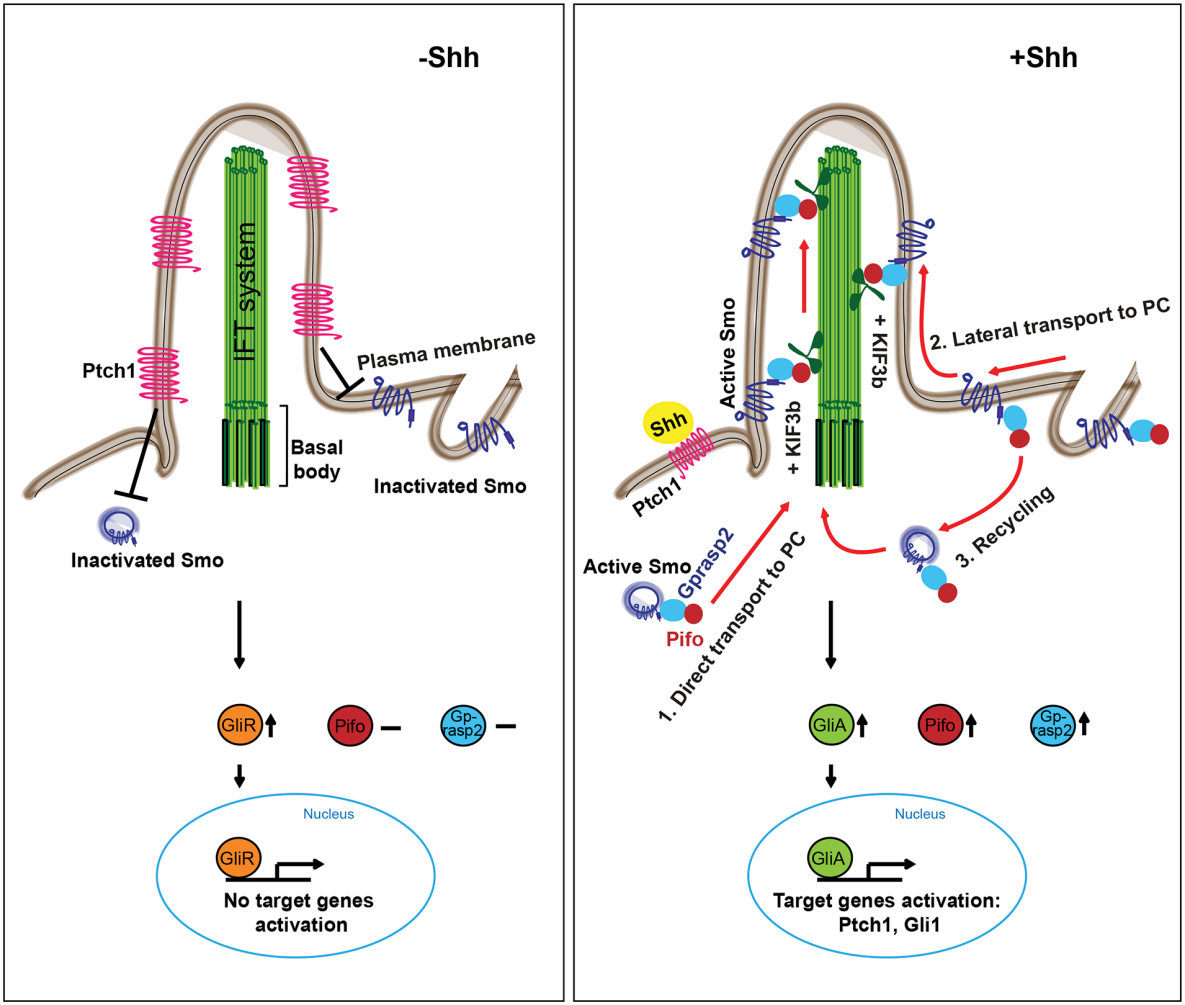
Hypothetical model of Smo transport and regulation by Pifo and Gprasp2 upon Shh signaling. In the absence of Shh signaling (**left**), Ptch1 localizes to the PC and suppresses Smo translocation to PC. The proteolytically processed repressor forms of Gli (GliR) accumulate and enter the nucleus to inhibit target gene expression. In the presence of Shh (**right**), Pifo and Gprasp2 rapidly form a Smo ciliary targeting complex. This can be transported to the PC from 1. intracellular pools or 2. via lateral plasma membrane transport or 3. via the recycling endosome pathway. Smo transport into the PC is mediated through the kinesine II motor complex leading to accumulation of Gli activator forms (GliA) in the cytoplasm and nucleus for Shh target gene activation.

The movement of the 7TMR Smo into the PC controls all Hh pathway activation and ultimately triggers cellular responses, such as differentiation and proliferation. Thus, a thorough understanding of the cellular and molecular regulation of Smo activity is key to decipher Hh pathway activation in development, regeneration and disease. Here we have identified Pifo and Gprasp2 as novel regulators of Smo ciliary translocation and Hh pathway activation. Interestingly, Pifo and Gprasp2 appear only within the chordate and vertebrate lineage, respectively (S6 Fig) [5], indicating that these genes are likely evolutionary adaptations to PC-dependent Hh signaling [19]. Mechanistically, both Pifo and Gprasp2 are functionally important components of the Smo ciliary targeting complex (Fig 7), as lack of either Pifo or Gprasp2 can lead to failure of Smo ciliary translocation and Shh target gene activation in cells with structurally normal PC. In the absence of Hh signaling, Ptch1 localizes to the PC and suppresses Smo by unknown mechanisms. Smo remains inactive in the cytoplasm and plasma membrane (PM) and cannot enter the PC compartment [20, 46]. Upon Hh binding, Smo is activated by post-transcriptional modifications and structural changes [43, 47]. This leads to an increased affinity to Kif3b, Pifo and Gprasp2 (this study), as well as Kif3a and β-arrestins [30] for rapid translocation into the PC compartment. But how is this ciliary targeting machinery regulated and via which route is Smo transported upon Hh signaling?

Elegant microscopy-based pulse-chase experiments shed light on Hh-induced Smo trafficking to the PC and suggest three different routes of transport (Fig 7) [46]. First, MT-dependent directed transport of Smo intracellular vesicles from the Golgi to the base of the PC. Second, transport of PM-localized Smo laterally into the PC compartment breaking a diffusion barrier. Third, endocytosis and vesicular recycling of PM-localized Smo for translocation into the PC. The authors provided experimental evidence for the first two modes of trafficking and suggested that PM-derived Smo enters the PC faster by lateral than by intracellular transport. They also measured half-life time of Smo with around 2 h, suggesting that newly synthesized Smo needs to be delivered from the endoplasmatic reticulum (ER) to the base of the PC for sustained Hh pathway activity. These distinct routes of transport and the fast turnover rate of Smo imply that different molecular machineries are important for surface and intracellular Smo transport. We identified the Smo-Gprasp2-Pifo complex as one of these machineries involved in Hh-induced ciliary targeting.

Careful time-resolved and quantitative protein and mRNA analysis combined with live-cell imaging and mathematical modeling allowed us to unravel some details of the cellular and molecular regulation of Smo targeting to and into the PC. Our data suggests that the Smo-Gprasp2-Pifo complex is regulated dynamically on the post-transcriptional level. Before Hh induction, Pifo and Gprasp2 are present in the cell at low quantities. The proteins do physically interact with each other, small GTPases and MT-motor proteins, but not with Smo. This leads to low-level accumulation in the PC where Smo is absent. Upon the first minutes of Hh induction, Smo strongly increases its affinity to Pifo and Gprasp2 for rapid translocation into the PC. Interestingly, several studies indicate that phosphorylation mediated by CK1a and Grk2 induces an active conformation of Smo [43, 47] that, in turn, mediates β-arrestins-Kif3a [30] and Gprasp2-Pifo-Kif3b motor protein-driven anterograde transport of Smo into the PC. This is very likely regulated via the accessibility of the Smo ciliary targeting motif [22] and we provide evidence that the Smo-Gprasp2 protein interaction depends on this motif. At this early phase of Smo translocation only small variations in the total protein amount of Smo, Gprasp2 or Pifo is detectable. This indicates that post-translational modification and increased affinity of these ciliary targeting complex components mobilizes existing pools of Smo very likely from PM pools by lateral migration and/or endocytosis-mediated 7TMR recycling [46]. Although Rohatgi and colleagues provided evidence to excluded endocytosis-mediated recycling as an optional route of Smo trafficking, the involvement of β-arrestins [30, 41] and Gprasp1 in postendocytic sorting of 7TM-GPCRs [48] would suggest otherwise, which needs further clarification.

After this first rapid phase of Smo translocation into the PC, increased levels of Pifo and Gprasp2 might additionally promote and secure ciliary targeting of Smo. In this second phase, proteins stored in intracellular pools and newly synthesized in the Golgi and ER are directed via the exocytotic pathway to the base of the PC [2, 49]. As Pifo and Gprasp2 interact with anterograde and retrograde MT-motor proteins and small GTPases involved in ciliary trafficking (this study) [5], this result in intracellular transport of the Smo ciliary targeting complex to and into the PC. Interestingly, the kinetics of Pifo and Gprasp2 accumulation and ciliary targeting complex formation in the hour range is consistent with the arrival of intracellular pools of Smo at the PC [46], indicating that different trafficking machineries recruit distinct pools of Smo for Hh pathway activation.

Additionally, we noticed that the increase of Pifo and Gprasp2 precedes the transcriptional activation of Gli1, Gli2 and Pifo mRNAs. This strongly suggests that in the absence of Hh signaling, Pifo and Gprasp2 are earmarked for proteasomal degradation. It is possible that the same kinases and SCF (Skp1-Cul1-F-box protein) E3 ubiquitin-ligase complex involved in degradation and proteolytic cleavage of the Gli transcription factors might also regulate Pifo and Gprasp2 [50]. This is supported by the fact that 1. Skp1 and Cul1 were identified as potential Pifo interaction partners (S1 Table) and that 2. endogenous Pifo and Gli2 show comparable kinetics of protein synthesis and stabilization, similar to what was recently observed for exogenous Gli2 [29]. Interestingly, although Gli2 is generally not assumed to be an Hh target gene [51], we found that both Pifo and Gli2 are activated, similar to what was previously found for Gli2 (Fig 6) [50]. Taken together, both post-transcriptional and translational mechanisms lead to the assembly and accumulation of an Hh-induced Smo ciliary targeting complex that in turn leads to the activation of Hh transcriptional program.

Taken together we have identified Pifo and Gprasp2 as critical components of the Smo ciliary targeting complex that likely translocates to the PC via different routes and is regulated by different mechanisms. Interestingly, Reiter and colleagues have recently reported on small molecule inhibitors of Smo that either disrupts slow intracellular trafficking or fast lateral PM entry of Smo into the PC [52]. This emphasizes that the mechanisms underlying Smo trafficking to and into the PC are different and that full pathway activation might require mobilization of different pools of Smo. Certainly, the identification of novel molecular machineries that regulate Smo translocation into the PC will identify novel drug targets for cancer therapy.

## Materials and Methods

### Ethics Statement

All mice were housed in the central facilities at HMGU in accordance with the German animal welfare legislation and acknowledged guidelines of the Society of Laboratory Animals (GV-SOLAS) and of the Federation of Laboratory Animal Science Associations (FELASA). Scarification of mice at embryonic stages was not subject to regulatory authorization.

### Cell culture for SILAC experiments

For SILAC experiments, HEK293T cells were grown in SILAC DMEM (PAA) supplemented with 3 mM L-Glutamine (PAA), 10% dialyzed fetal bovine serum (PAA), 0.55 mM lysine and 0.4 mM arginine. Light SILAC medium was supplemented with ^12^C_6_, ^14^N_2_ lysine and ^12^C_6_, ^14^N_4_ arginine. Heavy SILAC medium was supplemented with either ^13^C_6_ lysine and ^13^C_6_, ^15^N_4_ arginine or ^13^C_6_, ^15^N_2_ lysine and ^13^C_6_, ^15^N_4_ arginine. 0.5 mM proline was added to all SILAC media to prevent arginine to proline conversion [53]. All amino acids were purchased from Silantes. Cells were seeded, grown overnight and then transfected with the corresponding SF-TAP-tagged [54] DNA constructs using PEI reagent (Polysciences) [55]. 48 hours later, cells were harvested in lysis buffer containing 0.5% Nonidet-P40 (NP-40), protease inhibitor cocktail (Roche), and phosphatase inhibitor cocktails II and III (Sigma-Aldrich) in TBS (30 mM Tris-HCl, pH 7.4, and 150 mM NaCl) for 20 minutes at 4°C. Cell debris and nuclei were removed by centrifugation at 10,000 g for 10 minutes.

### Affinity purification of protein complexes

For one-step Strep purifications, SF-TAP tagged proteins and associated protein complexes were purified essentially as described earlier [54, 56]. Human embryonic kidney cells (HEK293T), transiently expressing the SF-TAP tagged constructs were lysed in lysis buffer containing 0.5% Nonidet-P40, protease inhibitor cocktail (Roche) and phosphatase inhibitor cocktails II and III (Sigma-Aldrich) in TBS (30 mM Tris-HCl, pH 7.4, 150 mM NaCl), for 20 min at 4°C. After sedimentation of nuclei at 10,000 × g for 10 min, the protein concentration was determined by a Bradford assay before equal amounts of each lysate were transferred to Strep-Tactin-Superflow beads (IBA) and incubated for 1 h. Then the resin was washed three times with washing buffer (0.1% NP-40, phosphatase inhibitor cocktail II and III in TBS). The protein complexes were eluted by incubation for 10 min in Strep-elution buffer (IBA). The eluted samples were concentrated using 10 kDa cut-off VivaSpin 500 centrifugal devices (Sartorius Stedim Biotech) and pre-fractionated using SDS-Page and in-gel tryptic cleavage as described elsewhere [57].

### Mass spectrometry and data analysis

LC-MS/MS analysis was performed on an Ultimate3000 nano RSLC system (Thermo Fisher Scientific) coupled to a LTQ Orbitrap Velos mass spectrometer (Thermo Fisher Scientific) by a nano spray ion source. Tryptic peptide mixtures were automatically injected and separated by a linear gradient from 5% to 40% of buffer B in buffer A (2% acetonitrile, 0.1% formic acid in HPLC grade water) in buffer A (0.1% formic acid in HPLC grade water) at a flow rate of 300 nl/min over 90 min. Remaining peptides were eluted by a short gradient from 40% to 100% buffer B in 5 min. The eluted peptides were analyzed by the LTQ Orbitrap Velos mass spectrometer. From the high resolution MS pre-scan with a mass range of 300 to 1500, the ten most intense peptide ions were selected for fragment analysis in the linear ion trap if they exceeded an intensity of at least 500 counts and if they were at least doubly charged. The normalized collision energy for CID was set to a value of 35 and the resulting fragments were detected with normal resolution in the linear ion trap. The lock mass option was activated, the background signal with a mass of 445.12002 was used as lock mass [58]. Every ion selected for fragmentation, was excluded for 20 sec by dynamic exclusion. For SILAC experiments, all acquired spectra were processed and analyzed using the MaxQuant software [59] (version 1.0.13.13) and the human specific IPI database version 3.52 (http://www.maxquant.org/) in combination with Mascot (Matrix Science, version 2.2). Cysteine carbamidomethylation was selected as fixed modification, methionine oxidation and protein acetylation were allowed as variable modifications. The peptide and protein false discovery rates were set to 1%. Contaminants like keratins were removed. Proteins, identified and quantified by at least two unique peptides were considered for further analysis. The significance B value was calculated and a p-value of 0.05 was selected as threshold for significant enrichment. Proteins, showing a significant enrichment in the PIFO sample compared to the SF-TAP negative control was considered to be potential PIFO interactors. All other proteins are non-specific binders to the matrix or the tag and are therefore eliminated for further analysis.

### Generation of Pifo targeting vector

5' and 3' homology regions (HR) for Pifo gene were amplified by PCR (for 5’ Ex2 HR: 5’-Pifo Ex2 HR NotI, 5’-Pifo Ex2 HR EcoRI/BamHI; for 5’ Ex3 HR: 5’-Pifo Ex3 HR EcoRI/ApaI, 5’-Pifo Ex3 HR KpnI; for 3’ after Ex6 HR1: 3’-Pifo after Ex6 HR1 NotI, 3’-Pifo after Ex6 HR1 EcoRI/BamHI; for 3’ after Ex6 HR2: 3’-Pifo after Ex6 HR2 EcoRI, 3’-Pifo after Ex6 HR2 KpnI) using BAC DNA from clone RP23-306O20 as a template. The purified 5’ and 3’ HR were cut with NotI/EcoRI (for 5’ Ex2 HR and 3’ after Ex6 HR1) or EcoRI/KpnI (for 5’ Ex3 HR and 3’ after Ex6 HR2) and three-way ligated into NotI and KpnI digested pBluescript KS- (pBKS-), resulting in pBKS-5'-HR and pBKS-3'-HR respectively. First, to insert the single 5' loxP site, the PGK-neo-pA cassette flanked by 2 LoxP sites (excised from pL452) [60] was inserted between EcoRI and BamHI sites located upstream of Exon3 in pKS-5'-HR. This construct was then digested with NotI and KpnI and the fragment (2.2kb) was transformed into the EL250 E. Coli strain containing retrieval of Pifo genomic DNA [5]. The PGK-neo-pA cassette was then removed by transformation into arabionose-induced Cre expressing EL350 cells [60], resulting in the single 5' LoxP site named EL350-Pifo 5’-LoxP. Next, the 3' LoxP site with FRT-flanked PGK-gb2-neo cassette (excised from pL451) [61] was inserted between EcoRI and BamHI sites located downstrem of Exon6 in pKS-3'-HR. The construct was subsequently cut with NotI and KpnI and the fragment (2.2kb) was transformed into the EL350-Pifo 5’-LoxP resulting in a targeting construct for Pifo conditional knockout.

### Homologous recombination in ES cells

Mouse ES cells were cultured as previously described [62]. Pifo targeting construct was linearized with AscI and electroporated into IDG3.2 cells (*S10*). After selection of neomycin resistant clones in the presence of G418 (300 μg/ml, Invitrogen), homologous recombination at the Pifo locus was confirmed by Southern blot analysis of BamHI-digested genomic DNA with the Pifo 3'-probe previously used [5]. Homologous recombined clone was aggregated with CD1 morulae to obtain chimeras and the FRT-flanked PGK-gb2-neo cassette was then removed in the germline by intercrossing the Flp-e expressing mice [63]. The deletion of PGK-gb2-neo cassette was confirmed by PCR using indicated primers: EP558, EP555, and EP212.

### Generation of immortalized PLCs

The dissected forelimbs of E12.5 *Pifo*^*wt/wt*^, *Pifo*^*wt/flox*^ or *Pifo*^*flox/flox*^ were mechanically dissociated by pipetting and subsequently placed in 0.05% Trypsin-EDTA at 37°C for 5 min. 10% FCS was added to the suspension to inhibit Trypsin activity and cells were centrifuged, resuspended and then maintained in standard DMEM medium (Invitrogen), supplemented with 10% FCS, 2 mM L-Glutamine and 1% Penicillin/Streptomycin. For generating the immortalized cell lines, cells were split every 2–3 days until cells underwent crisis after cell division stopped. Spontaneously immortalized cells were able to grow again and maintained in culture. All established polyclonal PLC lines showed apparently similar growth behaviors.

### Generation of conditional Pifo knock-out PLCs

In order to generate Pifo conditional knock-out PLCs, *Pifo*^*flox/flox*^ PLCs were infected with 1.5×10^7^ PFU of recombinant adenovirus expressing CRE recombinase with GFP fluorophore (Vector Biolabs) for various time points. The genotypes of established PLC lines were confirmed by allele-specific PCR with the indicated primers: EP551 and EP554, and the depletion of Pifo protein in PLCs was confirmed by immunoblotting and immunostaining with α-Pifo.

### Establishment of stable PLC lines for the rescue experiment and the live-cell imaging

To obtain stable populations of *Pifo*^*flox/flox*^ PLCs a linearized pCAG-Arl13b-RFP-2A-Venus-Pifo construct was transfected into *Pifo*^*flox/flox*^ PLCs using TransFectin (Bio-Rad). After 48h post-transfection, Venus-tagged Pifo expressing cells were selected with puromycin (1 μg/ml) and individual colonies were picked, expanded and maintained under puromycin selection. The expression of Venus-Pifo was analyzed by immunoblotting with α-Pifo or α-GFP antibodies. To obtain *Pifo*^*flox/flox*^ PLCs stably expressing the RFP-Smo and Venus-Arl13b, the linearized pCAG-RFP-Smo-2A-Venus-Arl13b construct was transfected into *Pifo*^*flox/flox*^ PLCs using TransFectin (Bio-Rad). After 48h post-transfection, cells were selected with puromycin (1 μg/ml) for 2–4 weeks and all cells were pooled, expanded and maintained under puromycin selection.

### Treatment with Shh, agonist and antagonist of Shh pathway

Cells were plated at a density of 70–80% confluency. At the following day, cells were serum deprived for 24h and subsequently treated with Shh (500 ng/ml, R&D Systems), SAG (1 μM, Enzo Life Sciences), or Cyc (10 μM, Sigma) for the indicated time points.

### Transient transfections

HEK293T cells were transiently transfected using PEI (polyethylenimine, Polysciences) as previously described [55]. Transfections of PLCs were performed with TransFectin (Bio-Rad) according to the manufacturer's protocol.

### Adenoviruses

Recombinant adenoviruses (Vector Biolabs) were replicated in HEK293T cells and purified using AdEasy virus purification kit (Stratagene) according to the manufacturer's protocol.

### RNA interference

Mouse Gprasp2 siRNAs were designed by using siDESIGN Center tool (http://www.thermoscientificbio.com/design-center/). Alexa Fluor 488 labeled control and Gprasp2 siRNA duplexes were generated by Eurogentec (Seraing, Belgium).

### RT-PCR and qPCR

Total RNA was obtained using Trizol and the RNeasy mini kit (Quiagen) according to the manufacturer's protocol. Subsequently, cDNA was prepared with the Super Script Synthesis System (Invitrogen) using 1–2 μg of total RNA. Real-time PCR was performed on the DNA Engine Opticon^®^ System (CFD-3200) using iQ SYBR Green supermix (BioRad). All primers (S2 Table) were designed by using AutoPrim software (http://www.autoprime.de/AutoPrimeWeb). The analysis was carried out in biological triplicates.

### FACS analysis

After infection with Adenovirus expressing Cre recombinase and GFP fluorophore, cells were trypsinized, centrifuged, and resuspended in FACS buffer (2% FCS, 0.5 mM EDTA, pH. 8.0 in PBS). GFP signals were detected with the FL1 channel (505–530 nm) of FACS machine (FACSAria, BD).

### GST pull-down assay

GST or GST-mPifo recombinant proteins expressed in E. coli strain BL21 were purified using Glutathione Sepharose 4B (GE Healthcare) according to the manufacturer's protocol. Equal amounts of beads coupled to GST or GST-mPifo proteins were incubated with *in vitro* translated HA-tagged hGPRASP2 (TNT Coupled Reticulocyte Lysate system, Promega) for overnight at 4°C on a rotating wheel (14rpm). After extensive washing with GST washing buffer (20 mM Tris-HCl, pH 7.4, 140 mM NaCl, 0.1% Triton X-100), the protein complex was analyzed by immunoblotting.

### Immunoprecipitation

The StrepII tag affinity purification was carried out as previously described [57]. To detect endogenous protein complex (Fig 2B), cells were lysed in IP lysis buffer (30 mM Tris-HCl, pH 7.5, 150 mM NaCl, 0.5% NP-40) containing protease inhibitor cocktail (Roche) and after centrifugation, the supernatant was subsequently incubated with the antibody (Smo, Abcam, ab38686; IgG, Santa Cruz, sc-2027) precoupled protein G sepharose (GE healthcare) for overnight at 4°C on a rotating wheel (14rpm). After extensive washing with IP lysis buffer, the protein complexes were analyzed by immunoblotting with indicated primary antibodies: Gprasp2 (1:1000, kindly provided by Dr. Erich Wanker, Max Planck Institute, Germany), Smo (1:1000, Abcam, ab38686), and β-arrestin 1/2 (1:1000, Cell Signaling, 4674). Note that Rabbit IgG TrueBlot^®^ (eBioscience, 18-8816-31) was used to minimize the detection of heavy and light chains from immunoprecipitated samples.

### Expression and purification of full-length hGPRASP2

The full length hGPRASP2 gene was cloned into pETM-11 giving a construct with a TEV protease cleavable N-terminal His_6_-tag. This construct was transformed into *E*. *coli* strain Rosetta2 (DE3) and cultured at 20°C in ZYM 5052 auto-induction medium [64]. Cells were lysed by sonication and the His-tagged protein purified by immobilized metal affinity chromatography (IMAC) using a 5-mL HiTrap Chelating HP column (GE Healthcare). The elution fractions were dialyzed overnight in the presence of TEV protease. The cleaved protein was further purified by IMAC and subjected to size exclusion chromatography using a HiLoad 16/600 Superdex 200 column (GE Healthcare). Fractions containing full length hGPRASP2 were pooled and concentrated to approx. 5 mg/mL and stored at 4°C. The protein concentration was determined by measuring the absorbance at 280 nm using the specific absorbance for full length hGprasp2 of 1.170 ml/mg.

### NMR Spectroscopy

NMR experiments were recorded on a 800 MHz spectrometer equipped with a TXI probehead at 298 K using 100 μM peptide (hSMO-WT or hSMO-CLD, PSL GmbH) in 50 mM Tris.HCl pH 8, 300 mM NaCl, 0.01% 1-thioglycerol (90% H_2_O / 10% D_2_O / 10% DMSO-d6). Interaction studies to hGPRASP2 were followed by recording 1D proton experiments before and after hGPRASP2 addition to a final concentration of 4 μM. 1D proton experiments were performed using a WATERGATE pulse sequence with 32k time domain points and 256 scans and recorded at 298 K on a 800 MHz spectrometer. Spectra were processed using TOPSPIN 3.2.

### Generation of Pifo antibodies

A rabbit polyclonal antibody against mouse Pifo has been generated as previously described [5] and used (Figs 3A, 4B and 6A and 6C, S2D and S4A Figs). A peptide comprising 14 amino acids RKHRSRVAYFSLYY from mouse Pifo protein was synthesized and coupled to BSA and OVA (PSL, Heidelberg, Germany). Rats were immunized subcutaneously and intraperitoneally with a mixture of 50 μg peptide-OVA, 5 nmol CPG oligonucleotide (Tib Molbiol, Berlin), 500 μl PBS and 500 μl incomplete Freund's adjuvant. A boost without adjuvant was given six weeks after the primary injection. Fusion was performed using standard procedures. Supernatants were tested in a differential ELISA with the Pifo peptide coupled to BSA and irrelevant peptides coupled to the same carrier. Monoclonal antibodies that reacted specifically with the Pifo peptide were further analyzed in immunostaining (S2E Fig). Tissue culture supernatant of Pifo 6E6 (rat IgG1) was used (Fig 6F).

### Immunoblotting

Immunoblotting analyses were performed by standard procedures with indicated antibodies: Gprasp2 (1:1000, kindly provided by Dr. Erich Wanker, Max Planck Institute, Germany), HA (1:1000, Sigma, H3663), GST (1:5000, Amersham Biosciences, 27-4577-50), His (1:1000, Abcam, ab18184), Flag (1:5000, Sigma, F1804), Smo (1:1000, Abcam, ab38686), KIF3b (1:1000, Santa Cruz, sc-50456), Dynein IC (1:1000, Sigma, D5167), α-tubulin (1:5000, Sigma, T6199), β-arrestin 1/2 (1:1000, Cell Signaling, 4674), Gli1 (1:1000, NEB, 2643), Gli2 (1:2000, kindly provided by Dr. Jonathan T. Eggenschwiler, Princeton University, USA), Pifo (1:250), GFP (1:1000, Biotrend, 600-101-215), and Actin (1:5000, BD, 612656). Protein bands were visualized on Hyperfilms (GE healthcare) using chemiluminescent detection (ECL, Millipore). NIH ImageJ software was used for quantification of the immunoblot analyses.

### Immunofluorescence and Imaging

For immunocytochemistry, cells were fixed with 4% PFA for 10 min (Figs 4C and 5E) or ice-cold methanol for 5 min and subsequently treated with 4% PFA for 5 min (Fig 6F and S2E Fig). After blocking (10% donkey serum, 1% BSA and 5% FCS, 0.5% Tween-20 in PBS) for 1 h, cells were incubated with the indicated concentration of primary antibodies for overnight at 4°C: Ace-tub (1:1000, Sigma, T7451), Smo (1:1000, Abcam, ab38686), GFP (1:500, Biotrend, 600-101-215), Gprasp2 (1:100, kindly provided by Dr. Erich Wanker, Max Planck Institute, Germany), Pericentrin (1:500, Covance, PRB-432C) and Pifo (1:10). The following secondary antibodies were applied for 2 h at room temperature: anti-rabbit IgG-555 (1:800, Invitrogen, A31572), anti-mouse IgG-647 (1:800, Dianova, 715-605-151), and anti-rat IgG-FITC (1:800, Southern Biotechnology, 3030–02). After DAPI staining (50 ng/ml), the samples were mounted with ProLong gold antifade reagent (Invitrogen) and images were acquired on a Leica laser-scanning SP5 confocal microscope with 63× objectives. Quantitative analysis of fluorescence intensities was carried out using NIH ImageJ or Imaris software.

### Mathematical modelling of Pifo in Shh signaling pathway

We employed a kinetic ODE model based on published knowledge about protein interactions to describe the *in vitro* data obtained from protein immunoblots. The ODEs we used were in the form $\frac{d\vec{x}\left(t\right)}{dt}=f\left(\vec{x}\left(t\right),shh\left(t\right),\vec{\Theta }\right)$, where $\vec{x}\left(t\right)$ is the state of the model at time t, *shh*(*t*) is the stimulation of the system by sonic hedgehog and $\vec{\Theta }$ is the set of kinetic parameters. Scaling constants that took differences in data scaling between immunoblots into account were fitted and the data then scaled to the interval [0,1]. Normally distributed noise was assumed for each time course. Parameters were estimated by maximum likelihood estimation for seven observed (Gli1, Gli2, Pifo, Venus-Pifo, Gli3, Gli3R, and Ptch), and five non-observed species (Smo, Shh-Ptch, Ptch, Gli1A, and Gli2A). A total of 613 data points across three conditions (*Pifo*^*wt/wt*^, *Pifo*^*FD/FD*^ and Rescue) were available (S1 Supporting Information) [65]. We assumed that the *Pifo*^*wt/wt*^ cells were in equilibrium prior to the first measurement, which allowed us to reduce the number of estimated parameters by establishing dependencies between parameters. The profile likelihood was calculated by applying the MATLAB implementation of the trust-region method (lsqnonlin). We used a lognormal error model to calculate the likelihood, including an individual noise parameter for each experimental measurement. For detailed description of the model and parameter fitting see S1 Supporting Information.

## Supporting Information

S1 Fig Generation of a Pifo conditional knock-out. (**A**) A targeting construct was generated by inserting a loxP site upstream of Exon3 and FRT-flanked PGK-neo-pA cassette followed by a second loxP site downstream of Exon6 of Pifo gene. (**B**) Southern blot of ES cells digested with BamHI and hybridized with external 3’ probe indicating wild-type allele (9782bp) and targeted allele (9145bp). Deletion of the PGK-neo-pA cassette (**C**) and genotypes of generated PLCs (**D**) were confirmed by allele-specific PCR. Yellow arrows indicate the locations of genotyping primers.
(TIF)

S2 Fig Cre recombinase mediated Pifo depletion in PLCs. (**A**) Representative result of PCR genotyping from genomic DNA of *Pifo*^*wt/wt*^ and *Pifo*^*flox/flox*^ PLCs (1, 2 lines) and PCR analysis of Cre-mediated recombination events (3, 4, 5, 6 lines) after infection of *Pifo*^*flox/flox*^ with AdCre recombinase. Transduction efficiency of AdCre was observed by GFP expression (**B**) and quantified by FACS analysis (**C**) for GFP positive cells. (**D**) Determination of endogenous Pifo protein levels from *Pifo*^*flox/flox*^ PLCs infected with or without AdCre for the indicated time points. Protein loading was controlled by Actin levels. Scale bar = 25 μm (**E**) Representative confocal images for ciliary Pifo in *Pifo*^*wt/wt*^, *Pifo*^*FD/FD*^ PLCs after Shh stimulation. Scale bar = 2 μm.
(TIF)

S3 Fig Pifo is required for Shh target gene activation and depletion of Pifo or Gprasp2 does not affect cilia assembly. (**A**-**B**) The mRNA levels of Ptch1, Gli1, Gli2, Gli3 and Smo in *Pifo*^*wt/wt*^, *Pifo*^*wt/FD*^, and *Pifo*^*FD/FD*^ PLCs treated with or without Shh. Graphs represent quantification of RT-PCR data that show the mean fold change of mRNA expression normalized to α-Actin levels. Mean ± SD of three independent experiments. (**C**) Representative confocal image of cilia and basal bodies. The quantification of ciliation (**D**) and cilia abnormality (**E**). Scale bar = 2 μm. >100 cilia per condition were analyzed. All error bars indicate the mean ± SD of three independent experiments. Data were analyzed using a two tailed unpaired *t*-test (* = p<0.1, ** = p<0.01, *** = p<0.001).
(TIF)

S4 Fig Gprasp2 is required for localization of ciliary Pifo. Selected still images (**A**) and quantification (**B**) of ciliary Pifo after 48h of siRNA-mediated knock-down of Gprasp2 in PLCs. Scale bar = 2 μm. >100 cilia per condition were analyzed. All error bars indicate the mean ± SD of three independent experiments. Data were analyzed using a two tailed unpaired *t*-test (**** = p<0.0001).
(TIF)

S5 Fig Gprasp2 is necessary for rapid ciliary localization of Smo in Shh signaling. Selected still images (**A**) and quantification (**B**) of confocal time-lapse movies of Gpraps2-depleted *Pifo*^*flox/flox*^ cells stably expressing Venus-tagged Arl13b and RFP-tagged Smo. Scale bar = 5 μm. >100 cilia per condition were analyzed. All error bars indicate the mean ± SD of three independent experiments. Data were analyzed using a two tailed unpaired *t*-test (*** = p<0.001).
(TIF)

S6 Fig Multiple species ClustalW protein alignment of Gprasp2. Gprasp2, particularly the C-terminus is highly conserved among the species (* indicates conserved protein residues).
(DOCX)

S1 Supporting Information Supplementary Modeling Supporting Information. (PDF)

S1 Table Pifo interactome analysis. For each protein group, the protein descriptions, the gene names, the uniprot identifiers as well as the sequence coverage and unique peptide counts are shown. Additionally, the mean ratio from two independent experiments for the affinity purified PIFO compared to the SF-TAP control is shown as well as the significance B for enrichment. Proteins with a significance level below 0.05 were considered to be potential PIFO interactors. Identification, quantification and significance determination was performed by using MaxQuant, including the Perseus software for statistical analysis. Protein groups significantly enriched are colored in green, those of special interest are colored in red.
(PDF)

S2 Table Primers used for targeting vector construction, PCR genotyping, RT-PCR and qPCR. The restriction enzyme sites are indicated in red.
(DOC)
