# Supplementary figures and images for: Pitchfork and Gprasp2 Target Smoothened to the Primary Cilium for Hedgehog Pathway Activation
Source: PLoS One. 2016 Feb 22;11(2):e0149477. doi: 10.1371/journal.pone.0149477 (PMC4763541; doi:10.1371/journal.pone.0149477)

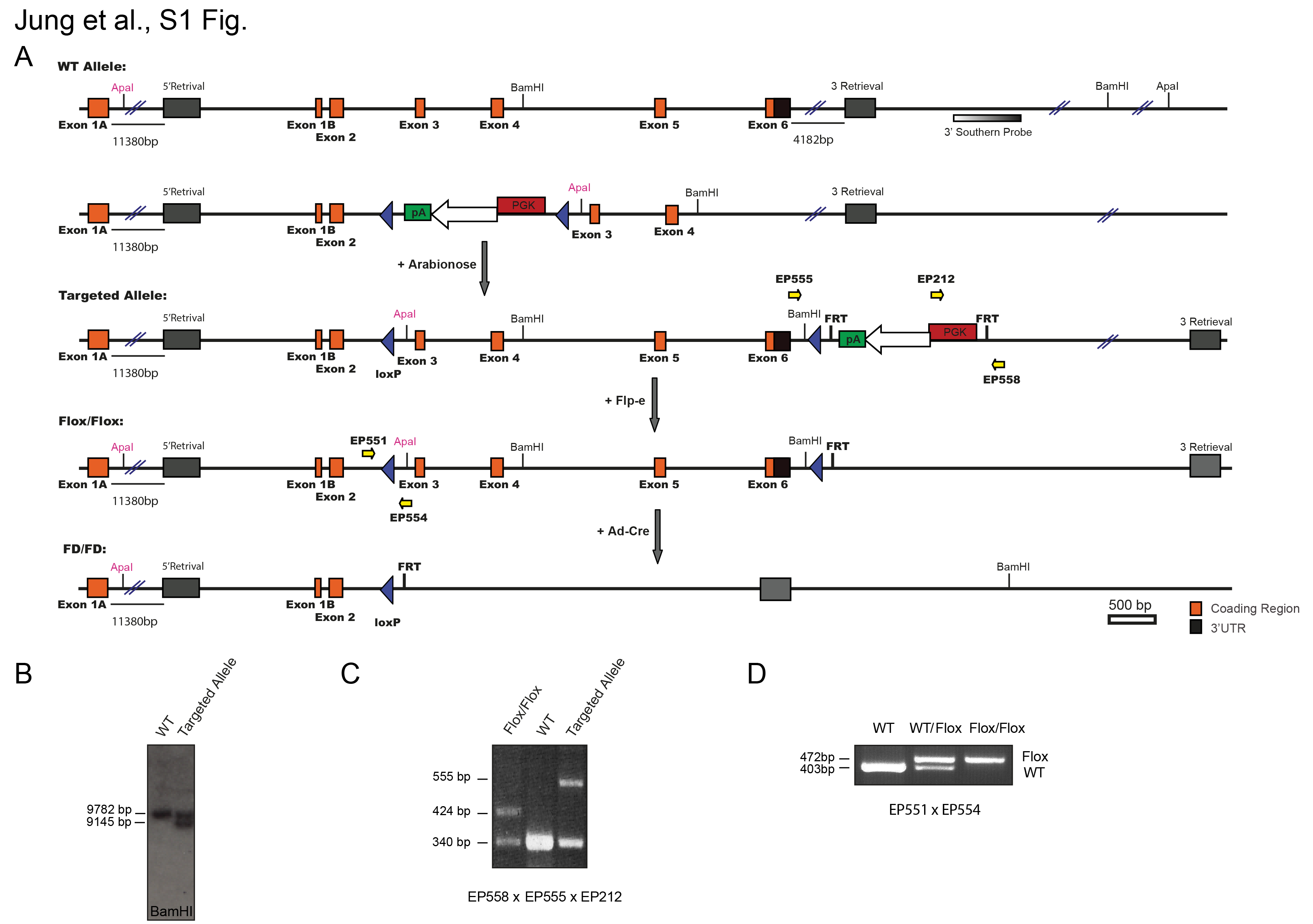

Supplement: S1 Fig — (A) A targeting construct was generated by inserting a loxP site upstream of Exon3 and FRT-flanked PGK-neo-pA cassette followed by a second loxP site downstream of Exon6 of Pifo gene. (B) Southern blot of ES cells digested with BamHI and hybridized with external 3’ probe indicating wild-type allele (9782bp) and targeted allele (9145bp). Deletion of the PGK-neo-pA cassette (C) and genotypes of generated PLCs (D) were confirmed by allele-specific PCR. Yellow arrows indicate the locations of genotyping primers. (TIF) [file pone.0149477.s001.tif]

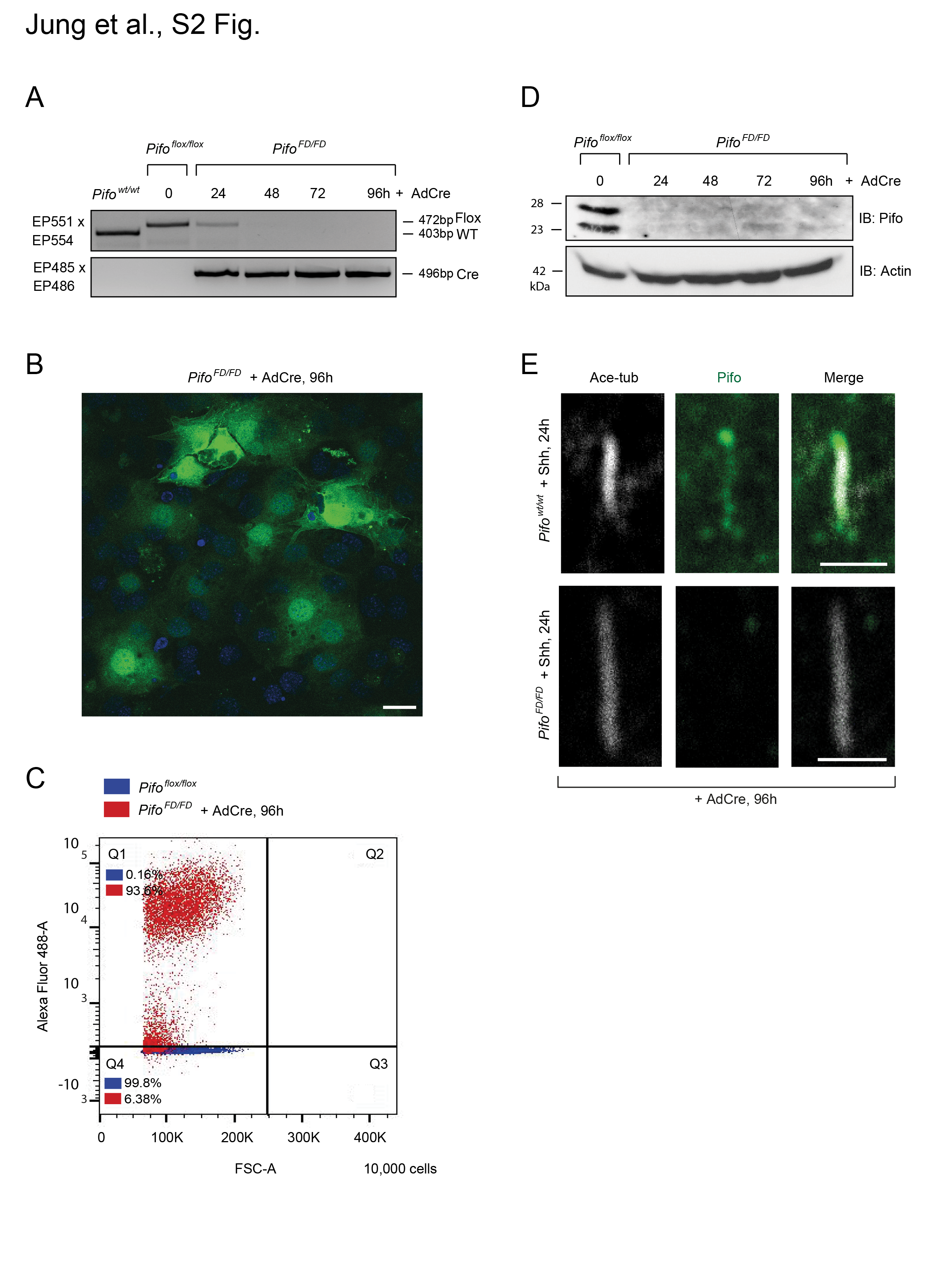

Supplement: S2 Fig — (A) Representative result of PCR genotyping from genomic DNA of Pifowt/wt and Pifoflox/flox PLCs (1, 2 lines) and PCR analysis of Cre-mediated recombination events (3, 4, 5, 6 lines) after infection of Pifoflox/flox with AdCre recombinase. Transduction efficiency of AdCre was observed by GFP expression (B) and quantified by FACS analysis (C) for GFP positive cells. (D) Determination of endogenous Pifo protein levels from Pifoflox/flox PLCs infected with or without AdCre for the indicated time points. Protein loading was controlled by Actin levels. Scale bar = 25 μm (E) Representative confocal images for ciliary Pifo in Pifowt/wt, PifoFD/FD PLCs after Shh stimulation. Scale bar = 2 μm. (TIF) [file pone.0149477.s002.tif]

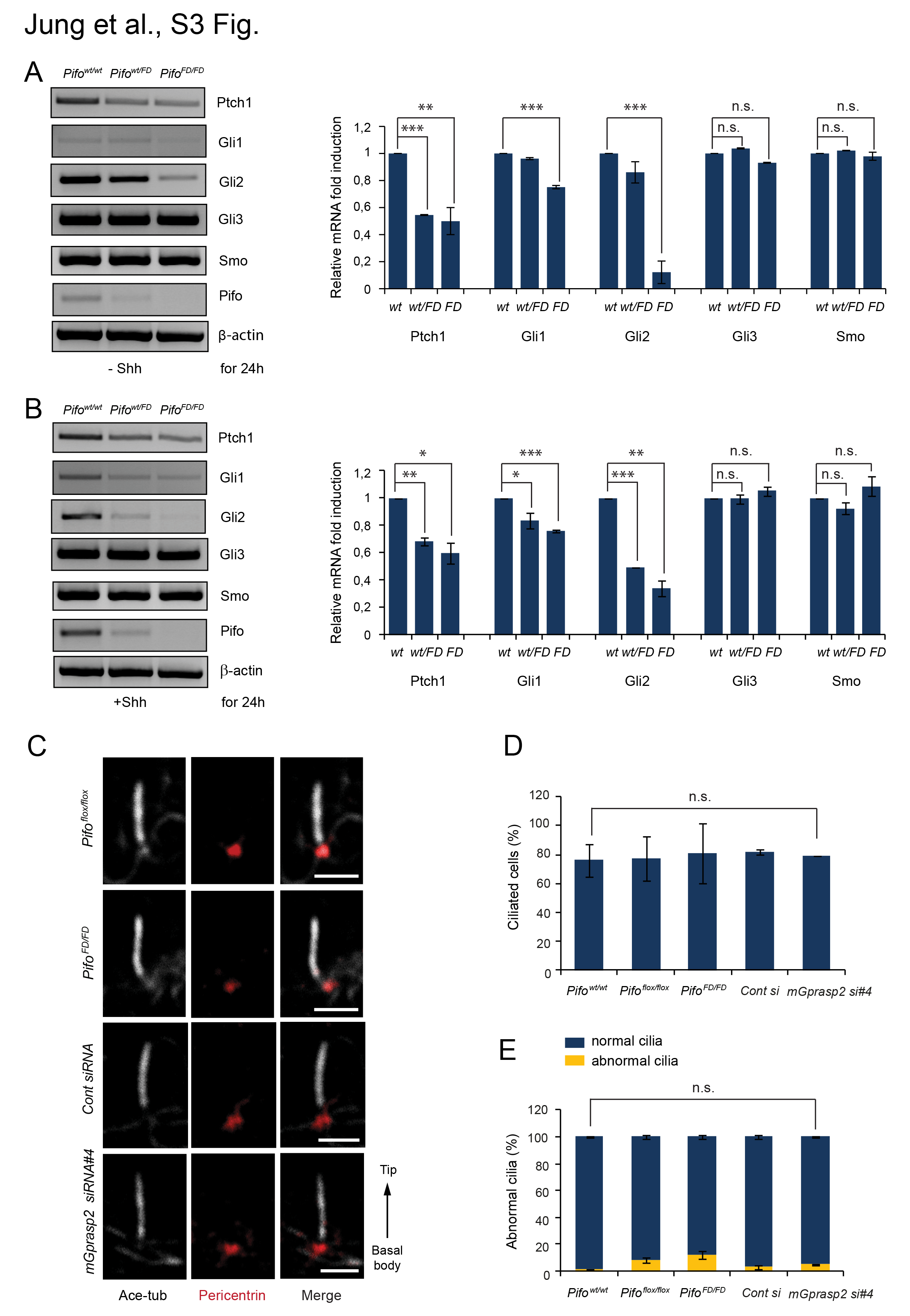

Supplement: S3 Fig — (A-B) The mRNA levels of Ptch1, Gli1, Gli2, Gli3 and Smo in Pifowt/wt, Pifowt/FD, and PifoFD/FD PLCs treated with or without Shh. Graphs represent quantification of RT-PCR data that show the mean fold change of mRNA expression normalized to α-Actin levels. Mean ± SD of three independent experiments. (C) Representative confocal image of cilia and basal bodies. The quantification of ciliation (D) and cilia abnormality (E). Scale bar = 2 μm. >100 cilia per condition were analyzed. All error bars indicate the mean ± SD of three independent experiments. Data were analyzed using a two tailed unpaired t-test (* = p<0.1, ** = p<0.01, *** = p<0.001). (TIF) [file pone.0149477.s003.tif]

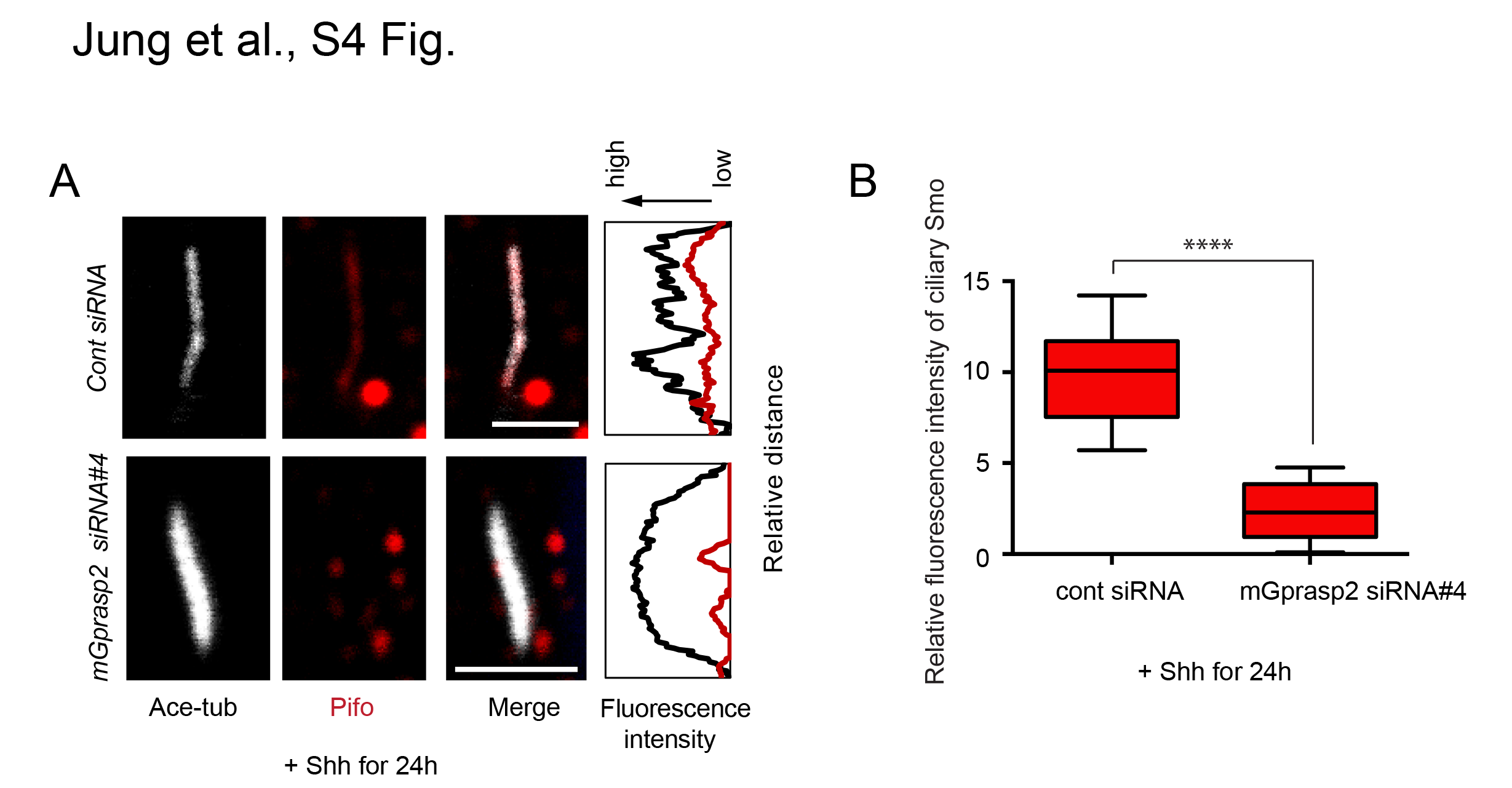

Supplement: S4 Fig — Selected still images (A) and quantification (B) of ciliary Pifo after 48h of siRNA-mediated knock-down of Gprasp2 in PLCs. Scale bar = 2 μm. >100 cilia per condition were analyzed. All error bars indicate the mean ± SD of three independent experiments. Data were analyzed using a two tailed unpaired t-test (**** = p<0.0001). (TIF) [file pone.0149477.s004.tif]

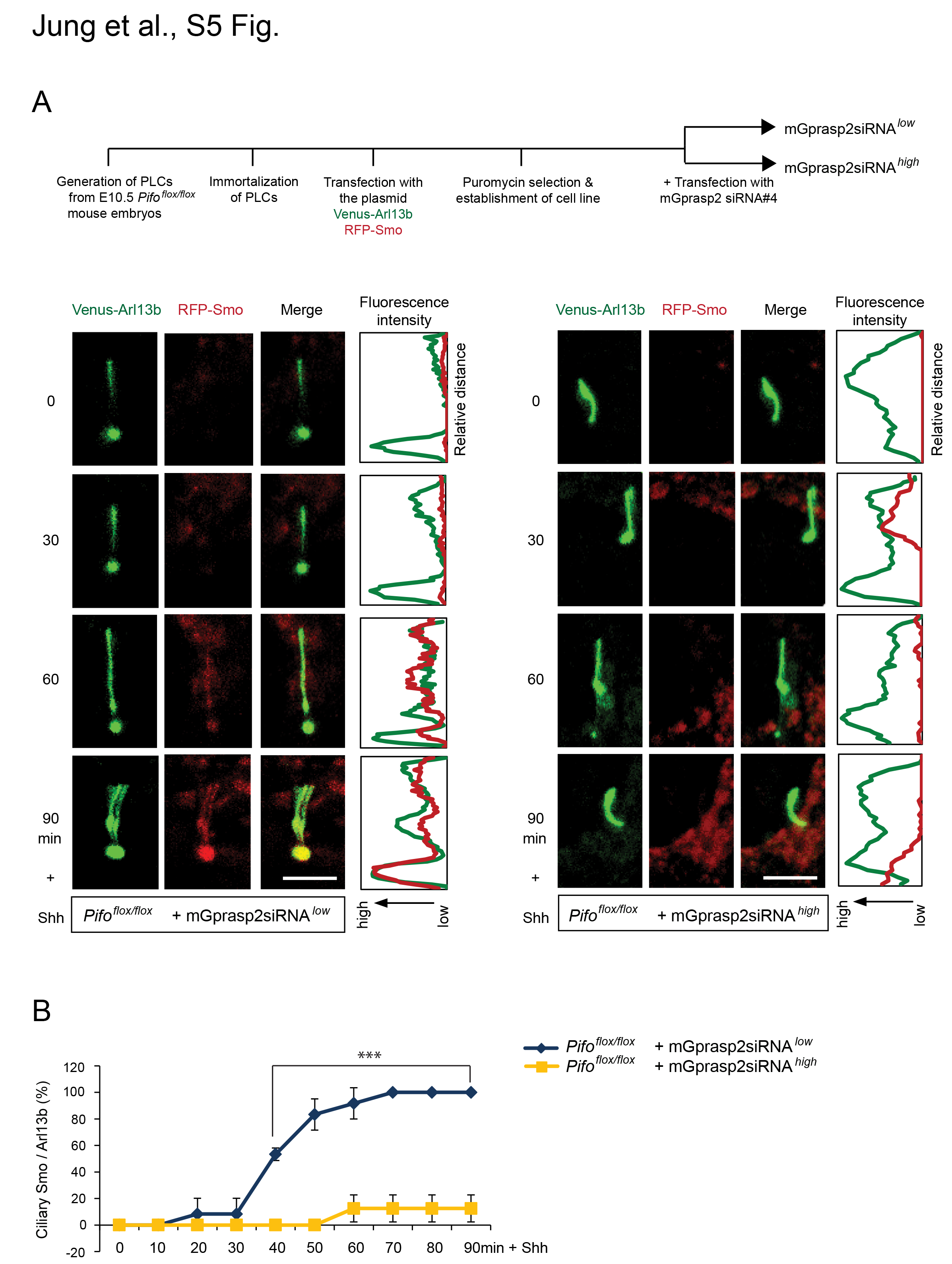

Supplement: S5 Fig — Selected still images (A) and quantification (B) of confocal time-lapse movies of Gpraps2-depleted Pifoflox/flox cells stably expressing Venus-tagged Arl13b and RFP-tagged Smo. Scale bar = 5 μm. >100 cilia per condition were analyzed. All error bars indicate the mean ± SD of three independent experiments. Data were analyzed using a two tailed unpaired t-test (*** = p<0.001). (TIF) [file pone.0149477.s005.tif]
