# Supplementary material for: Pitchfork and Gprasp2 Target Smoothened to the Primary Cilium for Hedgehog Pathway Activation
Source: PLoS One. 2016 Feb 22;11(2):e0149477. doi: 10.1371/journal.pone.0149477 (PMC4763541; doi:10.1371/journal.pone.0149477)
Supplement: S6 Fig — Gprasp2, particularly the C-terminus is highly conserved among the species (* indicates conserved protein residues). (DOCX) [file pone.0149477.s006.docx]

**Jung et al., S6 Fig.**

CLUSTAL W (1.81) multiple sequence alignment of Gprasp2

Equus MSPAVVRPKVRTQAQVMPG----------------ARPKTESKVMSGARPKT--ESQAVA 42

Sus MTGAEIEPGAQAKPQKKPGEEVVGRTERENEVPMVVRPKVRTQATPGARPKN--ESKGMA 58

Homo MTGAEIEPSAQAKPEKKAGEEV------------IAGPERENDVPLVVRPKV--RTQATT 46

Macaca MTGAEIEPSAQAKPEKKAGEEV------------IAGPERENDVPLVVRPKV--RTQATT 46

Pan MTGAEIEPSAQAKPEKKAGEEV------------IAGPERENDVPLVVRPKV--RTQATT 46

Pongo MTGAEIEPSAQAKPEKKAGEEV------------VAGPERENDVPLVVRPKV--RTQATT 46

Nomascus MTGAEIEPSAQAKPEKKAGEEV------------IAGAERENDVPLVVRPKV--RTQATT 46

Callithrix MTGAEVAPSAQAKPEKKAEEEV------------MPGAERENDVPLVVRPKV--RTQA-- 44

Mus MTGAEVETGSQAKPDKKPQEEVAG------------GAERESEAPLVVRPKVRPQAPATS 48

Rattus MTGAEVETGSQAKSDKKPQEEVVVD-----------GVERESEVPLVVRPKVRPQAPATS 49

*: * : . :::.: . : .... .*** .: .

Equus GARPKTDCQAMAGARPKTESQTVAGARPKTDCQAMAGARPRTESQAMAGARPKTEARAVG 102

Sus GARSKSESKNMSGARPKTESQAMSGARPKTESQAMAGARPKSESQAVAGARPKTEARAVG 118

Homo GARPKTETKSVPAARPKTEAQAMSGARPKTEVQVMGGARPKTEAQGITGARPKTDARAVG 106

Macaca GARPKTETKSVPAARPKTEAQAMSGARPKTEVQVMGGARPKTEAQGITGARPKTDARAVG 106

Pan GARPKTETKSVPAARPKTEAQAMSGARPKTEVQVMGGARPKTEAQGIAGARPKTDARAVG 106

Pongo GARPKTETKSVPAARPKTEAQAMSGARPKTEAQVMGGARPKTEAQGMAGARPKTDARAVG 106

Nomascus GARPKTETKSVPVARPKTEAQAMG------------GARPKTEAQGIAGARPKTDARAVG 94

Callithrix --------------------------------QVMGGARPKNEAQGKAGARPKTNARAVG 72

Mus GARPKTETKSSSRARPKTETQSVSGTR-----HRMSGARPRSEAQLMSGARPKTDARAVG 103

Rattus GARPKTETKSSSRARPKTESQSVSGRRPKTEAQSMTGARPRLEAQVMSGARPKTDARAVG 109

****: *:* :******:*****

Equus GARPKTEAKAVPGSRPKDEAQAWAQTEFVAEAMSQAEGMSQTHAVAWPLVGTESGSVVKP 162

Sus GARPKTEAKAIPGARSKDEAQAWAQTEFGAEAMSQAEGVSQTNVVAWPLVNNESGSAAKP 178

Homo GARSKTDAKAIPGARPKDEAQAWAQSEFGTEAVSQAEGVSQTNAVAWPLATAESGSVTKS 166

Macaca GARSKTDAKAIPGARPKDEAQAWAQSEFGTEAVSQAEGVSQTNAVAWPLATAESGSVTKS 166

Pan GARSKTDAKAIPGARPKDEAQAWAQSEFGTEAVSQAEGVSQTNAVAWPLATAESGSVTKS 166

Pongo GARSKTDAKAIPGARPEDEAQAWAQSEFGTEAVSQAEGVSQTNAVAWPLATAESGSVTKS 166

Nomascus GARSKTDAKAIPGARPKDEAQVWAQSEFGTEAVSQAEGVSQTNAIAWPLATAESGSVTKS 154

Callithrix GARPKTDAKAIPVARTKNEAQAWDQTEFGTEAVSQAEGVSQTNAVAWPPATPESGSVTKS 132

Mus GARPKTEAKPIPGARPKGDAQAWAHSEFGAEAMPRAERAHLSNSVTWPPVNVGSATVTKS 163

Rattus GARPKTEAKPIPGARPKNDSRAWAQSEFGAEAMSHAERAPLPNAVTWPLVNSESTSVTKS 169

***.**:**.:* :*.:.:::.* ::** :**:.:** .: ::** . * :..*.

Equus MALSVDRELVNVDSETFPGSQVQTGIQPWFASGEETNMGSWCYPRPRAREEASNESGFWS 222

Sus MALSVDRELANVDTETFPGSQVQTGIQPWFGAGEETNMGSWCYPRPRAREEASNESGFWS 238

Homo KGLSMDRELVNVDAETFPGTQGQKGIQPWFGPGEETNMGSWCYSRPRAREEASNESGFWS 226

Macaca KGLSMDRELVNVDAETFPGTQGQKGIQPWFGPGEETNMGSWCYSRPRAREEASNESGFWS 226

Pan KGLSMDRELVNVDAETFPGTQGQKGIQPWFGPGEETNMGSWCYSRPRAREEASNESGFWS 226

Pongo KGLSMDRELVNVDAETFPGTQGQKGIQPWFGPGEETNMGSWCYSRPRAREEASNESGFWS 226

Nomascus KGLSMDRELVNVDAETFPGTQGQKGIQPWFGPGEETNMGSWCYSRPRAREEASNESGFWS 214

Callithrix KGLSMDRELVNVDAETFPSAQDQKGIQPWFGPEEETNMGSWCYSRPRAREKASNESGFWS 192

Mus KSLSMNTELASMGSEIFSGTQGQPGIEPWFGPREEANMGSWCYPRPRAREETSNES---- 219

Rattus KSLSMDREMANMGSETFPSTQGQSGIEPWFGPREEANMGSWCYPRPRAREETSNESGFWS 229

.**:: *:..:.:* *..:* * **:***.. **:*******.******::****

Equus ADETSTMSSFWAGEEASIRSWPREEANTRSRHRAKHQPNPRSRPRSKQDPYIDSWSGSEE 282

Sus ADETSTMSSFWAGEEASIRSWPREEANTRSRHRAKHQPNPRSRPRSKQDPYIDSWSGSEE 298

Homo ADETSTASSFWTGEETSVRSWPREESNTRSRHRAKHQTNPRSRPRSKQEAYVDSWSGSED 286

Macaca ADETSTASSFWTGEETSVRSWPREESNTRSRHRAKHQTNPRSRPRSKQEAYVDSWSGSED 286

Pan ADETSTASSFWAGEETSVRSWPREESNTRSRHRAKHQTNPRSRPRSKQEAYVDSWSGSED 286

Pongo ADETSTASSFWAGEETSVRSWPREESNTRSRHRAKHQTNPRSRPRSKQEAYVDSWSGSED 286

Nomascus ADETSTASSFWAGEETSVRSWPREESNTRSRHRAKHQTNPRSRPRSKQEACVDSWSGSED 274

Callithrix ADETSTMSSFWAGEETSVRSWPREESNTRSRHRAKHQTNPSSGPRPQQEAYVDSWSGSED 252

Mus ADENSTMSSFWTREETSIRSWPREEVNTRSRHRAKHQTNARSKPRSKQDPYIDSLSGSED 279

Rattus ADETSTMSSLWTGQETNIRSWPREEVNTRSRHRAKHQANTRSKPRSKQDAYIDSLSGSED 289

***.** **:*: :*:.:******* ***********.*. * **.:*:. :** ****:

Equus ESGNPFCLWAGENTSNLFRPRVRDEANIRSKLRTKREDFFESESEDEYYKESWFLPGEEA 342

Sus ESANPFCLWAGENTNNLFRPRVRDEANMRSKLRTKREDFFESESEDEYYKESWFLPGEEA 358

Homo EASNPFSFWVGENTNNLFRPRVREEANIRSKLRTNREDCFESESEDEFYKQSWVLPGEEA 346

Macaca EASNPFSFWVGENTNNLFRPRVREEANIRSKLRTNREDCFESESEDEFYKQSWVLPGEEA 346

Pan EAGNPFSFWAGENTNNLFRPRVREEANIRSKLRTNREDCFESESEDEFYKQSWVLPGEEA 346

Pongo EAGNPFSFWAGENTNNLFRPRVREEANIRSKLRTNREDCFESESEDEFYKQSWVLPGEEA 346

Nomascus EAGNPFSFWAGENTNNLFRPRVREEANIRSKLRTNREDCFESESEDEFYKQSWVLPGEEA 334

Callithrix EAGNPFS-WAGENTNNLFGPRVKEEANIRSKPRTKREDCFESESEDEFYKQSWVLPGEEA 311

Mus EASNPFCFWAGENTNDMFRARGRDEANARPKIRTKREDYFEDE--DEIYKESWLLPGEEG 337

Rattus EAGNPFCFWAGENANDMFRPRGRDEANVRPKIRTKRENYFEDE--EEFCKESWLLPGEEA 347

*:.***. *.***:.::* .* ::*** *.* **:**: **.* :* *:**.*****.

Equus SSRFRPRDKEEPHTVLKPRVQKDVNNSDKVKQEPRFEEEVIIGSWFWAEKEAGMEAGASA 402

Sus NSRFRPRDKEEPNTILKPRAQKDANNGDRVKQEPRFEEEVIIGSWFWAEKEAGMEAGASA 418

Homo NSRFRHRDKEDPNTALKLRAQKDVD-SDRVKQEPRFEEEVIIGSWFWAEKEASLEGGASA 405

Macaca NSRFRHRDKEDPNTALKLRAQKDVD-SDRVKQEPRFEEEVIIGSWFWAEKEASLEGGASA 405

Pan NSRFRYRDKEDPNTALKLRAQKDVD-SDRVKQEPRFEEEVIIGSWFWAEKEASLEGGASA 405

Pongo DSRFRHRDKEDPNTALKLRAQKDVD-SDRVKQEPRFEEEVIIGSWFWAEKETSLEGGASA 405

Nomascus NSRFRCRDKEDPNTSLKPRAQKDVD-SDRVKQEPRFEEEVIIGSWFWAEKEASLEGGASA 393

Callithrix NSRFKRRDKEDPNTNLKSRAQKDVN-GDRVKQEPRFEEEVIIGSWFWAENEASLEGGASA 370

Mus N-RFRRRDKEEPNKTLKNENEKDVKNDETVEQESRLEEEVIIGSWFWAEQETNVEAGASA 396

Rattus N-RFRGRDKEEPNKTLKNSDQKDVKNDEKVKQESKIEEEVIIGSWFWAEQETSLEAAASA 406

. **: ****:*:. ** :**.. .: *:**.::*************:*:.:*..***

Equus ICESRPGAEEGAIGGSLFWTEEKSSLGAVAREEARPESEEEAIFGSWFWDRDEACFDLNP 462

Sus ICESEPGAEEGAIGGSLFWTEEKSSLGAVAREETKPESEEEAIFGSWFWDRDEACFDLNP 478

Homo ICESEPGTEEGAIGGSAYWAEEKSSLGAVAREEAKPESEEEAIFGSWFWDRDEACFDLNP 465

Macaca ICESEPGTEEGAIGGSAYWAEEKSSLGAVAREEAKPESEEEAIFGSWFWDRDEACFDLNP 465

Pan ICESEPGTEEGAIGGSAYWAEEKSSLGAVAREEAKPESEEEAIFGSWFWDRDEACFDLNP 465

Pongo ICESEPGTEEGAIGGSAYWAEEKSSLGAVAREEAKPESEEEAIFGSWFWDRDEACFDLNP 465

Nomascus ICESEPGTEEGAIGGSAYWAEEKSSLGAVAREEAKPESEEEAIFGSWFWDRDEACFDLNP 453

Callithrix ICESEPGTEEGAVGGSLYWAEEQSSLGAVARQEAKPESEEDAIFGSWFWDRDEACFDLNP 430

Mus ICDAEPGAEEGAIGGSLFWTEEKPSLGAVARDEVRPESEEEALFGSWFWDRDEACFDPNP 456

Rattus ICESEPGAEEGAIGGSLFWTEEKPDLGAVARDEVRPESEEEAIFGSWFWDRDEACFDPNP 466

**::.**:****:*** :*:**:..******:*.:*****:*:************** **

Equus SPVYRANSRFRDSVEEEVNVSSRPQTWEEVTVEFKPGPCHVVGFPSPSSFRISDEAASVF 522

Sus RPVYKASPRFRNPAEEEVNVSSRPQTWEEVTVEFKPGPCHGVGFPSPSPFRIPEEAASVY 538

Homo CPVYKVSDRFRD-AAEELNASSRPQTWDEVTVEFKPGLFHGVGFRSTSPFGIPEEAS--- 521

Macaca CPVYKVSDRFRD-AAEELNASSRPQTWDEVTVEFKPGLFHGVGFRSTSPFGIPEEAS--- 521

Pan CPVYKVSDRFRD-AAEELNASSRPQTWDEVTVEFKPGLFHGVGFRSTSPFRIPEEAS--- 521

Pongo CPVYKVSDRFRD-AAEELNASSRPQTWDEVTVEFKPGLFHGVGFRSTSPFRIPEEAS--- 521

Nomascus CPVYKVTDRFRD-AAEELNASSRPQTWDEVTVEFKPGLFHGVGFRSTSPFRIPQEAS--- 509

Callithrix CPVYKVSDRFRDTAEEELNASSRPQTWDEVTVEFKPGLFHGVGFPSTSPFRFTEDAS--- 487

Mus TPVYTAKSRYRD-PEEDLNLASRPKTWDEVTIEFKP-PCHGLGFPSPRPFIIPEGAS--- 511

Rattus TPVYTAKSRYRD-PEEDLNLASRPKSWDEVTIEFTP-PCHGVGFPFPRPFIIPEGAS--- 521

*** .. *:*: *::* :***::*:***:**.* * :** . .* :.: *:

Equus SEMFEGKPKNVELTPEGEEQESLLQSDQPESEFPFQYDPSYRSVREIREHLRARESAEPE 582

Sus SEMFEGKPKGVEVTPEGEEQESLLQSDQPDSEFTFQYDPSYRSVREIREHLRTRESADPE 598

Homo -EMLEAKPKNLELSPEGEEQESLLQPDQPSPEFTFQYDPSYRSVREIREHLRARESAESE 580

Macaca -EMLEAKPKNLELSPEGEEQESLLQPDQPSPEFTFQYDPSYRSVREIREHLRARESAESE 580

Pan -EMLEAKPKNLELSPEGEEQESLLQPDQPSPEFTFQYDPSYRSVREIREHLRARESAESE 580

Pongo -EMLEAKPKNLELSPEGEEQESLLQPDQPSPEFTFQYDPSYRSVREIREHLRARESAESE 580

Nomascus -EMLEAKPKNLELSPEGEEQESLLQPDQPSPEFTFQYDPSYRSVQEIREHLRARESAESE 568

Callithrix -EMFEAKPKNLELSPEGEEQESLLQPDQPSPEFTFQYDPSYRSVREIREHLKTRESAESE 546

Mus -GNSEEKAKNAELGAEGEEQDSVAQRDLPEPEFPFQYDPSYRSVQEIREHLKARESAQPE 570

Rattus -GNTEEKAKSAELGAEGEEQESVVQRDPPEPDFPFQYDPSYRSVREIREHLKARESAQPE 580

* *.*. *: .*****:*: * * *..:*.**********:******::****:.*

Equus TWSCSCIQCELKIGTAEFEELLLLMDKIRDPFIHEISKIAMGMRSASQFTRDFIRDSGVV 642

Sus NWSCSCIQCELKIGPEEFEELLLLMDKIRDPFIHEISKIAMGMRSASQFTRDFIRDSGVV 658

Homo SWSCSCIQCELKIGSEEFEEFLLLMDKIRDPFIHEISKIAMGMRSASQFTRDFIRDSGVV 640

Macaca SWSCSCIQCELKIGSEEFEEFLLLMDKIRDPFIHEISKIAMGMRSASQFTRDFIRDSGVV 640

Pan SWSCSCIQCELKIGSEEFEELLLLMDKIRDPFIHEISKIAMGMRSASQFTRDFIRDSGVV 640

Pongo SWSCSCIQCELKIGSEEFEELLLLMDKIRDPFIHEISKIAMGMRSASQFTRDFIRDSGVV 640

Nomascus SWSCSCIQCELKIGSEEFEELLLLMDKIRDPFIHEISKIAMGMRSASQFTRDFIRDSGVV 628

Callithrix SWSCSCIQCELKMVLEEFEELLILMDKIRDPFIQEISKIAMGMRSASQFTRDFIRDSGVV 606

Mus NWSCTCIQCELRISSAEFEELLLLMDRIRDPFIHEIAKIAMGMRTASQFTRDFIRDSGVV 630

Rattus NWSCNCIQCELRIGSAEFEELLLLMDRIRDPFIHEISKIAMGMRGASQFTRDFIRNSGVV 640

.***.******:: ****:*:***:******:**:******* **********:****

Equus SLIETLLNYPSSRVRTSFLENMIHMAPPYPNLNMIETFVCQVCEETLAHSVGSPEQLLGL 702

Sus SLIETLLNYPSSRVRTTFLENMIHMAPPYPNLNMIETFICQVCEETLAHSVGSPEQLLGL 718

Homo SLIETLLNYPSSRVRTSFLENMIHMAPPYPNLNMIETFICQVCEETLAHSVDSLEQLTGI 700

Macaca SLIETLLNYPSSRVRTSFLENMIHMAPPYPNLNMIETFICQVCEETLAHSVDSLEQLTGI 700

Pan SLIETLLNYPSSRVRTSFLENMIHMAPPYPNLNMIETFICQVCEETLAHSVDSLEQLTGI 700

Pongo SLIETLLNYPSSRVRTSFLENMIHMAPPYPNLNMIETFICQVCEETLAHSVDSLEQLTGI 700

Nomascus SLIETLLNYPSSRVRTSFLENMIHMAPPYPNLNMIETFICQVCEETLAHSVDSLEQLTGI 688

Callithrix SLIETLLNYPSSRARTSFLENMIHMAPPYPNLNMIETFICQVCEETLAHSVDSLEQLTGI 666

Mus SLIEALMNYPSSRVRTNFLENMVHMAPPYPNLNMIETFICQVCEETLSHSVNSPEQLTGM 690

Rattus SLIEALMNYPSSRARTAFLENMIEMAPPYPDLNMIETFICQVCEETLSHSVNSPEQLTGM 700

****:*:******.** *****:.******:*******:********:***.* *** *:

Equus KMLRHLTSTTDYHTLVANYMSGFLSLLTTGNARTKFHVLKMLLNLSGNAMVAKKLFSAKA 762

Sus RMLRHLTTTTDYHTLVANYMSGFLSLLTTGNARTKFHVLKMLLNLSENAMVAKKLFSAKA 778

Homo RMLRHLTMTIDYHTLIANYMSGFLSLLTTANARTKFHVLKMLLNLSENPAVAKKLFSAKA 760

Macaca RMLRHLTMTIDYHTLIANYMSGFLSLLTTANARTKFHVLKMLLNLSENPAVAKKLFSAKA 760

Pan RMLRHLTMTIDYHTLIANYMSGFLSLLTTANARTKFHVLKMLLNLSENPAVAKKLFSAKA 760

Pongo RMLRHLTMTIDYHTLIANYMSGFLSLLTTANARTKFHVLKMLLNLSENPAVAKKLFSAKA 760

Nomascus RMLRHLTMTIDYHTLIANYMSGFLSLLTTANARTKFHVLKMLLNLSENPAVAKKLFSAKA 748

Callithrix RMLRHLTMTIDYHTLIANYMSGFLSLLTTANATTKFHVLKMLLNLSENPAVAKHLFSAKA 726

Mus RMLRHLTITTDYHVLIANYVSGFLALLTTGDARTKFHVLKMLLNLSDNPMVAKKLFSAKA 750

Rattus RMLRHLTITTDYHTLIANYISGFLALLTTGDARTKFHVLKMLLNLSDNPMVAKKLFSAKA 760

:****** * ***.*:***:****:****.:* ************* *. ***:******

Equus LSIFVALFNVEETNDNIQIVIKMFQNISNIIKNGTMALIDDDFSLEPLISAFHEFEKLAK 822

Sus LSIFVGLFNIEETNDNIQIVIKMFQNISNIIKSGTMSLIDDDFSLEPLISAFHEFEKLAE 838

Homo LSIFVGLFNIEETNDNIQIVIKMFQNISNIIKSGKMSLIDDDFSLEPLISAFREFEELAK 820

Macaca LSIFVGLFNIEETNDNIQIVIKMFQNISNIIKSGKMSLIDDDFSLEPLISAFREFEELAK 820

Pan LSIFVGLFNIEETNDNIQIVIKMFQNISNIIKSGKMSLIDDDFSLEPLISAFREFEELAK 820

Pongo LSIFVGLFNIEETNDNIQIVIKMFQNISNIIKSGKMSLIDDDFSLEPLISAFREFEELAK 820

Nomascus LSIFVGLFNIEETNDNIQIVIKMFQNISNIIKSGKMSLIDDDFSLEPLISAFREFEELAK 808

Callithrix LSIFVGLFNIEETNDNIQIVIKMFQNISNIIKSGKMSLIDDDFSLEPLIAAFREFEELAK 786

Mus LSIFVGLFNIEETNDNIQIVIKMFQNISNIVKSGAMSLLDDDFSLEPLVSAFHEFEELAK 810

Rattus LSIFVGLFNIEETNDNIQIVIKMFQNISNIVKSGAMSLIDDDFSLEPLVSAFHEFEELAK 820

*****.***:********************:*.* *:*:*********::**:***:**:

Equus ELQVQIDNQKDPEVGQQS 840

Sus ELQVQIDNQKDPEVGQQS 856

Homo QLQAQIDNQNDPEVGQQS 838

Macaca QLQAQIDNQNDPEVGQQS 838

Pan QLQAQIDNQNDPEVGQQS 838

Pongo QLQAQIDNQNDPEVGQQS 838

Nomascus QLQAQIDNQNDPEVGQQS 826

Callithrix QLQAQIDNQNDPEVGQQS 804

Mus QLQIQIDNQNDPEEGQ-- 826

Rattus QLQIQIDNQNEPEEGQ-- 836

:** *****::** **
