# Supplementary material for: Pitchfork and Gprasp2 Target Smoothened to the Primary Cilium for Hedgehog Pathway Activation
Source: PLoS One. 2016 Feb 22;11(2):e0149477. doi: 10.1371/journal.pone.0149477 (PMC4763541; doi:10.1371/journal.pone.0149477)
Supplement: S1 Supporting Information — (PDF) [file pone.0149477.s007.pdf]

Supplementary Modeling Supporting Information

Contents

1 Model: Pifo+ 2

1.1 Experiment: Pifo$^{wt/wt}$ . . . 6
1.2 Experiment: Pifo Rescue Clone 1 . . . 8
1.3 Experiment: Pifo Rescue Clone 2 . . . 10
1.4 Experiment: Pifo Rescue Clone 3 . . . 11
1.5 Experiment: Pifo Rescue Clone 4 . . . 12
1.6 Experiment: Pifo Rescue Clone 5 . . . 13
1.7 Experiment: Pifo$^{FD/FD}$ . . . 14

2 Estimated model parameters 15

3 Profile likelihood of model parameters 18

4 Confidence intervals for the model parameters 27

5 Model: Pifo- 30

5.1 Experiment: Pifo$^{wt/wt}$ . . . 34
5.2 Experiment: Pifo Rescue Clone 1 . . . 36
5.3 Experiment: Pifo Rescue Clone 2 . . . 38
5.4 Experiment: Pifo Rescue Clone 3 . . . 39
5.5 Experiment: Pifo Rescue Clone 4 . . . 40
5.6 Experiment: Pifo Rescue Clone 5 . . . 41
5.7 Experiment: Pifo$^{FD/FD}$ . . . 42

6 Estimated model parameters 43

7 Profile likelihood of model parameters 46

8 Confidence intervals for the model parameters 55

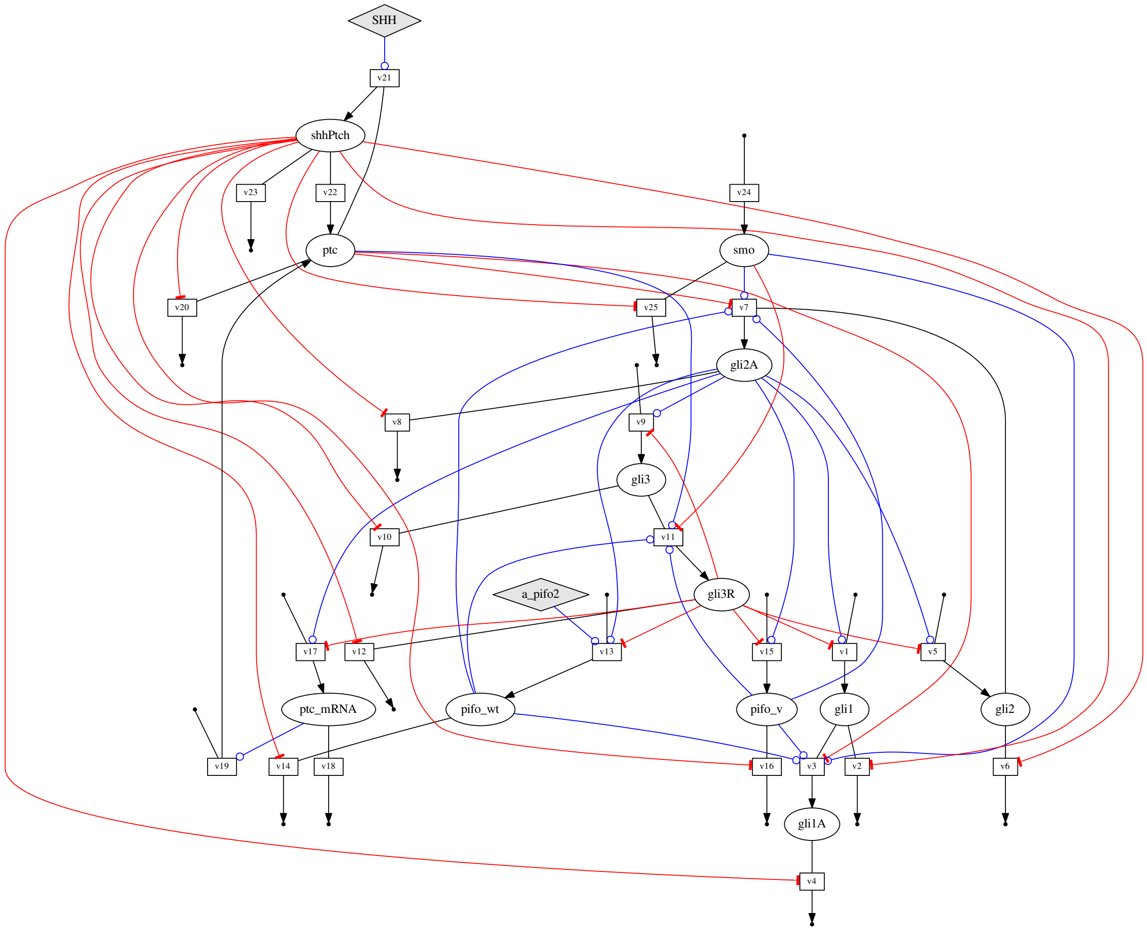

Figure 1: Network representation of the model Pifo+
Diamond shaped nodes correspond to model inputs, see Equation 140 – 141. Ellipsoid shaped nodes correspond to dynamical variables described by the ODE system, see Equation 167 – 178. Black arrows and box shaped nodes indicate reactions with corresponding rate equations given in Equation 142 – 166. Red T-shaped arrows indicated inhibitorial influence on a reaction and blue O-shaped arrows indicate catalysing influence on a reaction.

1 Model: Pifo+

The model consists of 2 external inputs, 12 dynamical variables indicated by square brackets and 25 reactions and was evaluated for 7 experimental conditions. In total 91 parameters are estimated from the experimental data, yielding a value of the objective function $-2\log(L) = -590.646$ for a total of 613 data points. The estimated parameter values are given in Section 6.

The model dynamics depends on the external inputs:

$$[\mathrm{SHH}](t) = \mathrm{SHH\_level} \quad (1)$$

$$a\_pifo2(t) = a\_wt2 \quad (2)$$

The rate equations corresponding to the reactions included in the model are give by:

$$v_1 = \frac{\mathrm{a1} \cdot [\mathrm{gli2A}]}{[\mathrm{gli3R}] \cdot \mathrm{r0} + 1} \quad (3)$$

$$v_2 = \frac{\mathrm{b1} \cdot [\mathrm{gli1}]}{\mathrm{g1} \cdot [\mathrm{shhPtch}] + 1} \quad (4)$$

$$v_3 = \frac{[\mathrm{gli1}] \cdot [\mathrm{smo}] \cdot \mathrm{t1} + [\mathrm{gli1}] \cdot [\mathrm{pifo\_v}] \cdot [\mathrm{smo}] \cdot \mathrm{t2} + [\mathrm{gli1}] \cdot \mathrm{pifo\_w}t \cdot [\mathrm{smo}] \cdot \mathrm{t2}}{[\mathrm{ptc}] \cdot \mathrm{q0} + 1} \quad (5)$$

$$v_4 = \frac{\mathrm{b2} \cdot [\mathrm{gli1A}]}{\mathrm{g2} \cdot [\mathrm{shhPtch}] + 1} \quad (6)$$

$$v_5 = \frac{\mathrm{a3} \cdot [\mathrm{gli2A}]}{[\mathrm{gli3R}] \cdot \mathrm{r1} + 1} \quad (7)$$

$$v_6 = \frac{\mathrm{b3} \cdot [\mathrm{gli2}]}{\mathrm{g3} \cdot [\mathrm{shhPtch}] + 1} \quad (8)$$

$$v_7 = \frac{[\mathrm{gli2}] \cdot [\mathrm{smo}] \cdot \mathrm{t3} + [\mathrm{gli2}] \cdot [\mathrm{pifo\_v}] \cdot [\mathrm{smo}] \cdot \mathrm{t4} + [\mathrm{gli2}] \cdot \mathrm{pifo\_w}t \cdot [\mathrm{smo}] \cdot \mathrm{t4}}{[\mathrm{ptc}] \cdot \mathrm{q0} + 1} \quad (9)$$

$$v_8 = \frac{\mathrm{b4} \cdot [\mathrm{gli2A}]}{\mathrm{g4} \cdot [\mathrm{shhPtch}] + 1} \quad (10)$$

$$v_9 = \frac{\mathrm{a5} \cdot [\mathrm{gli2A}]}{[\mathrm{gli3R}] \cdot \mathrm{r2} + 1} \quad (11)$$

$$v_{10} = \frac{\mathrm{b5} \cdot [\mathrm{gli3}]}{\mathrm{g5} \cdot [\mathrm{shhPtch}] + 1} \quad (12)$$

$$v_{11} = \frac{[\mathrm{gli3}] \cdot [\mathrm{ptc}] \cdot \mathrm{t5} + [\mathrm{gli3}] \cdot [\mathrm{pifo\_v}] \cdot [\mathrm{ptc}] \cdot \mathrm{t6} + [\mathrm{gli3}] \cdot \mathrm{pifo\_w}t \cdot [\mathrm{ptc}] \cdot \mathrm{t6}}{[\mathrm{smo}] \cdot (\mathrm{q2} + [\mathrm{pifo\_v}] \cdot \mathrm{q1} + \mathrm{pifo\_w}t \cdot \mathrm{q1}) + 1} \quad (13)$$

$$v_{12} = \frac{\mathrm{b6} \cdot [\mathrm{gli3R}]}{\mathrm{g6} \cdot [\mathrm{shhPtch}] + 1} \quad (14)$$

$$v_{13} = \frac{a\_pifo2 \cdot [\mathrm{gli2A}]}{[\mathrm{gli3R}] \cdot \mathrm{r3} + 1} \quad (15)$$

$$v_{14} = \frac{\mathrm{b7} \cdot \mathrm{pifo\_w}t}{\mathrm{g7} \cdot [\mathrm{shhPtch}] + 1} \quad (16)$$

$$v_{15} = \frac{a\_v2 \cdot [\mathrm{gli2A}]}{[\mathrm{gli3R}] \cdot \mathrm{r3} + 1} \quad (17)$$

$$v_{16} = \frac{\mathrm{b7} \cdot [\mathrm{pifo\_v}]}{\mathrm{g7} \cdot [\mathrm{shhPtch}] + 1} \quad (18)$$

$$v_{17} = \frac{[\mathrm{gli2A}] \cdot \mathrm{k1}}{[\mathrm{gli3R}] \cdot \mathrm{r4} + 1} \quad (19)$$

$$v_{18} = \mathrm{b9} \cdot [\mathrm{ptc\_mRNA}] \quad (20)$$

$$v_{19} = \mathrm{a15} \cdot [\mathrm{ptc\_mRNA}] \quad (21)$$

$$v_{20} = \frac{\mathrm{b10} \cdot [\mathrm{ptc}]}{\mathrm{g9} \cdot [\mathrm{shhPtch}] + 1} \quad (22)$$

$$v_{21} = \mathrm{a19} \cdot [\mathrm{ptc}] \cdot ([\mathrm{SHH}] + \mathrm{SHH\_b}) \quad (23)$$

$$v_{22} = \mathrm{d1} \cdot [\mathrm{shhPtch}] \quad (24)$$

$$v_{23} = \mathrm{b11} \cdot [\mathrm{shhPtch}] \quad (25)$$

$$v_{24} = \mathrm{smo\_base} \quad (26)$$

$$v_{25} = \frac{\mathrm{b12} \cdot [\mathrm{smo}]}{\mathrm{g10} \cdot [\mathrm{shhPtch}] + 1} \quad (27)$$

The ODE system determining the time evolution of the dynamical variables is given by:

$$\mathrm{d[gli1]/d}t = +v_1 - v_2 - v_3 \tag{28}$$
$$\mathrm{d[gli1A]/d}t = +v_3 - v_4 \tag{29}$$
$$\mathrm{d[gli2]/d}t = +v_5 - v_6 - v_7 \tag{30}$$
$$\mathrm{d[gli2A]/d}t = +v_7 - v_8 \tag{31}$$
$$\mathrm{d[gli3]/d}t = +v_9 - v_{10} - v_{11} \tag{32}$$
$$\mathrm{d[gli3R]/d}t = +v_{11} - v_{12} \tag{33}$$
$$\mathrm{dpifo\_w}t\mathrm{/d}t = +v_{13} - v_{14} \tag{34}$$
$$\mathrm{d[pifo\_v]/d}t = +v_{15} - v_{16} \tag{35}$$
$$\mathrm{d[ptc\_mRNA]/d}t = +v_{17} - v_{18} \tag{36}$$
$$\mathrm{d[ptc]/d}t = +v_{19} - v_{20} - v_{21} + v_{22} \tag{37}$$

$$\mathrm{d[smo]/d}t = +v_{24} - v_{25} \tag{38}$$
$$\mathrm{d[shhPtch]/d}t = +v_{21} - v_{22} - v_{23} \tag{39}$$

The ODE system was solved by a parallelized implementation of the CVODES algorithm [1]. It also supplies the parameter sensitivities utilized for parameter estimation.

The initial conditions for the ODE system are given by:

$$\mathrm{[gli1](0)} = \mathrm{init\_gli1} \tag{40}$$
$$\mathrm{[gli1A](0)} = \mathrm{init\_gli1A} \tag{41}$$
$$\mathrm{[gli2](0)} = \mathrm{init\_gli2} \tag{42}$$
$$\mathrm{[gli2A](0)} = \mathrm{init\_gli2A} \tag{43}$$
$$\mathrm{[gli3](0)} = \mathrm{init\_gli3} \tag{44}$$
$$\mathrm{[gli3R](0)} = \mathrm{init\_gli3R} \tag{45}$$
$$\mathrm{pifo\_w}t\mathrm{(0)} = \mathrm{init\_pifo\_w}t \tag{46}$$
$$\mathrm{[pifo\_v](0)} = \mathrm{init\_pifo\_v} \tag{47}$$
$$\mathrm{[ptc\_mRNA](0)} = \mathrm{init\_ptc\_mRNA} \tag{48}$$
$$\mathrm{[ptc](0)} = \mathrm{init\_ptc} \tag{49}$$

$$\mathrm{[smo](0)} = \mathrm{init\_smo} \tag{50}$$
$$\mathrm{[shhPtch](0)} = \mathrm{init\_shhPtch} \tag{51}$$

The ODE system is modified by the following parameter transformations:

$$\mathrm{a1} \rightarrow \frac{(\mathrm{init\_gli3R} \cdot \mathrm{r0} + 1) \cdot (\mathrm{b1} \cdot \mathrm{init\_gli1} + \mathrm{init\_gli1} \cdot \mathrm{init\_smo} \cdot \mathrm{t1} + \mathrm{b1} \cdot \mathrm{init\_gli1} \cdot \mathrm{init\_ptc} \cdot \mathrm{q0} + \mathrm{init\_gli1} \cdot \mathrm{init\_smo} \\ 1 \cdot \mathrm{init\_pifo\_}wt \cdot \mathrm{t2} + \mathrm{g1} \cdot \mathrm{init\_gli1} \cdot \mathrm{init\_smo} \cdot \mathrm{init\_shhPtch} \cdot \mathrm{t1} + \mathrm{g1} \cdot \mathrm{init\_gli1} \cdot \mathrm{init\_smo} \cdot \mathrm{init\_shhPtch} \cdot \mathrm{init\_pifo\_}wt \cdot \mathrm{t2})}{\mathrm{init\_gli2A} \cdot (\mathrm{g1} \cdot \mathrm{init\_shhPtch} + 1) \cdot (\mathrm{init\_ptc} \cdot \mathrm{q0} + 1)} \tag{52}$$

$$\mathrm{a15} \rightarrow \frac{\mathrm{b11} \cdot \mathrm{g9} \cdot \mathrm{init\_shhPtch}^2 + \mathrm{b11} \cdot \mathrm{init\_shhPtch} + \mathrm{b10} \cdot \mathrm{init\_ptc}}{\mathrm{init\_ptc\_mRNA} \cdot (\mathrm{g9} \cdot \mathrm{init\_shhPtch} + 1)} \tag{53}$$

$$\mathrm{a19} \rightarrow \frac{\mathrm{b11} \cdot \mathrm{init\_shhPtch} + \mathrm{d1} \cdot \mathrm{init\_shhPtch}}{\mathrm{SHH\_b} \cdot \mathrm{init\_ptc}} \tag{54}$$

$$\mathrm{a3} \rightarrow \frac{(\mathrm{init\_gli3R} \cdot \mathrm{r1} + 1) \cdot (\mathrm{b3} \cdot \mathrm{init\_gli2} + \mathrm{init\_gli2} \cdot \mathrm{init\_smo} \cdot \mathrm{t3} + \mathrm{b3} \cdot \mathrm{init\_gli2} \cdot \mathrm{init\_ptc} \cdot \mathrm{q0} + \mathrm{init\_gli2} \cdot \mathrm{init\_smo} \\ 1 \cdot \mathrm{init\_pifo\_}wt \cdot \mathrm{t4} + \mathrm{g3} \cdot \mathrm{init\_gli2} \cdot \mathrm{init\_smo} \cdot \mathrm{init\_shhPtch} \cdot \mathrm{t3} + \mathrm{g3} \cdot \mathrm{init\_gli2} \cdot \mathrm{init\_smo} \cdot \mathrm{init\_shhPtch} \cdot \mathrm{init\_pifo\_}wt \cdot \mathrm{t4})}{\mathrm{init\_gli2A} \cdot (\mathrm{g3} \cdot \mathrm{init\_shhPtch} + 1) \cdot (\mathrm{init\_ptc} \cdot \mathrm{q0} + 1)} \tag{55}$$

$$\mathrm{a5} \rightarrow \frac{(\mathrm{init\_gli3R} \cdot \mathrm{r2} + 1) \cdot (\mathrm{b5} \cdot \mathrm{init\_gli3} + \mathrm{init\_gli3} \cdot \mathrm{init\_ptc} \cdot \mathrm{t5} + \mathrm{b5} \cdot \mathrm{init\_gli3} \cdot \mathrm{init\_smo} \cdot \mathrm{q2} + \mathrm{init\_gli3} \cdot \mathrm{init\_ptc} \\ 1 \cdot \mathrm{init\_pifo\_}wt \cdot \mathrm{t6} + \mathrm{b5} \cdot \mathrm{init\_gli3} \cdot \mathrm{init\_smo} \cdot \mathrm{init\_pifo\_}wt \cdot \mathrm{q1} + \mathrm{g5} \cdot \mathrm{init\_gli3} \cdot \mathrm{init\_ptc} \cdot \mathrm{init\_shhPtch} \cdot \mathrm{t5} + \mathrm{g5} \cdot \mathrm{init\_gli3} \cdot \mathrm{init\_ptc} \cdot \mathrm{init\_shhPtch} \cdot \mathrm{init\_pifo\_}wt \cdot \mathrm{t6})}{\mathrm{init\_gli2A} \cdot (\mathrm{g5} \cdot \mathrm{init\_shhPtch} + 1) \cdot (\mathrm{init\_smo} \cdot \mathrm{q2} + \mathrm{init\_smo} \cdot \mathrm{init\_pifo\_}wt \cdot \mathrm{q1} + 1)} \tag{56}$$

$$\mathrm{b2} \rightarrow \frac{(\mathrm{init\_gli1} \cdot \mathrm{init\_smo} \cdot \mathrm{t1} + \mathrm{init\_gli1} \cdot \mathrm{init\_smo} \cdot \mathrm{init\_pifo\_}wt \cdot \mathrm{t2}) \cdot (\mathrm{g2} \cdot \mathrm{init\_shhPtch} + 1)}{\mathrm{init\_gli1A} \cdot (\mathrm{init\_ptc} \cdot \mathrm{q0} + 1)} \tag{57}$$

$$\mathrm{b4} \rightarrow \frac{(\mathrm{init\_gli2} \cdot \mathrm{init\_smo} \cdot \mathrm{t3} + \mathrm{init\_gli2} \cdot \mathrm{init\_smo} \cdot \mathrm{init\_pifo\_}wt \cdot \mathrm{t4}) \cdot (\mathrm{g4} \cdot \mathrm{init\_shhPtch} + 1)}{\mathrm{init\_gli2A} \cdot (\mathrm{init\_ptc} \cdot \mathrm{q0} + 1)} \tag{58}$$

$$\mathrm{b6} \rightarrow \frac{(\mathrm{init\_gli3} \cdot \mathrm{init\_ptc} \cdot \mathrm{t5} + \mathrm{init\_gli3} \cdot \mathrm{init\_ptc} \cdot \mathrm{init\_pifo\_}wt \cdot \mathrm{t6}) \cdot (\mathrm{g6} \cdot \mathrm{init\_shhPtch} + 1)}{\mathrm{init\_gli3R} \cdot (\mathrm{init\_smo} \cdot \mathrm{q2} + \mathrm{init\_smo} \cdot \mathrm{init\_pifo\_}wt \cdot \mathrm{q1} + 1)} \tag{59}$$

$$\mathrm{b7} \rightarrow \frac{a\_wt2 \cdot \mathrm{init\_gli2A} \cdot (\mathrm{g7} \cdot \mathrm{init\_shhPtch} + 1)}{\mathrm{init\_pifo\_}wt \cdot (\mathrm{init\_gli3R} \cdot \mathrm{r3} + 1)} \tag{60}$$

$$\mathrm{b9} \rightarrow \frac{\mathrm{init\_gli2A} \cdot \mathrm{k1}}{\mathrm{init\_ptc\_mRNA} \cdot (\mathrm{init\_gli3R} \cdot \mathrm{r4} + 1)} \tag{61}$$

$$\mathrm{smo\_base} \rightarrow \frac{\mathrm{b12} \cdot \mathrm{init\_smo}}{\mathrm{g10} \cdot \mathrm{init\_shhPtch} + 1} \tag{62}$$

(63)

1.1 Experiment: $\text{Pifo}^{wt/wt}$

The model outputs available in this data set are defined by:

$$\begin{aligned}
\text{gli1_gli1A_obs1} &= \text{scale_gli11} \cdot ([\text{gli1}] + [\text{gli1A}]) && (64)\\
\text{gli1_gli1A_obs2} &= \text{scale_gli12} \cdot ([\text{gli1}] + [\text{gli1A}]) && (65)\\
\text{gli1_gli1A_obs3} &= \text{scale_gli13} \cdot ([\text{gli1}] + [\text{gli1A}]) && (66)\\
\text{gli2_gli2A_obs1} &= \text{scale_gli21} \cdot ([\text{gli2}] + [\text{gli2A}]) && (67)\\
\text{gli2_gli2A_obs2} &= \text{scale_gli22} \cdot ([\text{gli2}] + [\text{gli2A}]) && (68)\\
\text{gli2_gli2A_obs3} &= \text{scale_gli23} \cdot ([\text{gli2}] + [\text{gli2A}]) && (69)\\
\text{gli3_obs1} &= [\text{gli3}] \cdot \text{scale_gli31} && (70)\\
\text{gli3_obs2} &= [\text{gli3}] \cdot \text{scale_gli3} && (71)\\
\text{pifo_obs1} &= \text{pifo_}wt \cdot \text{scale_pifo1} && (72)\\
\text{pifo_obs2} &= \text{pifo_}wt \cdot \text{scale_pifo2} && (73)
\end{aligned}$$

$$\begin{aligned}
\text{gli3R_obs1} &= [\text{gli3R}] \cdot \text{scale_gli3R1} && (74)\\
\text{gli3R_obs2} &= [\text{gli3R}] \cdot \text{scale_gli3} && (75)\\
\text{ptc_mRNA_obs} &= [\text{ptc_mRNA}] \cdot \text{scale_mRNA} && (76)
\end{aligned}$$

The error model that describes the measurement noise for each model output is given by:

$$\begin{aligned}
\text{gli1_gli1A_obs1} &= \text{sd_gli1_gli1A} && (77)\\
\text{gli1_gli1A_obs2} &= \text{sd_gli1_gli1A} && (78)\\
\text{gli1_gli1A_obs3} &= \text{sd_gli1_gli1A} && (79)\\
\text{gli2_gli2A_obs1} &= \text{sd_gli2_gli2A} && (80)\\
\text{gli2_gli2A_obs2} &= \text{sd_gli2_gli2A} && (81)\\
\text{gli2_gli2A_obs3} &= \text{sd_gli2_gli2A} && (82)\\
\text{gli3_obs1} &= \text{sd_gli3} && (83)\\
\text{gli3_obs2} &= \text{sd_gli3} && (84)\\
\text{pifo_obs1} &= \text{sd_pifo} && (85)\\
\text{pifo_obs2} &= \text{sd_pifo} && (86)
\end{aligned}$$

$$\begin{aligned}
\text{gli3R_obs1} &= \text{sd_gli3R} && (87)\\
\text{gli3R_obs2} &= \text{sd_gli3R} && (88)\\
\text{ptc_mRNA_obs} &= \text{sd_mRNA} && (89)
\end{aligned}$$

To evaluate the ODE system of Equation 167 – 190 for the conditions in this experiment, the following parameter transformations are applied:

$$\begin{aligned}
a_v2 &\rightarrow 0\\
\text{init_pifo_v} &\rightarrow 0
\end{aligned}$$

The agreement of the model outputs and the experimental data, given in Table 16, yields a value of the objective function $-2\log(L) = -315.208$ for 194 data points in this data set.

time [hour]	gli1_gli1A_obs1 conc. [au]	gli1_gli1A_obs2 conc. [au]	gli1_gli1A_obs3 conc. [au]	gli2_gli2A_obs1 conc. [au]	gli2_gli2A_obs2 conc. [au]	gli2_gli2A_obs3 conc. [au]	gli3_obs1 conc. [au]	gli3_obs2 conc. [au]	pifo_obs1 conc. [au]	pifo_obs2 conc. [au]	gli3R_obs1 conc. [au]	gli3R_obs2 conc. [au]	ptc_mRNA_obs conc. [au]
0.0	0.015564	0.0214446	0.0363197	0.178222	0.200916	0.056895	0.212785	0.000676336	0.0628137	0.0471802	0.54038	NaN	0.0177638
0.0	0.00855469	0.0203686	0.0361234	0.185775	0.313453	0.0571	NaN	NaN	NaN	0.046892	NaN	0.774863	NaN
0.0	0.0199044	0.0278261	0.0522542	0.198058	0.342151	0.0541885	NaN	NaN	NaN	0.041193	NaN	NaN	NaN
0.5	0.0241291	0.0367793	0.0880664	0.434706	0.224972	0.355579	0.286852	0.313212	0.138315	0.136339	0.668428	NaN	0.0538905
0.5	0.0389373	0.0330013	0.0756739	0.416143	0.364545	0.290495	NaN	NaN	NaN	0.108673	NaN	0.87813	NaN
0.5	0.0360606	0.0519847	0.0880195	0.427444	0.344853	0.382099	NaN	NaN	NaN	0.170844	NaN	NaN	NaN
1.0	0.0303636	0.0558414	0.128295	0.391266	0.370038	0.293007	0.320029	0.445293	0.532863	0.25741	0.833513	NaN	0.0866669
1.0	0.0853232	0.0449743	0.120734	0.408369	0.554766	0.291326	NaN	NaN	NaN	0.233159	NaN	0.914891	NaN
1.0	0.0980148	0.084227	0.13074	0.429824	0.570069	0.450989	NaN	NaN	NaN	0.372068	NaN	NaN	NaN
2.0	0.0750276	0.0784564	0.164607	0.723385	0.351728	0.600716	0.739916	0.945608	1	0.791907	0.842011	NaN	0.305466
2.0	0.147758	0.0638573	0.15793	0.72222	0.603407	0.561212	NaN	NaN	NaN	0.707039	NaN	1	NaN
2.0	0.163165	0.0986687	0.165783	0.738104	0.585102	0.775977	NaN	NaN	NaN	1	NaN	NaN	NaN
3.0	NaN	NaN	NaN	NaN	NaN	NaN	NaN	NaN	NaN	NaN	NaN	NaN	NaN
3.0	NaN	NaN	NaN	NaN	NaN	NaN	NaN	NaN	NaN	NaN	NaN	NaN	NaN
3.0	NaN	NaN	NaN	NaN	NaN	NaN	NaN	NaN	NaN	NaN	NaN	NaN	NaN
4.0	0.128378	0.103976	0.365019	0.991599	0.623771	1	1	1	0.811109	0.493087	0.897436	NaN	0.589372
4.0	0.202314	0.0835847	0.23147	0.929583	0.957862	0.932811	NaN	NaN	NaN	0.358881	NaN	0.979466	NaN
4.0	0.213664	0.150569	0.219145	1	1	0.978299	NaN	NaN	NaN	0.674253	NaN	NaN	NaN
6.0	0.218872	0.231773	0.381829	0.757754	0.588877	0.734054	0.839698	1	0.644192	0.33405	1	NaN	1
6.0	0.400663	0.230555	0.402404	0.821881	0.953563	0.778089	NaN	NaN	NaN	0.296624	NaN	NaN	NaN
6.0	0.437522	0.35172	0.373627	0.803729	0.978667	0.903556	NaN	NaN	NaN	0.458962	NaN	NaN	NaN
24.0	0.843905	0.717574	1	0.546695	0.526224	0.545752	0.419506	0.55356	0.554984	0.321622	0.211071	NaN	0.771944
24.0	0.971295	0.694357	0.787677	0.61271	0.819188	0.524619	NaN	NaN	NaN	0.258336	NaN	0.529352	NaN
24.0	1	1	0.996629	0.596012	0.869489	0.728985	NaN	NaN	NaN	0.463376	NaN	NaN	NaN
48.0	0.158902	NaN	NaN	NaN	0.367042	NaN	NaN	NaN	NaN	NaN	NaN	NaN	NaN
48.0	0.278934	NaN	NaN	NaN	0.564599	NaN	NaN	NaN	NaN	NaN	NaN	NaN	NaN
48.0	0.399441	NaN	NaN	NaN	0.600441	NaN	NaN	NaN	NaN	NaN	NaN	NaN	NaN

Table 1: Experimental data for the experiment Pifo$^{wt/wt}$

1.2 Experiment: Pifo Rescue Clone 1

The model outputs available in this data set are defined by:

$$\text{gli1\_gli1A\_obs} = \text{scale\_gli1\_clone03} \cdot ([\text{gli1}] + [\text{gli1A}]) \quad (90)$$

$$\text{gli2\_gli2A\_obs} = \text{scale\_gli2\_clone03} \cdot ([\text{gli2}] + [\text{gli2A}]) \quad (91)$$

$$\text{venus\_pifo\_obs} = [\text{pifo\_v}] \cdot \text{scale\_venus\_pifo03} \quad (92)$$

$$\text{wt\_pifo\_obs} = \frac{\text{pifo\_w}t}{\text{init\_pifo\_w}t} \quad (93)$$

$$\text{dummy\_pifo\_obs} = \text{scale\_dummy} \cdot ([\text{pifo\_v}] + \text{pifo\_w}t) \quad (94)$$

The error model that describes the measurement noise for each model output is given by:

$$\text{gli1\_gli1A\_obs} = \text{sd\_gli1\_gli1A\_clone} \quad (95)$$

$$\text{gli2\_gli2A\_obs} = \text{sd\_gli2\_gli2A\_clone} \quad (96)$$

$$\text{venus\_pifo\_obs} = \text{sd\_venus\_pifo} \quad (97)$$

$$\text{wt\_pifo\_obs} = 0.01 \quad (98)$$

$$\text{dummy\_pifo\_obs} = 0.05 \quad (99)$$

To evaluate the ODE system of Equation 167 – 178 for the conditions in this experiment, the following external inputs are given:

$$[\text{SHH}](t) = -\text{SHH\_level} \cdot \left(\frac{1}{\mathrm{e}^{100000000.0 \cdot t - 10000000000.0} + 1} - 1 \right) \quad (100)$$

$$a\_pifo2(t) = \frac{a\_wt2}{\mathrm{e}^{100000000.0 \cdot t - 5000000000.0} + 1} \quad (101)$$

To evaluate the ODE system of Equation 167 – 190 for the conditions in this experiment, the following parameter transformations are applied:

$$a\_v2 \rightarrow a\_v32$$
$$\text{init\_pifo\_v} \rightarrow 0$$

The agreement of the model outputs and the experimental data, given in Table 17, yields a value of the objective function $-2\log(L) = -107.704$ for 84 data points in this data set.

time [hour]	gli1_gli1A_obs conc. [au]	gli2_gli2A_obs conc. [au]	venus_pifo_obs conc. [au]	wt_pifo_obs conc. [au]	dummy_pifo_obs conc. [au]
40.000000	NaN	NaN	NaN	NaN	1
41.000000	NaN	NaN	NaN	NaN	1
42.000000	NaN	NaN	NaN	NaN	1
43.000000	NaN	NaN	NaN	NaN	1
44.000000	NaN	NaN	NaN	NaN	1
45.000000	NaN	NaN	NaN	NaN	1
46.000000	NaN	NaN	NaN	NaN	1
47.000000	NaN	NaN	NaN	NaN	1
48.000000	NaN	NaN	NaN	NaN	1
49.000000	NaN	NaN	NaN	NaN	1
50.000000	NaN	NaN	NaN	NaN	1
100.000000	0.182833	0.463423	0.120944	0.01	NaN
100.000000	0.240915	0.692974	0.0922276	NaN	NaN
100.000000	0.282956	0.739093	0.153121	NaN	NaN
100.500000	0.0575001	0.540771	0.567789	NaN	NaN
100.500000	0.114786	0.783652	0.46482	NaN	NaN
100.500000	0.112337	0.841869	0.721076	NaN	NaN
101.000000	0.0885261	0.523308	0.652547	NaN	NaN
101.000000	0.103852	0.708793	0.535404	NaN	NaN
101.000000	0.117757	0.752356	0.733981	NaN	NaN
102.000000	0.130983	0.676713	0.792149	NaN	NaN
102.000000	0.136415	0.945411	0.595297	NaN	NaN
102.000000	0.177838	1	1	NaN	NaN
104.000000	0.267093	0.427406	0.29869	NaN	NaN
104.000000	0.299726	0.706476	0.276722	NaN	NaN
104.000000	0.285777	0.691151	0.405361	NaN	NaN
106.000000	0.35012	0.386636	0.0498918	NaN	NaN
106.000000	0.347542	0.69705	0.0924	NaN	NaN
106.000000	0.40814	0.738608	0.124777	NaN	NaN
124.000000	1	0.120606	0.0490282	NaN	NaN
124.000000	0.953868	0.228043	0.0269504	NaN	NaN
124.000000	0.992917	0.248928	0.168091	NaN	NaN
148.000000	0.746878	0.00927686	0.112661	NaN	NaN
148.000000	0.721883	0.0150845	0.106491	NaN	NaN
148.000000	0.725758	0.0154096	0.359014	NaN	NaN

Table 2: Experimental data for the experiment Pifo Rescue Clone 1

time [hour]	gli1_gli1A_obs conc. [au]	gli2_gli2A_obs conc. [au]	venus_pifo_obs conc. [au]
100.000000	0.0368266	0.184742	0.0916695
100.000000	0.0560124	0.261114	0.112316
100.000000	0.0644724	0.316429	0.253573
100.500000	0.0718045	0.192446	0.193969
100.500000	0.111495	0.365173	0.199484
100.500000	0.0747356	0.362317	0.463917
101.000000	0.045422	0.634537	0.158465
101.000000	0.0799631	0.995833	0.314363
101.000000	0.0843231	0.970766	0.583984
102.000000	0.0648619	0.612743	0.466756
102.000000	0.0868798	0.978037	0.572587
102.000000	0.0550461	1	1
104.000000	0.0669407	0.207321	0.342571
104.000000	0.107634	0.37937	0.50336
104.000000	0.0402828	0.389485	0.870908
106.000000	0.0690348	0.279133	0.355211
106.000000	0.118163	0.476426	0.272168
106.000000	0.0375131	0.489825	NaN
124.000000	0.166348	0.00811415	0.0857238
124.000000	0.189582	0.00643713	0.164051
124.000000	0.186287	0.00174642	0.35449
148.000000	0.853527	0.00964686	0.125416
148.000000	0.931884	0.0131668	0.110746
148.000000	1	0.014068	0.256562

Table 3: Experimental data for the experiment Pifo Rescue Clone 2

1.3 Experiment: Pifo Rescue Clone 2

The model outputs available in this data set are defined by:

$$\text{gli1\_gli1A\_obs} = \text{scale\_gli1\_clone04} \cdot ([\text{gli1}] + [\text{gli1A}]) \tag{102}$$
$$\text{gli2\_gli2A\_obs} = \text{scale\_gli2\_clone04} \cdot ([\text{gli2}] + [\text{gli2A}]) \tag{103}$$
$$\text{venus\_pifo\_obs} = [\text{pifo\_v}] \cdot \text{scale\_venus\_pifo04} \tag{104}$$

The error model that describes the measurement noise for each model output is given by:

$$\text{gli1\_gli1A\_obs} = \text{sd\_gli1\_gli1A\_clone} \tag{105}$$
$$\text{gli2\_gli2A\_obs} = \text{sd\_gli2\_gli2A\_clone} \tag{106}$$
$$\text{venus\_pifo\_obs} = \text{sd\_venus\_pifo} \tag{107}$$

To evaluate the ODE system of Equation 167 – 178 for the conditions in this experiment, the following external inputs are given:

$$[\text{SHH}](t) = -\text{SHH\_level} \cdot \left(\frac{1}{e^{100000000.0 \cdot t - 10000000000.0} + 1} - 1 \right) \tag{108}$$
$$a\_pifo2(t) = \frac{a\_wt2}{e^{100000000.0 \cdot t - 5000000000.0} + 1} \tag{109}$$

To evaluate the ODE system of Equation 167 – 190 for the conditions in this experiment, the following parameter transformations are applied:

$$a\_v2 \rightarrow a\_v42$$
$$\text{init\_pifo\_v} \rightarrow 0$$

The agreement of the model outputs and the experimental data, given in Table 18, yields a value of the objective function $-2\log(L) = -68.9435$ for 71 data points in this data set.

time [hour]	gli1_gli1A_obs conc. [au]	gli2_gli2A_obs conc. [au]	venus_pifo_obs conc. [au]
100.000000	0.034737	0.10516	0.0531111
100.000000	0.0370827	0.180143	0.00408836
100.000000	0.0367413	0.206412	0.0279515
100.500000	0.0479489	0.213912	0.035578
100.500000	0.0576734	0.302668	0.0241159
100.500000	0.115088	0.352421	0.0622776
101.000000	0.0583906	0.320974	0.0387209
101.000000	0.0536062	0.506592	0.0330925
101.000000	0.0702539	0.534023	0.109485
102.000000	0.0793571	0.371164	0.377696
102.000000	0.0962706	0.584093	0.354038
102.000000	0.0960704	0.617612	0.402495
104.000000	0.227568	0.612039	0.538538
104.000000	0.194355	0.902327	0.641777
104.000000	0.252053	1	1
106.000000	0.20249	0.240238	0.464928
106.000000	0.207116	0.3771	0.331168
106.000000	0.197015	0.426412	0.705254
124.000000	0.891786	0.0148126	0.071469
124.000000	0.920639	0.0191236	0.0520516
124.000000	1	0.0388116	0.223205
148.000000	0.828158	0.0135249	0.0600894
148.000000	0.836879	0.0148126	0.0447066
148.000000	0.889168	0.0329954	0.219787

Table 4: Experimental data for the experiment Pifo Rescue Clone 3

1.4 Experiment: Pifo Rescue Clone 3

The model outputs available in this data set are defined by:

$$\mathrm{gli1\_gli1A\_obs} = \mathrm{scale\_gli1\_clone06} \cdot ([\mathrm{gli1}] + [\mathrm{gli1A}]) \tag{110}$$
$$\mathrm{gli2\_gli2A\_obs} = \mathrm{scale\_gli2\_clone06} \cdot ([\mathrm{gli2}] + [\mathrm{gli2A}]) \tag{111}$$
$$\mathrm{venus\_pifo\_obs} = [\mathrm{pifo\_v}] \cdot \mathrm{scale\_venus\_pifo06} \tag{112}$$

The error model that describes the measurement noise for each model output is given by:

$$\mathrm{gli1\_gli1A\_obs} = \mathrm{sd\_gli1\_gli1A\_clone} \tag{113}$$
$$\mathrm{gli2\_gli2A\_obs} = \mathrm{sd\_gli2\_gli2A\_clone} \tag{114}$$
$$\mathrm{venus\_pifo\_obs} = \mathrm{sd\_venus\_pifo} \tag{115}$$

To evaluate the ODE system of Equation 167 – 178 for the conditions in this experiment, the following external inputs are given:

$$[\mathrm{SHH}](t) = -\mathrm{SHH\_level} \cdot \left(\frac{1}{e^{100000000.0 \cdot t - 10000000000.0} + 1} - 1 \right) \tag{116}$$
$$a\_pifo2(t) = \frac{a\_wt2}{e^{100000000.0 \cdot t - 5000000000.0} + 1} \tag{117}$$

To evaluate the ODE system of Equation 167 – 190 for the conditions in this experiment, the following parameter transformations are applied:

$$a\_v2 \rightarrow a\_v62$$
$$\mathrm{init\_pifo\_v} \rightarrow 0$$

The agreement of the model outputs and the experimental data, given in Table 19, yields a value of the objective function $-2\log(L) = -75.0957$ for 72 data points in this data set.

time [hour]	gli1_gli1A_obs conc. [au]	gli2_gli2A_obs conc. [au]	venus_pifo_obs conc. [au]
100.000000	0.0733807	0.0557874	0.155678
100.000000	0.0752167	0.109196	0.0967391
100.000000	0.137857	0.118231	0.340206
100.500000	0.197893	0.267746	0.206915
100.500000	0.240754	0.394542	0.167624
100.500000	0.281363	0.42826	0.2639
101.000000	0.180212	0.450281	0.143651
101.000000	0.116151	0.656081	0.100912
101.000000	0.179284	0.676206	0.230983
102.000000	0.102097	0.669935	0.482911
102.000000	0.0427575	0.93507	0.304761
102.000000	0.0562424	1	0.531177
104.000000	0.0632155	0.488317	0.570103
104.000000	0.211053	0.709617	0.640054
104.000000	0.333295	0.721789	1
106.000000	0.136904	0.390879	0.149793
106.000000	0.148629	0.562657	0.192597
106.000000	0.257559	0.604689	0.396533
124.000000	0.833681	0.135585	0.0822378
124.000000	0.9674	0.207241	0.196406
124.000000	1	0.253548	0.302234
148.000000	0.26162	0.0153155	0.190012
148.000000	0.425067	0.0130094	0.327301
148.000000	0.57261	0.0165319	0.565653

Table 5: Experimental data for the experiment Pifo Rescue Clone 4

1.5 Experiment: Pifo Rescue Clone 4

The model outputs available in this data set are defined by:

$$\text{gli1\_gli1A\_obs} = \text{scale\_gli1\_clone07} \cdot ([\text{gli1}] + [\text{gli1A}]) \tag{118}$$

$$\text{gli2\_gli2A\_obs} = \text{scale\_gli2\_clone07} \cdot ([\text{gli2}] + [\text{gli2A}]) \tag{119}$$

$$\text{venus\_pifo\_obs} = [\text{pifo\_v}] \cdot \text{scale\_venus\_pifo07} \tag{120}$$

The error model that describes the measurement noise for each model output is given by:

$$\text{gli1\_gli1A\_obs} = \text{sd\_gli1\_gli1A\_clone} \tag{121}$$

$$\text{gli2\_gli2A\_obs} = \text{sd\_gli2\_gli2A\_clone} \tag{122}$$

$$\text{venus\_pifo\_obs} = \text{sd\_venus\_pifo} \tag{123}$$

To evaluate the ODE system of Equation 167 – 178 for the conditions in this experiment, the following external inputs are given:

$$[\text{SHH}](t) = -\text{SHH\_level} \cdot \left(\frac{1}{e^{100000000.0 \cdot t - 10000000000.0} + 1} - 1 \right) \tag{124}$$

$$a\_pifo2(t) = \frac{a\_wt2}{e^{100000000.0 \cdot t - 5000000000.0} + 1} \tag{125}$$

To evaluate the ODE system of Equation 167 – 190 for the conditions in this experiment, the following parameter transformations are applied:

$$a\_v2 \rightarrow a\_v72$$
$$\text{init\_pifo\_v} \rightarrow 0$$

The agreement of the model outputs and the experimental data, given in Table 20, yields a value of the objective function $-2\log(L) = -50.5278$ for 72 data points in this data set.

time [hour]	gli1_gli1A_obs conc. [au]	gli2_gli2A_obs conc. [au]	venus_pifo_obs conc. [au]
100.000000	0.0739123	0.1948	0.198494
100.000000	0.0636712	0.31717	0.179004
100.000000	0.0657867	0.323546	0.372559
100.500000	0.1244	0.506959	0.393045
100.500000	0.138967	0.731195	0.447628
100.500000	0.239535	0.762084	0.754138
101.000000	0.783126	0.563019	0.4447
101.000000	0.854814	0.799054	0.600356
101.000000	1	0.823444	0.933172
102.000000	0.639209	0.478818	0.249208
102.000000	0.602416	0.694089	0.234393
102.000000	0.751857	0.729338	0.456093
104.000000	0.429773	0.571087	0.293654
104.000000	0.535049	0.790858	0.342568
104.000000	0.488277	0.870161	0.730114
106.000000	0.0606591	0.639728	0.532743
106.000000	0.076192	0.920131	0.476698
106.000000	0.0680909	1	0.829352
124.000000	0.576613	0.420892	0.254142
124.000000	0.679178	0.573901	0.242631
124.000000	0.666618	0.659444	0.525376
148.000000	0.605284	0.0186576	0.425407
148.000000	0.700978	0.0108751	0.313511
148.000000	0.676982	0.023538	1

Table 6: Experimental data for the experiment Pifo Rescue Clone 5

1.6 Experiment: Pifo Rescue Clone 5

The model outputs available in this data set are defined by:

$$\text{gli1\_gli1A\_obs} = \text{scale\_gli1\_clone08} \cdot ([\text{gli1}] + [\text{gli1A}]) \quad (126)$$

$$\text{gli2\_gli2A\_obs} = \text{scale\_gli2\_clone08} \cdot ([\text{gli2}] + [\text{gli2A}]) \quad (127)$$

$$\text{venus\_pifo\_obs} = [\text{pifo\_v}] \cdot \text{scale\_venus\_pifo08} \quad (128)$$

The error model that describes the measurement noise for each model output is given by:

$$\text{gli1\_gli1A\_obs} = \text{sd\_gli1\_gli1A\_clone} \quad (129)$$

$$\text{gli2\_gli2A\_obs} = \text{sd\_gli2\_gli2A\_clone} \quad (130)$$

$$\text{venus\_pifo\_obs} = \text{sd\_venus\_pifo} \quad (131)$$

To evaluate the ODE system of Equation 167 – 178 for the conditions in this experiment, the following external inputs are given:

$$[\text{SHH}](t) = -\text{SHH\_level} \cdot \left(\frac{1}{e^{100000000.0 \cdot t - 10000000000.0} + 1} - 1 \right) \quad (132)$$

$$a\_pifo2(t) = \frac{a\_wt2}{e^{100000000.0 \cdot t - 5000000000.0} + 1} \quad (133)$$

To evaluate the ODE system of Equation 167 – 190 for the conditions in this experiment, the following parameter transformations are applied:

$$a\_v2 \rightarrow a\_v82$$
$$\text{init\_pifo\_v} \rightarrow 0$$

The agreement of the model outputs and the experimental data, given in Table 21, yields a value of the objective function $-2\log(L) = 20.3828$ for 72 data points in this data set.

time [hour]	gli1_gli1A_obs conc. [au]	gli2_gli2A_obs conc. [au]
120.000000	0.0148293	0.14167
120.000000	0.255391	0.422122
120.000000	0.2119	0.536753
120.500000	0.140849	0.121386
120.500000	0.217946	0.639094
120.500000	0.24315	0.518478
121.000000	0.237411	0.274256
121.000000	0.337304	0.804296
121.000000	0.464681	0.994742
122.000000	0.292327	0.217122
122.000000	0.462761	0.838048
122.000000	0.630735	1
123.000000	NaN	NaN
123.000000	NaN	NaN
123.000000	NaN	NaN
124.000000	0.202883	0.118393
124.000000	0.536092	0.556568
124.000000	0.787388	0.760585
126.000000	0.135418	0.148018
126.000000	0.363123	0.236529
126.000000	0.528869	0.496442
144.000000	0.0974319	0.173303
144.000000	0.530863	0.159731
144.000000	0.491572	0.83185
168.000000	0.320396	0.0582678
168.000000	1	0.456963
168.000000	0.802135	0.14838

Table 7: Experimental data for the experiment Pifo$^{FD/FD}$

1.7 Experiment: Pifo$^{FD/FD}$

The model outputs available in this data set are defined by:

$$\text{gli1\_gli1A\_obs} = \text{scale\_gli1\_}ko \cdot ([\text{gli1}] + [\text{gli1A}]) \tag{134}$$

$$\text{gli2\_gli2A\_obs} = \text{scale\_gli2\_}ko \cdot ([\text{gli2}] + [\text{gli2A}]) \tag{135}$$

The error model that describes the measurement noise for each model output is given by:

$$\text{gli1\_gli1A\_obs} = \text{sd\_gli1\_gli1A\_}ko \tag{136}$$

$$\text{gli2\_gli2A\_obs} = \text{sd\_gli2\_gli2A\_}ko \tag{137}$$

To evaluate the ODE system of Equation 167 − 178 for the conditions in this experiment, the following external inputs are given:

$$[\text{SHH}](t) = -\text{SHH\_level} \cdot \left(\frac{1}{e^{100000000.0 \cdot t - 12000000000.0} + 1} - 1 \right) \tag{138}$$

$$a\_pifo2(t) = \frac{a\_wt2}{e^{100000000.0 \cdot t - 500000000.0} + 1} \tag{139}$$

To evaluate the ODE system of Equation 167 − 190 for the conditions in this experiment, the following parameter transformations are applied:

$$a\_v2 \rightarrow 0$$
$$\text{init\_pifo\_v} \rightarrow 0$$

The agreement of the model outputs and the experimental data, given in Table 22, yields a value of the objective function $-2\log(L) = 23.2751$ for 48 data points in this data set.

	name	θ_{min}	$\hat{\theta}$	θ_{max}	log	non-log $\hat{\theta}$	fitted
1	SHH_b	-7	-6.7627	-1	1	$+1.73 \cdot 10^{-07}$	1
2	SHH_level	-5	+1.4868	+2	1	$+3.07 \cdot 10^{+01}$	1
3	a_v32	-5	+2.5541	+3	1	$+3.58 \cdot 10^{+02}$	1
4	a_v42	-5	+3.0000	+3	1	$+1.00 \cdot 10^{+03}$	1
5	a_v62	-5	+1.6499	+2	1	$+4.47 \cdot 10^{+01}$	1
6	a_v72	-5	+1.9641	+3	1	$+9.21 \cdot 10^{+01}$	1
7	a_v82	-5	-2.8431	+3	1	$+1.44 \cdot 10^{-03}$	1
8	a_wt2	-3	+0.0108	+1	1	$+1.03 \cdot 10^{+00}$	1
9	b1	-5	+1.0862	+2	1	$+1.22 \cdot 10^{+01}$	1
10	b10	-5	-5.0000	+1	1	$+1.00 \cdot 10^{-05}$	1
11	b11	-5	-0.4131	+2	1	$+3.86 \cdot 10^{-01}$	1
12	b12	-5	+1.7802	+2	1	$+6.03 \cdot 10^{+01}$	1
13	b3	-5	-5.0000	-1	1	$+1.00 \cdot 10^{-05}$	1
14	b5	-5	+0.3941	+3	1	$+2.48 \cdot 10^{+00}$	1
15	d1	-5	+3.0000	+3	1	$+1.00 \cdot 10^{+03}$	1
16	g1	-5	-1.0671	+1	1	$+8.57 \cdot 10^{-02}$	1
17	g10	-5	+1.5000	+2	1	$+3.16 \cdot 10^{+01}$	1
18	g2	-5	+1.2904	+3	1	$+1.95 \cdot 10^{+01}$	1
19	g3	-5	+1.0000	+1	1	$+1.00 \cdot 10^{+01}$	1
20	g4	-5	-4.9977	+1	1	$+1.01 \cdot 10^{-05}$	1
21	g5	-5	-2.3566	+1	1	$+4.40 \cdot 10^{-03}$	1
22	g6	-5	-1.4623	+3	1	$+3.45 \cdot 10^{-02}$	1
23	g7	-5	-1.7288	+1	1	$+1.87 \cdot 10^{-02}$	1
24	g9	-5	+3.0000	+3	1	$+1.00 \cdot 10^{+03}$	1

Table 8: Estimated parameter values
$\hat{\theta}$ indicates the estimated value of the parameters. θ_{min} and θ_{max} indicate the upper and lower bounds for the parameters. The log-column indicates if the value of a parameter was log-transformed. If log = 1 the non-log-column indicates the non-logarithmic value of the estimate. The fitted-column indicates if the parameter value was estimated (1), was temporarily fixed (0) or if its value was fixed to a constant value (2).

2 Estimated model parameters

The model parameter were estimated by maximum likelihood estimation applying the MATLAB lsqnonlin algorithm. In Table 23 – 26 the estimated parameter values are given. Parameters highlighted in red color indicate parameter values close to their bounds. The parameter name prefix init_ indicates the initial value of a dynamic variable. The parameter name prefix offset_ indicates a offset of the experimental data. The parameter name prefix scale_ indicates a scaling factor of the experimental data. The parameter name prefix sd_ indicates the magnitude of the measurement noise for a specific measurement.

	name	θ_{min}	$\hat{\theta}$	θ_{max}	log	non-log $\hat{\theta}$	fitted
25	init_gli1	-5	-1.3842	+1	1	$+4.13 \cdot 10^{-02}$	1
26	init_gli1A	-5	-4.5329	+1	1	$+2.93 \cdot 10^{-05}$	1
27	init_gli2	-5	+1.9988	+2	1	$+9.97 \cdot 10^{+01}$	1
28	init_gli2A	-1	+0.1395	+1	1	$+1.38 \cdot 10^{+00}$	1
29	init_gli3	-5	-3.4830	+1	1	$+3.29 \cdot 10^{-04}$	1
30	init_gli3R	-5	+0.1999	+1	1	$+1.58 \cdot 10^{+00}$	1
31	init_pifo_wt	-5	-0.3153	+1	1	$+4.84 \cdot 10^{-01}$	1
32	init_ptc	-5	+2.5000	+2	1	$+3.16 \cdot 10^{+02}$	1
33	init_ptc_mRNA	-5	-0.6338	+1	1	$+2.32 \cdot 10^{-01}$	1
34	init_shhPtch	-5	-2.7268	+0.5	1	$+1.88 \cdot 10^{-03}$	1
35	init_smo	-5	-1.6876	+0	1	$+2.05 \cdot 10^{-02}$	1
36	k1	-5	-1.5015	+1	1	$+3.15 \cdot 10^{-02}$	1
37	q0	-5	-2.3004	+1	1	$+5.01 \cdot 10^{-03}$	1
38	q1	-5	-4.7372	+1	1	$+1.83 \cdot 10^{-05}$	1
39	q2	-5	-0.7431	+1	1	$+1.81 \cdot 10^{-01}$	1
40	r0	-5	+0.8332	+2	1	$+6.81 \cdot 10^{+00}$	1
41	r1	-5	-4.5526	+1	1	$+2.80 \cdot 10^{-05}$	1
42	r2	-5	-1.1795	+1	1	$+6.62 \cdot 10^{-02}$	1
43	r3	-5	-0.5014	+1	1	$+3.15 \cdot 10^{-01}$	1
44	r4	-5	-1.7186	+1	1	$+1.91 \cdot 10^{-02}$	1
45	scale_dummy	-5	-2.3758	+1	1	$+4.21 \cdot 10^{-03}$	1
46	scale_gli11	-5	-2.0871	+1	1	$+8.18 \cdot 10^{-03}$	1
47	scale_gli12	-5	-2.1646	+1	1	$+6.85 \cdot 10^{-03}$	1
48	scale_gli13	-5	-2.0719	+1	1	$+8.47 \cdot 10^{-03}$	1
49	scale_gli1_clone03	-5	-1.8273	+1	1	$+1.49 \cdot 10^{-02}$	1

Table 9: Estimated parameter values
$\hat{\theta}$ indicates the estimated value of the parameters. θ_{min} and θ_{max} indicate the upper and lower bounds for the parameters. The log-column indicates if the value of a parameter was log-transformed. If log = 1 the non-log-column indicates the non-logarithmic value of the estimate. The fitted-column indicates if the parameter value was estimated (1), was temporarily fixed (0) or if its value was fixed to a constant value (2).

	name	θ_{min}	$\hat{\theta}$	θ_{max}	log	non-log $\hat{\theta}$	fitted
50	scale_gli1_clone04	-5	-2.4084	+1	1	$+3.90 \cdot 10^{-03}$	1
51	scale_gli1_clone06	-5	-2.2559	+1	1	$+5.55 \cdot 10^{-03}$	1
52	scale_gli1_clone07	-5	-2.1079	+1	1	$+7.80 \cdot 10^{-03}$	1
53	scale_gli1_clone08	-5	-1.1230	+1	1	$+7.53 \cdot 10^{-02}$	1
54	scale_gli1_ko	-5	-1.2413	+3	1	$+5.74 \cdot 10^{-02}$	1
55	scale_gli21	-5	-2.5048	+1	1	$+3.13 \cdot 10^{-03}$	1
56	scale_gli22	-5	-2.5260	+1	1	$+2.98 \cdot 10^{-03}$	1
57	scale_gli23	-5	-2.5278	+1	1	$+2.97 \cdot 10^{-03}$	1
58	scale_gli2_clone03	-5	-2.4102	+1	1	$+3.89 \cdot 10^{-03}$	1
59	scale_gli2_clone04	-5	-2.4541	+1	1	$+3.51 \cdot 10^{-03}$	1
60	scale_gli2_clone06	-5	-2.6346	+1	1	$+2.32 \cdot 10^{-03}$	1
61	scale_gli2_clone07	-5	-2.5273	+1	1	$+2.97 \cdot 10^{-03}$	1
62	scale_gli2_clone08	-5	-2.5546	+1	1	$+2.79 \cdot 10^{-03}$	1
63	scale_gli2_ko	-5	-2.6841	+1	1	$+2.07 \cdot 10^{-03}$	1
64	scale_gli3	-5	-1.2844	+1	1	$+5.20 \cdot 10^{-02}$	1
65	scale_gli31	-5	-1.3495	+1	1	$+4.47 \cdot 10^{-02}$	1
66	scale_gli3R1	-5	-1.3155	+1	1	$+4.84 \cdot 10^{-02}$	1
67	scale_mRNA	-5	-1.2758	+2	1	$+5.30 \cdot 10^{-02}$	1
68	scale_pifo1	-5	-1.6918	+2	1	$+2.03 \cdot 10^{-02}$	1
69	scale_pifo2	-5	-1.8584	+1	1	$+1.39 \cdot 10^{-02}$	1
70	scale_venus_pifo03	-5	-4.1812	+1	1	$+6.59 \cdot 10^{-05}$	1
71	scale_venus_pifo04	-5	-4.6137	+1	1	$+2.43 \cdot 10^{-05}$	1
72	scale_venus_pifo06	-5	-3.5914	+1	1	$+2.56 \cdot 10^{-04}$	1
73	scale_venus_pifo07	-5	-3.8173	+1	1	$+1.52 \cdot 10^{-04}$	1
74	scale_venus_pifo08	-5	+1.0000	+1	1	$+1.00 \cdot 10^{+01}$	1

Table 10: Estimated parameter values
$\hat{\theta}$ indicates the estimated value of the parameters. θ_{min} and θ_{max} indicate the upper and lower bounds for the parameters. The log-column indicates if the value of a parameter was log-transformed. If log = 1 the non-log-column indicates the non-logarithmic value of the estimate. The fitted-column indicates if the parameter value was estimated (1), was temporarily fixed (0) or if its value was fixed to a constant value (2).

	name	θ_{min}	$\hat{\theta}$	θ_{max}	log	non-log $\hat{\theta}$	fitted
75	sd_gli1_gli1A	-5	-1.1655	+1	1	$+6.83 \cdot 10^{-02}$	1
76	sd_gli1_gli1A_clone	-5	-1.0030	+1	1	$+9.93 \cdot 10^{-02}$	1
77	sd_gli1_gli1A_ko	-5	-0.6029	+1	1	$+2.50 \cdot 10^{-01}$	1
78	sd_gli2_gli2A	-5	-0.8711	+1	1	$+1.35 \cdot 10^{-01}$	1
79	sd_gli2_gli2A_clone	-5	-0.6791	+1	1	$+2.09 \cdot 10^{-01}$	1
80	sd_gli2_gli2A_ko	-5	-0.4468	+1	1	$+3.57 \cdot 10^{-01}$	1
81	sd_gli3	-5	-1.0464	+1	1	$+8.99 \cdot 10^{-02}$	1
82	sd_gli3R	-5	-0.3119	+1	1	$+4.88 \cdot 10^{-01}$	1
83	sd_mRNA	-5	-1.4361	+1	1	$+3.66 \cdot 10^{-02}$	1
84	sd_pifo	-5	-0.8867	+1	1	$+1.30 \cdot 10^{-01}$	1
85	sd_venus_pifo	-5	-0.6077	+1	1	$+2.47 \cdot 10^{-01}$	1
86	t1	-5	-5.0000	+1	1	$+1.00 \cdot 10^{-05}$	1
87	t2	-5	-1.1095	+1	1	$+7.77 \cdot 10^{-02}$	1
88	t3	-5	+2.7643	+3	1	$+5.81 \cdot 10^{+02}$	1
89	t4	-5	+0.2389	+3	1	$+1.73 \cdot 10^{+00}$	1
90	t5	-5	+0.7011	+1	1	$+5.02 \cdot 10^{+00}$	1
91	t6	-5	-2.4523	+1	1	$+3.53 \cdot 10^{-03}$	1

Table 11: Estimated parameter values
$\hat{\theta}$ indicates the estimated value of the parameters. θ_{min} and θ_{max} indicate the upper and lower bounds for the parameters. The log-column indicates if the value of a parameter was log-transformed. If log = 1 the non-log-column indicates the non-logarithmic value of the estimate. The fitted-column indicates if the parameter value was estimated (1), was temporarily fixed (0) or if its value was fixed to a constant value (2).

3 Profile likelihood of model parameters

In order to evaluate the identifiability of the model parameters and to assess confidence intervals the profile likelihood [2] was calculated. The mean calculation time of the profile likelihood per parameter was 00:05:06.74 ± 00:04:57.40. An overview is displayed in Figure 11 - 18.

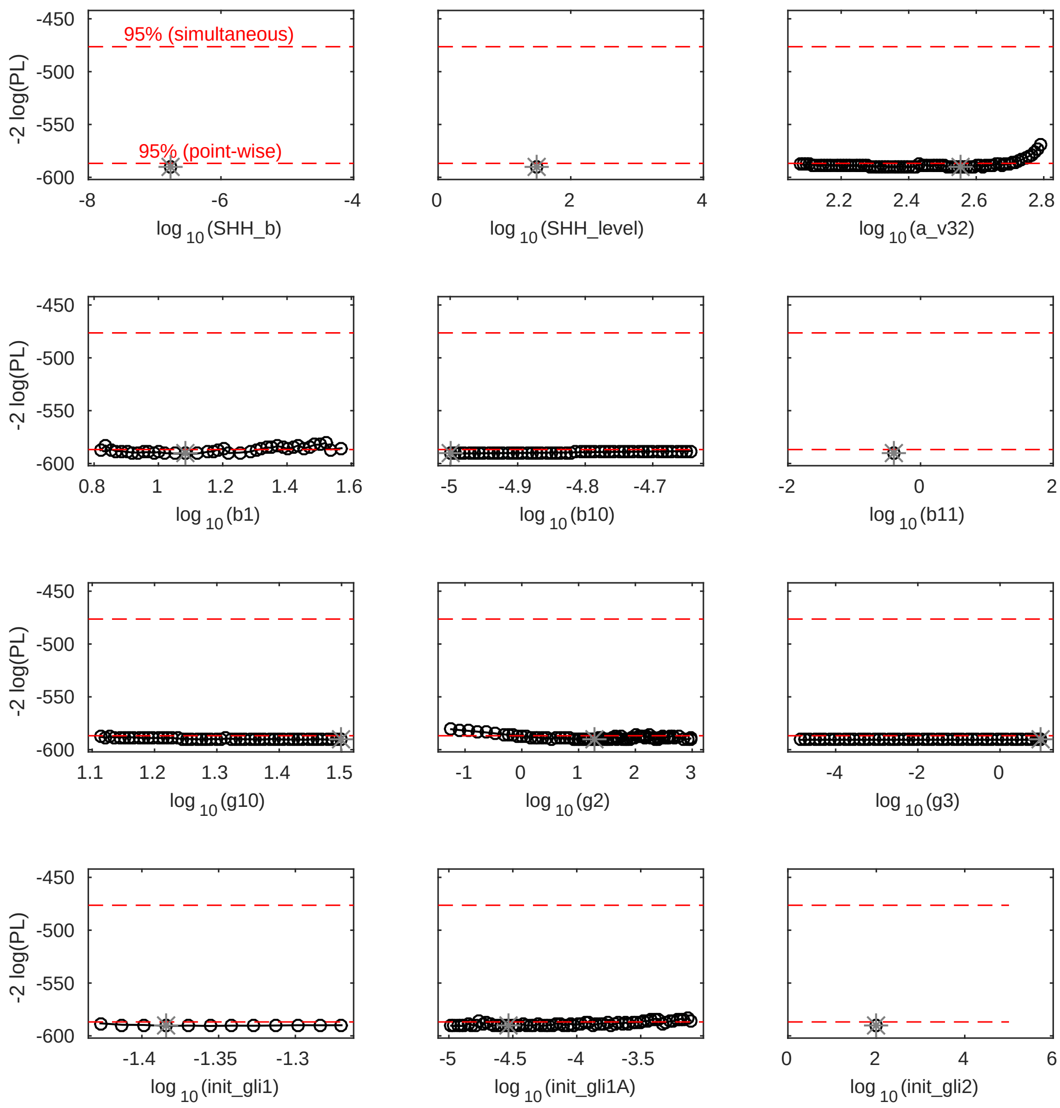

Figure 2: Overview of the profile likelihood of the model parameters
The solide lines indicate the profile likelihood. The broken lines indicate the threshold to assess confidence intervals. The asterisk indicate the optimal parameter values.

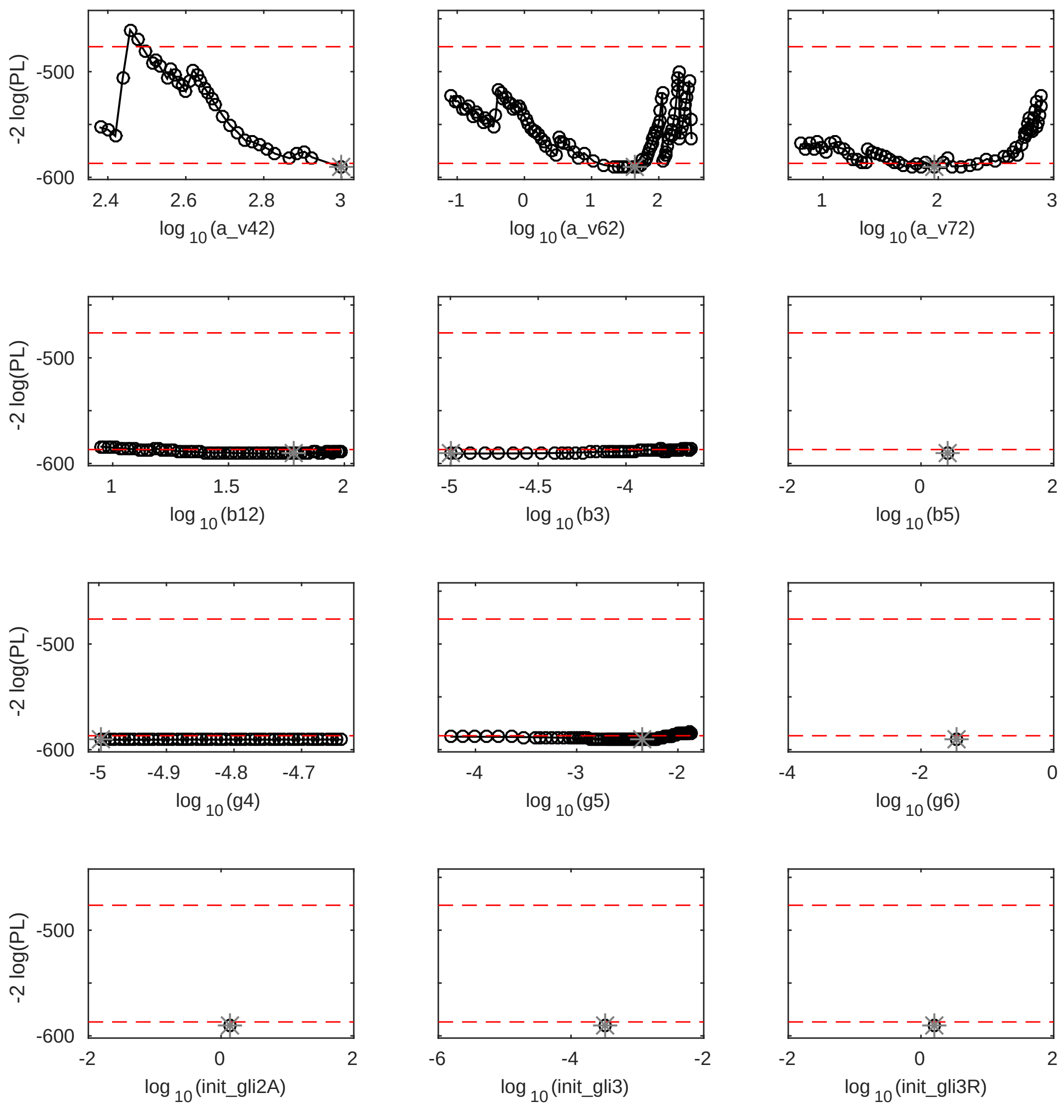

Figure 3: Overview of the profile likelihood of the model parameters
The solide lines indicate the profile likelihood. The broken lines indicate the threshold to assess confidence intervals. The asterisk indicate the optimal parameter values.

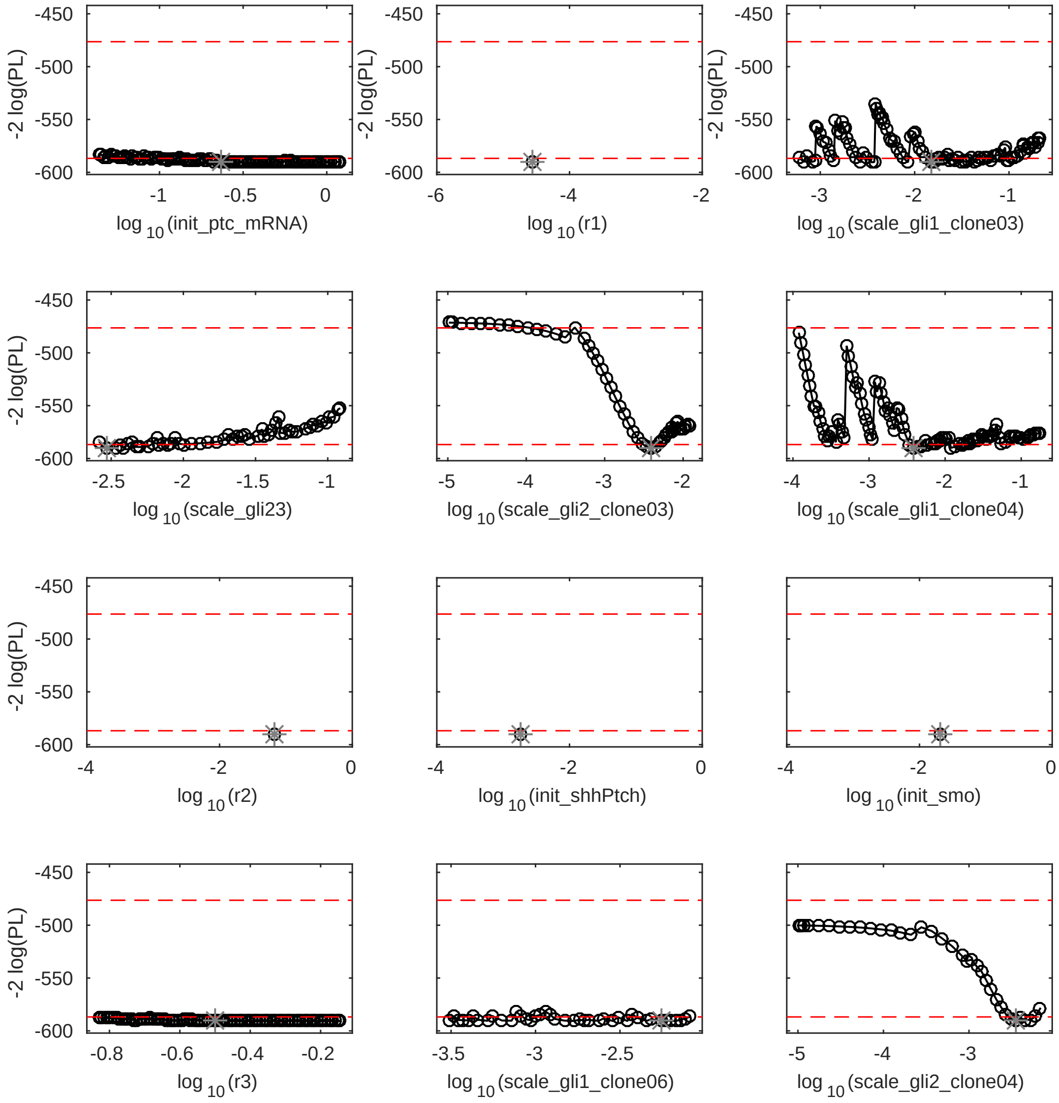

Figure 4: Overview of the profile likelihood of the model parameters
The solide lines indicate the profile likelihood. The broken lines indicate the threshold to assess confidence intervals. The asterisk indicate the optimal parameter values.

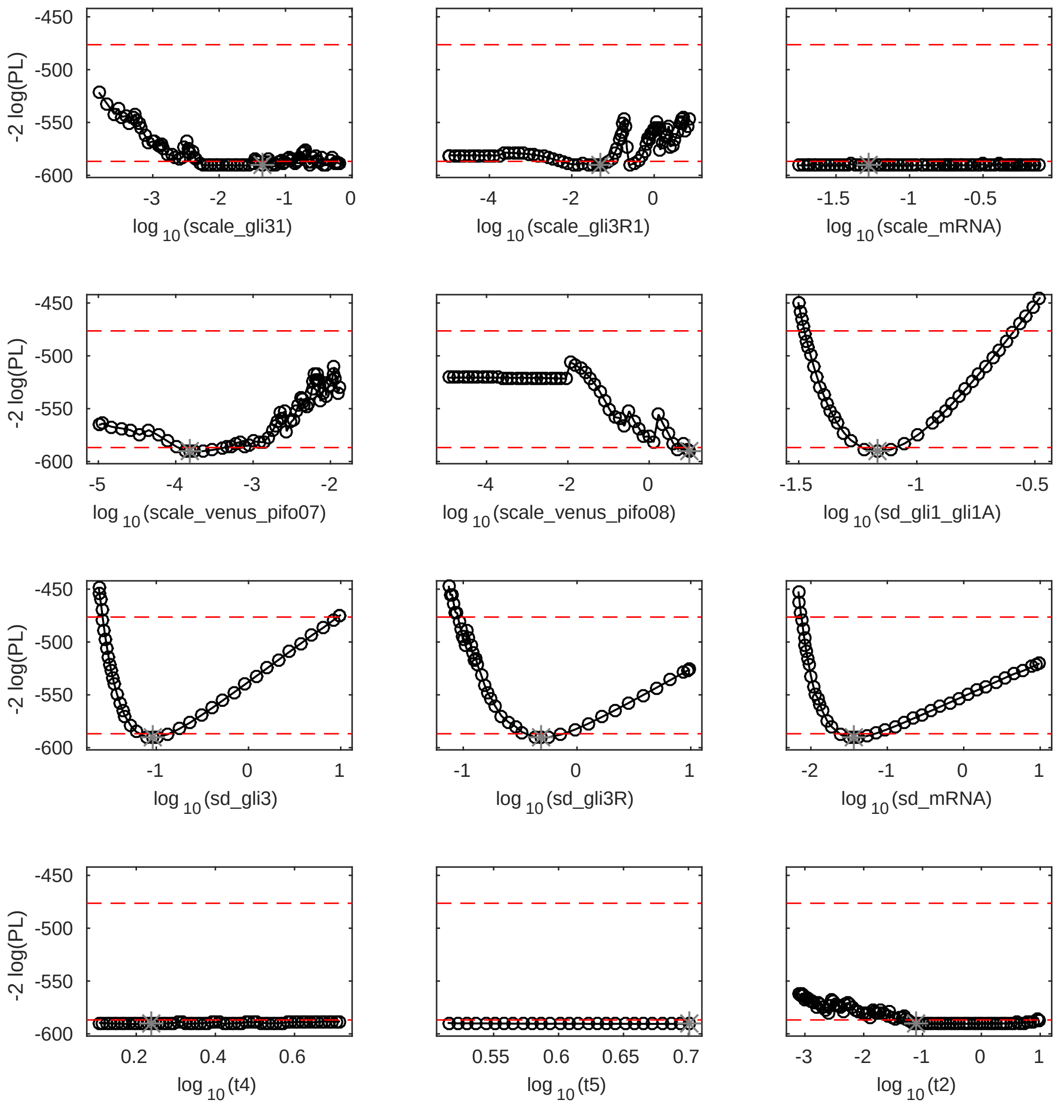

Figure 5: Overview of the profile likelihood of the model parameters
The solide lines indicate the profile likelihood. The broken lines indicate the threshold to assess confidence intervals. The asterisk indicate the optimal parameter values.

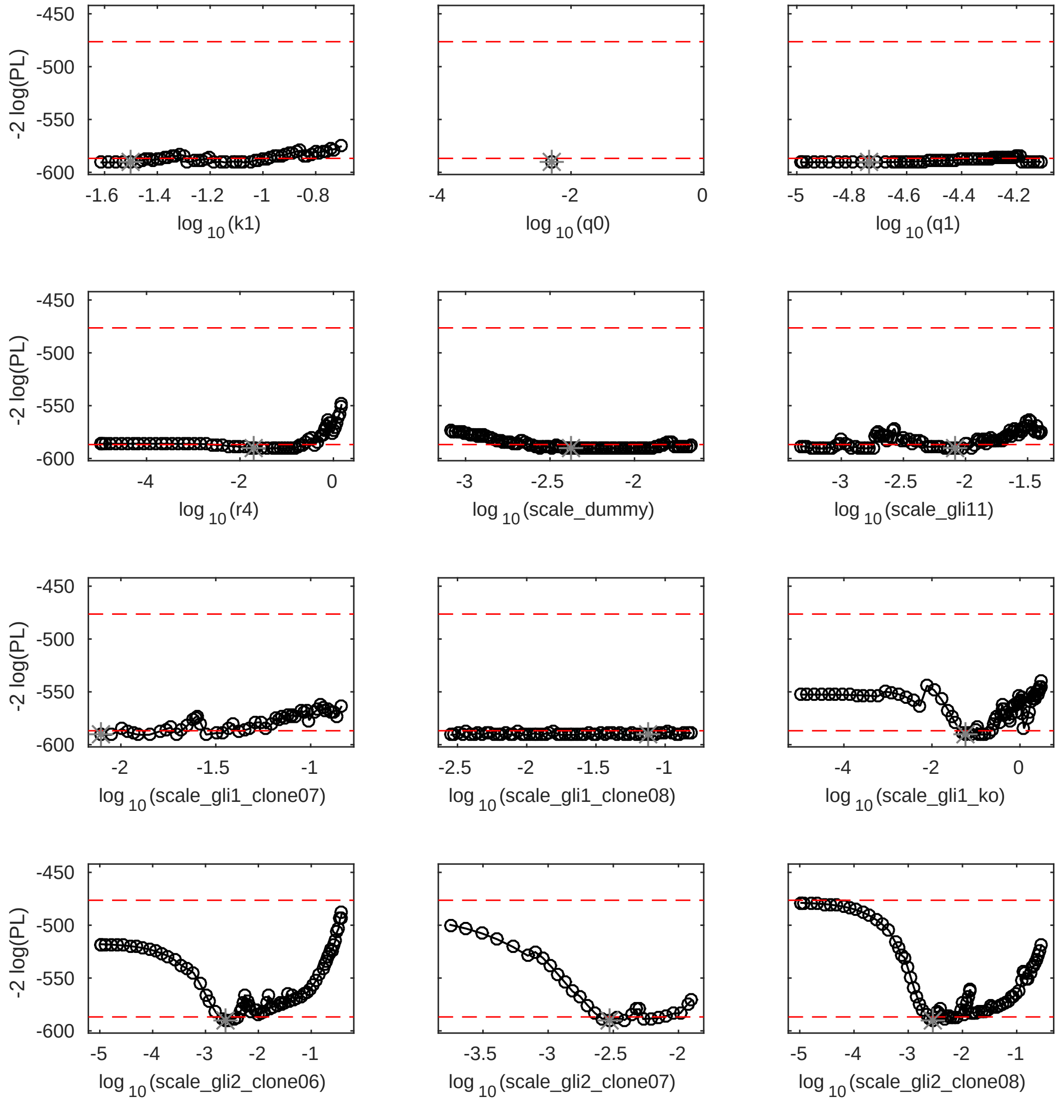

Figure 6: Overview of the profile likelihood of the model parameters
The solide lines indicate the profile likelihood. The broken lines indicate the threshold to assess confidence intervals. The asterisk indicate the optimal parameter values.

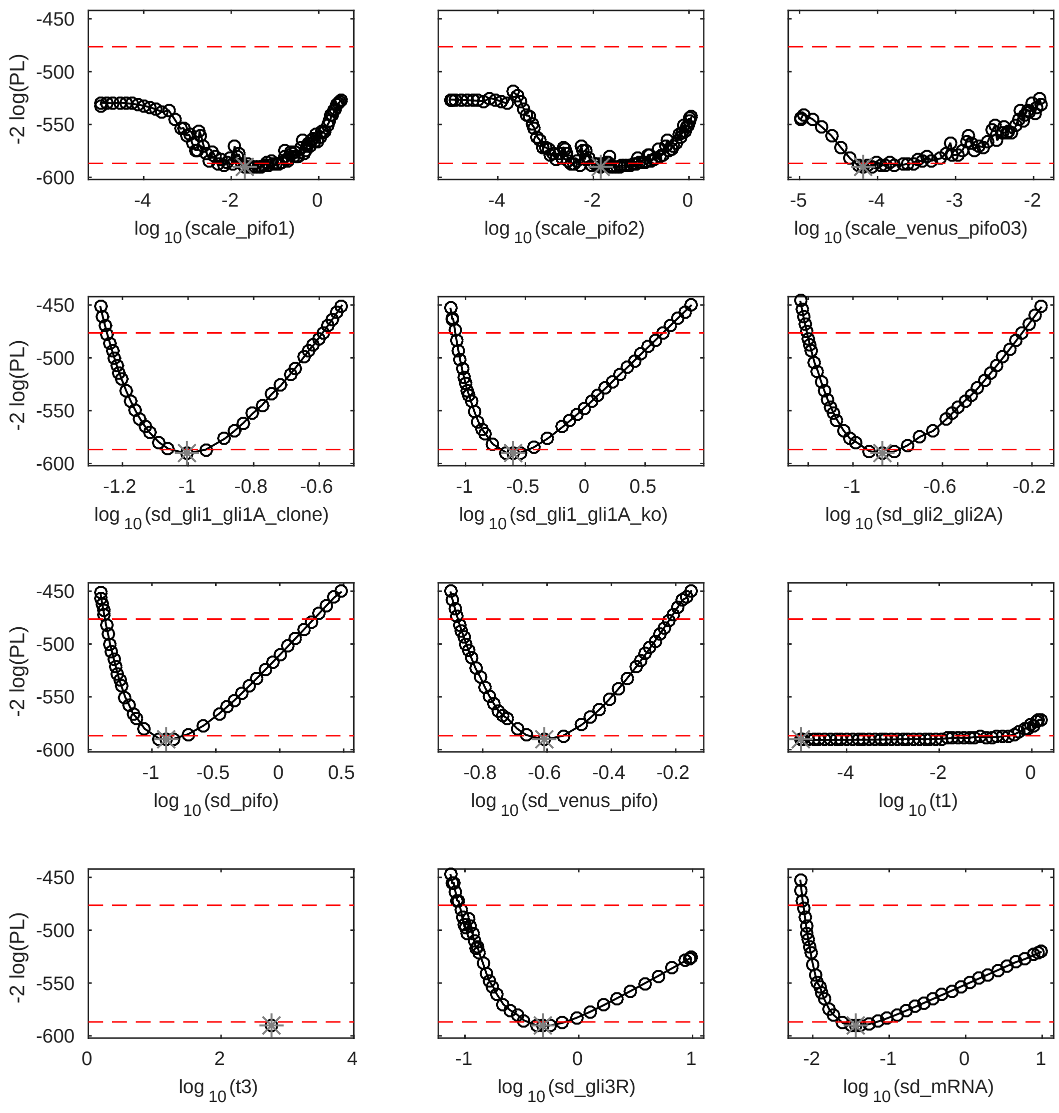

Figure 7: Overview of the profile likelihood of the model parameters
The solide lines indicate the profile likelihood. The broken lines indicate the threshold to assess confidence intervals. The asterisk indicate the optimal parameter values.

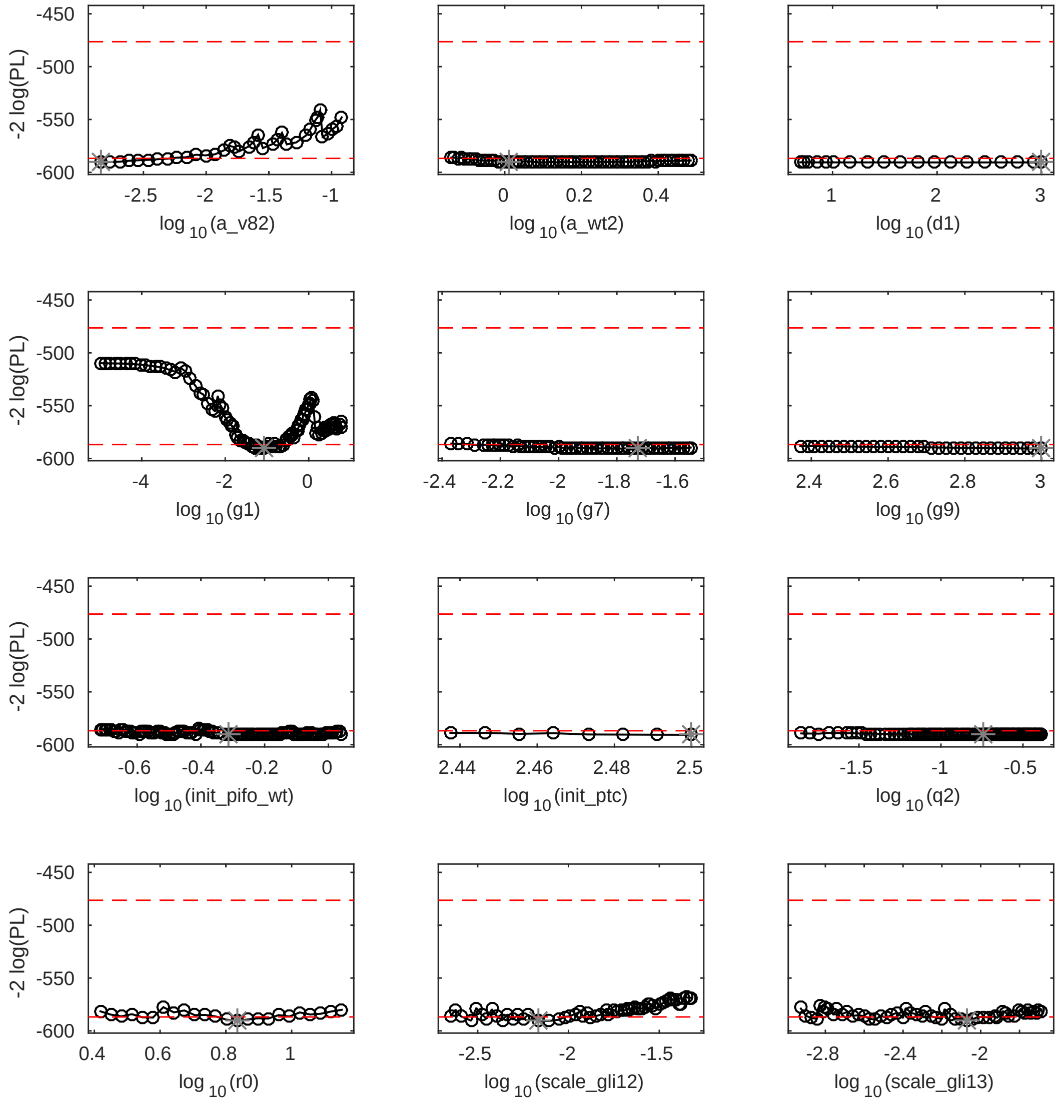

Figure 8: Overview of the profile likelihood of the model parameters
The solide lines indicate the profile likelihood. The broken lines indicate the threshold to assess confidence intervals. The asterisk indicate the optimal parameter values.

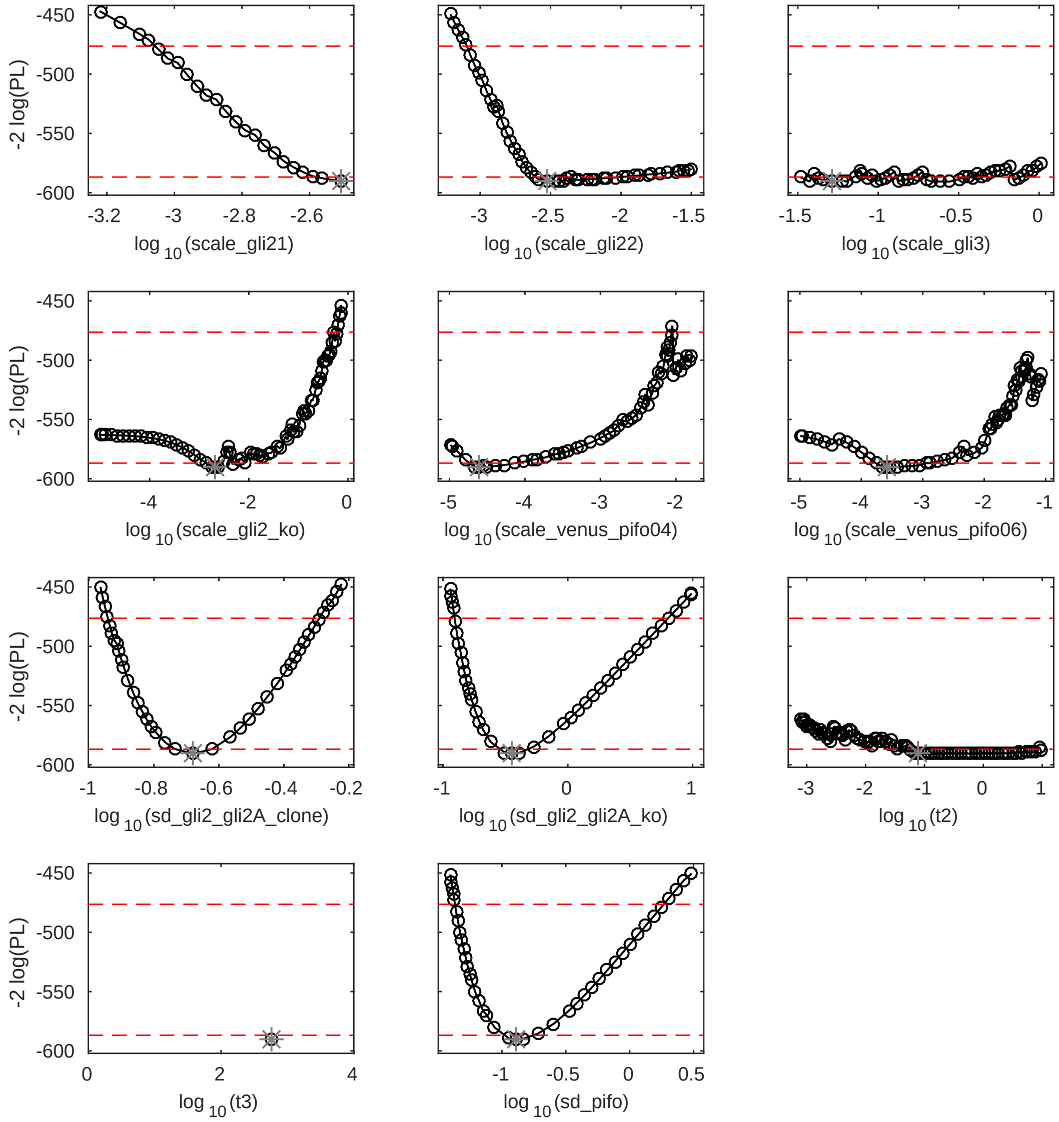

Figure 9: Overview of the profile likelihood of the model parameters
The solide lines indicate the profile likelihood. The broken lines indicate the threshold to assess confidence intervals. The asterisk indicate the optimal parameter values.

	name	$\hat{\theta}$	σ^-_{ptw}	σ^+_{ptw}	σ^-_{sim}	σ^+_{sim}
1	SHH_b	-6.763	NaN	NaN	NaN	NaN
2	SHH_level	+1.487	NaN	NaN	NaN	NaN
3	a_v32	+2.554	-Inf	+2.704	-Inf	+Inf
4	a_v42	+3.000	+2.969	+Inf	-Inf	+Inf
5	a_v62	+1.650	+1.113	+1.734	-Inf	+Inf
6	a_v72	+1.964	+1.668	+2.357	-Inf	+Inf
7	a_v82	-2.843	-Inf	-2.286	-Inf	+Inf
8	a_wt2	+0.011	-0.123	+Inf	-Inf	+Inf
9	b1	+1.086	-Inf	+1.313	-Inf	+Inf
10	b10	-5.000	-Inf	+Inf	-Inf	+Inf
11	b11	-0.413	NaN	NaN	NaN	NaN
12	b12	+1.780	+1.116	+Inf	-Inf	+Inf
13	b3	-5.000	-Inf	-3.638	-Inf	+Inf
14	b5	+0.394	NaN	NaN	NaN	NaN
15	d1	+3.000	-Inf	+Inf	-Inf	+Inf
16	g1	-1.067	-1.385	-0.614	-Inf	+Inf
17	g10	+1.500	-Inf	+Inf	-Inf	+Inf
18	g2	+1.290	-0.077	+Inf	-Inf	+Inf
19	g3	+1.000	-Inf	+Inf	-Inf	+Inf
20	g4	-4.998	-Inf	+Inf	-Inf	+Inf
21	g5	-2.357	-Inf	-2.056	-Inf	+Inf
22	g6	-1.462	NaN	NaN	NaN	NaN
23	g7	-1.729	-2.295	+Inf	-Inf	+Inf
24	g9	+3.000	-Inf	+Inf	-Inf	+Inf
25	init_gli1	-1.384	-Inf	+Inf	-Inf	+Inf
26	init_gli1A	-4.533	-Inf	-3.291	-Inf	+Inf
27	init_gli2	+1.999	NaN	NaN	NaN	NaN
28	init_gli2A	+0.139	NaN	NaN	NaN	NaN
29	init_gli3	-3.483	NaN	NaN	NaN	NaN

Table 12: Confidence intervals for the estimated parameter values derived by the profile likelihood $\hat{\theta}$ indicates the estimated optimal parameter value. σ^-_{ptw} and σ^+_{ptw} indicate 95% point-wise confidence intervals. σ^-_{sim} and σ^+_{sim} indicate 95% simultaneous confidence intervals.

4 Confidence intervals for the model parameters

In Table 27 – 30, 95% confidence intervals for the estimated parameter values derived by the profile likelihood [2] are given.

	name	$\hat{\theta}$	σ^-_{ptw}	σ^+_{ptw}	σ^-_{sim}	σ^+_{sim}
30	init_gli3R	+0.200	NaN	NaN	NaN	NaN
31	init_pifo_wt	-0.315	-0.677	+Inf	-Inf	+Inf
32	init_ptc	+2.500	-Inf	+Inf	-Inf	+Inf
33	init_ptc_mRNA	-0.634	-1.182	+Inf	-Inf	+Inf
34	init_shhPtch	-2.727	NaN	NaN	NaN	NaN
35	init_smo	-1.688	NaN	NaN	NaN	NaN
36	k1	-1.501	-Inf	-0.986	-Inf	+Inf
37	q0	-2.300	NaN	NaN	NaN	NaN
38	q1	-4.737	-Inf	+Inf	-Inf	+Inf
39	q2	-0.743	-Inf	+Inf	-Inf	+Inf
40	r0	+0.833	+0.543	+0.944	-Inf	+Inf
41	r1	-4.553	NaN	NaN	NaN	NaN
42	r2	-1.179	NaN	NaN	NaN	NaN
43	r3	-0.501	-Inf	+Inf	-Inf	+Inf
44	r4	-1.719	-2.606	-0.402	-Inf	+Inf
45	scale_dummy	-2.376	-2.624	+Inf	-Inf	+Inf
46	scale_gli11	-2.087	-Inf	-1.928	-Inf	+Inf
47	scale_gli12	-2.165	-2.584	-1.866	-Inf	+Inf
48	scale_gli13	-2.072	-2.886	-1.911	-Inf	+Inf
49	scale_gli1_clone03	-1.827	-3.220	-0.976	-Inf	+Inf
50	scale_gli1_clone04	-2.408	-2.466	-1.766	-Inf	+Inf
51	scale_gli1_clone06	-2.256	-Inf	-2.088	-Inf	+Inf
52	scale_gli1_clone07	-2.108	-Inf	-1.360	-Inf	+Inf
53	scale_gli1_clone08	-1.123	-Inf	+Inf	-Inf	+Inf
54	scale_gli1_ko	-1.241	-1.352	-0.658	-Inf	+Inf
55	scale_gli21	-2.505	-2.579	+Inf	-3.057	+Inf
56	scale_gli22	-2.526	-2.607	-2.004	-3.094	+Inf
57	scale_gli23	-2.528	-2.560	-2.103	-Inf	+Inf
58	scale_gli2_clone03	-2.410	-2.505	-2.324	-3.951	+Inf
59	scale_gli2_clone04	-2.454	-2.534	-2.231	-Inf	+Inf

Table 13: Confidence intervals for the estimated parameter values derived by the profile likelihood
$\hat{\theta}$ indicates the estimated optimal parameter value. σ^-_{ptw} and σ^+_{ptw} indicate 95% point-wise confidence intervals. σ^-_{sim} and σ^+_{sim} indicate 95% simultaneous confidence intervals.

	name	$\hat{\theta}$	σ^-_{ptw}	σ^+_{ptw}	σ^-_{sim}	σ^+_{sim}
60	scale_gli2_clone06	-2.635	-2.731	-2.402	-Inf	+Inf
61	scale_gli2_clone07	-2.527	-2.603	-2.122	-Inf	+Inf
62	scale_gli2_clone08	-2.555	-2.646	-2.096	-Inf	+Inf
63	scale_gli2_ko	-2.684	-2.850	-2.306	-Inf	-0.227
64	scale_gli3	-1.284	-Inf	-0.117	-Inf	+Inf
65	scale_gli31	-1.349	-2.314	+Inf	-Inf	+Inf
66	scale_gli3R1	-1.316	-2.265	-0.406	-Inf	+Inf
67	scale_mRNA	-1.276	-Inf	+Inf	-Inf	+Inf
68	scale_pifo1	-1.692	-2.292	-0.877	-Inf	+Inf
69	scale_pifo2	-1.858	-2.474	-1.108	-Inf	+Inf
70	scale_venus_pifo03	-4.181	-4.279	-3.551	-Inf	+Inf
71	scale_venus_pifo04	-4.614	-4.732	-4.130	-Inf	+Inf
72	scale_venus_pifo06	-3.591	-3.765	-2.872	-Inf	+Inf
73	scale_venus_pifo07	-3.817	-3.968	-3.372	-Inf	+Inf
74	scale_venus_pifo08	+1.000	+0.675	+Inf	-Inf	+Inf
75	sd_gli1_gli1A	-1.165	-1.232	-1.091	-1.476	-0.588
76	sd_gli1_gli1A_clone	-1.003	-1.051	-0.946	-1.247	-0.590
77	sd_gli1_gli1A_ko	-0.603	-0.701	-0.477	-1.085	+0.648
78	sd_gli2_gli2A	-0.871	-0.937	-0.796	-1.205	-0.244
79	sd_gli2_gli2A_clone	-0.679	-0.733	-0.622	-0.942	-0.291
80	sd_gli2_gli2A_ko	-0.447	-0.541	-0.320	-0.907	+0.804
81	sd_gli3	-1.046	-1.177	-0.862	-1.582	+0.967
82	sd_gli3R	-0.312	-0.448	-0.120	-1.046	+Inf
83	sd_mRNA	-1.436	-1.623	-1.163	-2.123	+Inf
84	sd_pifo	-0.887	-0.977	-0.754	-1.367	+0.266
85	sd_venus_pifo	-0.608	-0.656	-0.550	-0.877	-0.218
86	t1	-5.000	-Inf	-0.491	-Inf	+Inf
87	t2	-1.110	-1.253	+Inf	-Inf	+Inf
88	t3	+2.764	NaN	NaN	NaN	NaN
89	t4	+0.239	-Inf	+Inf	-Inf	+Inf

Table 14: Confidence intervals for the estimated parameter values derived by the profile likelihood
$\hat{\theta}$ indicates the estimated optimal parameter value. σ^-_{ptw} and σ^+_{ptw} indicate 95% point-wise confidence intervals. σ^-_{sim} and σ^+_{sim} indicate 95% simultaneous confidence intervals.

	name	$\hat{\theta}$	σ^-_{ptw}	σ^+_{ptw}	σ^-_{sim}	σ^+_{sim}
90	t5	+0.701	-Inf	+Inf	-Inf	+Inf
91	t6	-2.452	-Inf	+Inf	-Inf	+Inf

Table 15: Confidence intervals for the estimated parameter values derived by the profile likelihood
$\hat{\theta}$ indicates the estimated optimal parameter value. σ^-_{ptw} and σ^+_{ptw} indicate 95% point-wise confidence intervals. σ^-_{sim} and σ^+_{sim} indicate 95% simultaneous confidence intervals.

5 Model: Pifo-

The model consists of 2 external inputs, 12 dynamical variables indicated by square brackets and 25 reactions and was evaluated for 7 experimental conditions. In total 91 parameters are estimated from the experimental data, yielding a value of the objective function $-2\log(L) = -342.761$ for a total of 613 data points. The estimated parameter values are given in Section 6.

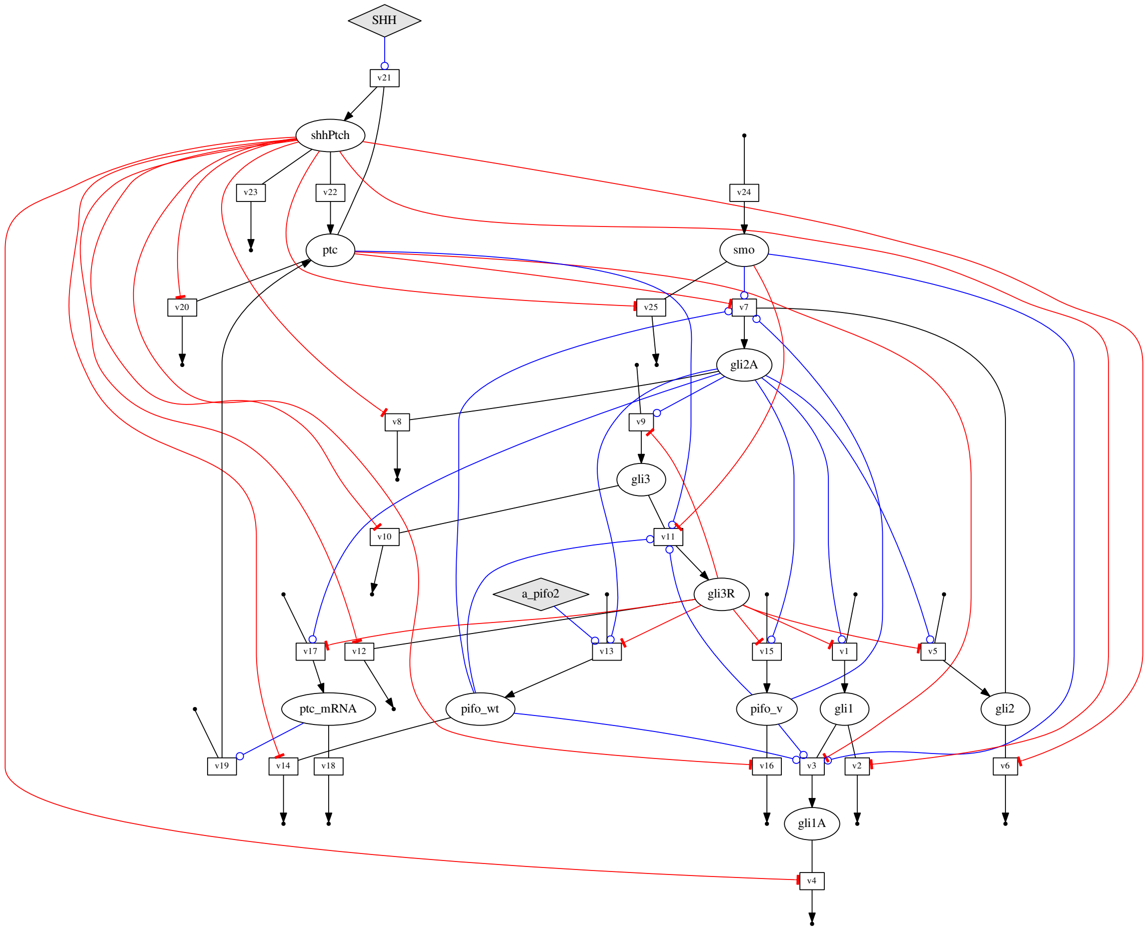

Figure 10: Network representation of the model Pifo-
Diamond shaped nodes correspond to model inputs, see Equation 140 – 141. Ellipsoid shaped nodes correspond to dynamical variables described by the ODE system, see Equation 167 – 178. Black arrows and box shaped nodes indicate reactions with corresponding rate equations given in Equation 142 – 166. Red T-shaped arrows indicated inhibitorial influence on a reaction and blue O-shaped arrows indicate catalysing influence on a reaction.

The model dynamics depends on the external inputs:

$$[\text{SHH}](t) = \text{SHH\_level} \tag{140}$$

$$a\_pifo2(t) = a\_wt2 \tag{141}$$

The rate equations corresponding to the reactions included in the model are give by:

$$v_1 = \frac{\mathrm{a1} \cdot [\mathrm{gli2A}]}{[\mathrm{gli3R}] \cdot \mathrm{r0} + 1} \quad (142)$$

$$v_2 = \frac{\mathrm{b1} \cdot [\mathrm{gli1}]}{\mathrm{g1} \cdot [\mathrm{shhPtch}] + 1} \quad (143)$$

$$v_3 = \frac{[\mathrm{gli1}] \cdot [\mathrm{smo}] \cdot \mathrm{t1} + [\mathrm{gli1}] \cdot [\mathrm{pifo\_v}] \cdot [\mathrm{smo}] \cdot \mathrm{t2} + [\mathrm{gli1}] \cdot \mathrm{pifo\_w}t \cdot [\mathrm{smo}] \cdot \mathrm{t2}}{[\mathrm{ptc}] \cdot \mathrm{q0} + 1} \quad (144)$$

$$v_4 = \frac{\mathrm{b2} \cdot [\mathrm{gli1A}]}{\mathrm{g2} \cdot [\mathrm{shhPtch}] + 1} \quad (145)$$

$$v_5 = \frac{\mathrm{a3} \cdot [\mathrm{gli2A}]}{[\mathrm{gli3R}] \cdot \mathrm{r1} + 1} \quad (146)$$

$$v_6 = \frac{\mathrm{b3} \cdot [\mathrm{gli2}]}{\mathrm{g3} \cdot [\mathrm{shhPtch}] + 1} \quad (147)$$

$$v_7 = \frac{[\mathrm{gli2}] \cdot [\mathrm{smo}] \cdot \mathrm{t3} + [\mathrm{gli2}] \cdot [\mathrm{pifo\_v}] \cdot [\mathrm{smo}] \cdot \mathrm{t4} + [\mathrm{gli2}] \cdot \mathrm{pifo\_w}t \cdot [\mathrm{smo}] \cdot \mathrm{t4}}{[\mathrm{ptc}] \cdot \mathrm{q0} + 1} \quad (148)$$

$$v_8 = \frac{\mathrm{b4} \cdot [\mathrm{gli2A}]}{\mathrm{g4} \cdot [\mathrm{shhPtch}] + 1} \quad (149)$$

$$v_9 = \frac{\mathrm{a5} \cdot [\mathrm{gli2A}]}{[\mathrm{gli3R}] \cdot \mathrm{r2} + 1} \quad (150)$$

$$v_{10} = \frac{\mathrm{b5} \cdot [\mathrm{gli3}]}{\mathrm{g5} \cdot [\mathrm{shhPtch}] + 1} \quad (151)$$

$$v_{11} = \frac{[\mathrm{gli3}] \cdot [\mathrm{ptc}] \cdot \mathrm{t5} + [\mathrm{gli3}] \cdot [\mathrm{pifo\_v}] \cdot [\mathrm{ptc}] \cdot \mathrm{t6} + [\mathrm{gli3}] \cdot \mathrm{pifo\_w}t \cdot [\mathrm{ptc}] \cdot \mathrm{t6}}{[\mathrm{smo}] \cdot (\mathrm{q2} + [\mathrm{pifo\_v}] \cdot \mathrm{q1} + \mathrm{pifo\_w}t \cdot \mathrm{q1}) + 1} \quad (152)$$

$$v_{12} = \frac{\mathrm{b6} \cdot [\mathrm{gli3R}]}{\mathrm{g6} \cdot [\mathrm{shhPtch}] + 1} \quad (153)$$

$$v_{13} = \frac{a\_pifo2 \cdot [\mathrm{gli2A}]}{[\mathrm{gli3R}] \cdot \mathrm{r3} + 1} \quad (154)$$

$$v_{14} = \frac{\mathrm{b7} \cdot \mathrm{pifo\_w}t}{\mathrm{g7} \cdot [\mathrm{shhPtch}] + 1} \quad (155)$$

$$v_{15} = \frac{a\_v2 \cdot [\mathrm{gli2A}]}{[\mathrm{gli3R}] \cdot \mathrm{r3} + 1} \quad (156)$$

$$v_{16} = \frac{\mathrm{b7} \cdot [\mathrm{pifo\_v}]}{\mathrm{g7} \cdot [\mathrm{shhPtch}] + 1} \quad (157)$$

$$v_{17} = \frac{[\mathrm{gli2A}] \cdot \mathrm{k1}}{[\mathrm{gli3R}] \cdot \mathrm{r4} + 1} \quad (158)$$

$$v_{18} = \mathrm{b9} \cdot [\mathrm{ptc\_mRNA}] \quad (159)$$

$$v_{19} = \mathrm{a15} \cdot [\mathrm{ptc\_mRNA}] \quad (160)$$

$$v_{20} = \frac{\mathrm{b10} \cdot [\mathrm{ptc}]}{\mathrm{g9} \cdot [\mathrm{shhPtch}] + 1} \quad (161)$$

$$v_{21} = \mathrm{a19} \cdot [\mathrm{ptc}] \cdot ([\mathrm{SHH}] + \mathrm{SHH\_b}) \quad (162)$$

$$v_{22} = \mathrm{d1} \cdot [\mathrm{shhPtch}] \quad (163)$$

$$v_{23} = \mathrm{b11} \cdot [\mathrm{shhPtch}] \quad (164)$$

$$v_{24} = \mathrm{smo\_base} \quad (165)$$

$$v_{25} = \frac{\mathrm{b12} \cdot [\mathrm{smo}]}{\mathrm{g10} \cdot [\mathrm{shhPtch}] + 1} \quad (166)$$

The ODE system determining the time evolution of the dynamical variables is given by:

$$\mathrm{d[gli1]/d}t = +v_1 - v_2 - v_3 \quad (167)$$
$$\mathrm{d[gli1A]/d}t = +v_3 - v_4 \quad (168)$$
$$\mathrm{d[gli2]/d}t = +v_5 - v_6 - v_7 \quad (169)$$
$$\mathrm{d[gli2A]/d}t = +v_7 - v_8 \quad (170)$$
$$\mathrm{d[gli3]/d}t = +v_9 - v_{10} - v_{11} \quad (171)$$
$$\mathrm{d[gli3R]/d}t = +v_{11} - v_{12} \quad (172)$$
$$\mathrm{dpifo\_w}t\mathrm{/d}t = +v_{13} - v_{14} \quad (173)$$
$$\mathrm{d[pifo\_v]/d}t = +v_{15} - v_{16} \quad (174)$$
$$\mathrm{d[ptc\_mRNA]/d}t = +v_{17} - v_{18} \quad (175)$$
$$\mathrm{d[ptc]/d}t = +v_{19} - v_{20} - v_{21} + v_{22} \quad (176)$$

$$\mathrm{d[smo]/d}t = +v_{24} - v_{25} \quad (177)$$
$$\mathrm{d[shhPtch]/d}t = +v_{21} - v_{22} - v_{23} \quad (178)$$

The ODE system was solved by a parallelized implementation of the CVODES algorithm [1]. It also supplies the parameter sensitivities utilized for parameter estimation.

The initial conditions for the ODE system are given by:

$$\mathrm{[gli1](0)} = \mathrm{init\_gli1} \quad (179)$$
$$\mathrm{[gli1A](0)} = \mathrm{init\_gli1A} \quad (180)$$
$$\mathrm{[gli2](0)} = \mathrm{init\_gli2} \quad (181)$$
$$\mathrm{[gli2A](0)} = \mathrm{init\_gli2A} \quad (182)$$
$$\mathrm{[gli3](0)} = \mathrm{init\_gli3} \quad (183)$$
$$\mathrm{[gli3R](0)} = \mathrm{init\_gli3R} \quad (184)$$
$$\mathrm{pifo\_w}t\mathrm{(0)} = \mathrm{init\_pifo\_w}t \quad (185)$$
$$\mathrm{[pifo\_v](0)} = \mathrm{init\_pifo\_v} \quad (186)$$
$$\mathrm{[ptc\_mRNA](0)} = \mathrm{init\_ptc\_mRNA} \quad (187)$$
$$\mathrm{[ptc](0)} = \mathrm{init\_ptc} \quad (188)$$

$$\mathrm{[smo](0)} = \mathrm{init\_smo} \quad (189)$$
$$\mathrm{[shhPtch](0)} = \mathrm{init\_shhPtch} \quad (190)$$

The ODE system is modified by the following parameter transformations:

$$\mathrm{a1} \rightarrow \frac{\begin{array}{l}(\mathrm{init\_gli3R} \cdot \mathrm{r0} + 1) \cdot (\mathrm{b1} \cdot \mathrm{init\_gli1} + \mathrm{init\_gli1} \cdot \mathrm{init\_smo} \cdot \mathrm{t1} + \mathrm{b1} \cdot \mathrm{init\_gli1} \cdot \mathrm{init\_ptc} \cdot \mathrm{q0} + \mathrm{init\_gli1} \cdot \mathrm{init\_smo} \\ 1 \quad \cdot \mathrm{init\_pifo\_}wt \cdot \mathrm{t2} + \mathrm{g1} \cdot \mathrm{init\_gli1} \cdot \mathrm{init\_smo} \cdot \mathrm{init\_shhPtch} \cdot \mathrm{t1} + \mathrm{g1} \cdot \mathrm{init\_gli1} \cdot \mathrm{init\_smo} \cdot \mathrm{init\_shhPtch} \cdot \mathrm{init\_pifo\_}wt \cdot \mathrm{t2})\end{array}}{\mathrm{init\_gli2A} \cdot (\mathrm{g1} \cdot \mathrm{init\_shhPtch} + 1) \cdot (\mathrm{init\_ptc} \cdot \mathrm{q0} + 1)} \tag{191}$$

$$\mathrm{a15} \rightarrow \frac{\mathrm{b11} \cdot \mathrm{g9} \cdot \mathrm{init\_shhPtch}^2 + \mathrm{b11} \cdot \mathrm{init\_shhPtch} + \mathrm{b10} \cdot \mathrm{init\_ptc}}{\mathrm{init\_ptc\_mRNA} \cdot (\mathrm{g9} \cdot \mathrm{init\_shhPtch} + 1)} \tag{192}$$

$$\mathrm{a19} \rightarrow \frac{\mathrm{b11} \cdot \mathrm{init\_shhPtch} + \mathrm{d1} \cdot \mathrm{init\_shhPtch}}{\mathrm{SHH\_b} \cdot \mathrm{init\_ptc}} \tag{193}$$

$$\mathrm{a3} \rightarrow \frac{\begin{array}{l}(\mathrm{init\_gli3R} \cdot \mathrm{r1} + 1) \cdot (\mathrm{b3} \cdot \mathrm{init\_gli2} + \mathrm{init\_gli2} \cdot \mathrm{init\_smo} \cdot \mathrm{t3} + \mathrm{b3} \cdot \mathrm{init\_gli2} \cdot \mathrm{init\_ptc} \cdot \mathrm{q0} + \mathrm{init\_gli2} \cdot \\ 1 \quad \mathrm{init\_smo} \cdot \mathrm{init\_pifo\_}wt \cdot \mathrm{t4} + \mathrm{g3} \cdot \mathrm{init\_gli2} \cdot \mathrm{init\_smo} \cdot \mathrm{init\_shhPtch} \cdot \mathrm{t3} + \mathrm{g3} \cdot \mathrm{init\_gli2} \cdot \mathrm{init\_smo} \cdot \mathrm{init\_shhPtch} \cdot \mathrm{init\_pifo\_}wt \cdot \mathrm{t4})\end{array}}{\mathrm{init\_gli2A} \cdot (\mathrm{g3} \cdot \mathrm{init\_shhPtch} + 1) \cdot (\mathrm{init\_ptc} \cdot \mathrm{q0} + 1)} \tag{194}$$

$$\mathrm{a5} \rightarrow \frac{\begin{array}{l}(\mathrm{init\_gli3R} \cdot \mathrm{r2} + 1) \cdot (\mathrm{b5} \cdot \mathrm{init\_gli3} + \mathrm{init\_gli3} \cdot \mathrm{init\_ptc} \cdot \mathrm{t5} + \mathrm{b5} \cdot \mathrm{init\_gli3} \cdot \mathrm{init\_smo} \cdot \mathrm{q2} + \mathrm{init\_gli3} \cdot \mathrm{init\_ptc} \cdot \\ 1 \quad \mathrm{init\_pifo\_}wt \cdot \mathrm{t6} + \mathrm{b5} \cdot \mathrm{init\_gli3} \cdot \mathrm{init\_smo} \cdot \mathrm{init\_pifo\_}wt \cdot \mathrm{q1} + \mathrm{g5} \cdot \mathrm{init\_gli3} \cdot \mathrm{init\_ptc} \cdot \mathrm{init\_shhPtch} \cdot \mathrm{t5} + \mathrm{g5} \cdot \mathrm{init\_gli3} \cdot \mathrm{init\_ptc} \cdot \mathrm{init\_shhPtch} \cdot \mathrm{init\_pifo\_}wt \cdot \mathrm{t6})\end{array}}{\mathrm{init\_gli2A} \cdot (\mathrm{g5} \cdot \mathrm{init\_shhPtch} + 1) \cdot (\mathrm{init\_smo} \cdot \mathrm{q2} + \mathrm{init\_smo} \cdot \mathrm{init\_pifo\_}wt \cdot \mathrm{q1} + 1)} \tag{195}$$

$$\mathrm{b2} \rightarrow \frac{(\mathrm{init\_gli1} \cdot \mathrm{init\_smo} \cdot \mathrm{t1} + \mathrm{init\_gli1} \cdot \mathrm{init\_smo} \cdot \mathrm{init\_pifo\_}wt \cdot \mathrm{t2}) \cdot (\mathrm{g2} \cdot \mathrm{init\_shhPtch} + 1)}{\mathrm{init\_gli1A} \cdot (\mathrm{init\_ptc} \cdot \mathrm{q0} + 1)} \tag{196}$$

$$\mathrm{b4} \rightarrow \frac{(\mathrm{init\_gli2} \cdot \mathrm{init\_smo} \cdot \mathrm{t3} + \mathrm{init\_gli2} \cdot \mathrm{init\_smo} \cdot \mathrm{init\_pifo\_}wt \cdot \mathrm{t4}) \cdot (\mathrm{g4} \cdot \mathrm{init\_shhPtch} + 1)}{\mathrm{init\_gli2A} \cdot (\mathrm{init\_ptc} \cdot \mathrm{q0} + 1)} \tag{197}$$

$$\mathrm{b6} \rightarrow \frac{(\mathrm{init\_gli3} \cdot \mathrm{init\_ptc} \cdot \mathrm{t5} + \mathrm{init\_gli3} \cdot \mathrm{init\_ptc} \cdot \mathrm{init\_pifo\_}wt \cdot \mathrm{t6}) \cdot (\mathrm{g6} \cdot \mathrm{init\_shhPtch} + 1)}{\mathrm{init\_gli3R} \cdot (\mathrm{init\_smo} \cdot \mathrm{q2} + \mathrm{init\_smo} \cdot \mathrm{init\_pifo\_}wt \cdot \mathrm{q1} + 1)} \tag{198}$$

$$\mathrm{b7} \rightarrow \frac{a\_wt2 \cdot \mathrm{init\_gli2A} \cdot (\mathrm{g7} \cdot \mathrm{init\_shhPtch} + 1)}{\mathrm{init\_pifo\_}wt \cdot (\mathrm{init\_gli3R} \cdot \mathrm{r3} + 1)} \tag{199}$$

$$\mathrm{b9} \rightarrow \frac{\mathrm{init\_gli2A} \cdot \mathrm{k1}}{\mathrm{init\_ptc\_mRNA} \cdot (\mathrm{init\_gli3R} \cdot \mathrm{r4} + 1)} \tag{200}$$

$$\mathrm{smo\_base} \rightarrow \frac{\mathrm{b12} \cdot \mathrm{init\_smo}}{\mathrm{g10} \cdot \mathrm{init\_shhPtch} + 1} \tag{201}$$

$$\tag{202}$$

5.1 Experiment: Pifo$^{wt/wt}$

The model outputs available in this data set are defined by:

$$\text{gli1\_gli1A\_obs1} = \text{scale\_gli11} \cdot ([\text{gli1}] + [\text{gli1A}]) \tag{203}$$
$$\text{gli1\_gli1A\_obs2} = \text{scale\_gli12} \cdot ([\text{gli1}] + [\text{gli1A}]) \tag{204}$$
$$\text{gli1\_gli1A\_obs3} = \text{scale\_gli13} \cdot ([\text{gli1}] + [\text{gli1A}]) \tag{205}$$
$$\text{gli2\_gli2A\_obs1} = \text{scale\_gli21} \cdot ([\text{gli2}] + [\text{gli2A}]) \tag{206}$$
$$\text{gli2\_gli2A\_obs2} = \text{scale\_gli22} \cdot ([\text{gli2}] + [\text{gli2A}]) \tag{207}$$
$$\text{gli2\_gli2A\_obs3} = \text{scale\_gli23} \cdot ([\text{gli2}] + [\text{gli2A}]) \tag{208}$$
$$\text{gli3\_obs1} = [\text{gli3}] \cdot \text{scale\_gli31} \tag{209}$$
$$\text{gli3\_obs2} = [\text{gli3}] \cdot \text{scale\_gli3} \tag{210}$$
$$\text{pifo\_obs1} = \text{pifo\_w}t \cdot \text{scale\_pifo1} \tag{211}$$
$$\text{pifo\_obs2} = \text{pifo\_w}t \cdot \text{scale\_pifo2} \tag{212}$$

$$\text{gli3R\_obs1} = [\text{gli3R}] \cdot \text{scale\_gli3R1} \tag{213}$$
$$\text{gli3R\_obs2} = [\text{gli3R}] \cdot \text{scale\_gli3} \tag{214}$$
$$\text{ptc\_mRNA\_obs} = [\text{ptc\_mRNA}] \cdot \text{scale\_mRNA} \tag{215}$$

The error model that describes the measurement noise for each model output is given by:

$$\text{gli1\_gli1A\_obs1} = \text{sd\_gli1\_gli1A} \tag{216}$$
$$\text{gli1\_gli1A\_obs2} = \text{sd\_gli1\_gli1A} \tag{217}$$
$$\text{gli1\_gli1A\_obs3} = \text{sd\_gli1\_gli1A} \tag{218}$$
$$\text{gli2\_gli2A\_obs1} = \text{sd\_gli2\_gli2A} \tag{219}$$
$$\text{gli2\_gli2A\_obs2} = \text{sd\_gli2\_gli2A} \tag{220}$$
$$\text{gli2\_gli2A\_obs3} = \text{sd\_gli2\_gli2A} \tag{221}$$
$$\text{gli3\_obs1} = \text{sd\_gli3} \tag{222}$$
$$\text{gli3\_obs2} = \text{sd\_gli3} \tag{223}$$
$$\text{pifo\_obs1} = \text{sd\_pifo} \tag{224}$$
$$\text{pifo\_obs2} = \text{sd\_pifo} \tag{225}$$

$$\text{gli3R\_obs1} = \text{sd\_gli3R} \tag{226}$$
$$\text{gli3R\_obs2} = \text{sd\_gli3R} \tag{227}$$
$$\text{ptc\_mRNA\_obs} = \text{sd\_mRNA} \tag{228}$$

To evaluate the ODE system of Equation 167 – 190 for the conditions in this experiment, the following parameter transformations are applied:

$$a\_v2 \rightarrow 0$$
$$\text{init\_pifo\_v} \rightarrow 0$$

The agreement of the model outputs and the experimental data, given in Table 16, yields a value of the objective function $-2\log(L) = -174.975$ for 194 data points in this data set.

time [hour]	gli1_gli1A_obs1 conc. [au]	gli1_gli1A_obs2 conc. [au]	gli1_gli1A_obs3 conc. [au]	gli2_gli2A_obs1 conc. [au]	gli2_gli2A_obs2 conc. [au]	gli2_gli2A_obs3 conc. [au]	gli3_obs1 conc. [au]	gli3_obs2 conc. [au]	pifo_obs1 conc. [au]	pifo_obs2 conc. [au]	gli3R_obs1 conc. [au]	gli3R_obs2 conc. [au]	ptc_mRNA_obs conc. [au]
0.000000	0.015564	0.0214446	0.0363197	0.178222	0.200916	0.056895	0.212785	0.000676336	0.0628137	0.0471802	0.54038	NaN	0.0177638
0.000000	0.00855469	0.0203686	0.0361234	0.185775	0.313453	0.0571	NaN	NaN	NaN	0.046892	NaN	0.774863	NaN
0.000000	0.0199044	0.0278261	0.0522542	0.198058	0.342151	0.0541885	NaN	NaN	NaN	0.041193	NaN	NaN	NaN
0.500000	0.0241291	0.0367793	0.0880664	0.434706	0.224972	0.355579	0.286852	0.313212	0.138315	0.136339	0.668428	NaN	0.0538905
0.500000	0.0389373	0.0330013	0.0756739	0.416143	0.364545	0.290495	NaN	NaN	NaN	0.108673	NaN	0.87813	NaN
0.500000	0.0360606	0.0519847	0.0880195	0.427444	0.344853	0.382099	NaN	NaN	NaN	0.170844	NaN	NaN	NaN
1.000000	0.0303636	0.0558414	0.128295	0.391266	0.370038	0.293007	0.320029	0.445293	0.532863	0.25741	0.833513	NaN	0.0866669
1.000000	0.0853232	0.0449743	0.120734	0.408369	0.554766	0.291326	NaN	NaN	NaN	0.233159	NaN	0.914891	NaN
1.000000	0.0980148	0.084227	0.13074	0.429824	0.570069	0.450989	NaN	NaN	NaN	0.372068	NaN	NaN	NaN
2.000000	0.0750276	0.0784564	0.164607	0.723385	0.351728	0.600716	0.739916	0.945608	1	0.791907	0.842011	NaN	0.305466
2.000000	0.147758	0.0638573	0.15793	0.72222	0.603407	0.561212	NaN	NaN	NaN	0.707039	NaN	1	NaN
2.000000	0.163165	0.0986687	0.165783	0.738104	0.585102	0.775977	NaN	NaN	NaN	1	NaN	NaN	NaN
3.000000	NaN	NaN	NaN	NaN	NaN	NaN	NaN	NaN	NaN	NaN	NaN	NaN	NaN
3.000000	NaN	NaN	NaN	NaN	NaN	NaN	NaN	NaN	NaN	NaN	NaN	NaN	NaN
3.000000	NaN	NaN	NaN	NaN	NaN	NaN	NaN	NaN	NaN	NaN	NaN	NaN	NaN
4.000000	0.128378	0.103976	0.365019	0.991599	0.623771	1	1	1	0.811109	0.493087	0.897436	NaN	0.589372
4.000000	0.202314	0.0835847	0.23147	0.929583	0.957862	0.932811	NaN	NaN	NaN	0.358881	NaN	0.979466	NaN
4.000000	0.213664	0.150569	0.219145	1	1	0.978299	NaN	NaN	NaN	0.674253	NaN	NaN	NaN
6.000000	0.218872	0.231773	0.381829	0.757754	0.588877	0.734054	0.839698	1	0.644192	0.33405	1	NaN	1
6.000000	0.400663	0.230555	0.402404	0.821881	0.953563	0.778089	NaN	NaN	NaN	0.296624	NaN	NaN	NaN
6.000000	0.437522	0.35172	0.373627	0.803729	0.978667	0.903556	NaN	NaN	NaN	0.458962	NaN	NaN	NaN
24.000000	0.843905	0.717574	1	0.546695	0.526224	0.545752	0.419506	0.55356	0.554984	0.321622	0.211071	NaN	0.771944
24.000000	0.971295	0.694357	0.787677	0.61271	0.819188	0.524619	NaN	NaN	NaN	0.258336	NaN	0.529352	NaN
24.000000	1	1	0.996629	0.596012	0.869489	0.728985	NaN	NaN	NaN	0.463376	NaN	NaN	NaN
48.000000	0.158902	NaN	NaN	NaN	0.367042	NaN	NaN	NaN	NaN	NaN	NaN	NaN	NaN
48.000000	0.278934	NaN	NaN	NaN	0.564599	NaN	NaN	NaN	NaN	NaN	NaN	NaN	NaN
48.000000	0.399441	NaN	NaN	NaN	0.600441	NaN	NaN	NaN	NaN	NaN	NaN	NaN	NaN

Table 16: Experimental data for the experiment Pifo$^{wt/wt}$

5.2 Experiment: Pifo Rescue Clone 1

The model outputs available in this data set are defined by:

$$\text{gli1\_gli1A\_obs} = \text{scale\_gli1\_clone03} \cdot ([\text{gli1}] + [\text{gli1A}]) \tag{229}$$

$$\text{gli2\_gli2A\_obs} = \text{scale\_gli2\_clone03} \cdot ([\text{gli2}] + [\text{gli2A}]) \tag{230}$$

$$\text{venus\_pifo\_obs} = [\text{pifo\_v}] \cdot \text{scale\_venus\_pifo03} \tag{231}$$

$$\text{wt\_pifo\_obs} = \frac{\text{pifo\_}wt}{\text{init\_pifo\_}wt} \tag{232}$$

$$\text{dummy\_pifo\_obs} = \text{scale\_dummy} \cdot ([\text{pifo\_v}] + \text{pifo\_}wt) \tag{233}$$

The error model that describes the measurement noise for each model output is given by:

$$\text{gli1\_gli1A\_obs} = \text{sd\_gli1\_gli1A\_clone} \tag{234}$$

$$\text{gli2\_gli2A\_obs} = \text{sd\_gli2\_gli2A\_clone} \tag{235}$$

$$\text{venus\_pifo\_obs} = \text{sd\_venus\_pifo} \tag{236}$$

$$\text{wt\_pifo\_obs} = 0.01 \tag{237}$$

$$\text{dummy\_pifo\_obs} = 0.05 \tag{238}$$

To evaluate the ODE system of Equation 167 – 178 for the conditions in this experiment, the following external inputs are given:

$$[\text{SHH}](t) = -\text{SHH\_level} \cdot \left(\frac{1}{\mathrm{e}^{100000000.0 \cdot t - 10000000000.0} + 1} - 1 \right) \tag{239}$$

$$a\_pifo2(t) = \frac{a\_wt2}{\mathrm{e}^{100000000.0 \cdot t - 5000000000.0} + 1} \tag{240}$$

To evaluate the ODE system of Equation 167 – 190 for the conditions in this experiment, the following parameter transformations are applied:

$$a\_v2 \rightarrow a\_v32$$
$$\text{init\_pifo\_v} \rightarrow 0$$

The agreement of the model outputs and the experimental data, given in Table 17, yields a value of the objective function $-2\log(L) = -107.704$ for 84 data points in this data set.

time [hour]	gli1_gli1A_obs conc. [au]	gli2_gli2A_obs conc. [au]	venus_pifo_obs conc. [au]	wt_pifo_obs conc. [au]	dummy_pifo_obs conc. [au]
40.000000	NaN	NaN	NaN	NaN	1
41.000000	NaN	NaN	NaN	NaN	1
42.000000	NaN	NaN	NaN	NaN	1
43.000000	NaN	NaN	NaN	NaN	1
44.000000	NaN	NaN	NaN	NaN	1
45.000000	NaN	NaN	NaN	NaN	1
46.000000	NaN	NaN	NaN	NaN	1
47.000000	NaN	NaN	NaN	NaN	1
48.000000	NaN	NaN	NaN	NaN	1
49.000000	NaN	NaN	NaN	NaN	1
50.000000	NaN	NaN	NaN	NaN	1
100.000000	0.182833	0.463423	0.120944	0.01	NaN
100.000000	0.240915	0.692974	0.0922276	NaN	NaN
100.000000	0.282956	0.739093	0.153121	NaN	NaN
100.500000	0.0575001	0.540771	0.567789	NaN	NaN
100.500000	0.114786	0.783652	0.46482	NaN	NaN
100.500000	0.112337	0.841869	0.721076	NaN	NaN
101.000000	0.0885261	0.523308	0.652547	NaN	NaN
101.000000	0.103852	0.708793	0.535404	NaN	NaN
101.000000	0.117757	0.752356	0.733981	NaN	NaN
102.000000	0.130983	0.676713	0.792149	NaN	NaN
102.000000	0.136415	0.945411	0.595297	NaN	NaN
102.000000	0.177838	1	1	NaN	NaN
104.000000	0.267093	0.427406	0.29869	NaN	NaN
104.000000	0.299726	0.706476	0.276722	NaN	NaN
104.000000	0.285777	0.691151	0.405361	NaN	NaN
106.000000	0.35012	0.386636	0.0498918	NaN	NaN
106.000000	0.347542	0.69705	0.0924	NaN	NaN
106.000000	0.40814	0.738608	0.124777	NaN	NaN
124.000000	1	0.120606	0.0490282	NaN	NaN
124.000000	0.953868	0.228043	0.0269504	NaN	NaN
124.000000	0.992917	0.248928	0.168091	NaN	NaN
148.000000	0.746878	0.00927686	0.112661	NaN	NaN
148.000000	0.721883	0.0150845	0.106491	NaN	NaN
148.000000	0.725758	0.0154096	0.359014	NaN	NaN

Table 17: Experimental data for the experiment Pifo Rescue Clone 1

time [hour]	gli1_gli1A_obs conc. [au]	gli2_gli2A_obs conc. [au]	venus_pifo_obs conc. [au]
100.000000	0.0368266	0.184742	0.0916695
100.000000	0.0560124	0.261114	0.112316
100.000000	0.0644724	0.316429	0.253573
100.500000	0.0718045	0.192446	0.193969
100.500000	0.111495	0.365173	0.199484
100.500000	0.0747356	0.362317	0.463917
101.000000	0.045422	0.634537	0.158465
101.000000	0.0799631	0.995833	0.314363
101.000000	0.0843231	0.970766	0.583984
102.000000	0.0648619	0.612743	0.466756
102.000000	0.0868798	0.978037	0.572587
102.000000	0.0550461	1	1
104.000000	0.0669407	0.207321	0.342571
104.000000	0.107634	0.37937	0.50336
104.000000	0.0402828	0.389485	0.870908
106.000000	0.0690348	0.279133	0.355211
106.000000	0.118163	0.476426	0.272168
106.000000	0.0375131	0.489825	NaN
124.000000	0.166348	0.00811415	0.0857238
124.000000	0.189582	0.00643713	0.164051
124.000000	0.186287	0.00174642	0.35449
148.000000	0.853527	0.00964686	0.125416
148.000000	0.931884	0.0131668	0.110746
148.000000	1	0.014068	0.256562

Table 18: Experimental data for the experiment Pifo Rescue Clone 2

5.3 Experiment: Pifo Rescue Clone 2

The model outputs available in this data set are defined by:

$$\text{gli1\_gli1A\_obs} = \text{scale\_gli1\_clone04} \cdot ([\text{gli1}] + [\text{gli1A}]) \tag{241}$$

$$\text{gli2\_gli2A\_obs} = \text{scale\_gli2\_clone04} \cdot ([\text{gli2}] + [\text{gli2A}]) \tag{242}$$

$$\text{venus\_pifo\_obs} = [\text{pifo\_v}] \cdot \text{scale\_venus\_pifo04} \tag{243}$$

The error model that describes the measurement noise for each model output is given by:

$$\text{gli1\_gli1A\_obs} = \text{sd\_gli1\_gli1A\_clone} \tag{244}$$

$$\text{gli2\_gli2A\_obs} = \text{sd\_gli2\_gli2A\_clone} \tag{245}$$

$$\text{venus\_pifo\_obs} = \text{sd\_venus\_pifo} \tag{246}$$

To evaluate the ODE system of Equation 167 – 178 for the conditions in this experiment, the following external inputs are given:

$$[\text{SHH}](t) = -\text{SHH\_level} \cdot \left(\frac{1}{\mathrm{e}^{100000000.0 \cdot t - 10000000000.0} + 1} - 1\right) \tag{247}$$

$$a\_pifo2(t) = \frac{a\_wt2}{\mathrm{e}^{100000000.0 \cdot t - 5000000000.0} + 1} \tag{248}$$

To evaluate the ODE system of Equation 167 – 190 for the conditions in this experiment, the following parameter transformations are applied:

$$a\_v2 \rightarrow a\_v42$$
$$\text{init\_pifo\_v} \rightarrow 0$$

The agreement of the model outputs and the experimental data, given in Table 18, yields a value of the objective function $-2\log(L) = -68.9435$ for 71 data points in this data set.

time [hour]	gli1_gli1A_obs conc. [au]	gli2_gli2A_obs conc. [au]	venus_pifo_obs conc. [au]
100.000000	0.034737	0.10516	0.0531111
100.000000	0.0370827	0.180143	0.00408836
100.000000	0.0367413	0.206412	0.0279515
100.500000	0.0479489	0.213912	0.035578
100.500000	0.0576734	0.302668	0.0241159
100.500000	0.115088	0.352421	0.0622776
101.000000	0.0583906	0.320974	0.0387209
101.000000	0.0536062	0.506592	0.0330925
101.000000	0.0702539	0.534023	0.109485
102.000000	0.0793571	0.371164	0.377696
102.000000	0.0962706	0.584093	0.354038
102.000000	0.0960704	0.617612	0.402495
104.000000	0.227568	0.612039	0.538538
104.000000	0.194355	0.902327	0.641777
104.000000	0.252053	1	1
106.000000	0.20249	0.240238	0.464928
106.000000	0.207116	0.3771	0.331168
106.000000	0.197015	0.426412	0.705254
124.000000	0.891786	0.0148126	0.071469
124.000000	0.920639	0.0191236	0.0520516
124.000000	1	0.0388116	0.223205
148.000000	0.828158	0.0135249	0.0600894
148.000000	0.836879	0.0148126	0.0447066
148.000000	0.889168	0.0329954	0.219787

Table 19: Experimental data for the experiment Pifo Rescue Clone 3

5.4 Experiment: Pifo Rescue Clone 3

The model outputs available in this data set are defined by:

$$\text{gli1\_gli1A\_obs} = \text{scale\_gli1\_clone06} \cdot ([\text{gli1}] + [\text{gli1A}]) \quad (249)$$
$$\text{gli2\_gli2A\_obs} = \text{scale\_gli2\_clone06} \cdot ([\text{gli2}] + [\text{gli2A}]) \quad (250)$$
$$\text{venus\_pifo\_obs} = [\text{pifo\_v}] \cdot \text{scale\_venus\_pifo06} \quad (251)$$

The error model that describes the measurement noise for each model output is given by:

$$\text{gli1\_gli1A\_obs} = \text{sd\_gli1\_gli1A\_clone} \quad (252)$$
$$\text{gli2\_gli2A\_obs} = \text{sd\_gli2\_gli2A\_clone} \quad (253)$$
$$\text{venus\_pifo\_obs} = \text{sd\_venus\_pifo} \quad (254)$$

To evaluate the ODE system of Equation 167 – 178 for the conditions in this experiment, the following external inputs are given:

$$[\text{SHH}](t) = -\text{SHH\_level} \cdot \left(\frac{1}{e^{100000000.0 \cdot t - 10000000000.0} + 1} - 1\right) \quad (255)$$
$$a\_pifo2(t) = \frac{a\_wt2}{e^{100000000.0 \cdot t - 5000000000.0} + 1} \quad (256)$$

To evaluate the ODE system of Equation 167 – 190 for the conditions in this experiment, the following parameter transformations are applied:

$$a\_v2 \rightarrow a\_v62$$
$$\text{init\_pifo\_v} \rightarrow 0$$

The agreement of the model outputs and the experimental data, given in Table 19, yields a value of the objective function $-2\log(L) = -75.0957$ for 72 data points in this data set.

time [hour]	gli1_gli1A_obs conc. [au]	gli2_gli2A_obs conc. [au]	venus_pifo_obs conc. [au]
100.000000	0.0733807	0.0557874	0.155678
100.000000	0.0752167	0.109196	0.0967391
100.000000	0.137857	0.118231	0.340206
100.500000	0.197893	0.267746	0.206915
100.500000	0.240754	0.394542	0.167624
100.500000	0.281363	0.42826	0.2639
101.000000	0.180212	0.450281	0.143651
101.000000	0.116151	0.656081	0.100912
101.000000	0.179284	0.676206	0.230983
102.000000	0.102097	0.669935	0.482911
102.000000	0.0427575	0.93507	0.304761
102.000000	0.0562424	1	0.531177
104.000000	0.0632155	0.488317	0.570103
104.000000	0.211053	0.709617	0.640054
104.000000	0.333295	0.721789	1
106.000000	0.136904	0.390879	0.149793
106.000000	0.148629	0.562657	0.192597
106.000000	0.257559	0.604689	0.396533
124.000000	0.833681	0.135585	0.0822378
124.000000	0.9674	0.207241	0.196406
124.000000	1	0.253548	0.302234
148.000000	0.26162	0.0153155	0.190012
148.000000	0.425067	0.0130094	0.327301
148.000000	0.57261	0.0165319	0.565653

Table 20: Experimental data for the experiment Pifo Rescue Clone 4

5.5 Experiment: Pifo Rescue Clone 4

The model outputs available in this data set are defined by:

$$\text{gli1\_gli1A\_obs} = \text{scale\_gli1\_clone07} \cdot ([\text{gli1}] + [\text{gli1A}]) \quad (257)$$

$$\text{gli2\_gli2A\_obs} = \text{scale\_gli2\_clone07} \cdot ([\text{gli2}] + [\text{gli2A}]) \quad (258)$$

$$\text{venus\_pifo\_obs} = [\text{pifo\_v}] \cdot \text{scale\_venus\_pifo07} \quad (259)$$

The error model that describes the measurement noise for each model output is given by:

$$\text{gli1\_gli1A\_obs} = \text{sd\_gli1\_gli1A\_clone} \quad (260)$$

$$\text{gli2\_gli2A\_obs} = \text{sd\_gli2\_gli2A\_clone} \quad (261)$$

$$\text{venus\_pifo\_obs} = \text{sd\_venus\_pifo} \quad (262)$$

To evaluate the ODE system of Equation 167 – 178 for the conditions in this experiment, the following external inputs are given:

$$[\text{SHH}](t) = -\text{SHH\_level} \cdot \left(\frac{1}{e^{100000000.0 \cdot t - 10000000000.0} + 1} - 1 \right) \quad (263)$$

$$a\_pifo2(t) = \frac{a\_wt2}{e^{100000000.0 \cdot t - 5000000000.0} + 1} \quad (264)$$

To evaluate the ODE system of Equation 167 – 190 for the conditions in this experiment, the following parameter transformations are applied:

$$a\_v2 \rightarrow a\_v72$$
$$\text{init\_pifo\_v} \rightarrow 0$$

The agreement of the model outputs and the experimental data, given in Table 20, yields a value of the objective function $-2\log(L) = -50.5278$ for 72 data points in this data set.

time [hour]	gli1_gli1A_obs conc. [au]	gli2_gli2A_obs conc. [au]	venus_pifo_obs conc. [au]
100.000000	0.0739123	0.1948	0.198494
100.000000	0.0636712	0.31717	0.179004
100.000000	0.0657867	0.323546	0.372559
100.500000	0.1244	0.506959	0.393045
100.500000	0.138967	0.731195	0.447628
100.500000	0.239535	0.762084	0.754138
101.000000	0.783126	0.563019	0.4447
101.000000	0.854814	0.799054	0.600356
101.000000	1	0.823444	0.933172
102.000000	0.639209	0.478818	0.249208
102.000000	0.602416	0.694089	0.234393
102.000000	0.751857	0.729338	0.456093
104.000000	0.429773	0.571087	0.293654
104.000000	0.535049	0.790858	0.342568
104.000000	0.488277	0.870161	0.730114
106.000000	0.0606591	0.639728	0.532743
106.000000	0.076192	0.920131	0.476698
106.000000	0.0680909	1	0.829352
124.000000	0.576613	0.420892	0.254142
124.000000	0.679178	0.573901	0.242631
124.000000	0.666618	0.659444	0.525376
148.000000	0.605284	0.0186576	0.425407
148.000000	0.700978	0.0108751	0.313511
148.000000	0.676982	0.023538	1

Table 21: Experimental data for the experiment Pifo Rescue Clone 5

5.6 Experiment: Pifo Rescue Clone 5

The model outputs available in this data set are defined by:

$$\text{gli1\_gli1A\_obs} = \text{scale\_gli1\_clone08} \cdot ([\text{gli1}] + [\text{gli1A}]) \tag{265}$$

$$\text{gli2\_gli2A\_obs} = \text{scale\_gli2\_clone08} \cdot ([\text{gli2}] + [\text{gli2A}]) \tag{266}$$

$$\text{venus\_pifo\_obs} = [\text{pifo\_v}] \cdot \text{scale\_venus\_pifo08} \tag{267}$$

The error model that describes the measurement noise for each model output is given by:

$$\text{gli1\_gli1A\_obs} = \text{sd\_gli1\_gli1A\_clone} \tag{268}$$

$$\text{gli2\_gli2A\_obs} = \text{sd\_gli2\_gli2A\_clone} \tag{269}$$

$$\text{venus\_pifo\_obs} = \text{sd\_venus\_pifo} \tag{270}$$

To evaluate the ODE system of Equation 167 – 178 for the conditions in this experiment, the following external inputs are given:

$$[\text{SHH}](t) = -\text{SHH\_level} \cdot \left(\frac{1}{e^{100000000.0 \cdot t - 10000000000.0} + 1} - 1 \right) \tag{271}$$

$$a\_pifo2(t) = \frac{a\_wt2}{e^{100000000.0 \cdot t - 5000000000.0} + 1} \tag{272}$$

To evaluate the ODE system of Equation 167 – 190 for the conditions in this experiment, the following parameter transformations are applied:

$$a\_v2 \rightarrow a\_v82$$
$$\text{init\_pifo\_v} \rightarrow 0$$

The agreement of the model outputs and the experimental data, given in Table 21, yields a value of the objective function $-2\log(L) = 20.3828$ for 72 data points in this data set.

time [hour]	gli1_gli1A_obs conc. [au]	gli2_gli2A_obs conc. [au]
120.000000	0.0148293	0.14167
120.000000	0.255391	0.422122
120.000000	0.2119	0.536753
120.500000	0.140849	0.121386
120.500000	0.217946	0.639094
120.500000	0.24315	0.518478
121.000000	0.237411	0.274256
121.000000	0.337304	0.804296
121.000000	0.464681	0.994742
122.000000	0.292327	0.217122
122.000000	0.462761	0.838048
122.000000	0.630735	1
123.000000	NaN	NaN
123.000000	NaN	NaN
123.000000	NaN	NaN
124.000000	0.202883	0.118393
124.000000	0.536092	0.556568
124.000000	0.787388	0.760585
126.000000	0.135418	0.148018
126.000000	0.363123	0.236529
126.000000	0.528869	0.496442
144.000000	0.0974319	0.173303
144.000000	0.530863	0.159731
144.000000	0.491572	0.83185
168.000000	0.320396	0.0582678
168.000000	1	0.456963
168.000000	0.802135	0.14838

Table 22: Experimental data for the experiment Pifo$^{FD/FD}$

5.7 Experiment: Pifo$^{FD/FD}$

The model outputs available in this data set are defined by:

$$\text{gli1\_gli1A\_obs} = \text{scale\_gli1\_}ko \cdot ([\text{gli1}] + [\text{gli1A}]) \tag{273}$$
$$\text{gli2\_gli2A\_obs} = \text{scale\_gli2\_}ko \cdot ([\text{gli2}] + [\text{gli2A}]) \tag{274}$$

The error model that describes the measurement noise for each model output is given by:

$$\text{gli1\_gli1A\_obs} = \text{sd\_gli1\_gli1A\_}ko \tag{275}$$
$$\text{gli2\_gli2A\_obs} = \text{sd\_gli2\_gli2A\_}ko \tag{276}$$

To evaluate the ODE system of Equation 167 − 178 for the conditions in this experiment, the following external inputs are given:

$$[\text{SHH}](t) = -\text{SHH\_level} \cdot \left(\frac{1}{e^{100000000.0 \cdot t - 12000000000.0} + 1} - 1 \right) \tag{277}$$
$$a\_pifo2(t) = \frac{a\_wt2}{e^{100000000.0 \cdot t - 500000000.0} + 1} \tag{278}$$

To evaluate the ODE system of Equation 167 − 190 for the conditions in this experiment, the following parameter transformations are applied:

$$a\_v2 \rightarrow 0$$
$$\text{init\_pifo\_v} \rightarrow 0$$

The agreement of the model outputs and the experimental data, given in Table 22, yields a value of the objective function $-2\log(L) = 23.2751$ for 48 data points in this data set.

	name	θ_{min}	$\hat{\theta}$	θ_{max}	log	non-log $\hat{\theta}$	fitted
1	SHH_b	-7	-2.5669	-1	1	$+2.71 \cdot 10^{-03}$	1
2	SHH_level	-5	-0.0910	+2	1	$+8.11 \cdot 10^{-01}$	1
3	a_v32	-5	+0.3513	+3	1	$+2.25 \cdot 10^{+00}$	1
4	a_v42	-5	+1.3395	+3	1	$+2.19 \cdot 10^{+01}$	1
5	a_v62	-5	+1.2803	+2	1	$+1.91 \cdot 10^{+01}$	1
6	a_v72	-5	+0.8116	+3	1	$+6.48 \cdot 10^{+00}$	1
7	a_v82	-5	+0.5421	+3	1	$+3.48 \cdot 10^{+00}$	1
8	a_wt2	-3	+0.0179	+1	1	$+1.04 \cdot 10^{+00}$	1
9	b1	-5	+1.9899	+2	1	$+9.77 \cdot 10^{+01}$	1
10	b10	-5	-1.4002	+1	1	$+3.98 \cdot 10^{-02}$	1
11	b11	-5	-0.7914	+2	1	$+1.62 \cdot 10^{-01}$	1
12	b12	-5	-0.3337	+2	1	$+4.64 \cdot 10^{-01}$	1
13	b3	-5	-1.0019	-1	1	$+9.96 \cdot 10^{-02}$	1
14	b5	-5	+1.0320	+3	1	$+1.08 \cdot 10^{+01}$	1
15	d1	-5	+0.1622	+3	1	$+1.45 \cdot 10^{+00}$	1
16	g1	-5	-1.7326	+1	1	$+1.85 \cdot 10^{-02}$	1
17	g10	-5	-0.0097	+2	1	$+9.78 \cdot 10^{-01}$	1
18	g2	-5	+2.9401	+3	1	$+8.71 \cdot 10^{+02}$	1
19	g3	-5	+0.1703	+1	1	$+1.48 \cdot 10^{+00}$	1
20	g4	-5	-2.8274	+1	1	$+1.49 \cdot 10^{-03}$	1
21	g5	-5	+0.3042	+1	1	$+2.01 \cdot 10^{+00}$	1
22	g6	-5	+2.3372	+3	1	$+2.17 \cdot 10^{+02}$	1
23	g7	-5	-0.7309	+1	1	$+1.86 \cdot 10^{-01}$	1
24	g9	-5	-3.0359	+3	1	$+9.21 \cdot 10^{-04}$	1

Table 23: Estimated parameter values
$\hat{\theta}$ indicates the estimated value of the parameters. θ_{min} and θ_{max} indicate the upper and lower bounds for the parameters. The log-column indicates if the value of a parameter was log-transformed. If log = 1 the non-log-column indicates the non-logarithmic value of the estimate. The fitted-column indicates if the parameter value was estimated (1), was temporarily fixed (0) or if its value was fixed to a constant value (2).

6 Estimated model parameters

The model parameter were estimated by maximum likelihood estimation applying the MATLAB lsqnonlin algorithm. In Table 23 – 26 the estimated parameter values are given. Parameters highlighted in red color indicate parameter values close to their bounds. The parameter name prefix init_ indicates the initial value of a dynamic variable. The parameter name prefix offset_ indicates a offset of the experimental data. The parameter name prefix scale_ indicates a scaling factor of the experimental data. The parameter name prefix sd_ indicates the magnitude of the measurement noise for a specific measurement.

	name	θ_{min}	$\hat{\theta}$	θ_{max}	log	non-log $\hat{\theta}$	fitted
25	init_gli1	-5	-1.2586	+1	1	$+5.51 \cdot 10^{-02}$	1
26	init_gli1A	-5	-2.0370	+1	1	$+9.18 \cdot 10^{-03}$	1
27	init_gli2	-5	-1.6138	+2	1	$+2.43 \cdot 10^{-02}$	1
28	init_gli2A	-1	-0.3264	+1	1	$+4.72 \cdot 10^{-01}$	1
29	init_gli3	-5	-0.6828	+1	1	$+2.08 \cdot 10^{-01}$	1
30	init_gli3R	-5	+0.3555	+1	1	$+2.27 \cdot 10^{+00}$	1
31	init_pifo_wt	-5	-0.9364	+1	1	$+1.16 \cdot 10^{-01}$	1
32	init_ptc	-5	+1.1151	+2	1	$+1.30 \cdot 10^{+01}$	1
33	init_ptc_mRNA	-5	-1.0583	+1	1	$+8.74 \cdot 10^{-02}$	1
34	init_shhPtch	-5	-0.7414	+0.5	1	$+1.81 \cdot 10^{-01}$	1
35	init_smo	-5	-0.0000	+0	1	$+1.00 \cdot 10^{+00}$	1
36	k1	-5	-1.6511	+1	1	$+2.23 \cdot 10^{-02}$	1
37	q0	-5	-4.4749	+1	1	$+3.35 \cdot 10^{-05}$	1
38	q1	-5	-0.3280	+1	1	$+4.70 \cdot 10^{-01}$	1
39	q2	-5	-2.3895	+1	1	$+4.08 \cdot 10^{-03}$	1
40	r0	-5	-4.9998	+2	1	$+1.00 \cdot 10^{-05}$	1
41	r1	-5	-0.0897	+1	1	$+8.13 \cdot 10^{-01}$	1
42	r2	-5	-4.9262	+1	1	$+1.19 \cdot 10^{-05}$	1
43	r3	-5	-4.8752	+1	1	$+1.33 \cdot 10^{-05}$	1
44	r4	-5	+0.9992	+1	1	$+9.98 \cdot 10^{+00}$	1
45	scale_dummy	-5	+0.3528	+1	1	$+2.25 \cdot 10^{+00}$	1
46	scale_gli11	-5	-0.0059	+1	1	$+9.87 \cdot 10^{-01}$	1
47	scale_gli12	-5	-0.0579	+1	1	$+8.75 \cdot 10^{-01}$	1
48	scale_gli13	-5	+0.0375	+1	1	$+1.09 \cdot 10^{+00}$	1
49	scale_gli1_clone03	-5	+0.1548	+1	1	$+1.43 \cdot 10^{+00}$	1

Table 24: Estimated parameter values
$\hat{\theta}$ indicates the estimated value of the parameters. θ_{min} and θ_{max} indicate the upper and lower bounds for the parameters. The log-column indicates if the value of a parameter was log-transformed. If log = 1 the non-log-column indicates the non-logarithmic value of the estimate. The fitted-column indicates if the parameter value was estimated (1), was temporarily fixed (0) or if its value was fixed to a constant value (2).

	name	θ_{min}	$\hat{\theta}$	θ_{max}	log	non-log $\hat{\theta}$	fitted
50	scale_gli1_clone04	-5	-1.7111	+1	1	$+1.94 \cdot 10^{-02}$	1
51	scale_gli1_clone06	-5	-1.0386	+1	1	$+9.15 \cdot 10^{-02}$	1
52	scale_gli1_clone07	-5	+0.0745	+1	1	$+1.19 \cdot 10^{+00}$	1
53	scale_gli1_clone08	-5	+0.0864	+1	1	$+1.22 \cdot 10^{+00}$	1
54	scale_gli1_ko	-5	+0.0038	+3	1	$+1.01 \cdot 10^{+00}$	1
55	scale_gli21	-5	+0.0430	+1	1	$+1.10 \cdot 10^{+00}$	1
56	scale_gli22	-5	+0.0466	+1	1	$+1.11 \cdot 10^{+00}$	1
57	scale_gli23	-5	+0.0072	+1	1	$+1.02 \cdot 10^{+00}$	1
58	scale_gli2_clone03	-5	+0.1220	+1	1	$+1.32 \cdot 10^{+00}$	1
59	scale_gli2_clone04	-5	-1.0828	+1	1	$+8.26 \cdot 10^{-02}$	1
60	scale_gli2_clone06	-5	-0.8851	+1	1	$+1.30 \cdot 10^{-01}$	1
61	scale_gli2_clone07	-5	+0.0207	+1	1	$+1.05 \cdot 10^{+00}$	1
62	scale_gli2_clone08	-5	+0.1492	+1	1	$+1.41 \cdot 10^{+00}$	1
63	scale_gli2_ko	-5	-0.0629	+1	1	$+8.65 \cdot 10^{-01}$	1
64	scale_gli3	-5	-0.4395	+1	1	$+3.63 \cdot 10^{-01}$	1
65	scale_gli31	-5	-0.5020	+1	1	$+3.15 \cdot 10^{-01}$	1
66	scale_gli3R1	-5	-0.5064	+1	1	$+3.12 \cdot 10^{-01}$	1
67	scale_mRNA	-5	+0.6602	+2	1	$+4.57 \cdot 10^{+00}$	1
68	scale_pifo1	-5	+0.3387	+2	1	$+2.18 \cdot 10^{+00}$	1
69	scale_pifo2	-5	+0.1679	+1	1	$+1.47 \cdot 10^{+00}$	1
70	scale_venus_pifo03	-5	-0.0969	+1	1	$+8.00 \cdot 10^{-01}$	1
71	scale_venus_pifo04	-5	-2.4359	+1	1	$+3.67 \cdot 10^{-03}$	1
72	scale_venus_pifo06	-5	-2.1612	+1	1	$+6.90 \cdot 10^{-03}$	1
73	scale_venus_pifo07	-5	-0.6420	+1	1	$+2.28 \cdot 10^{-01}$	1
74	scale_venus_pifo08	-5	-0.2028	+1	1	$+6.27 \cdot 10^{-01}$	1

Table 25: Estimated parameter values
$\hat{\theta}$ indicates the estimated value of the parameters. θ_{min} and θ_{max} indicate the upper and lower bounds for the parameters. The log-column indicates if the value of a parameter was log-transformed. If log = 1 the non-log-column indicates the non-logarithmic value of the estimate. The fitted-column indicates if the parameter value was estimated (1), was temporarily fixed (0) or if its value was fixed to a constant value (2).

	name	θ_{min}	$\hat{\theta}$	θ_{max}	log	non-log $\hat{\theta}$	fitted
75	sd_gli1_gli1A	-5	-1.1061	+1	1	$+7.83 \cdot 10^{-02}$	1
76	sd_gli1_gli1A_clone	-5	-0.6851	+1	1	$+2.06 \cdot 10^{-01}$	1
77	sd_gli1_gli1A_ko	-5	-0.4577	+1	1	$+3.49 \cdot 10^{-01}$	1
78	sd_gli2_gli2A	-5	-0.5941	+1	1	$+2.55 \cdot 10^{-01}$	1
79	sd_gli2_gli2A_clone	-5	-0.6094	+1	1	$+2.46 \cdot 10^{-01}$	1
80	sd_gli2_gli2A_ko	-5	-0.5030	+1	1	$+3.14 \cdot 10^{-01}$	1
81	sd_gli3	-5	-1.0532	+1	1	$+8.85 \cdot 10^{-02}$	1
82	sd_gli3R	-5	-0.6361	+1	1	$+2.31 \cdot 10^{-01}$	1
83	sd_mRNA	-5	-0.4157	+1	1	$+3.84 \cdot 10^{-01}$	1
84	sd_pifo	-5	-0.6869	+1	1	$+2.06 \cdot 10^{-01}$	1
85	sd_venus_pifo	-5	-0.6207	+1	1	$+2.39 \cdot 10^{-01}$	1
86	t1	-5	-0.4945	+1	1	$+3.20 \cdot 10^{-01}$	1
87	t2	-2e+01	-14.9694	-1e+01	1	$+1.07 \cdot 10^{-15}$	1
88	t3	-5	+1.8977	+3	1	$+7.90 \cdot 10^{+01}$	1
89	t4	-2e+01	-14.2343	-1e+01	1	$+5.83 \cdot 10^{-15}$	1
90	t5	-5	-2.9571	+1	1	$+1.10 \cdot 10^{-03}$	1
91	t6	-2e+01	-13.1750	-1e+01	1	$+6.68 \cdot 10^{-14}$	1

Table 26: Estimated parameter values

$\hat{\theta}$ indicates the estimated value of the parameters. θ_{min} and θ_{max} indicate the upper and lower bounds for the parameters. The log-column indicates if the value of a parameter was log-transformed. If log = 1 the non-log-column indicates the non-logarithmic value of the estimate. The fitted-column indicates if the parameter value was estimated (1), was temporarily fixed (0) or if its value was fixed to a constant value (2).

7 Profile likelihood of model parameters

In order to evaluate the identifiability of the model parameters and to assess confidence intervals the profile likelihood [2] was calculated. The mean calculation time of the profile likelihood per parameter was 00:03:10.81 ± 00:02:17.40. An overview is displayed in Figure 11 - 18.

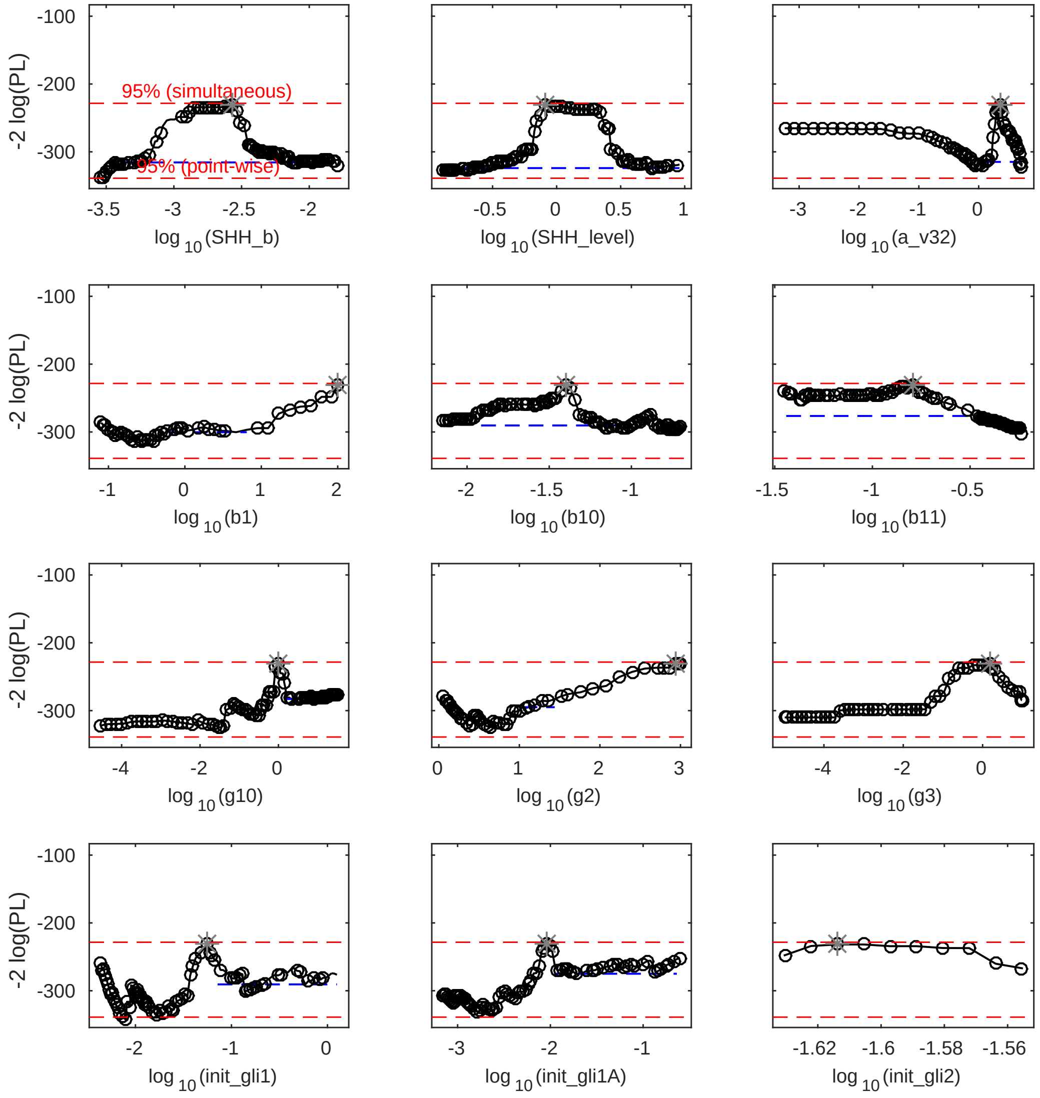

Figure 11: Overview of the profile likelihood of the model parameters
The solide lines indicate the profile likelihood. The broken lines indicate the threshold to assess confidence intervals. The asterisk indicate the optimal parameter values.

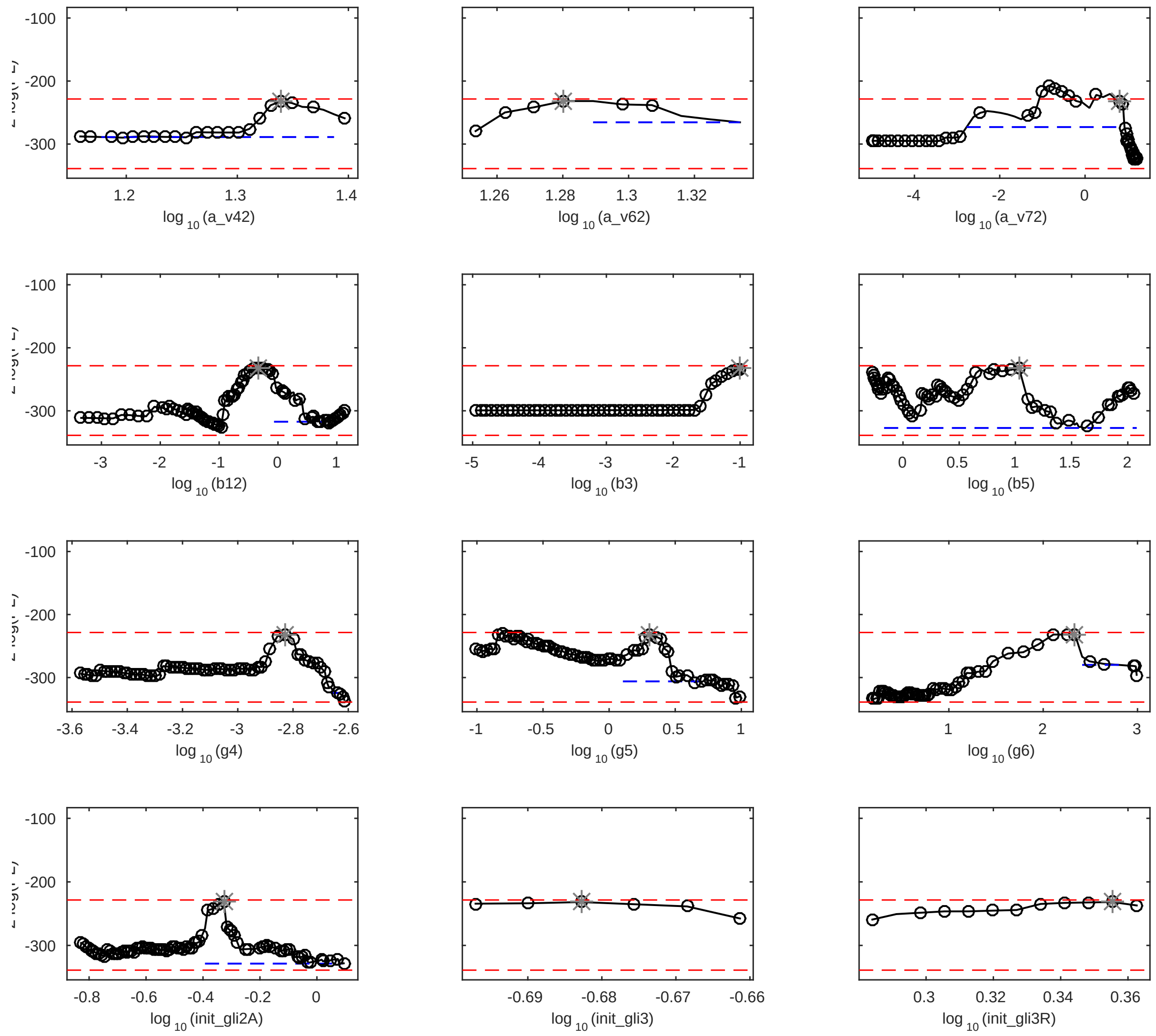

Figure 12: Overview of the profile likelihood of the model parameters
The solide lines indicate the profile likelihood. The broken lines indicate the threshold to assess confidence intervals. The asterisk indicate the optimal parameter values.

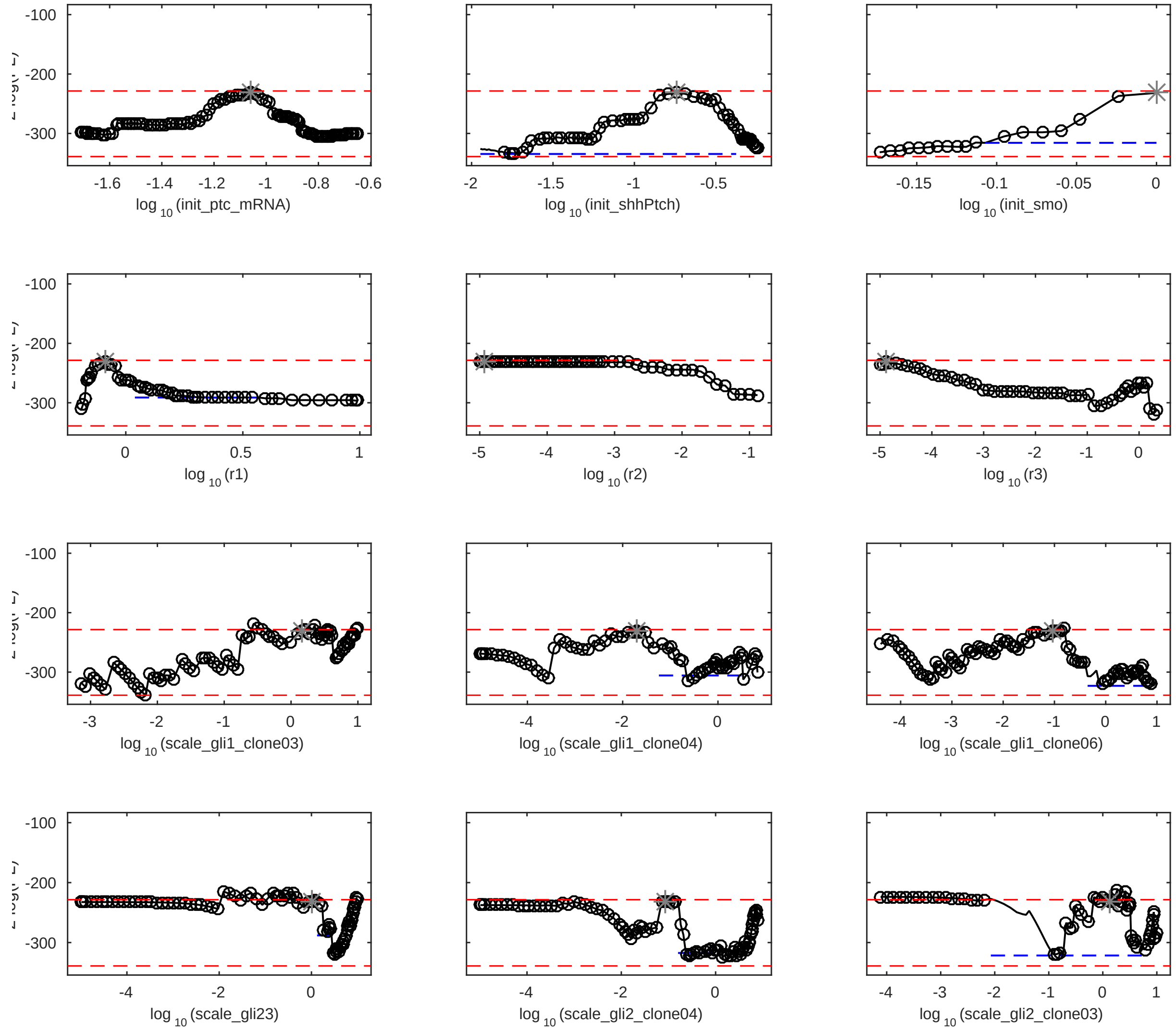

Figure 13: Overview of the profile likelihood of the model parameters
The solide lines indicate the profile likelihood. The broken lines indicate the threshold to assess confidence intervals. The asterisk indicate the optimal parameter values.

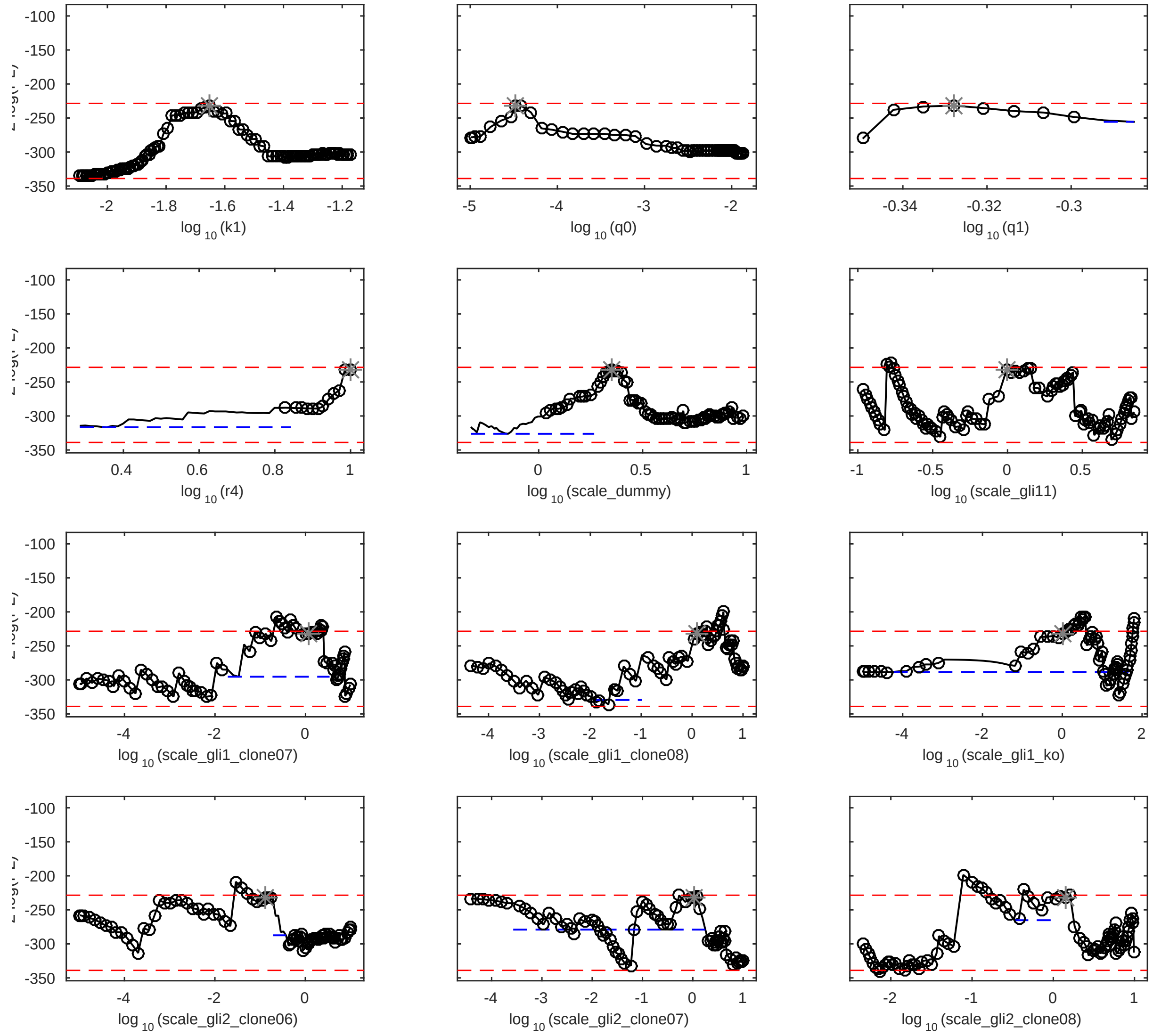

Figure 14: Overview of the profile likelihood of the model parameters
The solide lines indicate the profile likelihood. The broken lines indicate the threshold to assess confidence intervals. The asterisk indicate the optimal parameter values.

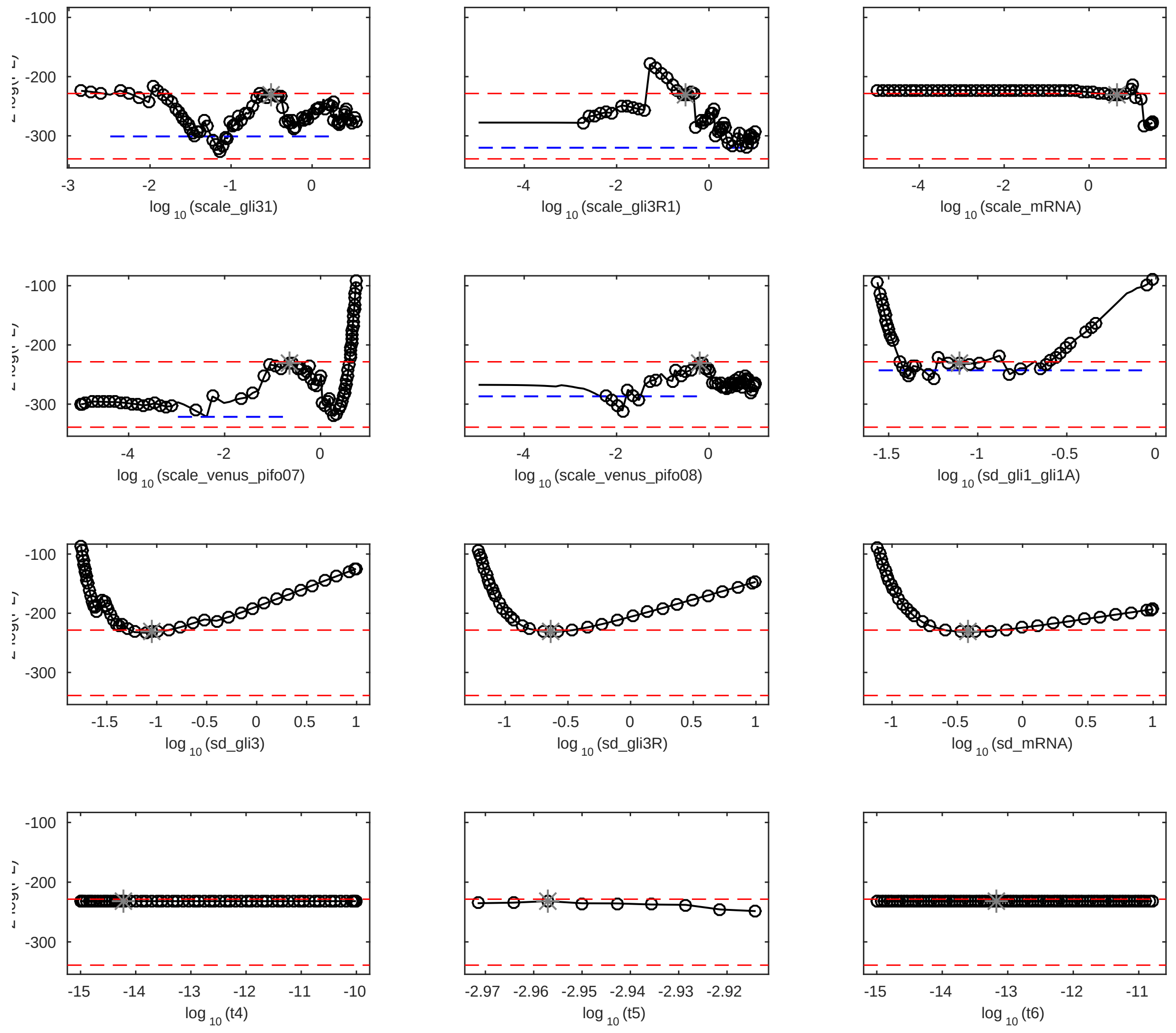

Figure 15: Overview of the profile likelihood of the model parameters
The solide lines indicate the profile likelihood. The broken lines indicate the threshold to assess confidence intervals. The asterisk indicate the optimal parameter values.

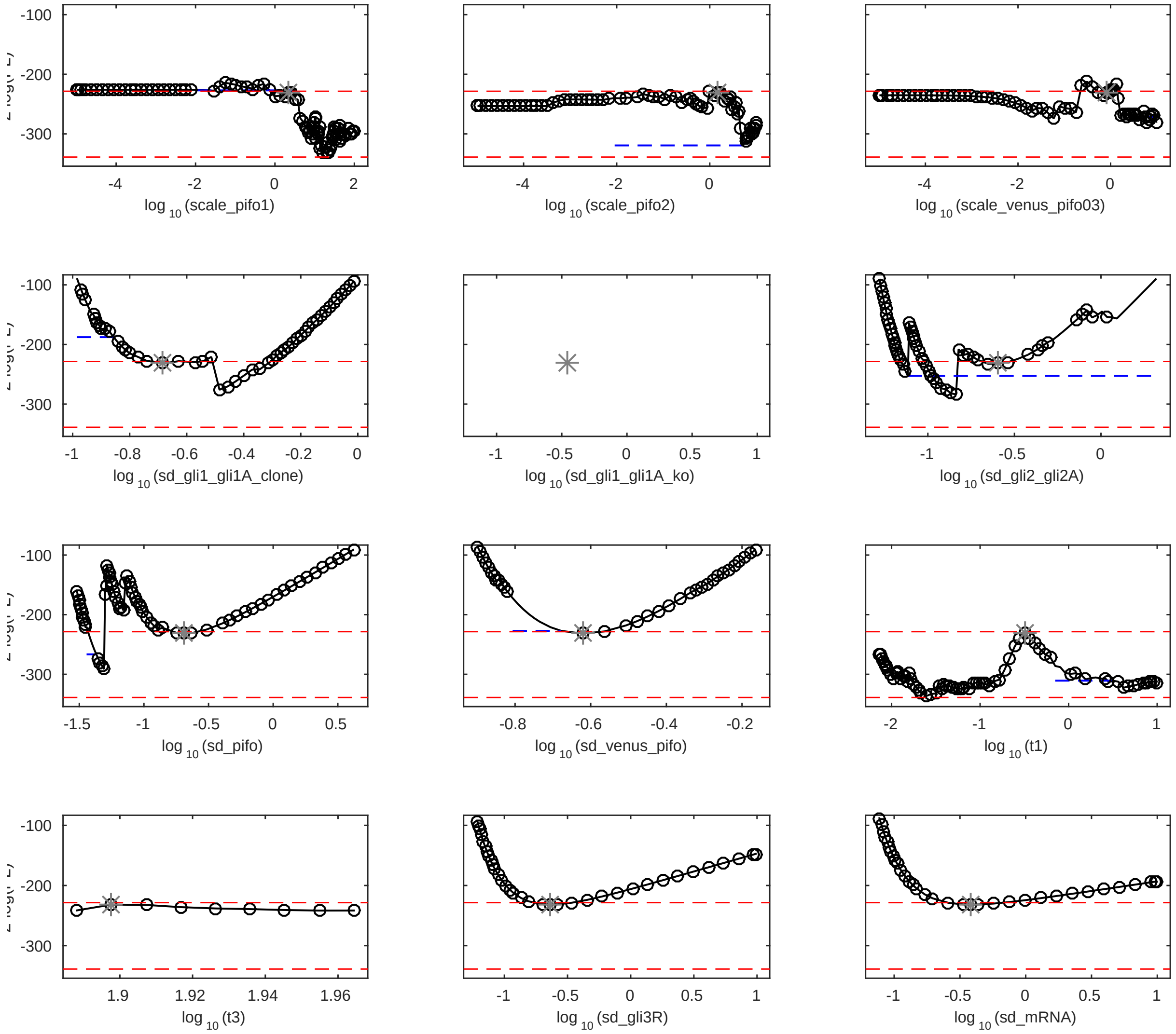

Figure 16: Overview of the profile likelihood of the model parameters
The solide lines indicate the profile likelihood. The broken lines indicate the threshold to assess confidence intervals. The asterisk indicate the optimal parameter values.

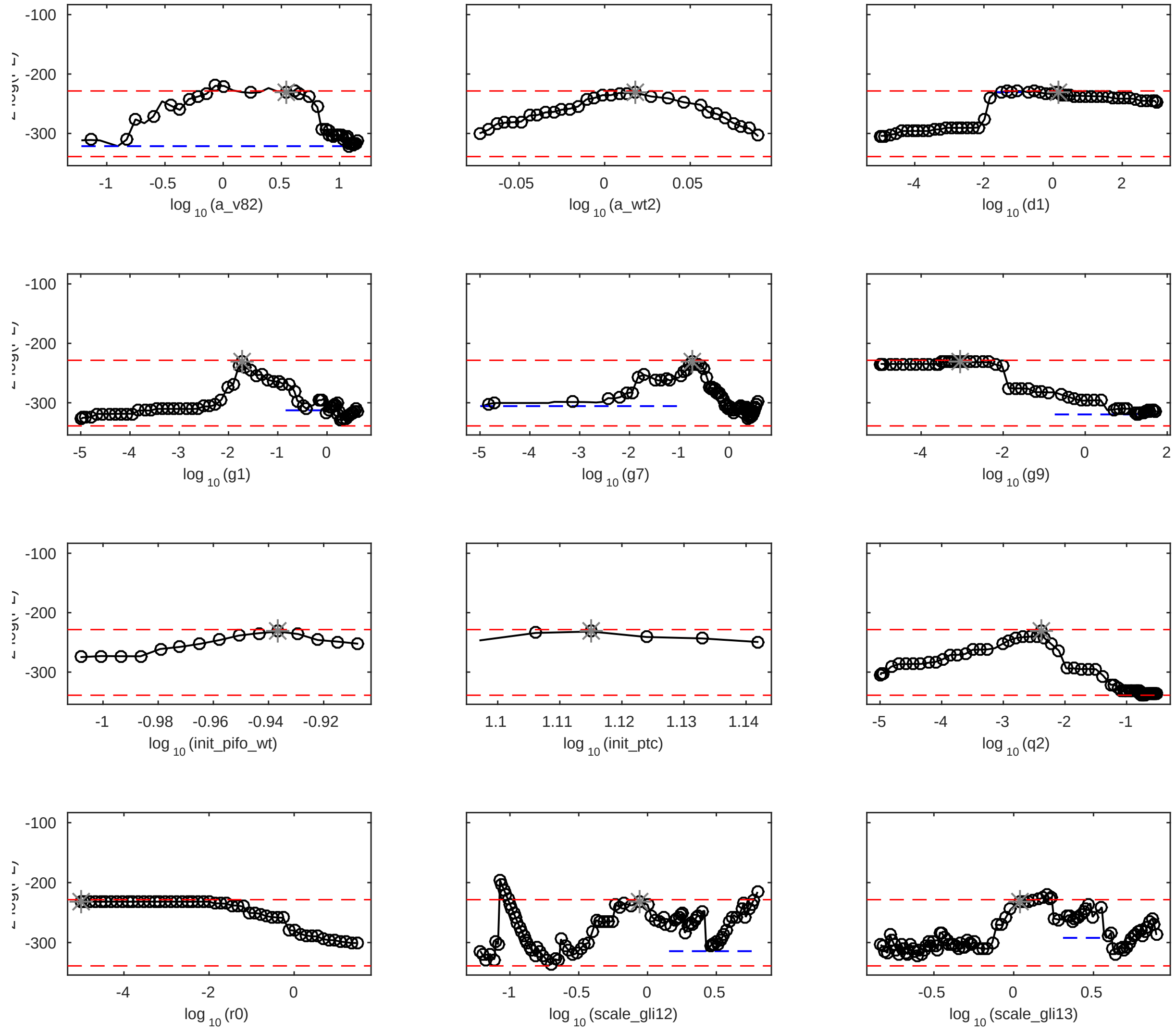

Figure 17: Overview of the profile likelihood of the model parameters
The solide lines indicate the profile likelihood. The broken lines indicate the threshold to assess confidence intervals. The asterisk indicate the optimal parameter values.

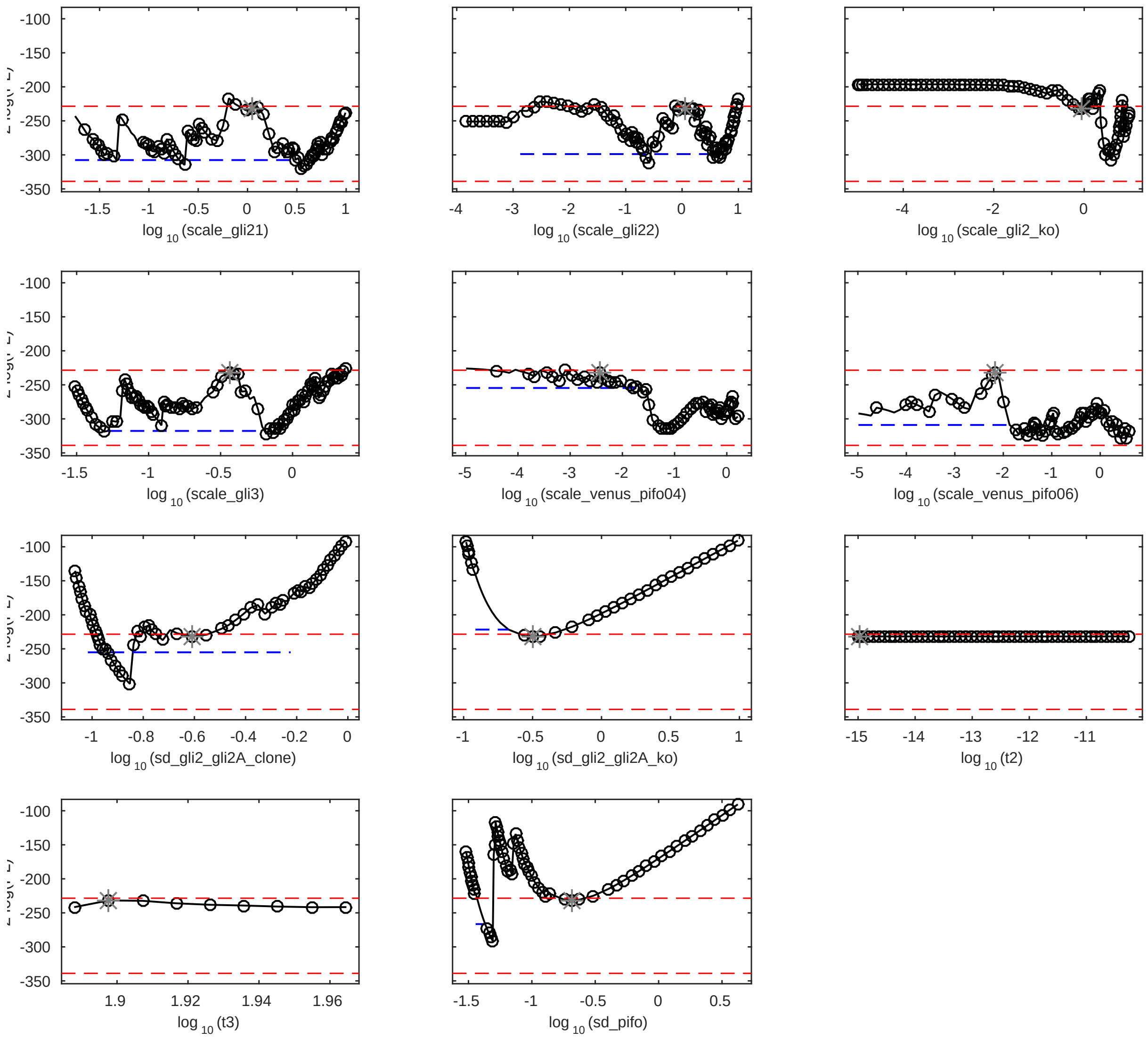

Figure 18: Overview of the profile likelihood of the model parameters
The solide lines indicate the profile likelihood. The broken lines indicate the threshold to assess confidence intervals. The asterisk indicate the optimal parameter values.

	name	$\hat{\theta}$	σ^-_{ptw}	σ^+_{ptw}	σ^-_{sim}	σ^+_{sim}
1	SHH_b	-2.567	-Inf	-3.514	-Inf	+Inf
2	SHH_level	-0.091	-Inf	-0.651	-Inf	+Inf
3	a_v32	+0.351	-0.070	+0.721	-Inf	+Inf
4	a_v42	+1.339	-Inf	+1.258	-Inf	+Inf
5	a_v62	+1.280	-Inf	+1.255	-Inf	+Inf
6	a_v72	+0.812	+1.139	+Inf	-Inf	+Inf
7	a_v82	+0.542	-0.966	+1.142	-Inf	+Inf
8	a_wt2	+0.018	+0.088	+Inf	-Inf	+Inf
9	b1	+1.990	-0.705	-0.401	-Inf	+Inf
10	b10	-1.400	-1.166	+Inf	-Inf	+Inf
11	b11	-0.791	-0.237	+Inf	-Inf	+Inf
12	b12	-0.334	-1.066	-0.961	-Inf	+Inf
13	b3	-1.002	-Inf	-1.628	-Inf	+Inf
14	b5	+1.032	+1.558	+1.646	-Inf	+Inf
15	d1	+0.162	-Inf	-4.525	-Inf	+Inf
16	g1	-1.733	-Inf	+0.350	-Inf	+Inf
17	g10	-0.010	-Inf	-1.402	-Inf	+Inf
18	g2	+2.940	+0.370	+0.656	-Inf	+Inf
19	g3	+0.170	-Inf	-3.677	-Inf	+Inf
20	g4	-2.827	-2.617	+Inf	-Inf	+Inf
21	g5	+0.304	+0.955	+Inf	-Inf	+Inf
22	g6	+2.337	-Inf	+0.519	-Inf	+Inf
23	g7	-0.731	+0.366	+0.430	-Inf	+Inf
24	g9	-3.036	+1.123	+1.431	-Inf	+Inf
25	init_gli1	-1.259	-2.129	-2.099	-Inf	+Inf
26	init_gli1A	-2.037	-2.819	-2.602	-Inf	+Inf
27	init_gli2	-1.614	-1.560	+Inf	-Inf	+Inf
28	init_gli2A	-0.326	-0.032	+Inf	-Inf	+Inf
29	init_gli3	-0.683	-0.663	+Inf	-Inf	+Inf

Table 27: Confidence intervals for the estimated parameter values derived by the profile likelihood $\hat{\theta}$ indicates the estimated optimal parameter value. σ^-_{ptw} and σ^+_{ptw} indicate 95% point-wise confidence intervals. σ^-_{sim} and σ^+_{sim} indicate 95% simultaneous confidence intervals.

8 Confidence intervals for the model parameters

In Table 27 – 30, 95% confidence intervals for the estimated parameter values derived by the profile likelihood [2] are given.

	name	$\hat{\theta}$	σ^{-}_{ptw}	σ^{+}_{ptw}	σ^{-}_{sim}	σ^{+}_{sim}
30	init_gli3R	+0.355	-Inf	+0.287	-Inf	+Inf
31	init_pifo_wt	-0.936	-Inf	-0.985	-Inf	+Inf
32	init_ptc	+1.115	-Inf	+Inf	-Inf	+Inf
33	init_ptc_mRNA	-1.058	-1.623	-0.699	-Inf	+Inf
34	init_shhPtch	-0.741	-1.833	-1.687	-Inf	+Inf
35	init_smo	-0.000	-Inf	-0.158	-Inf	+Inf
36	k1	-1.651	-Inf	-2.007	-Inf	+Inf
37	q0	-4.475	-1.962	+Inf	-Inf	+Inf
38	q1	-0.328	-Inf	-0.349	-Inf	+Inf
39	q2	-2.389	-0.783	+Inf	-Inf	+Inf
40	r0	-5.000	+0.994	+Inf	-Inf	+Inf
41	r1	-0.090	-Inf	-0.186	-Inf	+Inf
42	r2	-4.926	-1.257	+Inf	-Inf	+Inf
43	r3	-4.875	+0.249	+0.316	-Inf	+Inf
44	r4	+0.999	-Inf	+0.394	-Inf	+Inf
45	scale_dummy	+0.353	-0.303	-0.131	-Inf	+Inf
46	scale_gli11	-0.006	+0.693	+0.706	-Inf	+Inf
47	scale_gli12	-0.058	-0.718	-0.688	-Inf	+0.788
48	scale_gli13	+0.037	-0.721	+0.642	-Inf	+Inf
49	scale_gli1_clone03	+0.155	-2.216	-2.170	-Inf	+Inf
50	scale_gli1_clone04	-1.711	-0.648	+0.537	-Inf	+Inf
51	scale_gli1_clone06	-1.039	-0.126	+0.960	-Inf	+Inf
52	scale_gli1_clone07	+0.075	-2.966	+0.900	-Inf	+Inf
53	scale_gli1_clone08	+0.086	-1.892	-1.637	-Inf	+Inf
54	scale_gli1_ko	+0.004	+1.412	+1.454	-Inf	+Inf
55	scale_gli21	+0.043	+0.536	+0.564	-Inf	+Inf
56	scale_gli22	+0.047	-0.609	-0.575	-Inf	+Inf
57	scale_gli23	+0.007	+0.463	+0.516	-Inf	+Inf
58	scale_gli2_clone03	+0.122	-0.926	-0.731	-Inf	+Inf
59	scale_gli2_clone04	-1.083	-0.626	+0.444	-Inf	+Inf

Table 28: Confidence intervals for the estimated parameter values derived by the profile likelihood
$\hat{\theta}$ indicates the estimated optimal parameter value. σ^{-}_{ptw} and σ^{+}_{ptw} indicate 95% point-wise confidence intervals. σ^{-}_{sim} and σ^{+}_{sim} indicate 95% simultaneous confidence intervals.

	name	$\hat{\theta}$	σ^-_{ptw}	σ^+_{ptw}	σ^-_{sim}	σ^+_{sim}
60	scale_gli2_clone06	-0.885	-3.738	-3.684	-Inf	+Inf
61	scale_gli2_clone07	+0.021	-1.353	+0.804	-Inf	+Inf
62	scale_gli2_clone08	+0.149	-2.154	-1.821	-Inf	+Inf
63	scale_gli2_ko	-0.063	+0.588	+0.619	-Inf	+Inf
64	scale_gli3	-0.440	-1.313	-0.135	-Inf	+Inf
65	scale_gli31	-0.502	-1.152	-1.119	-Inf	+Inf
66	scale_gli3R1	-0.506	+0.451	+0.839	-Inf	+Inf
67	scale_mRNA	+0.660	+1.296	+1.429	-Inf	+Inf
68	scale_pifo1	+0.339	+1.197	+1.370	-Inf	+Inf
69	scale_pifo2	+0.168	+0.703	+0.748	-Inf	+Inf
70	scale_venus_pifo03	-0.097	+0.779	+Inf	-Inf	+Inf
71	scale_venus_pifo04	-2.436	-1.296	-1.024	-Inf	+Inf
72	scale_venus_pifo06	-2.161	-1.180	+0.549	-Inf	+Inf
73	scale_venus_pifo07	-0.642	-2.456	+0.319	-Inf	+0.628
74	scale_venus_pifo08	-0.203	-1.928	-1.861	-Inf	+Inf
75	sd_gli1_gli1A	-1.106	-1.394	-1.246	-1.527	-0.262
76	sd_gli1_gli1A_clone	-0.685	-0.487	-0.465	-0.915	-0.151
77	sd_gli1_gli1A_ko	-0.458	-0.551	-0.330	-1.085	+0.834
78	sd_gli2_gli2A	-0.594	-0.875	-0.836	-1.220	-0.183
79	sd_gli2_gli2A_clone	-0.609	-0.862	-0.851	-1.030	-0.290
80	sd_gli2_gli2A_ko	-0.503	-0.596	-0.375	-0.952	+0.749
81	sd_gli3	-1.053	-1.256	-0.904	-1.726	+Inf
82	sd_gli3R	-0.636	-0.772	-0.447	-1.181	+Inf
83	sd_mRNA	-0.416	-0.602	-0.143	-1.070	+Inf
84	sd_pifo	-0.687	-1.319	-1.308	-1.502	-0.058
85	sd_venus_pifo	-0.621	-0.668	-0.563	-0.874	-0.221
86	t1	-0.495	-1.649	-1.527	-Inf	+Inf
87	t2	-14.969	-Inf	+Inf	-Inf	+Inf
88	t3	+1.898	-Inf	+Inf	-Inf	+Inf
89	t4	-14.234	-Inf	+Inf	-Inf	+Inf

Table 29: Confidence intervals for the estimated parameter values derived by the profile likelihood $\hat{\theta}$ indicates the estimated optimal parameter value. σ^-_{ptw} and σ^+_{ptw} indicate 95% point-wise confidence intervals. σ^-_{sim} and σ^+_{sim} indicate 95% simultaneous confidence intervals.

	name	$\hat{\theta}$	σ^-_{ptw}	σ^+_{ptw}	σ^-_{sim}	σ^+_{sim}
90	t5	-2.957	-2.922	+Inf	-Inf	+Inf
91	t6	-13.175	-Inf	+Inf	-Inf	+Inf

Table 30: Confidence intervals for the estimated parameter values derived by the profile likelihood $\hat{\theta}$ indicates the estimated optimal parameter value. σ^-_{ptw} and σ^+_{ptw} indicate 95% point-wise confidence intervals. σ^-_{sim} and σ^+_{sim} indicate 95% simultaneous confidence intervals.

References

[1] A.C. Hindmarsh, P.N. Brown, K.E. Grant, S.L. Lee, R. Serban, D.E. Shumaker, and C.S. Woodward. Sundials: Suite of nonlinear and differential/algebraic equation solvers. *ACM Transactions on Mathematical Software (TOMS)*, 31(3):363–396, 2005.

[2] A. Raue, C. Kreutz, T. Maiwald, J. Bachmann, M. Schilling, U. Klingmüller, and J. Timmer. Structural and practical identifiability analysis of partially observed dynamical models by exploiting the profile likelihood. *Bioinformatics*, 25(15):1923–1929, Aug 2009.
