# Supplementary material for: Pitchfork and Gprasp2 Target Smoothened to the Primary Cilium for Hedgehog Pathway Activation
Source: PLoS One. 2016 Feb 22;11(2):e0149477. doi: 10.1371/journal.pone.0149477 (PMC4763541; doi:10.1371/journal.pone.0149477)

Protein Names	Gene Names	Uniprot	Ratio H/L Nc	Ratio H/L Nc	Intensity 16l	Peptides (se	Razor Peptic	Unique Pept
Protein pitchfork	PIFO	Q8TCI5	59,757	1,91E-21	5,83E+08	15	15	15
Mitochondrial import inner membrane transloca	TIMM8B;DDP	Q9Y5J9;B0YJA	9,124501	6,08E-08	2177500	4	4	4
Serine/threonine-protein phosphatase 2A 55 kD	PPP2R2A	P63151;B2RB	8,482399	4,97E-07	8,35E+07	19	19	12
Probable ATP-dependent RNA helicase DDX4;DE	DDX4;VASA	Q9NQI0-1;Q9	8,2539	6,85E-07	3771698	3	2	2
cDNA FLJ43800 fis, clone TESTI4000349, highly s	HECTD1;KIAA	B3KWT6;Q2K	6,7432	2,79E-06	176320,1	2	2	2
AH receptor-interacting protein;Aryl-hydrocarbc	AIP;XAP2	O00170;A0SZ	7,2332	3,06E-06	9443903	12	12	12
Lon protease homolog, mitochondrial;Lon prote	LONP1;PRSS1	P36776;B4DP	7,1946	3,25E-06	9,44E+07	39	39	39
STIP1 homology and U box-containing protein 1;	STUB1;CHIP;P	Q9UNE7-1;Q9	7,056601	4,01E-06	2,66E+07	16	16	16
Serine/threonine-protein phosphatase 2A 55 kD	PPP2R2D;KIA	Q66LE6;A8KA	5,7952	1,55E-05	1614101	9	2	2
Serine/threonine-protein phosphatase 2A cataly	PPP2CA;PPP2	P67775;B3KQ	5,950901	2,37E-05	3,41E+07	14	14	13
RING finger protein 219	RNF219;C13o	Q5W0B1;B2R	5,374401	3,45E-05	1357500	5	5	5
cDNA FLJ56382, highly similar to WD repeat prot	WDR6	B4DHK2;Q9N	5,7125	3,55E-05	2928200	9	9	9
Serine/threonine-protein phosphatase 2A 65 kD	PPP2R1A	P30153;A8K3	5,6348	4,06E-05	2,05E+08	25	25	19
Pyrroline-5-carboxylate reductase 3;Pyrroline-5-	PYCRL	Q53H96;B3K	5,539501	4,79E-05	7656197	10	10	10
Mitochondrial import inner membrane transloca	TIMM13;TIM1	Q9Y5L4	5,5114	5,03E-05	4818999	6	6	6
Mitochondrial import inner membrane transloca	TIMM8A;DDP	O60220;B2R5	4,965899	7,73E-05	906240	3	3	3
BAG family molecular chaperone regulator 2;Bcl	BAG2	O95816;B3K	5,1955	8,78E-05	4045700	7	7	7
Prefoldin subunit 5;C-myc-binding protein Mm-1	PFDN5;MM1;	Q99471;A8K9	4,8398	9,96E-05	1951699	4	4	4
Uncharacterized protein RCN2;Reticulocalbin-2;	RCN2;ERC55	A8MTG6;Q14	4,9864	0,000128	1,80E+07	9	9	9
HCLS1-associated protein X-1;HS1-associating pr	HAX1;HS1BP1	O00165;A8W	4,7551	0,0001958	3704400	6	6	6
SAPS domain family member 3;Sporulation-indu	SAPS3;C11orf	Q5H9R7-5;Q5	4,6065	0,0002585	1,30E+07	21	21	21
Prefoldin subunit 1	PFDN1;PFD1	O60925;B2RD	4,3511	0,0002739	2241299	5	5	5
A-kinase anchor protein 8-like;Neighbor of A-kin	AKAP8L;NAKA	Q9ULX6;B5BU	4,5612	0,0002815	7231301	7	7	7
Serine/threonine kinase 11-interacting protein;L	STK11IP;KIAA	Q8N1F8-1;Q8	4,1772	0,0003966	175070	2	2	2
PRO2620;Transcription factor 25;Nuclear localiz	TCF25;KIAA10	Q9H384;Q9B	4,1552	0,0004158	669420,1	4	4	4
CAD protein;Glutamine-dependent carbamoyl-p	CAD	P27708;Q53S	4,1219	0,0006566	8,21E+07	69	69	69
Prefoldin subunit 6;Protein Ke2	PFDN6;HKE2;	O15212;Q5ST	3,9413	0,0006611	793060,1	3	3	3
Nucleoporin NUP188 homolog	NUP188;KIAA	Q5SRE5-1;Q5	3,8766	0,0007619	874210,6	7	7	7
Sortilin;Neurotensin receptor 3;Glycoprotein 95;	SORT1	Q99523;A8KA	3,829	0,0008462	525929,9	2	2	2
Phosducin-like protein	PDCL	Q13371;Q4VX	3,8282	0,0008477	698510,1	3	3	3
Prefoldin subunit 3;Von Hippel-Lindau-binding p	VBP1;PFDN3	P61758;B2R8	3,9578	0,0009088	3676798	7	7	7
Prefoldin subunit 4;Protein C-1	PFDN4;PFD4	Q9NQP4;B2R	3,722	0,0010729	588030,4	4	4	4

ATP-dependent metalloprotease YME1L1;YME1-	YME1L1;FTSH	Q96TA2-1;Q9	3,6415	0,0012845	550259,9	3	3	3
DnaJ homolog subfamily A member 2;HIRA-inter	DNAJA2;CPR3	O60884;B2R7	3,7231	0,0014592	2,99E+07	12	12	12
Probable ubiquitin carboxyl-terminal hydrolase I	USP9X;DFFRX;	Q93008-2;Q9	3,6832	0,0015831	1,20E+07	50	50	50
G-protein coupled receptor-associated sorting p	GPRASP2	Q96D09;B3KV	3,4605	0,0019338	476669,8	2	2	2
Annexin A6;Annexin-6;Annexin VI;Lipocortin VI;p	ANXA6;ANX6	P08133;A8K3	3,3259	0,0026316	558240,3	3	3	3
Protein fem-1 homolog B;FEM1-beta;Fem-1-like	FEM1B;F1AA;I	Q9UK73	3,2983	0,0028044	588490	2	2	2
Phosphoglycerate mutase family member 5;Bcl-2	PGAM5	Q96HS1-1;Q9	3,3532	0,0031415	4085702	11	11	11
DnaJ homolog subfamily A member 3, mitochon	DNAJA3;HCA5	Q96EY1-1;Q9	3,3389	0,0032376	3807998	8	8	8
Insulin receptor substrate 4;160 kDa phosphotyr	IRS4	O14654	3,2835	0,0036401	3405701	10	10	10
Mitochondrial intermembrane space import and	CHCHD4;MIA4	Q8N4Q1-2;Q8	3,1756	0,0037267	980279,7	4	4	4
Caseinolytic peptidase B protein homolog;Suppr	CLPB;HSP78;S	Q9H078-2;Q9	3,1844	0,0044953	8464295	12	12	12
Putative eukaryotic translation initiation factor 3	KIAA0664	O75153	3,1155	0,0052112	1,37E+07	23	23	22
Proteasome assembly chaperone 4	PSMG4;C6orf	Q5JS54	3,0102	0,0054899	630899,9	3	3	3
Serine/threonine-protein phosphatase 6	PPP6C;PPP6	O00743;B2R5	3,0693	0,0057567	3684302	10	10	10
Prefoldin subunit 2	PFDN2;PFD2;I	Q9UHV9;B1A	2,9495	0,0063359	1763101	4	4	4
Methionine synthase;5-methyltetrahydrofolate-	MTR;RP11-18	Q99707;A1L4	2,9341	0,0065711	794499,9	3	3	3
Proteasome activator complex subunit 3;Protea	PSME3	P61289-2;P61	3,0018	0,0066626	1,89E+07	15	15	15
RNA polymerase II-associated protein 1	RPAP1;KIAA14	Q9BWH6-1;Q	2,9133	0,0069032	400809,8	3	3	3
T-complex protein 1 subunit gamma;CCT-gamma	CCT3;CCTG;TF	P49368;B4DU	2,9852	0,0069073	6,71E+08	40	40	40
T-complex protein 1 subunit epsilon;CCT-epsilon	CCT5;CCTE;KI	P48643;A8JZY	2,9837	0,0069299	5,28E+08	37	37	37
Tubulin beta-4 chain;Tubulin 5 beta;cDNA FLJ53	TUBB4;TUBB5	P04350;B3KQ	2,9577	0,0073334	2,11E+07	23	4	2
Apoptosis-inducing factor 1, mitochondrial;Prog	AIFM1;AIF;PD	O95831-1;O9	2,9329	0,0077409	3,28E+07	23	23	23
Suppressor of G2 allele of SKP1 homolog;Sgt1;P	SUGT1	Q9Y2Z0-1;Q9	2,9075	0,0081828	4762799	11	11	11
Zinc finger protein 294;RING finger protein 160	ZNF294;C21o	O94822;A7E2	2,836	0,0082959	761569,5	6	6	6
Calcium-binding mitochondrial carrier protein Ar	SLC25A12;AR	O75746;B3KM	2,8954	0,0084024	2940199	10	4	4
DnaJ homolog subfamily A member 1;Heat shoc	DNAJA1;DNAJ	P31689;Q5T7	2,8402	0,0094843	8,18E+07	14	14	14
T-complex protein 1 subunit beta;CCT-beta	CCT2;99D8.1;	P78371;A8K4	2,7636	0,0112297	6,43E+08	32	32	32
Mitochondrial-processing peptidase subunit alph	PMPCA;INPP5	Q10713;B4DK	2,6962	0,0115925	2108802	6	6	6
T-complex protein 1 subunit alpha;CCT-alpha	CCT1;CCTA;TC	P17987	2,7478	0,0116293	5,97E+08	34	34	34
Proteasome assembly chaperone 3	PSMG3;C7orf	Q9BT73;A8MI	2,6826	0,0119778	1047700	4	4	4
DNA-directed RNA polymerase II subunit RPB2;D	POLR2B	P30876;A8K1	2,7328	0,0120222	5806097	14	14	14
cDNA FLJ40895 fis, clone UTERU2002294, highly	PTGES3;hCG_	B3KUY2;Q151	2,7326	0,0120275	7095001	5	5	5
T-complex protein 1 subunit eta;CCT-eta;HIV-1 N	CCT7;CCTH;NI	Q99832;A8K7	2,7272	0,0121724	4,89E+08	30	30	30

Cell division protein kinase 4;Cyclin-dependent k	CDK4	P11802;B2R9A	2,6757	0,0121782	2353201	7	7	7
Tubulin beta chain;Tubulin beta-5 chain;cDNA FL	TUBB;TUBB5;(	P07437;B4DY	2,6923	0,013153	1,20E+09	24	24	4
T-complex protein 1 subunit delta;CCT-delta;Stir	CCT4;CCTD;SR	P50991;B2R6I	2,6562	0,0142533	6,42E+08	27	27	27
Endoribonuclease Dicer;Helicase with RNase mo	DICER1;DICER	Q9UPY3;A7E2	2,5704	0,0156996	217870,1	4	4	4
40S ribosomal protein S27;Metallopan-stimulin 1	RPS27;MPS1;I	P42677;Q5T4	2,595	0,0163404	6801503	5	5	3
T-complex protein 1 subunit zeta;CCT-zeta;CCT-z	CCT6A;CCT6;C	P40227;A1JUI	2,5824	0,0168077	5,20E+08	26	26	21
T-complex protein 1 subunit theta;CCT-theta;Re	CCT8;CCTQ;KI	P50990;B4DEI	2,5732	0,0171577	7,87E+08	34	34	34
cDNA FLJ16129 fis, clone BRACE2039823, highly	CDIPT;PIS;PIS1	B3KY94;O147	2,5145	0,0179749	373400,1	3	3	3
Fanconi anemia group I protein	FANCI;KIAA17	Q9NVI1-3;Q9I	2,5505	0,0180534	3124900	13	13	13
Mitochondrial import inner membrane transloca	TIMM9;TIM9;	Q9Y5J7;B2R58	2,5035	0,0184607	1002901	5	5	5
Tubulin alpha-1C chain;Tubulin alpha-6 chain;Alp	TUBA1C;TUBA	Q9BQE3;Q53C	2,5281	0,0189845	8239901	19	3	3
Serine palmitoyltransferase 1;Serine-palmitoyl-C	SPTLC1;LCB1	O15269;A8K6	2,4917	0,0189967	2156101	3	3	3
Ribonuclease inhibitor;Ribonuclease/angiogenin	RNH1;PRI;RNH	P13489;Q59G	2,4686	0,0200923	1253100	4	4	4
Heat shock cognate 71 kDa protein;Heat shock 7	HSPA8;HSC70	P11142-1;P11	2,4779	0,0212545	1,43E+09	30	30	23
Programmed cell death protein 5;TF-1 cell apopt	PDCD5;TFAR1	O14737;B4DE	2,4323	0,021945	975420,1	3	3	3
ADP-ribosylation factor 4	ARF4;ARF2	P18085;B2R7J	2,4245	0,0223652	957419,4	8	4	4
Mitochondrial dicarboxylate carrier;Solute carrie	SLC25A10;DIC	Q9UBX3-2;Q9	2,4084	0,0232585	1252400	8	8	8
UPF0556 protein C19orf10;Stromal cell-derived	C19orf10;IL25	Q969H8	2,3725	0,0253826	151140	3	3	3
Anaphase-promoting complex subunit 1;Cycloso	ANAPC1;TSG2	Q9H1A4	2,3654	0,0258254	730280,1	8	8	8
WD repeat-containing protein 68;WD repeat-co	WDR68;HAN1	P61962;B4DH	2,3383	0,029144	2773801	6	6	6
Cat eye syndrome critical region protein 5;Uncha	CECR5	Q9BXW7-1;Q9	2,3021	0,0301345	164440	2	2	2
DnaJ homolog subfamily C member 10;ER-reside	DNAJC10;ERD	Q8IXB1-1;Q8I	2,2813	0,0317032	523070,2	3	3	3
cDNA FLJ54705, highly similar to Serine/threonin	PPP2R4;RP11-	B4DZF8;A9IZU	2,2579	0,0335664	105250	2	2	2
DNA mismatch repair protein Msh6;MutS-alpha	MSH6;GTBP	P52701-1;P52	2,2585	0,034939	9301898	20	20	20
cDNA FLJ59750, highly similar to Ubiquitin-conju	UBE2L3;UBCE	B4DSZ4;P680	2,2303	0,0359059	151510	3	3	3
Fanconi anemia group D2 protein	FANCD2;FACD	Q9BXW9-1;Q9	2,2193	0,0368833	874729,7	3	3	3
Peptidyl-prolyl cis-trans isomerase A;Cyclophilin	PPIA;CYPA	P62937;A8K2	2,2182	0,0382977	2,44E+07	11	11	11
Calcium-binding mitochondrial carrier protein Ar	SLC25A13;ARA	Q9UJS0;Q53G	2,2166	0,0384376	1,67E+07	20	20	14
60 kDa heat shock protein, mitochondrial;Heat s	HSPD1;HSP60	P10809;B2R5I	2,195	0,0403779	4,37E+08	41	41	41
Serine/threonine-protein phosphatase 5;Protein	PPP5C;PPP5	P53041;B2R6I	2,1666	0,0419486	311079,8	2	2	2
cDNA FLJ77398, highly similar to Homo sapiens U	UGCGL1;GT;U	A8KAK1;Q9NY	2,17	0,0427476	4325003	17	17	17
Peptidyl-prolyl cis-trans isomerase;Peptidyl-prol	FKBP1A;FKBP1	Q0VDC6;P629	2,1581	0,0428283	199639,9	2	2	2
ADP-ribosylation factor 1;ADP-ribosylation facto	ARF1;ARF3;AF	P84077;P612	2,1552	0,0442163	4529301	10	10	6

60S ribosomal protein L31;RPL31 protein	RPL31	P62899;B2R4(	2,1217	0,0468085	825039,8	5	5	5
Nuclear pore complex protein Nup205;Nucleopo	NUP205;C7or	Q92621;A6H8	2,1144	0,04765	1837500	9	9	9
Tricarboxylate transport protein, mitochondrial;	SLC25A1;SLC2	P53007;A8K8I	2,1007	0,0492702	1109400	7	7	7
Heat shock 70 kDa protein 1;HSP70.1;HSP70-1/H	HSPA1A;HSPA	P08107;A8K5I	2,0669	0,0540986	8,58E+08	26	24	4
Serpin H1;Collagen-binding protein;47 kDa heat	SERPINH1;CBF	P50454;A8K2!	2,0657	0,0542472	4179000	7	7	7
Ubiquitin carboxyl-terminal hydrolase 11;Ubiqui	USP11;UHX1	P51784;B2RT)	2,0602	0,0543863	2156700	9	9	8
DnaJ homolog subfamily C member 7;Tetratrico	DNAJC7;TPR2,	Q99615;A8K4	2,0576	0,0547322	808650,3	2	2	2
Lactoylglutathione lyase;Methylglyoxalase;Aldol	GLO1	Q04760;B2R6	2,0383	0,0577535	5106601	11	11	11
Proliferation-associated protein 2G4;Cell cycle p	PA2G4;EBP1	Q9UQ80;A8K(	2,0339	0,0583374	1,22E+07	12	12	12
Exportin-T;tRNA exportin;Exportin(tRNA)	XPOT	O43592;A8KA	2,0299	0,0588732	1,66E+07	24	24	24
A kinase anchor protein 1, mitochondrial;Proteir	AKAP1;AKAP1	Q92667-1;Q9:	2,0168	0,0604534	438519,9	2	2	2
Splicing factor 3B subunit 3;Spliceosome-associa	SF3B3;KIAA00	Q15393-1;Q1!	2,0111	0,0614585	2,57E+07	27	27	27
Ferritin heavy chain;Cell proliferation-inducing g	FTH1;FTH;FTH	P02794;A9JQI	2,0082	0,0617326	1543700	3	3	3
DnaJ homolog subfamily B member 11;ER-assoc	DNAJB11;EDJ;	Q9UBS4;B3KV	2,0024	0,062693	6201403	8	8	8
Emerin;Emerin (Emery-Dreifuss muscular dystro	EMD;EDMD;S	P50402;Q6FI(	1,994	0,0639035	2368401	7	7	7
Putative ribosomal RNA methyltransferase 1;rRI	FTSJ1;JM23	Q9UET6;B2RC	1,9938	0,0639346	185249,9	2	2	2
Annexin A5;Annexin-5;Annexin V;Lipocortin V;Er	ANXA5;ANX5;	P08758;B4DN	1,9921	0,0641865	3902898	12	12	12
Cofilin-1;Cofilin, non-muscle isoform;18 kDa pho	CFL1;CFL	P23528;B3KU	1,9911	0,0643334	1,05E+07	10	10	10
Spermidine synthase;Putrescine aminopropyltra	SRM;SPS1;SRI	P19623;B1AKI	1,9838	0,065509	1307700	3	3	3
Phosphoribosylformylglycinamidine synthase;Fo	PFAS;KIAA036	O15067;A6H8	1,9801	0,0659714	8609195	15	15	15
Histidine triad nucleotide-binding protein 1;Ade	HINT1;HINT;P	P49773	1,9675	0,0681573	432280,2	5	5	5
Ubiquitin carboxyl-terminal hydrolase isozyme L	UCHL1;hCG_3	P09936;B2RD	1,9657	0,0684561	2072799	5	5	5
NEFA-interacting nuclear protein NIP30;RcNIP30	NIP30;CDA10;	Q9GZU8;Q6P	1,9655	0,0684894	450630	2	2	2
Melanoma-associated antigen D1;MAGE-D1 anti	MAGED1;NRA	Q9Y5V3-2;Q9	1,9606	0,0689777	2724899	4	4	3
NADH dehydrogenase [ubiquinone] 1 alpha subc	NDUFA4	O00483;A4D1	1,9463	0,0717593	760760,4	2	2	2
Presequence protease, mitochondrial;Pitrilysin r	PITRM1;KIAA1	Q5JRX3-2;Q5J	1,936	0,073576	112550	2	2	2
Phosducin-like protein 3;PhPL3;Viral IAP-associa	PDCL3;VIAF1	Q9H2J4;B2RA	1,9304	0,0745825	538590	3	3	3
Telomere length regulation protein TEL2 homolo	TELO2;KIAA06	Q9Y4R8;B4DF	1,9287	0,0748907	654579,8	3	3	3
Aldose reductase;Aldehyde reductase;Aldo-keto	AKR1B1;ALDR	P15121;B2R8I	1,9125	0,0769876	2566099	9	9	9
Peptidyl-prolyl cis-trans isomerase B;Cyclophilin	PPIB;CYPB	P23284	1,9146	0,0774953	1981301	8	8	8
Superoxide dismutase [Cu-Zn]	SOD1	P00441;A8MS	1,9132	0,0777586	741200,4	4	4	4
Adenine phosphoribosyltransferase	APRT	P07741	1,9093	0,078497	958550,4	5	5	5
Reticulocalbin-1	RCN1;RCN	Q15293;Q5J7	1,9054	0,0792421	1341299	3	3	3

Thyroid receptor-interacting protein 13;Thyroid	TRIP13	Q15645-1;Q1!	1,9029	0,0797234	621419,8	2	2	2
cDNA, FLJ92076, Homo sapiens likely ortholog o	HIGD1A;HIG1;	B2R4G1;Q9Y2	1,8956	0,081145	992580,1	2	2	2
Heterogeneous nuclear ribonucleoprotein F;Nuc	HNRNPF;HNRI	P52597;B3KM	1,8817	0,0825931	1,22E+07	11	9	9
Elongation factor Tu, mitochondrial;P43	TUFM	P49411	1,874	0,0840563	2,95E+07	21	21	21
Cytosolic non-specific dipeptidase;CNDP dipepti	CNDP2;CN2;C	Q96KP4;B3KU	1,874	0,0854974	441920	2	2	2
Destrin;Actin-depolymerizing factor;cDNA FLJ52	DSTN;ACTDP;I	P60981;B2R6I	1,8731	0,0856836	649439,7	3	3	3
60S ribosomal protein L23;Ribosomal protein L1	RPL23	P62829;Q9BT	1,8592	0,0869406	5318101	6	6	6
Profilin-1;Profilin I	PFN1	P07737;Q53Y	1,8559	0,0875969	1,29E+07	11	11	11
Protein DJ-1;Oncogene DJ1;Parkinson disease pr	PARK7	Q99497;B2R4	1,8526	0,0900323	1934401	10	10	10
Phosphate carrier protein, mitochondrial;Phospl	SLC25A3;PHC;	Q00325-2;Q0	1,8284	0,0932584	1,40E+07	13	13	13
Low molecular weight phosphotyrosine protein	ACP1	P24666-1;P24	1,8277	0,0956018	910070,1	3	3	3
Importin-4;Importin-4b;Ran-binding protein 4;IP	IPO4;IMP4B;R	Q8TEX9-2;Q8	1,8057	0,0982004	1,03E+07	19	19	19
Protein pelota homolog	PELO;CGI-17	Q9BRX2;B3KP	1,8138	0,0988538	219690	2	2	2
Transportin-3;Transportin-SR;Importin-12	TNPO3;IPO12	B5MD86;Q9Y!	1,7912	0,10149	3038499	11	11	11
HEAT repeat-containing protein 1;Protein BAP2	HEATR1;BAP2	Q9H583;A2VD	1,7917	0,104244	77981,02	2	2	2
60S ribosomal protein L38	RPL38	P63173;B2R5	1,7783	0,1045068	3509101	5	5	5
Mitochondrial 2-oxoglutarate/malate carrier pro	SLC25A11;SLC	Q02978;Q6IBI	1,7744	0,1054359	5180099	11	11	11
Hcp beta-lactamase-like protein C1orf163	C1orf163	Q96BR5	1,7792	0,1074163	440880,1	2	2	2
Ubiquitin-like modifier-activating enzyme 6;Ubic	UBA6;MOP4;U	A0AVT1-1;A0	1,7737	0,1088411	1435200	8	8	8
DNA-dependent protein kinase catalytic subunit	PRKDC;HYRC;I	P78527-1;P78	1,7516	0,11103	5,32E+07	145	145	145
Myotrophin;Protein V-1	MTPN	P58546;Q69Y	1,7637	0,111478	313469,8	3	3	3
Eukaryotic translation initiation factor 5A-1;Euka	EIF5A;EIF5AL1	P63241-2;P63	1,7421	0,1134448	4156002	8	8	8
cAMP-dependent protein kinase type I-alpha reg	PRKAR1A;PKR	P10644;B2R5	1,7557	0,1136312	2196801	5	5	5
Isoleucyl-tRNA synthetase, mitochondrial;Isoleu	IARS2	Q9NSE4;A8K5	1,7554	0,1137128	1108100	7	7	7
Prolyl 3-hydroxylase 1;Leucine- and proline-enri	LEPRE1;GROS	Q32P28-3;Q3	1,7533	0,1142849	288659,9	2	2	2
Nuclear pore complex protein Nup93;Nucleopor	NUP93;KIAA0	Q8N1F7;A8K8	1,7316	0,1161729	5983697	13	13	13
Nuclear pore complex protein Nup155;Nucleopo	NUP155;KIAA	O75694-1;O7!	1,7291	0,1168317	3342298	9	9	9
DnaJ homolog subfamily B member 6;Heat shocl	DNAJB6;HSJ2;	O75190-1;O7!	1,7433	0,1170471	964860,6	4	4	4
Platelet-activating factor acetylhydrolase IB sub	PAFAH1B2;PA	P68402;A8DP	1,7429	0,1171589	321129,8	2	2	2
Mitochondrial import inner membrane transloca	TIMM50;TIM5	Q3ZCQ8-2;Q3	1,7315	0,120388	2219000	6	6	6
E3 ubiquitin-protein ligase HUWE1;HECT, UBA a	HUWE1;KIAA	Q7Z6Z7-1;Q7	1,7104	0,1218751	1,06E+07	47	47	39
Heat shock protein 105 kDa;Heat shock 110 kDa	HSPH1;HSP10	Q92598-2;Q9	1,7096	0,1220955	7,26E+07	32	30	29
Cystatin-B;Stefin-B;Liver thiol proteinase inhibit	CSTB;CST6;STI	P04080;Q76L	1,7105	0,1265579	308010,2	3	3	3

Succinate dehydrogenase [ubiquinone] iron-sulf	SDHB;SDH;SD	P21912;B2R5	1,6863	0,1286842	2616499	5	5	5
Protein mago nashi homolog 2;Uncharacterized	MAGOHB;MA	Q96A72;A6NE	1,6957	0,1310841	349970,1	3	3	3
Glutathione S-transferase P;GST class-pi;GSTP1-1	GSTP1;FAEES3	P09211;A8MX	1,6776	0,1312306	5586101	9	9	9
Exosome complex exonuclease RRP44;Ribosoma	DIS3;KIAA100	Q9Y2L1-1;Q9Y	1,6767	0,1314967	5718202	22	22	22
Glutathione transferase omega-1;GSTO 1-1;Glut	GSTO1;GSTTL	P78417;B2R98	1,6928	0,1319886	715100,4	6	6	6
Transgelin-2;SM22-alpha homolog	TAGLN2;KIAA(	P37802;Q6FG	1,6733	0,1325069	2671101	9	9	9
Mitotic spindle assembly checkpoint protein MA	MAD2L1;MAD	Q13257;Q53F	1,6905	0,1327101	974989,5	4	4	4
ADP/ATP translocase 2;Adenine nucleotide trans	SLC25A5;ANT2	P05141;B2RC	1,6718	0,1329549	1,14E+08	18	18	7
DNA damage-binding protein 1;Damage-specific	DDB1;XAP1	Q16531;B2R6	1,67	0,1334944	4,97E+07	37	37	37
Tyrosine-protein kinase Lyn;Fibroblast growth fa	LYN;FGFR3;FG	P07948-1;P07	1,6865	0,1339739	1479100	2	2	2
Thioredoxin;ATL-derived factor;Surface-associat	TXN;TRDX;TR	P10599;Q9UD	1,6675	0,1342472	8542405	6	6	6
Ribosomal protein L10;60S ribosomal protein L1	RPL10;XX-FW8	Q5HY50;P276	1,6779	0,1367291	564760,2	5	5	5
Acylglycerol kinase, mitochondrial;Multiple subs	AGK;MULK	Q53H12-1;Q5	1,6528	0,138756	6697397	4	4	4
Vinculin;Metavinculin	VCL	P18206-1;P18	1,6497	0,139725	1,61E+07	26	26	26
cDNA FLJ76284, highly similar to Homo sapiens s	SUCLG1	A8K4W7;B2R8	1,6621	0,1419296	745890,4	5	5	5
Spliceosome RNA helicase BAT1;DEAD box prote	BAT1;UAP56;	Q13838-2;Q1	1,6422	0,1420961	1,93E+07	14	14	5
Chloride intracellular channel protein 1;Nuclear	CLIC1;NCC27	O00299;Q53F	1,6554	0,14419	629510,2	7	7	6
S-formylglutathione hydrolase;Esterase D	ESD	P10768	1,6549	0,1443601	1485601	7	7	7
Succinyl-CoA ligase [ADP-forming] subunit beta,	SUCLA2;RP11-	Q9P2R7-1;Q9	1,6545	0,1444962	2138200	3	3	3
Uncharacterized protein ENSP00000348237;Pho	PGAM1;PGAM	A6NJ80;P1866	1,6257	0,1474475	8941804	12	12	12
Chromosome 1 open reading frame 57 (Chromo	C1orf57;RP4-6	Q5TDF0;Q9BS	1,6441	0,1480785	544610,4	4	4	4
Mitochondrial import inner membrane transloca	TIMM23;TIM2	O14925;Q5SR	1,6426	0,1486019	513889,9	3	3	3
Peroxiredoxin-5, mitochondrial;Prx-V;Peroxisom	PRDX5;ACR1;S	P30044-1;P30	1,6378	0,1502885	232309,9	5	5	5
Arsenite-resistance protein 2	ARS2;ASR2	Q9BXP5-1;Q9	1,6146	0,1511542	5203897	13	13	13
Phosphatidylethanolamine-binding protein 1;Pro	PEBP1;PBP;PE	P30086;B2R4S	1,6284	0,1536427	758290,3	8	8	8
Mitochondrial import receptor subunit TOM70;M	TOMM70A;KIA	O94826;B3KQ	1,6049	0,154465	2870699	5	5	5
E3 ubiquitin-protein ligase UBR5;E3 ubiquitin-pro	UBR5;EDD;ED	O95071;B2RP	1,6221	0,1559293	2008399	2	2	2
Leukotriene A-4 hydrolase;Leukotriene A(4) hyd	LTA4H;LTA4	P09960-1;P09	1,6005	0,1559892	3631498	10	10	10
Sodium/potassium-transporting ATPase subunit	ATP1A1	P05023-1;P05	1,5982	0,1567916	6,81E+07	32	32	28
Triosephosphate isomerase;Triose-phosphate is	TPI1;TPI	P60174-1;P60	1,5977	0,1569665	1,62E+07	14	14	14
Aspartate aminotransferase, mitochondrial;mAs	GOT2	P00505;A8K48	1,5929	0,1586553	1,01E+07	12	12	12
Thioredoxin domain-containing protein 17;Thior	TXNDC17;TXN	Q9BRA2;A8K7	1,6147	0,1586553	65508,02	2	2	2
10 kDa heat shock protein, mitochondrial;10 kDa	HSPE1	P61604;Q53X	1,5897	0,1597905	2,06E+07	8	8	8

Brain protein 16;cDNA FLJ40907 fis, clone UTERU	BRP16;C8orf3	Q9BTY7;B3KU	1,6116	0,1598102	331430	3	3	3
Adenylate kinase isoenzyme 2, mitochondrial;AT	AK2;ADK2	P54819-1;P54	1,6035	0,1628645	2003999	11	11	11
Ras-related C3 botulinum toxin substrate 1;p21-	RAC1;MIG5;h	P63000-2;P63	1,6016	0,1635886	897680,6	4	4	4
BolA-like protein 2	BOLA2;BOLA2	Q9H3K6-1;Q9	1,5957	0,1658561	198449,9	4	4	2
Importin-9;Ran-binding protein 9	IPO9;IMP9;KI	Q96P70;B1AS	1,5674	0,1679155	7564798	11	11	11
Carbonic anhydrase 2;Carbonic anhydrase II;Carl	CA2	P00918;B2R7	1,5891	0,1684268	1750600	7	7	7
Kinesin-like protein KIF1A;Axonal transporter of	KIF1A;ATSV;C	Q12756-2;Q1	1,5865	0,1694494	1030700	5	5	4
Splicing factor, arginine/serine-rich 1;pre-mRNA-	SFRS1;ASF;SF	Q07955-1;Q0	1,5852	0,1699628	2200001	8	8	8
Mitogen-activated protein kinase 1;Extracellular	MAPK1;ERK2;	P28482;B4DH	1,5596	0,1708471	2562400	7	7	6
Elongation factor 2;cDNA FLJ56548, highly simila	EEF2;EF2	P13639;B2RM	1,5575	0,1716445	3,32E+08	42	42	41
Malate dehydrogenase, cytoplasmic;Cytosolic m	MDH1;MDHA	P40925;B2R5	1,5521	0,1737108	8464295	10	10	10
Interleukin enhancer-binding factor 3;Nuclear fa	ILF3;DRBF;MP	Q12906-4;Q1	1,5738	0,1745263	643699,6	3	3	3
3'(2'),5'-bisphosphate nucleotidase 1;Bisphospha	BPNT1	O95861-2;O9	1,5705	0,175868	344430,2	2	2	2
Phosphoglycerate kinase 1;Primer recognition p	PGK1;PGKA;M	P00558;A8K4	1,5456	0,1762285	3,52E+07	18	18	18
Ubiquitin-conjugating enzyme E2 K;Ubiquitin-co	UBE2K;HIP2;L	P61086-1;P61	1,5686	0,1766447	223580	2	2	2
Carbonyl reductase [NADPH] 1;NADPH-depende	CBR1;CBR;CR	P16152;B2RB	1,5663	0,1775891	485570,2	6	6	6
Tumor protein, translationally-controlled 1;Tran	TPT1;RP11-29	Q5W0H4;P13	1,5609	0,1798244	456510,1	4	4	2
Puromycin-sensitive aminopeptidase;cDNA FLJ3	NPEPPS;PSA	P55786;B3KU	1,5321	0,181566	1,29E+07	20	20	20
Stromal cell-derived factor 2-like protein 1;PWP	SDF2L1;UNQ1	Q9HCN8;A2RU	1,556	0,1818747	1393900	5	5	4
Testin;TESS	TES	Q9UGI8-1;Q9	1,5546	0,1824644	449320	3	3	3
60S ribosomal protein L22;Epstein-Barr virus sm	RPL22	P35268;B2R4	1,5535	0,1829289	1927801	3	3	3
Glutaredoxin-3;Thioredoxin-like protein 2;PKC-ir	GLRX3;PICOT;	O76003;B3KM	1,5245	0,1846359	2712499	6	6	6
Obg-like ATPase 1;GTP-binding protein 9;cDNA F	OLA1;GTPBP9	Q9NTK5-1;Q9	1,5232	0,1851658	5529903	8	8	8
Deoxyuridine 5'-triphosphate nucleotidohydrola	DUT	P33316-1;P33	1,5464	0,1859529	296070,1	4	4	4
Mitochondrial glutamate carrier 1;Glutamate/H(	SLC25A22;GC	Q9H936;A8K3	1,543	0,1874169	1528501	4	4	4
40S ribosomal protein S27a;cDNA FLJ75516, higl	RPS27A;UBA8	P62979;B2RD	1,5138	0,189039	1,05E+08	8	8	8
Ubiquitin-conjugating enzyme E2 N;Ubiquitin-pr	UBE2N;BLU;U	P61088;A8MU	1,5388	0,1892397	286020,2	4	4	4
Pre-mRNA-splicing factor ATP-dependent RNA h	DHX38;DDX38	Q92620;A8K6	1,5358	0,1905514	217809,9	2	2	2
Up-regulated during skeletal muscle growth pro	USMG5;HCVF	Q96IX5;B2R4	1,5357	0,1905953	1664699	2	2	2
15 kDa selenoprotein	Sep 15	O60613-1;O6	1,5347	0,1910344	193440,1	2	2	2
Mitochondrial import receptor subunit TOM20 h	TOMM20;KIA	Q15388	1,5331	0,191739	101740	2	2	2
40 kDa peptidyl-prolyl cis-trans isomerase;Cyclo	PPID;CYP40;C	Q08752;B2R9	1,5325	0,1920037	385719,8	3	3	3
Transmembrane protein 33;DB83 protein	TMEM33;DB8	P57088;A6QK	1,5298	0,1931993	448179,7	3	3	3

ADP/ATP translocase 3;Adenine nucleotide trans	SLC25A6;ANT3	P12236;Q59E	1,5035	0,1933682	7303496	14	6	4
Stathmin;Phosphoprotein p19;Leukemia-associa	STMN1;LAP18	P16949;B2R4I	1,5294	0,1933771	1537099	4	4	4
FACT complex subunit SPT16;Facilitates chromat	SUPT16H;FACT	Q9Y5B9;Q0VG	1,5012	0,1943472	3,02E+07	25	25	25
Protein VPRBP;HIV-1 Vpr-binding protein;Vpr-int	VPRBP;DCAF1	Q9Y4B6-1;Q9Y	1,5258	0,1949829	811130,1	7	7	7
Nicotinamide phosphoribosyltransferase;Pre-B-c	NAMPT;PBEF;	P43490-1;P43	1,5249	0,1953862	676379,6	2	2	2
Poly(rC)-binding protein 1;Alpha-CP1;Nucleic aci	PCBP1	Q15365;Q53S	1,4939	0,1974844	8737795	9	6	6
ATP-dependent RNA helicase A;Nuclear DNA hel	DHX9;DDX9;LI	Q08211;B2RN	1,4924	0,1981347	3084902	8	8	8
Angio-associated migratory cell protein	AAMP	Q13685;Q59E	1,5184	0,1983212	288840,1	2	2	2
cDNA FLJ75459;Nucleolysin TIAR;TIA-1-related p	TIAL1;hCG_40	A8K4L9;A8K5(	1,5169	0,1990041	1364499	4	4	4
Growth factor receptor-bound protein 2;Adapte	GRB2;ASH	P62993-1;P62	1,5121	0,2012034	739379,9	4	4	4
Ribose-phosphate pyrophosphokinase 1;Phosph	PRPS1;RP11-5	P60891;B2R6T	1,4833	0,2021219	1,05E+07	10	10	3
Phospholipase C, gamma 1 (Phospholipase C, ga	PLCG1;RP3-51	A2A284;B4DM	1,505	0,204496	510359,7	3	3	3
2',3'-cyclic-nucleotide 3'-phosphodiesterase;cDN	CNP	P09543-1;P09	1,5013	0,2062308	735700	3	3	3
Malate dehydrogenase, mitochondrial	MDH2	P40926;B2RE7	1,4739	0,2063168	2,21E+07	14	14	14
RNA-binding protein 8A;RNA-binding motif prote	RBM8A;RBM8	Q9Y5S9-1;Q9Y	1,4985	0,2075522	222619,9	2	2	2
cDNA FLJ77432, highly similar to Homo sapiens p	PCBP2;hCG_2	A8K7X6;Q59H	1,4708	0,2077173	1,49E+07	12	12	9
Protein phosphatase 1G;Protein phosphatase 2C	PPM1G;PPM1	O15355;B2R6	1,4698	0,208171	6639001	9	9	9
SAPK substrate protein 1;UBA/UBX 33.3 kDa pro	SAKS1	Q04323-2;Q04	1,4966	0,2084531	202839,9	2	2	2
Ubiquitin-conjugating enzyme E2 O;Ubiquitin-pr	UBE2O;KIAA1	Q9C0C9	1,4942	0,2095961	2277601	9	9	9
Protein TRS85 homolog	KIAA1012	Q9Y2L5-1;Q9Y	1,4935	0,2099305	157139,9	2	2	2
Dolichyl-phosphate mannosyltransferase polype	DPM1;RP5-91	Q5QPK0;Q5Q	1,4927	0,2103133	1535700	6	6	6
Coproporphyrinogen III oxidase, mitochondrial	CPOX;CPO;CP.	P36551;A8K2	1,4927	0,2103133	717550,4	3	3	3
Glyoxylate reductase/hydroxypyruvate reductas	GRHPR;RP11-3	Q5T946;Q5M	1,4919	0,2106966	360470,2	2	2	2
S-phase kinase-associated protein 1;Cyclin A/CD	SKP1;EMC19;(	P63208-1;P63	1,4833	0,2148568	1759101	3	3	3
Rab GDP dissociation inhibitor beta;Guanosine d	GDI2;RABGDII	P50395;B4DLY	1,4543	0,2153172	1,82E+07	22	22	15
Sarcoplasmic/endoplasmic reticulum calcium AT	ATP2A2;ATP2	P16615-1;P16	1,4532	0,2158326	1,64E+07	22	22	22
Platelet-activating factor acetylhydrolase IB subu	PAFAH1B3;PA	Q15102;Q53X	1,4805	0,2162268	450759,9	3	3	3
Nardilysin (N-arginine dibasic convertase) (Nardi	NRD1;RP4-65	B1AKJ5;Q6UU	1,4792	0,2168655	1266401	7	7	7
Aconitase 2, mitochondrial;Aconitate hydratase,	ACO2;RP3-34	A2A274;Q997	1,4503	0,2171968	2791600	8	8	8
Transcription elongation factor SPT5;DRB sensiti	SUPT5H;SPT5;	O00267-1;O0(	1,4733	0,2197848	975590,4	5	5	5
Phosphatidylinositol transfer protein beta isofor	PITPNB;RP3-3	P48739-2;P48	1,4428	0,2207606	2451001	6	6	6
Ubiquitin carboxyl-terminal hydrolase isozyme L	UCHL3;RP11-1	P15374;B2R9	1,4713	0,2207821	415999,9	3	3	3
Rho GDP-dissociation inhibitor 1;Rho-GDI alpha;	ARHGDIA;GDI	P52565;B2R5	1,4707	0,2210821	2222899	6	6	6

Protein disulfide-isomerase A3;Disulfide isomera	PDIA3;ERP57;	P30101;B3KQ	1,4416	0,2213357	1,13E+07	15	15	12
Regulator of nonsense transcripts 1;ATP-depend	UPF1;KIAA022	Q92900-1;Q9	1,4414	0,2214316	3079698	10	10	10
Delta-1-pyrroline-5-carboxylate synthetase;Alde	ALDH18A1;GS	P54886-1;P54	1,4392	0,2224897	1,32E+07	16	16	16
5'-nucleotidase domain-containing protein 2	NT5DC2	Q9H857-2;Q9	1,4388	0,2226826	2,35E+07	2	2	2
Eukaryotic peptide chain release factor subunit 1	ETF1;ERF1;RF1	P62495;B2R6I	1,4375	0,2233104	3788500	8	8	8
CDNA FLJ25678 fis, clone TST04067, highly simil	NP;PNP	Q8N7G1;P004	1,4341	0,2249599	2984899	14	14	14
Serine/threonine protein phosphatase;Serine/th	PPP3CB;hCG_	Q8N1F0;P162	1,4613	0,2258284	1514799	2	2	2
Hexokinase-2;Hexokinase type II;Muscle form he	HK2	P52789;A8K2	1,4608	0,2260833	1789799	9	6	6
Programmed cell death protein 6;Apoptosis-link	PDCD6;ALG2	O75340;B2RD	1,4603	0,2263385	511589,9	3	3	3
Calumenin;Crocalbin;IEF SSP 9302	CALU	O43852-1;O4	1,4578	0,2276181	1365001	2	2	2
GTP-binding protein SAR1a;COPII-associated sm	SAR1A;SAR1;S	Q9NR31;B2R6	1,4545	0,2293169	1512300	3	3	3
Transforming protein RhoA;H12;Rho-related GTI	RHOA;ARH12;	P61586;B4DK	1,4245	0,229676	2391101	6	6	6
Heat shock protein HSP 90-alpha;HSP 86;Renal c	HSP90AA1;HS	P07900-2;P07	1,4239	0,2299736	8,73E+08	45	45	25
Electron transfer flavoprotein subunit beta	ETFB;FP585	P38117-2;P38	1,452	0,2306111	1108600	9	9	9
ATPase family AAA domain-containing protein 3	ATAD3A;RP5-	Q9NVI7-1;Q9	1,4506	0,2313386	658550,1	5	2	2
Ribosome maturation protein SBDS;Shwachman	SBDS;CGI-97	Q9Y3A5;A8K0	1,4492	0,2320681	262979,9	3	3	3
Peroxiredoxin-6;Antioxidant protein 2;1-Cys per	PRDX6;AOP2;	P30041;A4UC	1,4186	0,2326176	1,17E+07	15	15	15
Guanine nucleotide-binding protein G(I)/G(S)/G(	GNB2;GNB1;R	P62879;B3KP	1,4416	0,236063	1598300	2	2	2
WD repeat-containing protein 44;Rabphilin-11;V	WDR44;RP13-	Q5JSH3-1;Q5J	1,4409	0,2364339	352490,1	2	2	2
Thymidylate kinase;dTMP kinase	DTYMK;CDC8;	P23919;Q53F	1,4406	0,2365929	224709,9	3	3	3
Ubiquitin-like modifier-activating enzyme 1;Ubic	UBA1;A1S9T;	P22314;B4DD	1,4098	0,2370669	9,47E+07	32	32	32
FK506-binding protein 4;Peptidyl-prolyl cis-trans	FKBP4	Q02790;B2R9	1,4084	0,2377816	2,54E+07	18	18	18
cDNA FLJ46359 fis, clone TESTI4049786, highly s	HK1	B3KXY9;P193	1,4083	0,2378327	3847699	11	11	8
Proliferating cell nuclear antigen;Cyclin	PCNA	P12004;B2R8	1,4074	0,2382933	1,68E+07	12	12	12
Barrier-to-autointegration factor;Breakpoint clu	BANF1;BAF;B	O75531;B2R4	1,4351	0,2395264	158660,1	2	2	2
Ubiquitin carboxyl-terminal hydrolase 7;Ubiquiti	USP7;HAUSP	Q93009;Q6U	1,4011	0,2415391	1,28E+07	24	24	24
Ubiquitin carboxyl-terminal hydrolase 5;Ubiquiti	USP5;ISOT	P45974-1;P45	1,4006	0,2417984	3351600	9	9	9
GMP synthase [glutamine-hydrolyzing];Glutamir	GMPS	P49915;A8K6	1,4295	0,2425451	1253100	6	6	6
Cytosolic acyl coenzyme A thioester hydrolase;L	ACOT7;BACH	O00154-1;O0	1,4265	0,2441757	291679,8	2	2	2
Flap endonuclease 1;Flap structure-specific end	FEN1;RAD2	P39748;B4DW	1,3912	0,2467178	3887901	6	6	6
ATP-binding cassette sub-family E member 1;RN	ABCE1;RLI;RN	P61221	1,391	0,2468234	5210203	8	8	8
Phosphoserine aminotransferase;Phosphohydro	PSAT1;PSA	Q9Y617-1;Q9	1,3904	0,2471404	5672603	14	14	14
Signal transducer and activator of transcription 1	STAT1	P42224-1;P42	1,4182	0,2487355	495390	3	3	3

Bifunctional methylenetetrahydrofolate dehydrc	MTHFD2;NMI	P13995;B2R7V	1,4169	0,2494562	1160099	2	2	2
Isocitrate dehydrogenase [NADP], mitochondrial	IDH2	P48735;B2R6I	1,4161	0,2499006	1047800	4	4	3
Nicastrin;cDNA FLJ60331, highly similar to Nicast	NCSTN;KIAA0	Q92542-1;Q9	1,4146	0,2507357	1366001	4	4	4
UV excision repair protein RAD23 homolog B;XP-	RAD23B;RP11	P54727;B3KW	1,4131	0,2515731	2178701	4	4	4
Electron transfer flavoprotein subunit alpha, mit	ETFA	P13804;Q53X	1,4127	0,2517968	2370600	7	7	7
Bcl-2-associated transcription factor 1	BCLAF1;BTF;K	Q9NYF8-1;Q9	1,4104	0,2530864	488749,9	3	3	3
Heat shock 70 kDa protein 4;Heat shock 70-relat	HSPA4;APG2	P34932;B0AZS	1,3786	0,2534485	9,39E+07	35	35	32
Small ubiquitin-related modifier 2;Ubiquitin-like	SUMO2;SMT3	P61956;B2R4I	1,4073	0,2548334	509880,2	2	2	2
Putative heat shock protein HSP 90-beta 4	HSP90AB4P	Q58FF6	1,4073	0,2548334	798950,3	8	2	2
Bifunctional protein NCOAT;Nuclear cytoplasmic	MGEA5;HEXC;	O60502-1;O6	1,4065	0,2552859	334590,2	3	3	3
Heterogeneous nuclear ribonucleoproteins A2/B	HNRNPA2B1;I	P22626-1;P22	1,3733	0,2563269	1,50E+07	11	11	11
Macrophage migration inhibitory factor;Phenylp	MIF;GLIF;MM	P14174;A5Z1I	1,3731	0,2564361	8,09E+07	4	4	4
Single-stranded DNA-binding protein, mitochonc	SSBP1;SSBP	Q04837;A4D1	1,3695	0,258408	3424800	8	8	8
Heat shock 70 kDa protein 4L;Osmotic stress prc	HSPA4L;APG1	O95757;A2ICT	1,3685	0,2589581	1,54E+07	26	22	22
NSFL1 cofactor p47;p97 cofactor p47;NSFL1 (P97	NSFL1C;RP4-7	Q9UNZ2-5;Q9	1,3999	0,2590448	1384100	4	4	4
Citrate synthase, mitochondrial;cDNA FLJ38537	CS	O75390;B4DJY	1,3664	0,2601165	1,34E+07	10	10	10
Uncharacterized protein RPL23A (Ribosomal pro	RPL23A;hCG_	A8MUS3;A8M	1,3976	0,2603656	1329501	4	4	4
FK506-binding protein 3;Peptidyl-prolyl cis-trans	FKBP3;FKBP25	Q00688;B2R4	1,3975	0,2604232	453410	6	6	6
Aldehyde dehydrogenase, mitochondrial;ALDH c	ALDH2;ALDM	P05091;B4YAI	1,3609	0,2631716	3042201	10	10	10
3,2-trans-enoyl-CoA isomerase, mitochondrial;D	DCI	P42126-1;P42	1,3601	0,2636185	2571601	6	6	6
Peroxiredoxin-2;Thioredoxin peroxidase 1;Thior	PRDX2;NKEFB	P32119;B4DF	1,3591	0,2641781	7220497	10	9	9
cDNA FLJ10554 fis, clone NT2RP2002385, highly	GPSN2;SC2	B3KM97;B3KS	1,3855	0,2674075	1272500	7	7	7
Cathepsin D;Cathepsin D light chain;Cathepsin D	CTSD;CPSD	P07339	1,3852	0,267584	664449,8	7	7	7
Glycogen phosphorylase, liver form	PYGL	P06737;B2R8	1,3516	0,2684074	3,15E+07	30	30	26
ADP/ATP translocase 1;Adenine nucleotide trans	SLC25A4;ANT	P12235;A8K7	1,3832	0,2687638	113560	12	2	2
Rho-associated protein kinase 2;Rho-associated,	ROCK2;KIAA0	O75116;Q53S	1,3827	0,2690594	1966000	5	5	5
MMS19 nucleotide excision repair protein homc	MMS19;MMS	Q96T76-1;Q9	1,3826	0,2691186	710389,9	2	2	2
Monocarboxylate transporter 1;Solute carrier fa	SLC16A1;MCT	P53985;B2R6	1,3477	0,2706294	2438099	2	2	2
Hypoxanthine-guanine phosphoribosyltransfera	HPRT1;HPRT	P00492;B2R8I	1,3476	0,2706866	6791601	10	10	10
GTPase-activating protein and VPS9 domain-con	GAPVD1;GAPI	Q14C86-6;Q1	1,3461	0,2715456	2740800	12	12	12
Golgi resident protein GCP60;Acyl-CoA-binding c	ACBD3;GCP60	Q9H3P7;B2RE	1,3737	0,2744266	720349,6	2	2	2
Adenylosuccinate synthetase isozyme 2;Adenylc	ADSS;ADSS2	P30520;B1AQ	1,341	0,2744832	2612299	5	5	5
14-3-3 protein gamma;Protein kinase C inhibitor	YWHAG	P61981;B3KN	1,3377	0,2763984	4916598	9	6	6

DNA polymerase delta catalytic subunit;DNA pol	POLD1;POLD	P28340;Q308	1,3364	0,2771559	4319603	8	8	8
Programmed cell death 6-interacting protein;AL	PDCD6IP;AIP1	Q8WUM4;B4I	1,3362	0,2772726	1,40E+07	22	22	22
Transaldolase	TALDO1;TAL;T	P37837;B2R8I	1,3357	0,2775646	5668097	10	10	10
Aspartyl-tRNA synthetase, mitochondrial;Aspart	DARS2	Q6PI48;A8K4A	1,3353	0,2777983	2456701	5	5	5
NEDD8;Ubiquitin-like protein Nedd8;Neddylin;N	NEDD8	Q15843;A8MU	1,3664	0,2788448	354699,9	2	2	2
Acidic leucine-rich nuclear phosphoprotein 32 fa	ANP32B;APRIL	Q92688-1;Q9	1,3313	0,2801448	5100299	8	8	5
cDNA FLJ53303, highly similar to Glucose-6-phos	GPI	B4DE36;B4DG	1,3313	0,2801448	2,68E+07	14	14	14
Cell division control protein 2 homolog;p34 prot	CDC2	P06493-1;P06	1,3303	0,2807341	2550001	6	6	6
DNA mismatch repair protein Msh2;MutS protei	MSH2	P43246;B4DL3	1,3627	0,2811064	1140701	6	6	6
Chromobox protein homolog 5;Heterochromatin	CBX5;HP1A	P45973;B2R8T	1,3605	0,2824582	292329,9	3	3	3
Heat shock protein beta-1;Heat shock 27 kDa pr	HSPB1;HSP27	P04792;B2R4I	1,3604	0,2825198	733639,9	3	3	3
ATPase family AAA domain-containing protein 3	ATAD3B;KIAA	Q5T9A4-1;Q5	1,3268	0,2828046	4058601	5	5	2
Protein 4.1;Band 4.1;P4.1;EPB4.1;4.1R;EPB41 pr	EPB41;E41P	P11171-1;P11	1,3589	0,2834447	304560,2	3	3	3
Pterin-4-alpha-carbinolamine dehydratase;4-alp	PCBD1;DCOH;	P61457;Q6FG	1,358	0,2840008	240220	3	3	3
Peroxiredoxin-1;Thioredoxin peroxidase 2;Thior	PRDX1;PAGA;	Q06830;B2R4	1,3247	0,2840531	5,94E+07	14	14	12
Translocon-associated protein subunit delta;Sigr	SSR4;TRAPD	P51571;A8K3	1,3223	0,2854856	3634702	4	4	4
Alpha-galactosidase A;Alpha-D-galactoside galac	GLA	P06280;Q53H	1,3514	0,2881064	1170300	3	3	3
cDNA, FLJ92409, highly similar to Homo sapiens	H2AFX;H2AX;I	B2R5B6;P161	1,3501	0,2889207	1975001	3	3	3
Tubulin-specific chaperone D;Tubulin-folding co	TBCD;KIAA098	Q9BTW9-4;Q9	1,3497	0,2891717	378870	2	2	2
Alanyl-tRNA synthetase, cytoplasmic;Alanine--tR	AARS	P49588;B4DR	1,3155	0,289577	8526304	20	20	20
DNA (cytosine-5)-methyltransferase 1;MCMT;DN	DNMT1;AIM;	P26358-1;P26	1,3141	0,2904254	3051700	15	15	15
14-3-3 protein epsilon	YWHAE	P62258;B3KY	1,3139	0,2905468	6,80E+07	19	19	17
Eukaryotic initiation factor 4A-I;ATP-dependent	EIF4A1;DDX2A	P60842;A8K0	1,312	0,2917019	8,19E+07	19	19	10
Aspartate aminotransferase, cytoplasmic;Transa	GOT1	P17174;B2R6I	1,3455	0,2918172	511270,1	5	5	5
Splicing factor, arginine/serine-rich 2;Splicing fac	SFRS2;hCG_27	Q01130;B3KU	1,3442	0,29264	2103400	2	2	2
NEDD8-activating enzyme E1 catalytic subunit;U	UBA3;UBE1C	Q8TBC4;A8K0	1,344	0,2927668	1527501	4	4	4
Activator of 90 kDa heat shock protein ATPase h	AHSA1;C14orf	O95433;B2R9	1,3417	0,2942277	2108099	4	4	4
Ras GTPase-activating-like protein IQGAP1;p195	IQGAP1;KIAA	P46940;A4QP	1,3062	0,2952517	1,96E+07	43	43	43
Ezrin;p81;Cytovillin;Villin-2;Cytovillin 2	EZR;VIL2	P15311;B2R6J	1,3054	0,2957442	1,23E+07	18	18	8
Creatine kinase B-type;Creatine kinase B chain;B	CKB;CKBB	P12277;A8K2	1,3001	0,2990237	7,13E+07	19	19	19
Peflin;PEF protein with a long N-terminal hydrop	PEF1;ABP32;U	Q9UBV8	1,3339	0,2992257	100890	2	2	2
DNA fragmentation factor subunit alpha;DNA fra	DFFA;DFF1;DF	O00273-1;O0	1,3334	0,2995484	278020	2	2	2
Calponin-3;Calponin, acidic isoform;cDNA FLJ53	CNN3	Q15417;B4DF	1,3299	0,3018151	519250,3	3	3	3

tRNA (cytosine-5-)-methyltransferase NSUN2;NC	NSUN2;SAKI;T	Q08J23;A8K52	1,3299	0,3018151	1908801	4	4	4
Methylosome protein 50;WD repeat-containing	WDR77;MEP5	Q9BQA1;B3KN	1,2954	0,3019569	1,14E+07	8	8	8
XTP3-transactivated gene A protein;RS21C6	XTP3TPA;CDA	Q9H773	1,328	0,3030513	600970,1	3	3	3
Translocon-associated protein subunit alpha;Sigi	SSR1;TRAPA;P	P43307-1;P43	1,2879	0,3066865	4440899	4	4	4
Zinc phosphodiesterase ELAC protein 2;Ribonucl	ELAC2;HPC2	Q9BQ52-1;Q9	1,322	0,3069816	596769,9	2	2	2
Transferrin receptor protein 1;T9;p90;Transferri	TFRC	P02786;A8K6(	1,2874	0,3070039	1,09E+07	16	16	16
Aflatoxin B1 aldehyde reductase member 2;AFB:	AKR7A2;AFAR	O43488;Q8NH	1,3217	0,3071792	375350,2	2	2	2
RCC1-I;Regulator of chromosome condensation;	RCC1;CHC1	Q16269;Q6NT	1,2868	0,3073852	3921798	6	6	6
Xaa-Pro dipeptidase;Proline dipeptidase;Imidodi	PEPD;PRD	P12955;A8K3Z	1,321	0,3076406	590799,8	2	2	2
6-phosphogluconate dehydrogenase, decarboxy	PGD;PGDH	P52209;A9Z1X	1,2862	0,3077668	1,50E+07	14	14	14
Inorganic pyrophosphatase;Pyrophosphate phos	PPA1;IOPPP;P	Q15181	1,2854	0,3082763	7654497	14	14	14
cDNA FLJ54528, highly similar to Serine/threonir	STK24;MST3;S	B4E0Y9;B4DT!	1,3196	0,308565	505729,9	2	2	2
Protein transport protein Sec61 subunit beta	SEC61B	P60468;Q53F/	1,3194	0,3086973	860209,6	2	2	2
Ornithine aminotransferase, mitochondrial;Orni	OAT	P04181;Q59H	1,2838	0,3092974	1,36E+07	13	13	13
ES1 protein homolog, mitochondrial;Protein KNI	C21orf33;HES	P30042-1;P30	1,315	0,311618	78703,03	3	3	3
NudC domain-containing protein 3;KIAA1068 pr	NUDCD3;KIAA	Q8IVD9;B0FTY	1,3138	0,3124184	408060,1	2	2	2
Histidyl-tRNA synthetase, cytoplasmic;Histidine-	HARS;HRS	P12081;B3KW	1,2772	0,3135385	3570098	10	10	10
cDNA FLJ75174, highly similar to Homo sapiens (	CALM1;CALM	A8K1M2;P621	1,276	0,3143145	1,10E+07	6	6	6
Alpha-enolase;2-phospho-D-glycerate hydro-lyas	ENO1;ENO1L1	P06733-1;P06	1,2751	0,3148977	2,07E+08	21	21	16
Endoplasmic reticulum protein ERp29;ERp31;ER	ERP29;C12orf	P30040	1,3098	0,3150978	1279800	7	7	7
Protein transport protein Sec61 subunit gamma	SEC61G	P60059;B2R4J	1,3091	0,3155685	91045,04	2	2	2
Crk-like protein	CRKL	P46109;A8KA	1,3089	0,3157032	1042800	4	4	4
Armadillo repeat-containing X-linked protein 3;A	ARMCX3;ALEX	Q9UH62;A8K5	1,3084	0,3160398	152280,1	2	2	2
Heterogeneous nuclear ribonucleoprotein H2;He	HNRNPH2;FTF	P55795;A1L4(	1,3081	0,316242	2001100	11	4	4
78 kDa glucose-regulated protein;GRP 78;Heat s	HSPA5;GRP78	P11021;B0QZ	1,2704	0,317957	9,51E+07	27	25	25
Lysosomal-associated membrane protein 2C (Lys	LAMP2;hCG_3	Q6Q3G8;P134	1,2704	0,317957	4129499	4	4	4
Transcription factor BTF3;RNA polymerase B tra	BTF3;NACB;OI	P20290-1;P20	1,3051	0,3182692	595120,1	5	5	5
Inosine-5'-monophosphate dehydrogenase 2;IM	IMPDH2;IMPD	P12268;Q6QE	1,2694	0,3186111	8,70E+07	21	21	21
Protein disulfide-isomerase A4;Protein ERp-72	PDIA4;ERP70;	P13667;A8K4I	1,2662	0,3207112	1,18E+07	12	12	12
Cullin-3	CUL3;KIAA061	Q13618-1;Q1:	1,3013	0,3208514	371540,2	2	2	2
Protein RCC2;Telophase disk protein of 60 kDa;R	RCC2;KIAA147	Q9P258;A5PL	1,2644	0,3218974	8157803	9	9	9
High mobility group protein B1;High mobility grc	HMGB1;HMG	P09429;Q5T7(	1,2641	0,3220954	7814398	9	9	8
CTP synthase 1;UTP--ammonia ligase 1;CTP synt	CTPS	P17812;B4DM	1,2634	0,3225579	1,86E+07	11	11	11

Isochorismatase domain-containing protein 1	ISOC1;CGI-111	Q96CN7	1,2968	0,3239305	512780,3	3	3	3
Heat shock protein 75 kDa, mitochondrial;Tumo	TRAP1;HSP75	Q12931;B4DR	1,2609	0,3242139	1,07E+08	12	12	12
Alcohol dehydrogenase class-3;Alcohol dehydro	ADH5;ADHX;F	P11766;Q5U0	1,2952	0,3250308	992640,5	3	3	3
Eukaryotic translation initiation factor 1;Protein	EIF1;SUI1;EIF1	P41567;Q6IAV	1,2943	0,325651	126940	3	3	3
DNA-(apurinic or apyrimidinic site) lyase;Apurini	APEX1;APE;AF	P27695;Q5TZ	1,2938	0,3259959	1517401	7	7	7
Uridine 5'-monophosphate synthase;Orotate ph	UMPS;OK/SW	P11172-1;P11	1,2579	0,3262101	3459801	7	7	7
Thioredoxin-dependent peroxide reductase, mit	PRDX3;AOP1;	P30048;A4UC	1,2575	0,3264769	4839398	6	6	6
Uncharacterized protein NACAP1;Nascent polyp	NACAP1;NAC	A8MTN7;Q13	1,2571	0,326744	7585399	4	4	4
Protein KIAA0196 variant (Uncharacterized prot	KIAA0196	Q53EL1;Q127	1,2926	0,3268249	291050	2	2	2
Transcription elongation factor SPT6;Tat-cotrans	SUPT6H;KIAA(	Q7KZ85-1;Q7	1,2904	0,3283489	706959,8	4	4	4
ATP-dependent DNA helicase Q1;DNA-depender	RECQL;RECQL	P46063;A8K6	1,2877	0,3302268	2073199	4	4	4
Cob(I)yrinic acid a,c-diamide adenosyltransfera	MMAB	Q96EY8;B2R6	1,2838	0,332954	97879,05	2	2	2
Ataxin-10;Spinocerebellar ataxia type 10 protein	ATXN10;SCA1	Q9UBB4;B4D	1,2478	0,3330024	2438499	5	5	5
Cullin-associated NEDD8-dissociated protein 1;C	CAND1;KIAA0	Q86VP6-1;Q8	1,2463	0,3340207	7,18E+07	39	39	38
14-3-3 protein theta;14-3-3 protein tau;14-3-3 p	YWHAQ	P27348;B4DM	1,246	0,3342246	1,10E+07	13	10	10
Cytochrome c oxidase polypeptide VIc	COX6C	P09669;B2R4	1,2817	0,3344295	380569,8	3	3	3
Protein transport protein Sec61 subunit alpha is	SEC61A1;SEC6	P61619-1;P61	1,2812	0,3347816	1648301	4	4	4
Thyroid hormone receptor-associated protein 3;	THRAP3;TRAP	Q9Y2W1;Q05	1,2775	0,3373956	375630,2	2	2	2
Heterogeneous nuclear ribonucleoprotein H	HNRNPH1;HN	P31943;B3KW	1,241	0,337638	6,62E+07	14	14	6
DAZ-associated protein 1;Deleted in azoospermi	DAZAP1	Q96EP5-1;Q9	1,2766	0,3380338	1245200	3	3	3
14-3-3 protein zeta/delta;Protein kinase C inhibi	YWHAZ	P63104;A8K1	1,2404	0,3380495	2,69E+07	13	11	10
C-1-tetrahydrofolate synthase, cytoplasmic;Met	MTHFD1;MTH	P11586;B2R5	1,238	0,3396992	5,49E+07	40	40	40
Staphylococcal nuclease domain-containing prot	SND1;TDRD11	Q7KZF4;B2R5	1,2378	0,339837	1,09E+07	26	26	26
Acidic leucine-rich nuclear phosphoprotein 32 fa	ANP32E;RP4-7	Q9BTT0;Q53F	1,2723	0,3410956	843110,3	4	4	4
14-3-3 protein beta/alpha;Protein kinase C inhib	YWHAB	P31946-1;P31	1,2345	0,3421163	5211299	9	6	5
Basic leucine zipper and W2 domain-containing	BZW1;BZAP45	Q7L1Q6-1;Q7	1,2342	0,3423241	5563504	9	9	8
FACT complex subunit SSRP1;Facilitates chroma	SSRP1;FACT8	Q08945	1,2334	0,3428788	2,16E+07	18	18	18
General transcription factor IIF subunit 1;Transc	GTF2F1;RAP7	P35269;B2RC	1,2668	0,3450422	697620,4	2	2	2
Translational activator GCN1;GCN1-like protein	GCN1L1;KIAA(	Q92616;A8KA	1,2297	0,345453	8679101	45	45	45
Polypyrimidine tract binding protein 1 (Polypyrir	PTBP1;hCG_2	Q9BUQ0;P265	1,2296	0,3455228	1,15E+07	12	12	11
Histone acetyltransferase type B catalytic subun	HAT1;hCG_16	O14929;Q6P5	1,2295	0,3455925	1,81E+07	14	14	14
Exportin-5;Ran-binding protein 21;cDNA FLJ142	XPO5;KIAA12	Q9HAV4-1;Q9	1,2275	0,3469907	1,23E+07	18	18	18
cDNA FLJ53231, highly similar to 4-trimethylami	ALDH9A1;ALD	B4DX14;P491	1,2238	0,3495888	4861599	8	8	8

RuvB-like 2;48 kDa TATA box-binding protein-int	RUVBL2;TIP48	Q9Y230;B3KN	1,2233	0,349941	5,11E+07	22	22	22
14-3-3 protein eta;Protein AS1;Tyrosine 3-mono	YWHAH;YWHA	Q04917;B2R6	1,2232	0,3500115	2393001	9	7	7
L-lactate dehydrogenase A chain;LDH muscle sul	LDHA;PIG19	P00338-1;P00	1,2227	0,3503641	7,01E+07	23	23	21
SUMO-activating enzyme subunit 2;Ubiquitin-lik	UBA2;SAE2;UI	Q9UBT2;B2RD	1,2225	0,3505052	5467299	8	8	8
Transcription elongation factor B polypeptide 2;I	TCEB2	Q15370	1,2209	0,3516355	3365600	5	5	5
Asparagine synthetase [glutamine-hydrolyzing];(	ASNS;TS11	P08243;A4D1	1,2207	0,351777	1,05E+07	13	13	13
Hypoxia up-regulated protein 1;150 kDa oxygen-	HYOU1;ORP15	Q9Y4L1;A8C1	1,2174	0,3541181	2,54E+07	28	28	28
Poly [ADP-ribose] polymerase 1;ADPRT;NAD(+) A	PARP1;ADPRT	P09874;B1AN	1,2161	0,3550436	1,76E+08	46	46	46
Acidic leucine-rich nuclear phosphoprotein 32 fa	ANP32A;C15o	P39687;B2R6	1,2151	0,3557567	3661198	7	4	4
Glyceraldehyde-3-phosphate dehydrogenase;Gly	GAPDH;GAPD	P04406;Q2TSI	1,2122	0,3578311	1,66E+08	16	16	4
Ubiquitin carboxyl-terminal hydrolase 24;Ubiqui	USP24;KIAA1C	Q9UPU5	1,2486	0,358346	841510,1	4	4	4
Amidophosphoribosyltransferase;Glutamine pho	PPAT;GPAT	Q06203;A8K4	1,2484	0,3584943	1206200	3	3	3
Alkyldihydroxyacetonephosphate synthase, pero	AGPS;AAG5	O00116;A5D8	1,248	0,358791	887219,9	3	3	3
Proteasome subunit alpha type-3;Proteasome co	PSMA3;PSC8	P25788-1;P25	1,2065	0,361935	1,31E+07	11	11	11
Tripeptidyl peptidase II;Tripeptidyl-peptidase 2;T	TPP2;RP11-29	Q5VZU9;P291	1,2434	0,3622158	1231300	4	4	4
Sorcin;22 kDa protein;CP-22;V19;Uncharacterize	SRI	P30626;B4DH	1,2429	0,3625894	1040901	6	6	6
ATP-binding cassette sub-family F member 1;ATI	ABCF1;ABC50	Q8NE71-1;Q8	1,2045	0,3633835	2978001	8	8	8
Calreticulin;CRP55;Calregulin;HACBP;ERp60;grp6	CALR;CRTC	P27797;B4DH	1,204	0,3637463	9074496	9	9	8
60S ribosomal protein L5;Ribosomal protein L5	RPL5;MSTP03	P46777;A2RU	1,2385	0,3658901	1948600	4	4	4
Tubulin gamma-1 chain;Gamma-1-tubulin;Gamm	TUBG1;TUBG;	P23258;Q53X	1,2377	0,3664926	656129,8	2	2	2
Uncharacterized protein C3orf60;C3orf60 protei	C3orf60;hCG_	Q9BU61;A4FU	1,2377	0,3664926	683129,6	3	3	3
Heterogeneous nuclear ribonucleoprotein H3;cD	HNRNPH3;HN	P31942-1;P31	1,1958	0,3697349	2573499	5	4	4
Adenylosuccinate lyase;Adenylosuccinase	ADSL;AMPS	P30566-1;P30	1,1945	0,370691	6028904	7	7	7
Elongator complex protein 1;IkappaB kinase con	IKBKAP;ELP1;I	O95163;B3KM	1,1936	0,371354	7472100	15	15	15
Thimet oligopeptidase;Endopeptidase 24.15;MP	THOP1	P52888;Q96C	1,231	0,3715664	1474101	5	5	5
Importin-5;Importin subunit beta-3;Karyopherin	IPO5;KPNB3;R	O00410;B3KW	1,1915	0,3729046	3,66E+07	33	33	24
Cytochrome b5 type B;Cytochrome b5 outer mit	CYB5B;CYB5M	O43169;A8K6	1,2282	0,3737016	753379,8	4	4	4
UPF0557 protein C10orf119	C10orf119	Q9BTE3-1;Q9I	1,1877	0,3757223	3927298	8	8	8
Moesin;Membrane-organizing extension spike p	MSN	P26038;Q6PJT	1,2233	0,3774595	1831701	16	8	7
Glucose-6-phosphate 1-dehydrogenase;cDNA FL	G6PD	P11413-2;P11	1,2227	0,3779214	973679,4	8	8	8
Glutamate dehydrogenase 1, mitochondrial;Glut	GLUD1;GLUD;	P00367;B3KT1	1,1814	0,3804287	1,58E+07	13	13	13
Cysteine and histidine-rich domain-containing pr	CHORDC1;CHI	Q9UHD1-1;Q9	1,2191	0,3807018	1461501	5	5	5
Hydroxyacyl-coenzyme A dehydrogenase, mitoc	HADH;HAD;HA	Q16836-2;Q1	1,2188	0,3809342	625099,8	2	2	2

Threonyl-tRNA synthetase, cytoplasmic;Threonin	TARS	P26639;A8K8I	1,1777	0,3832127	2,41E+07	29	29	29
DnaJ homolog subfamily C member 8;Splicing pr	DNAJC8;SPF31	O75937;B4DU	1,2145	0,3842755	2026000	4	4	4
Putative uncharacterized protein PPA2;Inorganic	PPA2;HSPC12	Q4W5E9;Q96	1,2141	0,3845873	943829,9	6	6	6
Estradiol 17-beta-dehydrogenase 12;17-beta-hy	HSD17B12	Q53GQ0;A8K9	1,2138	0,3848214	1909300	8	8	8
40S ribosomal protein S14	RPS14;PRO26	P62263;B2R5	1,1755	0,3848752	4672798	5	5	5
Radixin isoform b (Radixin, isoform CRA_a) (Radi	RDX;hCG_391	A7YIJ8;A7YIK3	1,2105	0,3874021	1001300	15	4	4
Ras-related protein Rab-1B;Ras-related protein F	RAB1B;RAB1A	Q9H0U4;A8K7	1,1717	0,3877591	2668199	7	7	7
NMDA receptor-regulated protein 1;N-terminal a	NARG1;GA19;	Q9BXJ9-1;Q9E	1,1711	0,3882158	1,09E+07	16	16	16
UNC45 homolog A;GCUNC-45;Smooth muscle ce	UNC45A;SMA	Q9H3U1-1;Q9	1,171	0,388292	3056202	7	7	7
Putative uncharacterized protein DKFZp686P031	DKFZp686P03	Q6N0B3;Q8TE	1,1704	0,3887492	3417599	11	11	11
Heterogeneous nuclear ribonucleoprotein A1;He	HNRNPA1;HN	P09651-1;P09	1,1689	0,389894	1,20E+07	10	10	9
Proteasome inhibitor PI31 subunit;cDNA FLJ5721	PSMF1;RP4-54	Q92530;B4DU	1,2064	0,3906252	203099,9	2	2	2
Pre-mRNA-splicing factor RBM22;RNA-binding m	RBM22;ZC3H1	Q9NW64;B4D	1,206	0,3909407	1380100	3	3	3
Transketolase;cDNA FLJ36348 fis, clone THYMU2	TKT;DKFZp686	P29401;A8K0	1,1648	0,3930353	4,68E+07	25	25	25
Microtubule-associated protein RP/EB family me	MAPRE1	Q15691;A2VC	1,1639	0,3937273	6075504	9	9	9
L-lactate dehydrogenase B chain;LDH heart subu	LDHB	P07195;Q5U0	1,1631	0,3943432	1,26E+08	18	16	16
Pyruvate dehydrogenase E1 component subunit	PDHB;PHE1B	P11177-1;P11	1,1999	0,3957731	2064201	6	6	6
Methylosome subunit pICln;Chloride conductanc	CLNS1A;CLCI;I	P54105;B2RC	1,1992	0,3963302	1604501	3	3	3
Rab3 GTPase-activating protein catalytic subunit	RAB3GAP1;KI	Q15042-1;Q1	1,1605	0,3963494	2615600	9	9	9
Splicing factor, arginine/serine-rich 3;Pre-mRNA-	SFRS3;SRP20;I	P84103;B2R6I	1,1989	0,3965692	1952001	3	3	2
Glutathione S-transferase Mu 3;GSTM3-3;GST cl	GSTM3;GST5	P21266;B4E2J	1,1988	0,3966489	522310	4	4	3
Four and a half LIM domains protein 1;Skeletal n	FHL1;SLIM1;R	Q13642-2;Q1	1,1978	0,3974461	969459,9	5	5	5
Band 4.1-like protein 2;Generally expressed prot	EPB41L2;DKFZ	O43491;Q6R5	1,1589	0,3975876	6706204	14	14	13
Cytochrome b-c1 complex subunit Rieske-like pr	UQCRFSL1;UC	P0C7P4;A8K5	1,1975	0,3976856	1600700	6	6	6
Heterogeneous nuclear ribonucleoprotein A/B;A	HNRNPAB;ABI	Q99729-2;Q9	1,1975	0,3976856	1392600	3	2	2
Putative uncharacterized protein DKFZp686M24	DKFZp686M2	Q5HYG7;Q5T	1,1969	0,3981647	2387900	5	5	5
Cytoskeleton-associated protein 5;Colonic and h	CKAP5;KIAA0	Q14008-1;Q1	1,1573	0,3988286	1,26E+07	42	42	42
Leucine-rich PPR motif-containing protein, mito	LRPPRC;LRP13	P42704;B4DSI	1,1573	0,3988286	5,83E+07	55	55	55
Heterogeneous nuclear ribonucleoprotein D0;AU	HNRNPD;AUF	Q14103-1;Q1	1,1556	0,4001501	6867799	4	4	3
Small glutamine-rich tetratricopeptide repeat-co	SGTA;SGT	O43765;Q6FI	1,1935	0,400887	1305401	4	4	4
Asparaginyl-tRNA synthetase, cytoplasmic;Aspar	NARS;ASNS	O43776;B4DN	1,1526	0,4024897	4600198	6	6	6
UBX domain-containing protein 8;Protein ETEA	UBXD8;ETEA;I	Q96CS3;B4E2I	1,1897	0,4039446	2363599	4	4	4
cDNA FLJ78564, highly similar to Homo sapiens a	ALDH5A1;SSA	A8K435;B2RD	1,1892	0,4043481	574799,8	3	3	3

Serine/threonine-protein kinase TAO1;Thousanc	TAOK1;KIAA13	Q7L7X3-1;Q7L	1,1838	0,408723	310399,9	3	3	3
Talin-1	TLN1;KIAA102	Q9Y490;Q5TC	1,1439	0,4093289	1,81E+07	54	54	54
Bifunctional purine biosynthesis protein PURH;P	ATIC;PURH;OI	P31939;A8K2	1,1438	0,409408	1,75E+07	20	20	20
Stress-70 protein, mitochondrial;75 kDa glucose	HSPA9;GRP75	P38646;A1XP	1,1412	0,4114679	4,51E+07	21	21	21
TAR DNA-binding protein 43;TAR DNA binding pı	TARDBP;TDP4	Q13148;A4GL	1,1411	0,4115471	6452802	7	7	7
Acylamino-acid-releasing enzyme;Acyl-peptide h	APEH;D3F15S	P13798	1,179	0,4126382	1211900	2	2	2
RuvB-like 1;49 kDa TATA box-binding protein-int	RUVBL1;NMP	Q9Y265-1;Q9	1,1395	0,4128184	5,24E+07	19	19	19
Sialic acid synthase;N-acetylneuraminate syntha	NANS;SAS	Q9NR45;B2RE	1,1394	0,412898	8515906	11	11	11
Transcription elongation factor B polypeptide 1;	TCEB1	Q15369	1,1393	0,4129776	3661499	6	6	6
Glutamine-rich protein 1;CDNA FLJ20259 fis, clo	QRICH1	Q2TAL8;A1L3	1,1784	0,4131293	208959,9	2	2	2
DnaJ homolog subfamily B member 1;Heat shocl	DNAJB1;DNAJ	P25685;B4DX	1,1375	0,4144113	3367999	13	13	13
Importin-7;Ran-binding protein 7	IPO7;RANBP7	O95373;B2R7	1,1368	0,4149698	2,55E+07	20	20	19
NEDD8-conjugating enzyme Ubc12;Ubiquitin-coı	UBE2M;UBC1	P61081	1,1754	0,4155908	1553901	5	5	5
Heat shock protein HSP 90-beta;HSP 84	HSP90AB1;HS	P08238;A8K3	1,1359	0,4156887	4,62E+08	44	28	27
cAMP-dependent protein kinase type II-alpha re	PRKAR2A;PKR	P13861;A8KA	1,1749	0,416002	1695101	5	5	5
Golgi-specific brefeldin A-resistance guanine nuc	GBF1;KIAA02	Q92538;Q05B	1,1742	0,4165781	450450,1	7	6	6
Seryl-tRNA synthetase;Seryl-tRNA synthetase, cy	SARS;RP11-35	Q5T5C7;P495	1,1344	0,4168887	7257199	6	6	6
Protein tyrosine phosphatase-like protein PTPLA	PTPLAD1;BINI	Q9P035;B4DR	1,1694	0,4205427	1885201	3	3	3
Ribonucleoside-diphosphate reductase large sub	RRM1;RR1	P23921;B4DN	1,1297	0,4206639	4532800	9	9	9
Dynamin-1-like protein;Dynamin-like protein;Dn	DNM1L;DLP1;	O00429-3;O0	1,1678	0,4218696	1325701	5	5	5
Importin subunit alpha-7;Karyopherin subunit al	KPNA6;IPOA7	O60684;B2RD	1,1668	0,4227003	780380,5	3	3	3
Enoyl-CoA hydratase, mitochondrial;Short chain	ECHS1	P30084	1,166	0,4233656	1897200	7	7	7
Tubulin--tyrosine ligase-like protein 12;Tubulin t	TTLL12;KIAA0	Q14166;B1AH	1,1219	0,42698	5237303	8	8	8
cDNA FLJ40287 fis, clone TESTI2027909, highly s	PRKAG1	Q8N7V9;P546	1,1609	0,427623	706889,8	4	4	4
Acetyl-CoA acetyltransferase, mitochondrial;Ace	ACAT1;ACAT;I	P24752;B2R6I	1,1197	0,4287729	4619597	12	12	12
Aldehyde dehydrogenase X, mitochondrial;Aldel	ALDH1B1;ALD	P30837;B2R8I	1,1592	0,4290482	2140800	5	5	5
cDNA FLJ51313, highly similar to Microsomal sig	SEC11L1;hCG_	B4DUL4;Q7Z4	1,159	0,429216	753560,1	2	2	2
SUMO-activating enzyme subunit 1;Ubiquitin-lik	SAE1;AOS1;SU	Q9UBE0;B2RD	1,1565	0,4313179	1791200	5	5	5
Importin-8;Ran-binding protein 8	IPO8;RANBP8	O15397;Q59F	1,1555	0,4321604	320580,1	3	2	2
Dedicator of cytokinesis protein 7	DOCK7;KIAA1	Q96N67-1;Q9	1,1143	0,4331948	4874503	10	10	10
Ras-related protein Rab-6A;Rab6A variant 3;Ras-	RAB6A;RAB6;I	P20340-1;P20	1,154	0,4334262	1385500	6	6	6
Signal recognition particle receptor subunit beta	SRPRB;PSEC02	Q9Y5M8;Q54	1,1133	0,434017	2395901	8	8	8
Syntaxin-binding protein 3;Unc-18 homolog 3;Uı	STXBP3	O00186;A8K5	1,1533	0,4340178	679830,3	3	3	3

cDNA FLJ56890, highly similar to Fructose-bisph	ALDOA;ALDA	B4DXI7;P0407	1,113	0,4342638	1,10E+08	22	22	19
Epoxide hydrolase 1;Microsomal epoxide hydrol	EPHX1;EPHX;E	P07099;B2R8I	1,1128	0,4344284	2678300	5	5	5
Hsp90 co-chaperone Cdc37;Hsp90 chaperone pr	CDC37;CDC37	Q16543;A1L0	1,1122	0,4349225	9435704	8	8	8
Phenylalanyl-tRNA synthetase alpha chain;Pheny	FARSA;FARS;F	Q9Y285;Q6IBI	1,1504	0,4364737	1984801	3	3	3
Ras-related protein Rab-11B;GTP-binding protei	RAB11B;YPT3;	Q15907;A5YM	1,1103	0,4364896	5255601	9	9	9
Membrane-associated progesterone receptor cc	PGRMC1;HPR	O00264;Q6IB	1,1078	0,4385571	5585304	8	8	7
Fumarate hydratase, mitochondrial	FH	P07954-1;P07	1,1076	0,4387228	8818802	9	9	9
Elongation factor 1-alpha 1;eEF1A-1;Elongation	EEF1A1;EEF1A	P68104;A8K9	1,1074	0,4388886	6,38E+08	19	19	8
60S ribosomal protein L17;60S ribosomal protei	RPL17	P18621;B2R4I	1,1469	0,4394492	493829,8	3	3	3
NADH dehydrogenase [ubiquinone] iron-sulfur p	NDUFS3	O75489;B2R9	1,1064	0,4397177	2406599	9	9	9
Hepatoma-derived growth factor;High-mobility g	HDGF;HMG1L	P51858;B2RD	1,1463	0,4399607	1971101	6	6	5
RNA-binding protein 26;RNA-binding motif prote	RBM26;C13or	Q5T8P6-2;Q5	1,1056	0,4403818	3594000	11	11	10
Nucleoside diphosphate kinase 3;Nucleoside dip	NME3	Q13232;Q9NU	1,1455	0,440643	291610	2	2	2
Superkiller viralicidic activity 2-like 2;ATP-depen	SKIV2L2;KIAA	P42285;A8K6I	1,1451	0,4409844	710439,6	4	4	4
C-jun-amino-terminal kinase-interacting protein	SPAG9;HSS;KI	O60271-1;O6	1,1434	0,4424374	648659,7	6	6	6
RNA-binding protein 39;RNA-binding motif prote	RBM39;HCC1;	Q14498-1;Q1	1,143	0,4427796	2054601	4	4	4
Actin-related protein 2/3 complex subunit 4;Arp	ARPC4;ARC20	P59998;B2RC	1,1022	0,4432114	3772002	5	5	5
Oxysterol-binding protein 1	OSBP;OSBP1	P22059-1;P22	1,1016	0,443712	2628499	7	7	6
Insulin-degrading enzyme;Insulin protease;Insuly	IDE	P14735;B2R7	1,1016	0,443712	4526602	11	11	11
Ras-related protein Rap-1A;GTP-binding protein	RAP1A;KREV1	P62834;A8KA	1,1417	0,4438932	1768601	5	5	5
Delta(3,5)-Delta(2,4)-dienoyl-CoA isomerase, mi	ECH1	Q13011;A8K7	1,1398	0,4455239	578869,9	3	3	2
Heterogeneous nuclear ribonucleoprotein U (Sc	HNRNPU;RP1	Q5RI17;Q96B	1,0983	0,4464716	5285598	12	12	11
Glutaminase kidney isoform, mitochondrial;L-glu	GLS;GLS1;KIA	O94925-1;O9	1,1371	0,4478473	1258801	3	3	3
Mitochondrial import receptor subunit TOM40 h	TOMM40;C19	O96008-1;O9	1,1352	0,4494866	2065001	4	4	4
MHC class I antigen;HLA class I histocompatibilit	HLA-A;HLAA;H	A1Z1D7;A9YW	1,1346	0,4500051	306130,1	2	2	2
NADH dehydrogenase [ubiquinone] iron-sulfur p	NDUFS2	O75306;Q53H	1,093	0,4509263	6640704	10	10	10
Developmentally-regulated GTP-binding protein	DRG2	P55039;B2R8	1,1332	0,4512162	457010,1	2	2	2
GTP-binding nuclear protein Ran;GTPase Ran;Ra	RAN;ARA24;O	B5MDF5;P62	1,0916	0,4521078	1,91E+07	13	13	13
cDNA, FLJ93564, highly similar to Homo sapiens	SOD2;hCG_34	B2R7R1;Q4ZJI	1,131	0,4531233	945989,9	5	5	5
U5 small nuclear ribonucleoprotein 200 kDa heli	ASCC3L1;HELI	O75643-1;O7	1,089	0,4543069	1,47E+07	46	46	46
Monofunctional C1-tetrahydrofolate synthase, n	MTHFD1L;FTH	Q6UB35-1;Q6	1,0887	0,4545611	3165600	9	9	9
Thioredoxin domain-containing protein 5;Thiore	TXNDC5;TLP4	Q8NBS9;Q65	1,0884	0,4548154	1,26E+07	11	11	11
cDNA FLJ76863, highly similar to Homo sapiens s	STIP1	A8K690;P319	1,0874	0,4556636	5,25E+07	23	23	23

N-acetyltransferase 13	NAT13;MAK3;	Q9GZZ1-1;Q9	1,1274	0,4562545	178220,1	2	2	2
N-terminal acetyltransferase complex ARD1 sub	ARD1A;ARD1;	P41227;A6NM	1,0862	0,4566827	2615800	6	6	6
Extended-synaptotagmin-1;Membrane-bound C	FAM62A;ESYT	Q9BSJ8-2;Q9E	1,0849	0,4577884	5487401	11	11	11
Heterogeneous nuclear ribonucleoprotein A3;cD	HNRNPA3;HN	P51991-1;P51	1,122	0,4609751	1612000	4	3	3
Platelet-activating factor acetylhydrolase IB sub	PAFAH1B1;LIS	P43034-1;P43	1,0774	0,4641994	3279500	8	8	8
Glycyl-tRNA synthetase;Glycine--tRNA ligase	GARS	P41250;B3KQ	1,0772	0,464371	1,18E+07	15	15	15
Eukaryotic translation initiation factor 5	EIF5	P55010;Q05D	1,1163	0,4659886	1788801	5	5	5
Calcyclin-binding protein;Siah-interacting protei	CACYBP;S100/	Q9HB71-1;Q9	1,0742	0,4669512	5947999	9	9	9
Tryptophanyl-tRNA synthetase, cytoplasmic;Tryp	WARS;IFI53;W	P23381;B4DTI	1,0732	0,4678133	4613299	9	9	9
STE20-like serine/threonine-protein kinase;STE2	SLK;KIAA0204	Q9H2G2-1;Q9	1,1134	0,4685513	1863400	9	9	9
Glucosamine--fructose-6-phosphate aminotrans	GFPT1;GFAT;G	Q06210-1;Q0	1,1124	0,4694368	1848599	6	6	6
Probable ATP-dependent RNA helicase DDX46;D	DDX46;KIAA0	Q7L014;A8K6	1,1116	0,4701459	1439700	4	4	4
Eukaryotic translation initiation factor 5B;Transl	EIF5B;IF2;KIA/	O60841;A0JLF	1,0683	0,4720507	7497303	11	11	11
Filamin-C;Gamma-filamin;Filamin-2;Protein FLN	FLNC;ABPL;FL	Q14315-1;Q14	1,1071	0,474146	189590,1	8	3	3
Probable ATP-dependent RNA helicase DDX17;D	DDX17;RP3-43	Q92841-1;Q9	1,0656	0,4743953	4,56E+07	24	24	18
Proteasome subunit alpha type-1;Proteasome c	PSMA1;PROS3	P25786-2;P25	1,0652	0,4747433	9539801	9	9	9
Sideroflexin-1;Tricarboxylate carrier protein	SFXN1	Q9H9B4;B3KP	1,0641	0,4757009	3913299	9	9	9
Interleukin enhancer-binding factor 2;Nuclear fa	ILF2;NF45;PR	Q12905;B2R8	1,105	0,4760191	839989,5	4	4	4
Far upstream element-binding protein 2;KH type	KHSRP;FUBP2	Q92945-1;Q9	1,0634	0,4763108	4,14E+07	18	18	18
Basigin;Leukocyte activation antigen M6;Collage	BSG;UNQ6505	P35613-1;P35	1,0623	0,4772703	1,51E+07	8	8	8
Cullin-1	CUL1	Q13616;B3KT	1,1035	0,4773597	1405301	7	7	7
Mannosyl-oligosaccharide glucosidase;Processin	GCS1	Q13724;A8K9	1,1033	0,4775386	2134100	4	4	4
DNA-directed RNA polymerases I and III subunit	POLR1C;POLR	O15160-1;O15	1,1027	0,4780754	1157700	4	4	4
4F2 cell-surface antigen heavy chain;Lymphocyt	SLC3A2;MDU1	P08195;B4E2	1,061	0,4784057	1,12E+07	13	13	13
cDNA FLJ54828, highly similar to Src substrate c	CTTN;hCG_23	B4E358;Q8N7	1,0593	0,4798927	2905498	4	4	4
Synaptojanin-2-binding protein;Mitochondrial o	SYNJ2BP;OMF	P57105	1,0991	0,4813037	183350,1	3	3	3
Signal recognition particle receptor subunit alph	SRPR	P08240;B2R5	1,0991	0,4813037	733730,1	3	3	3
Trifunctional purine biosynthetic protein adenos	GART;PGFT;PI	P22102-1;P22	1,0571	0,481821	2,07E+07	25	25	25
40S ribosomal protein S12	RPS12;RP5-11	Q76M58;P25	1,0555	0,4832263	7330097	8	8	8
Tumor protein D52-like 2 (Tumor protein D52-lik	TPD52L2;RP4-	Q5JWU6;Q5U	1,0964	0,4837327	428340	2	2	2
Uncharacterized protein MATR3;Matrin-3	MATR3;KIAA0	A8MXP9;P432	1,0542	0,4843697	3847801	7	7	7
Protein transport protein Sec24C;SEC24-related	SEC24C;KIAA0	P53992;B4DSI	1,0529	0,4855148	5623603	13	13	13
cDNA FLJ58953, highly similar to 40S ribosomal	RPS20	B4DW28;P60	1,0513	0,4869261	9359605	3	3	3

GDP-mannose 4,6 dehydratase;GDP-D-mannose	GMDS;RP1-11	O60547;B2R9	1,0925	0,4872529	427990,2	4	4	4
Protein disulfide-isomerase;Prolyl 4-hydroxylase	P4HB;ERBA2L	P07237;B2RD	1,0508	0,4873677	9510196	11	11	11
40S ribosomal protein S18;Ke-3	RPS18;D6S218	P62269;Q5SU	1,0489	0,4890475	5152798	9	9	9
Glycine cleavage system H protein, mitochondria	GCSH	P23434;Q53X	1,09	0,4895165	473280,2	2	2	2
Nuclear migration protein nudC;Nuclear distribu	NUDC;NPD01	Q9Y266;A8MU	1,0477	0,4901101	1,63E+07	11	11	11
DNA replication licensing factor MCM3;DNA poly	MCM3	P25205;B2R7	1,0466	0,4910854	1,62E+07	22	22	22
U6 snRNA-associated Sm-like protein LSm6	LSM6	P62312;Q4W5	1,0881	0,4912407	660050,2	3	3	3
cDNA FLJ31747 fis, clone NT2RI2007377, highly s	EWSR1;hCG_2	Q96MX4;Q01	1,0458	0,4917953	5078800	4	4	4
NAD(P) transhydrogenase, mitochondrial;Nicotir	NNT	Q13423;B4DN	1,0867	0,4925131	517970,2	6	6	6
Transcription elongation regulator 1;TATA box-b	TCERG1;CA15	O14776-1;O14	1,0447	0,4927724	4413099	9	9	9
Serine/arginine repetitive matrix protein 1;Ser/A	SRRM1;SRM1	Q8IYB3-2;Q8I	1,0857	0,493423	214080	2	2	2
Eukaryotic translation initiation factor 2 subunit	EIF2S2;EIF2B	P20042;B5BU	1,0856	0,493514	1324800	2	2	2
Proteasome-associated protein ECM29 homolog	ECM29;KIAA0	Q5VYK3;B3KV	1,0406	0,4964237	3592802	20	20	20
Afadin;Protein AF-6;Afadin variant	MLLT4;AF6;RF	P55196-5;P55	1,0824	0,496432	1155000	6	6	6
Putative pre-mRNA-splicing factor ATP-depende	DHX15;DBP1;	O43143;B4E0	1,0405	0,4965128	1,35E+07	20	20	19
Eukaryotic translation initiation factor 4 gamma	EIF4G2;DAP5;	P78344;B4DZ	1,0403	0,4966914	4588002	8	8	8
Calpain small subunit 1;Calcium-dependent prot	CAPNS1;CAPN	P04632;A8K0	1,0803	0,4983516	571749,9	3	3	3
Chromobox protein homolog 3;Heterochromatir	CBX3	Q13185;A4D1	1,0375	0,4991941	5042498	6	6	5
Vacuolar protein sorting-associated protein 35;V	VPS35;MEM3	Q96QK1;Q53F	1,0373	0,4993731	1,02E+07	17	17	17
SAM domain and HD domain-containing protein	SAMHD1;MOI	Q9Y3Z3;B4E2	1,0366	0,5	3929199	10	10	10
Thioredoxin domain-containing protein 1;Transn	TXNDC1;TMX;	Q9H3N1;B2R7	1,0785	0,5	1099000	6	6	6
40S ribosomal protein S16;cDNA FLJ56786, mod	RPS16	P62249;B2RD	1,0362	0,5003584	6032803	7	7	7
60S ribosomal protein L11;CLL-associated antige	RPL11;RP11-2	P62913-1;P62	1,0781	0,5003668	368499,8	2	2	2
Ras-related protein Rab-5C;RAB5L;L1880	RAB5C;RABL	P51148	1,0356	0,5008963	2924699	5	5	3
Signal peptidase complex subunit 2;Microsomal	SPCS2;KIAA01	Q15005;B4DD	1,0773	0,5011006	975369,8	5	5	5
Cyclin-dependent kinase inhibitor 2B-related pro	P15RS	Q96P16-1;Q9	1,077	0,5013758	574979,9	3	3	3
Sorting nexin-5;Sorting nexin 5	SNX5;RP11-50	Q9Y5X3;Q53F	1,0761	0,5022022	2113901	5	5	5
ADP-ribosylation factor-like protein 8B;ADP-ribo	ARL8B;ARL10	Q9NVJ2;B4E1	1,0755	0,5027535	474299,9	3	3	3
Pre-mRNA-processing-splicing factor 8;Splicing fa	PRPF8;PRPC8	Q6P2Q9;B4DK	1,0306	0,5053905	3503302	16	16	16
Vacuolar protein sorting-associated protein 29;V	VPS29;DC7;D	Q9UBQ0-2;Q9	1,0283	0,5074648	2394001	6	6	6
Hepatoma-derived growth factor-related protein	HDGFRP2;HD	Q7Z4V5-1;Q7	1,0685	0,5092076	524290,1	4	3	3
Heme oxygenase 2	HMOX2;HO2	P30519;A8MT	1,0676	0,5100403	381710,2	2	2	2
Plastin-3;T-plastin	PLS3	P13797;A7E2	1,0254	0,5100867	3,31E+07	25	25	22

Eukaryotic translation initiation factor 4B;cDNA I	EIF4B	P23588;B4DR	1,0675	0,5101329	840100,6	2	2	2
C-terminal-binding protein 2;C-terminal binding	CTBP2;RP11-5	P56545-2;P56	1,025	0,510449	4251602	5	5	3
Ras-related protein Rab-7a	RAB7A;RAB7	P51149;B4DP	1,0222	0,5129881	3699701	9	9	9
RNA-binding protein 12;RNA-binding motif prote	RBM12;KIAA0	Q9NTZ6;B3KC	1,0189	0,5159888	6860303	9	9	9
Nucleophosmin;Nucleolar phosphoprotein B23;I	NPM1;NPM	P06748-1;P06	1,0186	0,516262	1,98E+07	7	7	7
DNA polymerase;DNA polymerase alpha catalyti	POLA1;POLA	A6NMQ1;P09	1,0601	0,5170052	1480701	8	8	8
Uncharacterized protein KIAA0152	KIAA0152	Q14165	1,0599	0,5171915	430229,9	3	3	3
Eukaryotic translation initiation factor 4H;Williar	EIF4H;KIAA00	Q15056-1;Q1	1,0592	0,5178439	2203500	6	6	6
Acetyl-CoA acetyltransferase, cytosolic;Cytosolic	ACAT2;ACTL	Q9BWD1;A8K	1,0592	0,5178439	965210,2	4	4	4
Vigilin;High density lipoprotein-binding protein;c	HDLBP;HBP;V	Q00341;B2R5	1,015	0,5195465	1,49E+07	25	25	25
Protein flightless-1 homolog;cDNA FLJ50164, hig	FLII;FLIL	Q13045;B4DI	1,0562	0,5206444	952640,4	3	3	3
Translin	TSN	Q15631;B3KR	1,0121	0,5221998	2607100	11	11	11
E3 ubiquitin-protein ligase BRE1B;RING finger pr	RNF40;BRE1B	O75150-1;O7	1,0533	0,5233582	189920	3	2	2
Fatty acid synthase;[Acyl-carrier-protein] S-acety	FASN;FAS	P49327;Q13	1,01	0,5241254	9,98E+07	91	91	91
Heterogeneous nuclear ribonucleoprotein L;Unc	HNRNPL;HNRI	P14866;B2R9	1,0093	0,524768	6075199	6	6	3
DNA replication licensing factor MCM5;CDC46 h	MCM5;CDC46	P33992;A8K5	1,0075	0,5264221	1,51E+07	24	24	24
Splicing factor, arginine/serine-rich 9;Pre-mRNA-	SFRS9;SRP30C	Q13242;A8K3	1,0499	0,5265479	669969,9	4	4	4
Diablo homolog, mitochondrial;Second mitocho	DIABLO;SMAC	Q9NR28-1;Q9	1,0496	0,5268298	597579,9	2	2	2
DNA replication licensing factor MCM2;Minichro	MCM2;BM28;	P49736;B3KX	1,0063	0,5275263	2,45E+07	24	24	24
Sodium/potassium-transporting ATPase subunit	ATP1B3	P54709	1,006	0,5278025	1,36E+07	8	8	8
Protein DEK;cDNA FLJ53031, highly similar to Pr	DEK	P35659;B2R6I	1,0051	0,5286316	3184300	5	5	5
GrpE protein homolog 1, mitochondrial;Mt-GrpE	GRPEL1;GREP	Q9HAV7;B2R7	1,0476	0,5287106	1666701	8	8	8
Voltage-dependent anion-selective channel prot	VDAC3	Q9Y277-2;Q9	1,003	0,5305684	3705198	4	4	4
THO complex subunit 2;cDNA FLJ61096, highly si	THOC2;CXorf3	Q8NI27-2;Q8	1,045	0,5311602	1752100	3	3	3
Voltage-dependent anion-selective channel prot	VDAC2;RP11-	P45880-3;P45	1,0023	0,5312148	1,42E+07	9	9	9
Phenylalanyl-tRNA synthetase beta chain;Phenyl	FARSB;FARSLE	Q9NSD9;A8K6	1,0019	0,5315844	1,12E+07	14	14	14
Histone H2B type 1-N;H2B.d;H2B/d;cDNA FLJ56	HIST1H2BN;H	Q99877;A8K9	1,0436	0,5324812	1418700	4	4	4
Receptor expression-enhancing protein 5;Polyp	REEP5;C5orf1	Q00765;B2R6	1,0434	0,5326699	516510,3	2	2	2
Transcription elongation factor SPT4;DRB sensiti	SUPT4H1;SPT	P63272;B2R4	1,042	0,5339925	362810,1	2	2	2
Kinesin-like protein KIF14	KIF14;KIAA00	Q15058	1,0416	0,5343707	1509599	2	2	2
cDNA FLJ76830, highly similar to Homo sapiens S	HLTF;HIP116A	A8K5B6;Q145	1,0411	0,5348435	329170,2	2	2	2
cAMP-dependent protein kinase catalytic subuni	PRKACB;RP11	P22694-2;P22	1,0406	0,5353166	1646101	3	3	3
Eukaryotic translation initiation factor 3 subunit	EIF3A;EIF3S10	Q14152;B1AN	0,99714	0,5359908	2,50E+07	45	45	45

Ran GTPase-activating protein 1	RANGAP1;KIA	P46060;B4DY	0,99713	0,5360001	1,19E+07	13	13	13
Lupus La protein;Sjoegren syndrome type B anti	SSB	P05455;B5BU	0,99636	0,5367144	2,38E+07	19	19	19
Tyrosine-protein phosphatase non-receptor typ	PTPN1;PTP1B	P18031;A8K3	1,0374	0,5383481	625470,1	3	3	3
40S ribosomal protein S28	RPS28	P62857;B2R4	1,0365	0,539202	2285301	2	2	2
26S proteasome non-ATPase regulatory subunit	PSMD4;MCB1	P55036-2;P55	0,99365	0,5392321	5598900	7	7	5
Proteasome assembly chaperone 1;Down syndr	PSMG1;C21LR	O95456-1;O9	1,0359	0,5397716	483699,9	3	3	3
40S ribosomal protein S25	RPS25	P62851;B2R4	0,99256	0,5402463	3858402	3	3	3
Tropomyosin alpha-3 chain;Tropomyosin-3;Gam	TPM3;RP11-2	P06753-2;P06	0,99229	0,5404976	8720196	11	11	10
Protein diaphanous homolog 1;Diaphanous-rela	DIAPH1;DIAP1	O60610-1;O6	0,99201	0,5407584	3676599	12	12	12
ATP-dependent RNA helicase DDX42;DEAD box	DDX42	Q86XP3-1;Q8	1,0346	0,5410065	376069,9	2	2	2
PEST proteolytic signal-containing nuclear prote	PCNP	Q8WW12-1;Q	1,0344	0,5411966	709970,2	3	3	3
Eukaryotic translation initiation factor 2 subunit	EIF2S1;EIF2A	P05198;Q53X	0,98946	0,5431353	4799397	12	12	10
Eukaryotic translation initiation factor 4 gamma	EIF4G1;EIF4F;	Q04637-5;Q0	0,98944	0,5431539	1,52E+07	25	25	22
Activating signal cointegrator 1 complex subunit	ASCC3;HELIC1	Q8N3C0;B4DF	1,0311	0,5443371	683129,6	3	3	3
Glycogen phosphorylase, brain form	PYGB	P11216;B4DS	1,031	0,5444324	778410,4	7	3	3
Lipopolysaccharide-responsive and beige-like an	LRBA;BGL;CD	P50851;B3KV	1,0303	0,5450995	143340	2	2	2
Cytochrome c1, heme protein, mitochondrial;Ub	CYC1	P08574;Q8TB	0,98702	0,5454139	2903601	6	6	6
Casein kinase 2, beta polypeptide (Casein kinase	CSNK2B;DADE	Q5SRQ6;P678	0,98683	0,5455915	3351400	4	4	4
Ras-related protein Rab-14	RAB14	P61106;B3KR	1,029	0,5463393	501050	3	3	3
Eukaryotic translation initiation factor 4 gamma	EIF4G3	O43432-1;O4	1,0269	0,5483445	186910	5	2	2
High mobility group protein B2;High mobility gr	HMGB2;HMG	P26583;B2R4	1,0259	0,5493004	1454000	6	5	5
Methionine adenosyltransferase 2 subunit beta;	MAT2B;TGR;	Q9NZL9-1;Q9	1,0256	0,5495874	2274201	4	4	4
116 kDa U5 small nuclear ribonucleoprotein con	EFTUD2;KIAA	Q15029;A8KA	0,98136	0,5507154	1,01E+07	16	15	15
60S acidic ribosomal protein P0;L10E;cDNA FLJ5	RPLP0	P05388;A8K4	1,0226	0,5524593	593600,4	3	3	3
Protein kinase C and casein kinase substrate in n	PACSIN2;AL04	Q9UNF0-1;Q9	1,0223	0,5527469	1514501	4	4	4
Polyadenylate-binding protein 1;cDNA FLJ59219	PABPC1;PAB1	P11940-1;P11	0,97902	0,5529136	8488097	8	8	8
26S proteasome non-ATPase regulatory subunit	PSMD10;RP5-	O75832;Q5U	1,0208	0,5541853	376269,8	2	2	2
Eukaryotic translation initiation factor 3 subunit	EIF3J;EIF3S1;P	O75822;B4DU	0,977	0,5548141	2771301	7	7	7
Importin subunit beta-1;Karyopherin subunit be	KPNB1;NTF97	Q14974;B2RB	0,97695	0,5548612	6,36E+07	29	29	29
Acyl carrier protein, mitochondrial;NADH-ubiqui	NDUFAB1	O14561;A4U	1,0192	0,5557212	106990	2	2	2
Serine/threonine-protein kinase MRCK beta;CD	CDC42BPB;KI	Q9Y5S2;A9JR	1,0192	0,5557212	223330,1	3	3	3
Actin-related protein 2;Actin-like protein 2;cDN	ACTR2;ARP2	P61160;B2RC	0,97592	0,5558312	4288398	6	6	6
Nodal modulator 2;pM5 protein 2;Nodal modul	NOMO2;NOM	Q5JPE7-1;Q5J	0,9759	0,5558501	3790399	9	9	9

Uncharacterized protein CD81;CD81 antigen;26	CD81;TAPA1;T	A6NMH8;P60	1,0189	0,5560093	238320,1	2	2	2
Nuclear autoantigenic sperm protein isoform 2 v	NASP;RP4-697	Q53H03;Q5T6	0,97565	0,5560857	2,60E+07	18	18	18
Glucosamine-6-phosphate isomerase 1;Glucosar	GNPDA1;GNP	P46926;A8K3	1,0178	0,5570664	506759,9	3	3	3
60 kDa SS-A/Ro ribonucleoprotein;Ro 60 kDa au	TROVE2;RO60	P10155-1;P10	0,97401	0,557632	3288400	6	6	6
Far upstream element-binding protein 3	FUBP3;FBP3	Q96I24-1;Q96	1,0165	0,5583166	1957100	5	5	5
Polyribonucleotide nucleotidyltransferase 1, mit	PNPT1;PNPAS	Q8TCS8	1,0162	0,5586052	1004601	3	3	3
Cytochrome b5;Uncharacterized protein CYB5A	CYB5A;CYB5	P00167-1;P00	1,0161	0,5587014	454499,9	2	2	2
Acetyl-CoA carboxylase 1;ACC-alpha;Biotin carb	ACACA;ACAC;	Q13085-4;Q1	0,97277	0,5588024	8,38E+07	81	81	81
Microtubule-associated protein 4	MAP4	P27816-1;P27	0,97191	0,5596146	1,39E+07	19	19	19
Eukaryotic translation initiation factor 2 subunit	EIF2S3;EIF2G	P41091;A8K2	0,97151	0,5599926	9491505	12	12	12
Fatty acyl-CoA reductase 1;Male sterility domair	FAR1;MLSTD2	Q8WVX9;B2R	1,0147	0,5600492	877320,5	3	3	3
AP-1 complex subunit gamma-1;Adapter-related	AP1G1;ADTG;	O43747;B3KX	0,97002	0,5614014	8088395	12	12	12
Putative ATP-dependent Clp protease proteolyti	CLPP	Q16740;A4UC	1,0131	0,561591	286359,9	3	3	3
Cytochrome c oxidase subunit 2;Cytochrome c o	MT-CO2;COII;	P00403;A0S0	1,0131	0,561591	963469,9	3	3	3
Calcium-binding mitochondrial carrier protein S	SLC25A24;AP	Q6NUK1-1;Q6	1,0131	0,561591	600369,8	2	2	2
Rab3 GTPase-activating protein non-catalytic su	RAB3GAP2;KI	Q9H2M9-1;Q	1,0129	0,5617838	1918201	8	8	8
Eukaryotic initiation factor 4A-II;ATP-dependent	EIF4A2;DDX2	Q14240-2;Q1	1,0118	0,5628448	499349,8	12	3	3
Actin-related protein 2/3 complex subunit 2;Arp	ARPC2;ARC34	O15144;Q53R	0,96762	0,5636734	5420001	8	8	8
Protein disulfide-isomerase A6;Protein disulfide	PDIA6;TXNDC	Q15084-2;Q1	0,96724	0,5640335	2,76E+07	11	11	11
CDGSH iron sulfur domain-containing protein 2;	CISD2;CDGSH	Q8N5K1	1,0097	0,5648723	362919,9	3	3	3
NADH dehydrogenase [ubiquinone] 1 alpha subc	NDUFA13;GRI	Q9P0J0;B4DF	1,0095	0,5650654	1605301	4	4	4
Myosin-10;Myosin heavy chain 10;Myosin heav	MYH10	P35580-3;P35	0,96614	0,5650764	1,36E+07	47	36	31
Alpha-aminoadipic semialdehyde dehydrogenas	ALDH7A1;ATC	P49419;B2R6	1,0085	0,5660319	855249,6	2	2	2
40S ribosomal protein S19	RPS19	P39019;B0ZB	0,96445	0,5666799	3143102	7	7	7
Mitochondrial carrier homolog 2;Met-induced m	MTCH2;MIMP	Q9Y6C9;B2R7	1,0072	0,5672891	657100,3	3	3	3
DCC-interacting protein 13-alpha;Adapter protei	APPL1;APPL;D	Q9UKG1;B4D	1,0046	0,5698063	453900	2	2	2
Cytosol aminopeptidase;Leucine aminopeptidas	LAP3;LAPEP;P	P28838-1;P28	0,96064	0,5703013	6047904	10	10	10
Chromodomain-helicase-DNA-binding protein 4;	CHD4	Q14839-2;Q1	1,0034	0,5709694	1542200	8	8	8
40S ribosomal protein S3	RPS3;OK/SW-	P23396;B2R7	0,95964	0,5712532	2,08E+07	13	13	13
Histone-binding protein RBBP7;Retinoblastoma-	RBBP7;RBAP4	Q16576;Q5JN	0,9583	0,5725296	4,12E+07	13	13	8
40S ribosomal protein S7;Uncharacterized prote	RPS7	P62081;Q57Z	1,0014	0,5729096	1660301	4	4	4
Uncharacterized protein KIAA0564	KIAA0564	A3KMH1-1;A3	1,0013	0,5730066	214010,1	2	2	2
Proteasome subunit alpha type-4;Proteasome c	PSMA4;PSC9	P25789;B2RD	0,95722	0,5735591	9832403	9	9	9

Developmentally-regulated GTP-binding protein	DRG1;NEDD3	Q9Y295;B2RD	0,99704	0,5771463	1407299	5	5	5
Nucleosome assembly protein 1-like 4;Nucleoso	NAP1L4;NAP2	Q99733;A8M	0,95344	0,5771677	1,71E+07	8	8	7
Structural maintenance of chromosomes protei	SMC4;CAPC;S	Q9NTJ3-1;Q9	0,95332	0,5772824	3983402	15	15	15
Vesicular integral-membrane protein VIP36;Lect	LMAN2;C5orf	Q12907;A8K7	0,99658	0,5775939	1003100	3	3	3
Large proline-rich protein BAT3;HLA-B-associate	BAT3;G3;DAD	P46379-3;P46	0,95283	0,5777507	3077301	6	6	6
Vesicle-trafficking protein SEC22b;SEC22 vesicle-	SEC22B;SEC22	O75396;A8K1	0,99573	0,5784212	812740,3	4	4	4
cDNA FLJ60015, highly similar to Adapter-relate	AP1S2;DC22;	B4DIM6;P563	0,99469	0,5794339	368380	2	2	2
40S ribosomal protein S11	RPS11	P62280;B2R4	0,95059	0,5798936	2568302	8	8	4
cDNA FLJ53425, highly similar to Far upstream e	FUBP1	B4DT31;A6NL	0,94906	0,5813588	8711496	11	11	11
ATP-dependent DNA helicase 2 subunit 2;ATP-de	XRCC5;G22P2	P13010;Q53T	0,94879	0,5816175	3,51E+07	28	28	28
Sigma 1-type opioid receptor;SR31747-binding p	OPRS1;SIGMA	Q99720-1;Q9	0,99227	0,5817925	876369,9	3	3	3
cDNA FLJ50661, highly similar to Dihydrolipoyl d	DLD;GCSL;LAD	B4DT69;P096	0,94824	0,5821446	6760104	9	9	9
cDNA FLJ44241 fis, clone THYMU3008436, highl	PFKM;PFKX	Q6ZTT1;P082	0,99152	0,582524	2300300	9	9	7
Transmembrane emp24 domain-containing prot	TMED2;RNP2	Q15363;Q6FH	0,98984	0,5841636	818380,4	2	2	2
26S proteasome non-ATPase regulatory subunit	PSMD6;KIAA0	Q15008;A8K2	0,94111	0,5889914	9877496	16	16	16
Myosin-9;Myosin heavy chain 9;Myosin heavy c	MYH9	P35579-1;P35	0,94061	0,5894725	5,17E+07	74	74	59
Gem-associated protein 5	GEMIN5	Q8TEQ6;Q58	0,98436	0,5895209	1645501	8	8	8
Cullin-4B;Uncharacterized protein CUL4B (Cullin	CUL4B;KIAA0	Q13620-2;Q1	0,9401	0,5899634	6757503	15	15	11
NADH dehydrogenase [ubiquinone] 1 alpha subc	NDUFA5;DKF	Q16718;B2RD	0,98378	0,5900887	795139,9	2	2	2
Chromosome-associated kinesin KIF4A;Chromos	KIF4A;KIF4;KI	O95239-1;O9	0,9823	0,5915382	459310	4	4	4
Replication factor C subunit 3;Activator 1 subuni	RFC3	P40938;B4DK	0,97862	0,5951464	224470	3	3	3
40S ribosomal protein S3a;Uncharacterized prot	RPS3A;MFTL	P61247;A8K4	0,93453	0,5953321	8682200	12	12	12
Serine/threonine-protein kinase Nek9;Never in	NEK9;KIAA19	Q8TD19;B3KY	0,97793	0,5958235	391880,3	2	2	2
UPF0389 protein FAM162A;E2-induced gene 5 p	FAM162A;C3	Q96A26;B4DF	0,97744	0,5963045	876400,1	3	3	3
Guanine nucleotide binding protein (G protein),	GNB2L1	Q53HU2;Q5V	0,93332	0,5965003	3,84E+07	19	19	19
S-adenosylmethionine synthetase isoform type-	MAT2A;AMS2	P31153;A8K5	0,93277	0,5970315	7791104	10	10	10
40S ribosomal protein S10;Ribosomal protein S1	RPS10;RPS10	P46783;B2R4	0,93234	0,5974469	2731500	5	5	5
Filamin B;Filamin-B;Beta-filamin;Actin-binding-li	FLNB;FLN1L;F	B2ZZ83;O753	0,93193	0,5978431	1,10E+07	52	50	47
YTH domain family protein 2;High-glucose-regul	YTHDF2;HGR	Q9Y5A9-1;Q9	0,9312	0,5985486	6630598	5	5	5
Uncharacterized protein FAM98B;Protein FAM9	FAM98B	A8MUW5;Q5	0,93079	0,598945	3785601	7	7	7
Vacuolar protein sorting-associated protein 26A	VPS26A;VPS2	O75436;A8K3	0,92991	0,5997959	3715598	8	8	8
Proteasome subunit alpha type-7;Proteasome s	PSMA7;RP5-1	O14818-1;O1	0,92969	0,6000087	1,89E+07	12	12	8
Thioredoxin domain-containing protein 9;Protei	TXNDC9;APA	O14530;B2R9	0,97261	0,6010508	432109,9	2	2	2

Protein transport protein Sec23A;SEC23-related	SEC23A	Q15436;B2R5	0,92716	0,6024573	4230900	9	9	6
26S proteasome non-ATPase regulatory subunit	PSMD2;TRAP2	Q13200;B4DN	0,92675	0,6028544	1,94E+07	22	22	22
Lamin-A/C;70 kDa lamin;Renal carcinoma antige	LMNA;LMN1;I	P02545-1;P02	0,97073	0,6029005	340489,9	2	2	2
WD40 repeat-containing protein SMU1;Smu-1 su	SMU1	Q2TAY7;A0MI	0,97013	0,6034911	1471101	3	3	3
Cytochrome P450 51A1;CYPLI;P450LI;Sterol 14-a	CYP51A1;CYP5	Q16850;A4D1	0,9682	0,6053918	489689,7	2	2	2
Microsomal glutathione S-transferase 3 variant;I	MGST3;RP11-	Q53GB9;Q5VV	0,96769	0,6058943	112050	2	2	2
Filamin-A;Alpha-filamin;Filamin-1;Endothelial ac	FLNA;FLN;FLN	P21333-1;P21	0,92319	0,6063049	1,77E+07	55	55	53
Membrane-associated progesterone receptor cc	PGRMC2;DG6	O15173	0,96727	0,6063081	435150,1	4	3	3
SRA stem-loop-interacting RNA-binding protein,	SLIRP;C14orf1	Q9GZT3	0,92281	0,6066735	2477802	7	7	7
Ran-specific GTPase-activating protein;Ran-bind	RANBP1	P43487;Q53EY	0,92277	0,6067123	8404304	6	6	6
60S ribosomal protein L24;Ribosomal protein L3	RPL24;RP1-12	P83731;B2R4Y	0,96643	0,607136	500300,1	3	3	3
Trifunctional enzyme subunit beta, mitochondria	HADHB;MSTP	P55084;B2RB	0,92224	0,6072265	1,25E+07	11	11	11
Proteasome subunit alpha type-5;Proteasome ze	PSMA5	P28066;B2R8I	0,9212	0,6082359	1,98E+07	10	10	10
AP-2 complex subunit beta-1;Adapter-related pr	AP2B1;ADTB2	P63010-2;P63	0,9207	0,6087213	2526902	14	6	6
40S ribosomal protein S15;RIG protein	RPS15;RIG	P62841;A5D8	0,96404	0,6094929	1289701	2	2	2
Importin subunit alpha-2;Karyopherin subunit al	KPNA2;RCH1;S	P52292;A8K7I	0,91931	0,6100712	8619605	8	8	8
Glutamine synthetase;Glutamate--ammonia liga	GLUL;GLNS	P15104;A1L19	0,96208	0,6114271	1589200	5	5	5
Uncharacterized protein ENSP00000352682;40S	RPS17	A6NH77;P087	0,91727	0,6120537	4261101	4	4	4
Activated RNA polymerase II transcriptional coa	SUB1;PC4;RP	P53999;Q59G	0,91725	0,6120731	2490400	3	3	3
Serine-threonine kinase receptor-associated pro	STRAP;MAWD	Q9Y3F4;B0AZ	0,91678	0,6125301	9044900	12	12	12
LRRC15 protein (HCG2043616) (Leucine rich rep	LRRC15;hCG_	Q495Q6;Q8TF	0,96063	0,6128588	407699,8	3	3	3
Transportin-1;Importin beta-2;Karyopherin beta	TNPO1;KPNB2	Q92973;B4DV	0,91586	0,6134248	1,78E+07	16	16	16
3-hydroxyacyl-CoA dehydrogenase type-2;3-hyd	HSD17B10;ER	Q99714-1;Q9	0,91565	0,613629	7134799	15	15	15
Synaptic vesicle membrane protein VAT-1 homo	VAT1	Q99536;A8K3	0,91537	0,6139014	3984102	6	6	6
Trifunctional enzyme subunit alpha, mitochondr	HADHA;HADH	P40939;B2R7I	0,91382	0,6154097	7281398	13	13	13
Ras-related protein Rab-2A;Ras-related protein F	RAB2A;RAB2;I	P61019;B2R5V	0,95792	0,6155361	565760,1	3	3	3
Acyl-CoA dehydrogenase family member 9, mito	ACAD9;hCG_2	Q9H845;Q59F	0,95586	0,6175727	1464600	4	4	4
Capping protein (Actin filament) muscle Z-line, b	CAPZB;RP4-65	B1AK88;B4DV	0,91149	0,6176786	3566301	9	9	9
Actin-related protein 3;Actin-like protein 3	ACTR3;ARP3	P61158;B4DT	0,9114	0,6177662	6746702	6	6	6
Adenosylhomocysteinase;S-adenosyl-L-homocys	AHCY;SAHH	P23526;A8K3	0,91137	0,6177955	6,36E+07	17	17	17
Sister chromatid cohesion protein PDS5 homolo	PDS5A;KIAA0	Q29RF7-1;Q2	0,95439	0,6190267	780639,5	4	4	4
Exportin-1;Chromosome region maintenance 1 p	XPO1;CRM1	O14980;B3KW	0,90851	0,6205829	2,92E+07	30	30	30
Histone H4	HIST1H4A;H4/	P62805;B2R4I	0,95279	0,6206099	1836599	3	3	3

cDNA FLJ56155, highly similar to UTP--glucose-1	UGP2;UGP1	B4DUP2;Q16	0,95269	0,6207089	1788699	5	5	5
Isocitrate dehydrogenase [NAD] subunit alpha, n	IDH3A	P50213-1;P50	0,90716	0,6218996	5183299	7	7	7
COP9 signalosome complex subunit 1;JAB1-cont	GPS1;COPS1;C	Q13098-1;Q1	0,90622	0,6228166	3063001	6	6	6
Isocitrate dehydrogenase [NAD] subunit beta, m	IDH3B	O43837-1;O4	0,95047	0,6229068	2326702	9	9	9
Sorbitol dehydrogenase;L-iditol 2-dehydrogenas	SORD	Q00796;B2R6	0,90572	0,6233045	2799701	7	7	7
Ras-related protein Rab-10	RAB10	P61026;Q53S	0,94963	0,6237386	273640,1	2	2	2
E3 ubiquitin-protein ligase BRE1A;RING finger pr	RNF20;BRE1A	Q5VTR2;A0PJ	0,94853	0,6248283	678149,8	3	3	2
AP-3 complex subunit beta-1;Adapter-related pr	AP3B1;ADTB3	O00203-1;O0	0,90379	0,6251886	4838099	14	14	14
Uncharacterized protein CARM1 (Coactivator-as	CARM1;hCG_	A6NN38;Q86	0,90374	0,6252374	3479199	5	5	5
Uncharacterized protein ARPC3;Actin-related pr	ARPC3;ARC21	A8MYY8;O15	0,94662	0,626721	1499201	7	7	7
General transcription factor II-I;Bruton tyrosine	GTF2I;BAP135	P78347-1;P78	0,90177	0,6271616	2,56E+07	26	26	26
40S ribosomal protein S21;Ribosomal protein S2	RPS21;RP5-90	P63220;Q6FG	0,90109	0,627826	2658102	5	5	5
Mitochondrial import receptor subunit TOM22 h	TOMM22;TOM	Q9NS69;Q53	0,90019	0,6287056	4943498	3	3	3
Mitotic checkpoint protein BUB3;Uncharacterize	BUB3;hCG_41	O43684;B2R6	0,90015	0,6287447	8930498	8	8	8
CDGSH iron sulfur domain-containing protein 1;I	CISD1;C10orf	Q9NZ45	0,94308	0,6302308	546949,6	2	2	2
Stromal antigen 2 (Stromal antigen 2, isoform C	STAG2;RP11-5	B1AMT5;Q68	0,94262	0,630687	556209,9	5	5	5
UPF0368 protein Cxorf26;Uncharacterized prote	CXorf26	Q9BVG4;Q9P	0,94222	0,6310838	854910,5	3	3	3
Echinoderm microtubule-associated protein-like	EML4;C2orf2;	Q9HC35;B2RE	0,94221	0,6310937	879720,1	3	3	3
Transmembrane emp24 domain-containing prot	TMED9;GP25	Q9BVK6;B2R8	0,94138	0,6319171	1401999	3	3	3
Sorting nexin-2;Transformation-related gene 9 p	SNX2;TRG9	O60749;B3KM	0,9408	0,6324926	1332300	2	2	2
Protein transport protein Sec31A;SEC31-related	SEC31A;KIAA	O94979-8;O9	0,89384	0,634917	1,09E+07	15	15	15
40S ribosomal protein S2;S4;LLRep3 protein;Unc	RPS2;RPS4	P15880;B2R5	0,89361	0,6351422	4411798	5	5	5
cDNA FLJ54534, highly similar to Homo sapiens	DKFZp686F16	B4DPV7;Q5HY	0,89261	0,6361213	2594299	7	7	7
Proteasome activator complex subunit 2;Protea	PSME2	Q9UL46;A8M	0,93659	0,6366713	440959,9	5	5	5
Ankyrin repeat and FYVE domain-containing pro	ANKFY1;ANKH	Q9P2R3-1;Q9	0,93632	0,6369393	2366201	5	5	5
Protein phosphatase 1 regulatory subunit 7;Prot	PPP1R7;SDS2	Q15435-1;Q1	0,93587	0,6373862	1227600	3	3	3
Sorting nexin-6;TRAF4-associated factor 2;cDNA	SNX6;hCG_20	Q9UNH7;A8K	0,93503	0,6382204	1684799	6	6	6
Heterogeneous nuclear ribonucleoprotein Q;Syn	SYNCRIP;HNR	O60506-1;O6	0,89026	0,6384229	3522302	7	7	7
UPF0527 transmembrane protein;Hydrophobic	DC2;HDCMD4	Q9NRP0;B2R5	0,93467	0,6385779	394560,2	2	2	2
Endoplasmin;Heat shock protein 90 kDa beta m	HSP90B1;TRA	P14625;B4DH	0,88968	0,6389911	9,07E+07	40	39	39
Cytochrome c oxidase subunit 4 isoform 1, mito	COX4I1;COX4	P13073;B2R4	0,88928	0,639383	5526103	5	5	5
cDNA FLJ56442, highly similar to ATP-citrate syn	ACLY variant	B4DIM0;B4E3	0,88815	0,6404904	1,23E+08	45	45	2
HIV Tat-specific factor 1;cDNA FLJ60139, highly	HTATSF1;RP1-	O43719;B4DR	0,93274	0,6404949	1647100	3	3	3

Putative uncharacterized protein DKFZp779B024	DKFZp779B02	Q68DF1;O755	0,88767	0,6409608	1,64E+07	24	24	24
Uncharacterized protein KIAA0090;KIAA0090 (U	KIAA0090;PSE	Q8N766-1;Q8	0,8874	0,6412255	2986099	4	4	4
40S ribosomal protein S9;RPS9 protein	RPS9	P46781;A9C4	0,88733	0,641294	3547102	9	9	9
RNA (guanine-9-)-methyltransferase domain-cor	RG9MTD1	Q7L0Y3	0,93126	0,6419652	1851999	6	6	6
AP1B1 protein;AP-1 complex subunit beta-1;Ada	AP1B1;ADTB1	Q86X54;Q105	0,886	0,6425979	1,12E+07	15	15	7
BTB/POZ domain-containing protein KCTD12;Pfe	KCTD12;C13o	Q96CX2;B3KY	0,88578	0,6428136	5975104	11	11	11
Tyrosyl-tRNA synthetase, cytoplasmic;Tyrosyl--t	YARS	P54577;B3KW	0,88545	0,6431371	4,09E+07	26	26	26
Coatomer subunit alpha;Alpha-coat protein;HEP	COPA	P53621-1;P53	0,88504	0,6435391	5,28E+07	47	47	47
Kinesin-1 heavy chain;Ubiquitous kinesin heavy	KIF5B;KNS;KN	P33176;A8K0	0,88478	0,6437941	8293004	19	19	19
Cytoplasmic FMR1-interacting protein 1;Specific	CYFIP1;KIAA0	Q7L576-1;Q7	0,88421	0,644353	2,58E+07	28	28	15
Junction plakoglobin;Desmoplakin-3;Desmoplak	JUP;CTNNG;D	P14923;Q7KZ	0,92789	0,645314	299910	3	3	2
U6 snRNA-associated Sm-like protein LSm2;snRN	LSM2;C6orf28	Q9Y333;Q6FG	0,9271	0,6460991	726410,1	2	2	2
26S proteasome non-ATPase regulatory subunit	PSMD1	Q99460-1;Q9	0,88199	0,6465305	1,93E+07	26	26	26
Peroxiredoxin-4;Prx-IV;Thioredoxin peroxidase A	PRDX4	Q13162	0,88185	0,6466679	1,41E+07	13	12	12
26S protease regulatory subunit 6B;Proteasome	PSMC4;MIP22	P43686-1;P43	0,87853	0,6499256	1,70E+07	13	13	13
AP-2 complex subunit alpha-2;Adapter-related p	AP2A2;ADTAB	O94973-2;O9	0,87819	0,6502594	4121999	8	8	4
26S protease regulatory subunit S10B;Proteason	PSMC6;SUG2	P62333;B2R9	0,87732	0,6511133	1,23E+07	15	15	15
Uncharacterized protein VAPA;Vesicle-associate	VAPA;VAP33	A6NDZ0;A8M	0,92098	0,6521831	1426300	5	5	4
Protein transport protein Sec23B;SEC23-related	SEC23B;RP11-	Q15437;B4DJ	0,92051	0,6526504	318600,1	5	2	2
Uncharacterized protein NUDT5;ADP-sugar pyro	NUDT5;HSPC1	A6NFX8;Q9U	0,87573	0,6526742	2560101	8	8	8
Actin-related protein 2/3 complex subunit 1B;Ar	ARPC1B;ARC4	O15143;A4D2	0,92038	0,6527796	393720,2	2	2	2
6-phosphofructokinase type C;Phosphofructokin	PFKP;PFKF;PF	Q01813;Q5VS	0,92029	0,6528691	1331001	6	5	5
Putative RNA-binding protein Luc7-like 2	LUC7L2;CGI-5	Q9Y383-1;Q9	0,87548	0,6529196	4332500	8	8	8
40S ribosomal protein S4, X isoform;Single copy	RPS4X;CCG2;	P62701;B2R4	0,87548	0,6529196	2971999	12	12	12
Transmembrane emp24 domain-containing prot	TMED10;TMP	P49755;B2R6	0,8746	0,6537836	2884799	4	4	4
AP-3 complex subunit delta-1;Adapter-related p	AP3D1;PRO0	O14617-5;O1	0,87304	0,6553154	4132503	10	10	10
40S ribosomal protein S13	RPS13	P62277;B2R5	0,87246	0,655885	2482500	5	5	5
Proteasome subunit alpha type-2;Proteasome c	PSMA2;PSC3	P25787;Q53G	0,87142	0,6569063	5832902	8	8	8
Signal recognition particle 72 kDa protein	SRP72	O76094;A0PJ	0,87065	0,6576626	4027902	9	9	9
Protein KIAA1967;Deleted in breast cancer gene	KIAA1967;DB	Q8N163-1;Q8	0,91473	0,6583975	1530400	3	3	3
Cation-independent mannose-6-phosphate rece	IGF2R;MPRI	P11717;A0N9	0,91436	0,6587654	650640,3	7	7	7
Calnexin;Major histocompatibility complex class	CANX	P27824;B2R5	0,86943	0,6588609	6,05E+07	19	19	19
Ubiquilin-2;Protein linking IAP with cytoskeleton	UBQLN2;N4B	Q9UHD9;Q5D	0,86912	0,6591653	2586299	3	3	3

Proteasome subunit beta type-1;Proteasome co	PSMB1;PSC5	P20618;B5BU	0,86892	0,6593618	9846400	9	9	9
CCR4-NOT transcription complex subunit 1;Nega	CNOT1;CDC39	A5YKK6-1;A5Y	0,91343	0,6596901	680349,9	5	5	5
ATP-dependent RNA helicase DDX3X;DEAD box p	DDX3X;DBX;D	O00571;A8K5	0,86841	0,6598628	2,66E+07	21	20	19
Secretory carrier-associated membrane protein	SCAMP3;C1or	O14828-1;O14	0,91306	0,660058	630529,7	2	2	2
Arginyl-tRNA synthetase, cytoplasmic;Arginine--t	RARS	P54136-1;P54	0,86809	0,6601771	4411903	13	13	13
Protein arginine N-methyltransferase 5;Shk1 kin	PRMT5;HRMT	O14744;B2RD	0,86758	0,6606781	2,30E+07	18	18	18
COP9 signalosome complex subunit 4;JAB1-cont	COPS4;CSN4	Q9BT78;B3KM	0,86755	0,6607075	5122403	9	9	9
Replication factor C subunit 4;Activator 1 subuni	RFC4;hCG_20	P35249;B4DM	0,91213	0,6609827	143609,9	2	2	2
Nucleoplasmin-3	NPM3	O75607	0,91082	0,6622851	629430,3	2	2	2
Nuclear pore membrane glycoprotein 210	NUP210;KIAA	Q8TEM1-1;Q8	0,91037	0,6627325	251860	2	2	2
Proteasome subunit beta type-5;Proteasome ep	PSMB5;LMPX	P28074;B2R4I	0,86511	0,6631045	1,15E+07	10	10	10
cDNA FLJ75633, highly similar to Homo sapiens s	SCFD1;C14orf	A8K2Z5;A8MV	0,9093	0,6637963	1504699	3	3	3
Quinone oxidoreductase;NADPH:quinone reduct	CRYZ	Q08257;A6NP	0,90879	0,6643033	482379,9	3	3	3
Neutral amino acid transporter B(0);Sodium-dep	SLC1A5;ASCT2	Q15758;A8K9	0,86358	0,6646076	7448202	6	6	6
NADH-cytochrome b5 reductase 3;Diaphorase-1	CYB5R3;DIA1;	P00387-1;P00	0,90806	0,665029	1184700	3	3	3
Actin-related protein 2/3 complex subunit 1A;SC	ARPC1A;SOP2	Q92747;A4D2	0,90804	0,6650489	1097400	4	4	4
COP9 signalosome complex subunit 8;JAB1-cont	COPS8;CSN8;h	Q99627;B2R8	0,9077	0,6653868	872229,5	4	4	4
Ubiquitin carboxyl-terminal hydrolase L5;Ubiquit	UCHL5;RP11-1	Q5LJA9;Q5LJA	0,90741	0,6656751	1928199	7	7	7
Structural maintenance of chromosomes protein	SMC3;BAM;BI	Q9UQE7;A8K1	0,86087	0,66727	7280503	17	17	17
Protein ADRM1;Adhesion-regulating molecule 1;	ADRM1;GP110	Q16186;Q68E	0,90563	0,6674444	1920800	3	3	3
Lamina-associated polypeptide 2, isoforms beta,	TMPO;LAP2	P42167-1;P42	0,86068	0,6674566	7903199	10	10	4
Bleomycin hydrolase;cDNA FLJ57650, highly sim	BLMH	Q13867;B2R7	0,86046	0,6676728	7231397	7	7	7
Complement component 1 Q subcomponent-bir	C1QBP;GC1QE	Q07021;A8K6	0,86021	0,6679184	1,75E+07	6	6	6
Histone deacetylase 1	HDAC1;RPD3L	Q13547;B4DR	0,85985	0,668272	1,36E+07	10	10	5
U2-associated protein SR140;140 kDa Ser/Arg-ri	SR140;KIAA03	O15042-1;O15	0,85897	0,6691366	4296598	9	9	9
Nucleosome assembly protein 1-like 1;NAP-1-rel	NAP1L1;NRP;I	P55209;B3KN	0,85887	0,6692348	2,67E+07	8	7	7
TRM112-like protein	AD-001;HSPC1	Q9UI30;B2R5	0,90269	0,6703661	491120	2	2	2
Zinc finger CCCH domain-containing protein 15;D	ZC3H15;DFRP	Q8WU90;B4D	0,90262	0,6704356	407550	2	2	2
40S ribosomal protein S26;Ribosomal protein 26	RPS26;RP11-1	P62854;Q76N	0,90136	0,6716876	907950,1	2	2	2
Ras GTPase-activating protein-binding protein 1;	G3BP1;G3BP	Q13283;Q32P	0,85548	0,6725649	1,23E+07	13	13	12
Ribonucleoside-diphosphate reductase subunit M	RRM2;RR2	P31350;B2R9I	0,90013	0,6729095	145610,1	2	2	2
Peroxisomal multifunctional enzyme type 2;D-bi	HSD17B4;EDH	P51659;B2R6	0,85362	0,6743919	3086501	7	7	7
7-dehydrocholesterol reductase;Sterol Delta(7)-	DHCR7;D7SR	Q9UBM7;A8K	0,89768	0,6753428	1759201	5	5	5

LETM1 and EF-hand domain-containing protein :	LETM1	O95202;B4DE	0,89736	0,6756605	1340200	3	3	3
Proteasome activator complex subunit 1;Protea:	PSME1;IFI511	Q06323;Q6FH	0,897	0,676018	306349,9	2	2	2
Small nuclear ribonucleoprotein F	SNRPF;PBSCF	P62306;B2R4!	0,85188	0,6761008	2579402	5	5	5
Basic leucine zipper and W2 domain-containing		BZW2;HSPC02	Q9Y6E2;A4D1	0,8967	0,6763158	572470	3	2	2
Proteasome subunit beta type-6;Proteasome de	PSMB6;LMPY	P28072;Q6IAT	0,84958	0,6783594	4029100	3	3	3
26S protease regulatory subunit 8;Proteasome 2	PSMC5;SUG1;	P62195;A8K7(	0,84834	0,6795768	1,60E+07	13	13	13
cDNA FLJ14622 fis, clone NT2RP2000147, highly	AP1M1;CLTNI	B3KNH5;Q4TT	0,84818	0,6797339	3651801	8	8	6
Heterogeneous nuclear ribonucleoprotein K;Trai	HNRNPK;HNR	P61978-2;P61	0,84777	0,6801364	9,09E+07	18	18	18
Apoptosis inhibitor 5;Fibroblast growth factor 2-	API5;MIG8	Q9BZZ5-2;Q9I	0,84723	0,6806665	3946499	5	5	5
Voltage-dependent anion-selective channel prot	VDAC1;VDAC	P21796;B3KT!	0,84706	0,6808334	1,03E+07	10	10	10
Coatomer subunit beta;Beta-coat protein	COPB1;COPB;	P53618	0,84612	0,6817561	3,65E+07	28	28	28
40S ribosomal protein S5	RPS5	P46782;B2R4T	0,846	0,6818738	2788899	6	6	6
26S proteasome non-ATPase regulatory subunit	PSMD7;MOV3	P51665;B2RD	0,89069	0,6822801	2212000	3	3	3
Lamina-associated polypeptide 2 isoform alpha;T	TMPO;LAP2	P42166	0,89066	0,6823099	542220,1	9	3	3
DnaJ homolog subfamily C member 9;DnaJ prote	DNAJC9	Q8WXX5;B2RI	0,88982	0,683143	58434	2	2	2
26S protease regulatory subunit 6A;Proteasome	PSMC3;TBP1	P17980;A8K7ε	0,84464	0,6832086	1,08E+07	13	13	13
U6 snRNA-associated Sm-like protein LSm3	LSM3;MDS01	P62310;B2R5I	0,88952	0,6834405	811699,8	2	2	2
Gephyrin	GPHN;GPH;KI/	Q9NQX3-2;Q9	0,8441	0,6837385	2833000	7	7	7
Clathrin interactor 1;Epsin-4;Epsin-related prote	CLINT1;ENTH;	Q14677-1;Q1	0,88826	0,6846898	1256800	3	3	3
Serine/threonine-protein phosphatase PP1-alph	PPP1CA;PPP1	P62136;B2R9(	0,84227	0,685534	9852703	12	12	4
Nck-associated protein 1;p125Nap1;Membrane-	NCKAP1;HEM	Q9Y2A7;B3KN	0,84216	0,6856419	2,03E+07	28	28	28
Elongation factor 1-gamma;eEF-1B gamma;cDN/	EEF1G;EF1G;P	P26641;B4DT(	0,84085	0,6869269	2,56E+08	22	22	2
Adipocyte plasma membrane-associated protein	APMAP;C20or	Q9HDC9;A8K5	0,88561	0,6873162	1449300	3	3	3
Transitional endoplasmic reticulum ATPase;15S		VCP	P55072;B2R5T	0,83941	0,6883391	1,94E+08	39	39	39
Leucyl-tRNA synthetase, cytoplasmic;Leucine--tF	LARS;KIAA135	Q9P2J5;A7E2(	0,83935	0,6883979	6975899	18	18	18
Pyrroline-5-carboxylate reductase 2;cDNA FLJ54	PYCR2;hCG_3	Q96C36;A8K7	0,83925	0,6884959	3969801	7	7	6
Zinc finger protein 207 variant;Zinc finger protei	ZNF207	Q59G94;O43(	0,83915	0,688594	2899301	2	2	2
Translin-associated protein X;Translin-associatec	TSNAX;TRAX;F	Q99598;B1AP	0,88416	0,6887525	1137500	4	4	4
NADH dehydrogenase ubiquinone 1 alpha subco	NDUFA10	Q8WXC9;O95	0,88372	0,6891883	1143500	5	5	5
DNA-directed RNA polymerases I, II, and III subu	POLR2H	P52434	0,88371	0,6891982	152700	2	2	2
Eukaryotic translation initiation factor 3 subunit	EIF3B;EIF3S9	P55884-2;P55	0,83795	0,6897706	2,53E+07	22	22	22
Lamin-B receptor;Integral nuclear envelope inne	LBR	Q14739;B2R5	0,88299	0,6899111	1489199	4	4	4
Serine hydroxymethyltransferase, mitochondrial	SHMT2;hCG_4	P34897;B4DJ(	0,83774	0,6899764	1,90E+07	17	17	17

Treacle protein;Treacher Collins syndrome prote	TCOF1;DKFZp4	Q13428-3;Q1	0,88253	0,6903666	585660,1	4	4	4
WW domain-binding protein 11;SH3 domain-bin	WBP11;NPWE	Q9Y2W2;B4D	0,8818	0,6910892	180600	2	2	2
NADH dehydrogenase [ubiquinone] 1 beta subco	NDUFB4	O95168;B2RU	0,88164	0,6912476	611790,1	2	2	2
DNA replication licensing factor MCM4;CDC21 h	MCM4;CDC21	P33991;B3KM	0,83637	0,6913192	2,05E+07	24	24	24
N8 protein long isoform variant;Tumor protein D	TPD52	Q53EK8;Q9U	0,87947	0,6933948	67875,98	2	2	2
DNA replication licensing factor MCM6;p105MC	MCM6	Q14566;B2R6	0,83168	0,6959133	2,27E+07	23	23	23
Calcium homeostasis endoplasmic reticulum pro	CHERP;DAN26	Q8IWX8-1;Q8	0,87679	0,6960447	2363799	6	6	6
cDNA FLJ54776, highly similar to Cell division co	CDC42;RP1-22	B4E1U9;P609	0,87559	0,6972305	524849,8	5	5	5
Nuclear protein Hcc-1;Proliferation-associated c	HCC1;HSPC31	P82979;A8K3	0,87411	0,6986925	667210,4	3	3	3
E3 ubiquitin-protein ligase UBR4;N-recognin-4;Zi	UBR4;KIAA04	Q5T4S7-2;Q5	0,87287	0,6999167	97228	3	3	2
Leucine-rich repeat-containing protein 59	LRRC59;PRO1	Q96AG4;B2RE	0,82709	0,7004046	5665602	6	6	6
Structural maintenance of chromosomes protei	SMC1A;DXS42	Q14683;A8K7	0,8268	0,7006882	7671000	19	19	19
Cullin-5;Vasopressin-activated calcium-mobilizin	CUL5;VACM1	Q93034;A8K9	0,87154	0,7012293	1107400	4	4	4
40S ribosomal protein SA;Laminin receptor 1;34	RPSA;LAMBR;	P08865;A6NE	0,82532	0,7021351	5,34E+07	15	15	4
cDNA, FLJ92904, highly similar to Homo sapiens	CSNK2A1;CK2	B2R6D7;B5BU	0,8237	0,7037182	6545097	13	12	12
Putative uncharacterized protein ABCF2 (ATP-bi	ABCF2;hCG_1	Q75MJ1;Q9U	0,86899	0,7037442	1037300	3	3	3
Bifunctional aminoacyl-tRNA synthetase;Prolifer	EPRS;GLNS;PA	P07814;B4DP	0,82317	0,7042359	1,03E+07	30	30	10
Serine/threonine-protein phosphatase PP1-beta	PPP1CB	P62140;B2R5	0,86772	0,7049958	1001999	10	3	3
Kinesin-like protein KIF2A;HK2	KIF2A;KIF2;KN	O00139-4;O0	0,86699	0,705715	781240,4	3	3	3
SEC23-interacting protein;p125	SEC23IP;MSTF	Q9Y6Y8-1;Q9	0,82163	0,7057399	3456701	7	7	7
Structural maintenance of chromosomes protei	SMC2;CAPE;SI	O95347-1;O9	0,82128	0,7060816	8940303	20	20	20
Dolichyl-diphosphooligosaccharide--protein glyc	RPN1	P04843;B2R5	0,82075	0,706599	2,63E+07	18	18	18
Uncharacterized protein HCFC1;Host cell factor;	HCFC1;HCF1;H	A6NEM2;P51	0,86603	0,7066604	1677999	4	4	4
26S protease regulatory subunit 4;P26s4;Protea	PSMC1	P62191;B4DR	0,81902	0,7082871	8912499	10	10	10
Proteasome subunit beta type-2;Proteasome co	PSMB2	P49721;Q59F	0,81772	0,709555	4932002	6	6	6
Putative RNA-binding protein 3;RNA-binding mo	RBM3;RNPL	P98179;A6ND	0,86304	0,7096026	184330,1	2	2	2
U2 small nuclear ribonucleoprotein A';SNRPA1 p	SNRPA1;hCG_	P09661;B2R5	0,81748	0,709789	2653902	6	6	6
COP9 signalosome complex subunit 5;Jun activa	COPS5;CSN5;J	Q92905;Q59G	0,81539	0,7118261	3237301	7	7	7
ATP-dependent RNA helicase DDX1;DEAD box pr	DDX1	Q92499;A3RJ	0,81427	0,712917	2,74E+07	25	25	25
Vacuolar proton pump subunit G 1;V-ATPase su	ATP6V1G1;AT	O75348;Q6IB	0,85945	0,7131303	260660	2	2	2
Uncharacterized protein EIF4E;Eukaryotic transl	EIF4E;EIF4EL1	A8MX72;Q32	0,85848	0,7140824	314160,2	2	2	2
COP9 signalosome complex subunit 2;JAB1-cont	COPS2;CSN2;T	P61201-2;P61	0,81222	0,7149127	7013101	9	9	9
PRMT1 protein;Protein arginine N-methyltransfe	PRMT1;HMT2	Q8WUW5;Q9	0,81198	0,7151462	3,10E+07	18	18	18

Nucleolin;Protein C23;cDNA FLJ10452 fis, clone I	NCL;hCG_339	P19338;B3KTI	0,8116	0,7155159	8141900	13	13	13
Signal recognition particle 14 kDa protein;18 kDa	SRP14	P37108;B5BU	0,85627	0,7162502	617729,7	3	3	3
Dihydropyrimidinase-related protein 5;ULIP6 pro	DPYSL5;CRMP	Q9BPU6;Q53S	0,85626	0,71626	1224801	4	4	4
Small nuclear ribonucleoprotein E;Putative smal	SNRPE;SNRPE	P62304;B2R5I	0,85613	0,7163874	1969699	2	2	2
Myristoylated alanine-rich C-kinase substrate;Pr	MARCKS;MAC	P29966;Q05C	0,81024	0,7168386	6668902	5	5	5
Coatomer subunit beta';Beta'-coat protein;p102	COPB2	P35606;B4DZI	0,80976	0,7173053	2,83E+07	26	26	26
F-actin-capping protein subunit alpha-1;CapZ alp	CAPZA1	P52907;A8K0T	0,80954	0,7175192	4541203	7	7	7
Stomatin-like protein 2;EPB72-like 2;cDNA FLJ61	STOML2;SLP2	Q9UJZ1;B4E1I	0,85448	0,7180042	733280,2	3	3	3
26S proteasome non-ATPase regulatory subunit	PSMD3	O43242;B3KM	0,80845	0,7185784	1,58E+07	18	18	18
COP9 signalosome complex subunit 6;JAB1-cont	COPS6;CSN6;H	Q7L5N1;A4D2	0,80845	0,7185784	5426002	8	8	8
cDNA FLJ56334, highly similar to SEC13-related p	SEC13;D3S123	B4DXJ1;Q53G	0,80839	0,7186367	3331701	6	6	6
ATP-dependent DNA helicase 2 subunit 1;ATP-de	XRCC6;G22P1	P12956;B2RD	0,80803	0,7189864	5,87E+07	31	31	31
Heterogeneous nuclear ribonucleoprotein M	HNRNPM;HNF	P52272-1;P52	0,80654	0,7204333	7504304	10	10	10
Signal transducing adapter molecule 1	STAM;STAM1	Q92783-1;Q9	0,8062	0,7207634	2526300	3	3	3
Proteasome subunit alpha type-6;Proteasome ic	PSMA6;PROS2	P60900;B2R7J	0,80344	0,7234407	1,44E+07	11	11	10
THO complex subunit 4;Ally of AML-1 and LEF-1;	THOC4;ALY;BE	Q86V81	0,84789	0,7244481	577270,3	2	2	2
Clathrin heavy chain 1;CLH-17	CLTC;CLH17;C	Q00610-1;Q0	0,80198	0,7248554	1,10E+07	31	31	31
SWI/SNF complex subunit SMARCC1;SWI/SNF-re	SMARCC1;BAF	Q92922;B4DY	0,84744	0,7248873	2211301	6	6	6
Cystathionine beta-synthase;Serine sulfhydrase;	CBS	P35520-2;P35	0,80169	0,7251364	3661702	6	6	6
26S protease regulatory subunit 7;Proteasome 2	PSMC2;MSS1	P35998;A4D0	0,80141	0,7254075	1,29E+07	11	11	11
Splicing factor 3B subunit 1;Pre-mRNA-splicing fa	SF3B1;SAP155	O75533;A0JLT	0,79958	0,727179	4892801	11	11	11
Eukaryotic translation initiation factor 3 subunit	EIF3I;EIF3S2;T	Q13347;Q53H	0,79835	0,7283687	1,22E+07	11	11	11
Cytoplasmic dynein 1 heavy chain 1;Cytoplasmic	DYNC1H1;DH(	Q14204;B0I1F	0,79739	0,7292967	1,67E+07	47	47	47
Lysyl-tRNA synthetase;Lysine--tRNA ligase	KARS;hCG_21	A8MSK1;Q9HI	0,84184	0,7303429	847349,9	3	3	3
Coatomer subunit delta;Delta-coat protein;Coat	ARCN1;COPD;	P48444;B0YIV	0,79514	0,7314699	1,70E+07	16	16	16
Poly(U)-binding-splicing factor PUF60;60 kDa po	PUF60;FIR;RO	Q9UHX1-1;Q9	0,79289	0,7336405	9978905	11	11	11
Splicing factor U2AF 65 kDa subunit;U2 auxiliary	U2AF2;U2AF6	P26368-1;P26	0,79173	0,7347584	1,04E+07	6	6	6
26S proteasome non-ATPase regulatory subunit	PSMD14;POH	O00487;B3KN	0,79098	0,7354809	4640502	7	7	7
Cytochrome c oxidase subunit 5A, mitochondria	COX5A	P20674;Q71U	0,83636	0,7356635	1361000	2	2	2
Cytochrome b-c1 complex subunit 1, mitochond	UQCRC1	P31930;B2R7I	0,7907	0,7357504	1,71E+07	14	14	14
Ribophorin II (Ribophorin II, isoform CRA_d);cDN	RPN2;RP3-343	Q5JYR7;B2RE4	0,79027	0,7361645	7957098	8	8	8
Hsc70-interacting protein;Suppression of tumori	ST13;FAM10A	P50502;B4E0U	0,79016	0,7362703	2,95E+07	10	10	10
Cytoplasmic FMR1-interacting protein 2;p53-ind	CYFIP2;KIAA1	Q96F07-2;Q9	0,83561	0,7363902	1472101	18	5	5

Cleavage stimulation factor 77 kDa subunit;CF-1	CSTF3	Q12996;A8K4	0,83531	0,7366807	441929,9	5	5	5
Alpha-actinin-4;Non-muscle alpha-actinin 4;F-ac	ACTN4	O43707;Q96B	0,78932	0,7370786	9,04E+07	46	46	27
Transcription intermediary factor 1-beta;Triparti	TRIM28;KAP1	Q13263-1;Q1	0,789	0,7373865	6,95E+07	25	25	25
Vacuolar protein sorting-associated protein VTA	VTA1;C6orf55	Q9NP79;Q9C	0,83455	0,7374165	586880,1	3	3	3
Signal recognition particle 68 kDa protein;cDNA	SRP68	Q9UHB9-1;Q9	0,78865	0,7377231	3911302	7	7	7
DNA replication licensing factor MCM7;CDC47 h	MCM7;CDC47	P33993-1;P33	0,78783	0,7385115	1,41E+07	17	17	17
Signal recognition particle 9 kDa protein;SRP9 pr	SRP9;hCG_19	P49458;A8K0	0,83271	0,7391965	2097502	4	4	4
cDNA FLJ56327, highly similar to 26S proteasom	PSMD8;hCG_4	B4DX18;Q5U	0,78609	0,740183	3772102	6	6	6
Elongation factor 1-beta	EEF1B2;EEF1B	P24534;A4D1	0,78478	0,7414402	2,34E+07	8	7	7
Exportin-2;Importin-alpha re-exporter;Chromos	CSE1L;CAS;XP	P55060-1;P55	0,78453	0,74168	7,34E+07	34	34	34
Cleavage and polyadenylation specificity factor s	CPSF7	Q8N684-1;Q8	0,82949	0,7423057	2282801	4	4	4
Small nuclear ribonucleoprotein G-like protein;S	SNRPG;PBSCG	A8MWD9;P62	0,78318	0,7429743	5771299	3	3	3
Cytochrome c oxidase subunit 5B, mitochondrial	COX5B	P10606;Q53Y	0,82797	0,7437709	381449,8	2	2	2
40S ribosomal protein S15a	RPS15A;OK/S	P62244;A8K7	0,78108	0,7449854	4924600	6	6	6
Splicing factor U2AF 35 kDa subunit;U2 auxiliary	U2AF1;U2AF3	Q01081;B5BU	0,82576	0,7458982	1697799	4	4	4
Methionyl-tRNA synthetase, cytoplasmic;Methic	MARS	P56192;A8K4	0,77954	0,7464583	5402302	12	12	12
General vesicular transport factor p115;Protein	USO1;VDP	O60763;B2RA	0,77873	0,7472325	3934002	10	10	10
24-dehydrocholesterol reductase;3-beta-hydrox	DHCR24;KIAA	Q15392;Q3LI	0,82418	0,7474169	421489,9	2	2	2
D-3-phosphoglycerate dehydrogenase;Phospho	PHGDH;PGDH	O43175;B2RD	0,77813	0,7478056	1,10E+08	17	17	17
26S proteasome non-ATPase regulatory subunit	PSMD12;hCG_	O00232;A6NP	0,77533	0,7504769	1,02E+07	11	11	11
40S ribosomal protein S23	RPS23	P62266;A8K5	0,82038	0,7510616	1089100	3	3	3
COP9 signalosome complex subunit 3;JAB1-cont	COPS3;CSN3	Q9UNS2;B2R	0,8203	0,7511381	1652800	4	4	4
C-terminal-binding protein 1;C-terminal binding	CTBP1;CTBP;h	Q13363;Q4K	0,82006	0,7513679	613109,9	4	2	2
Ubiquitin fusion degradation protein 1 homolog;	UFD1L	Q92890-1;Q9	0,81927	0,752124	612469,9	2	2	2
NADH dehydrogenase [ubiquinone] 1 beta subc	NDUFB10;hC	O96000;A8K7	0,81834	0,7530134	599670,1	3	3	3
Ubiquitin-associated protein 2-like;Protein NICE-	UBAP2L;KIAA	Q14157-2;Q1	0,77241	0,7532568	1,06E+07	8	8	8
TBC1 domain family member 4;Akt substrate of	TBC1D4;KIAA	O60343;A7E2	0,81777	0,7535582	1318700	3	3	3
40S ribosomal protein S8;Ribosomal protein S8	RPS8;OK/SW-	P62241;Q5JR	0,76855	0,756922	2914401	7	7	7
Dolichyl-diphosphooligosaccharide--protein glyc	DAD1	P61803;Q53G	0,81278	0,7583159	2330100	3	3	3
Proteasome subunit beta type-7;Proteasome su	PSMB7	Q99436;B2RA	0,7666	0,7587692	2614099	5	5	5
ACTN1 protein;Alpha-actinin-1;Alpha-actinin cyt	ACTN1;hCG_2	A1L0V1;B4DH	0,76646	0,7589017	2,94E+07	44	26	23
Splicing factor 3 subunit 1;Spliceosome-associat	SF3A1;SAP11	Q15459;B4E0	0,76633	0,7590248	2,60E+07	23	23	23
Enhancer of rudimentary homolog	ERH	P84090;B2R5	0,81163	0,7594094	1514100	2	2	2

Small nuclear ribonucleoprotein-associated prot	SNRPB;COD;SI	P14678-3;P14	0,76381	0,7614071	5422904	6	6	6
High mobility group protein B3;High mobility grc	HMGB3;HMG.	O15347;Q164	0,80904	0,7618678	986340,3	3	3	3
28S ribosomal protein S34, mitochondrial;MRP-S	MRPS34	P82930;A4UC	0,8085	0,7623796	1031300	4	4	4
WD repeat and HMG-box DNA-binding protein 1	WDHD1;AND1	O75717;A8KA	0,80775	0,7630901	695870,2	2	2	2
Probable RNA-binding protein 25;RNA-binding m	RBM25;RNPC1	P49756;A0PJL	0,76202	0,7630961	6,39E+07	28	28	28
Dihydropyrimidinase-related protein 2;Collapsin	DPYSL2;CRMP	Q16555;A8K5	0,80666	0,7641216	1086300	3	2	2
Heterogeneous nuclear ribonucleoprotein U-like	HNRNPUL1;E1	Q9BUJ2-1;Q9I	0,76066	0,7643777	4288199	10	9	9
LanC-like protein 1;40 kDa erythrocyte membrai	LANCL1;GPR6	O43813;B2R6	0,75906	0,7658833	4637202	10	10	10
Vesicle-fusing ATPase;N-ethylmaleimide-sensitiv	NSF	P46459;A8K2I	0,80451	0,7661532	2070599	9	9	9
Casein kinase II subunit alpha'	CSNK2A2;CK2.	P19784	0,80423	0,7664174	1274401	6	5	5
Trafficking protein particle complex subunit 3;BE	TRAPPC3;BET3	O43617;B2RD	0,80394	0,766691	347930	2	2	2
Protein ERGIC-53;ER-Golgi intermediate compar	LMAN1;ERGIC	P49257;A0PJ8	0,75787	0,7670018	4028099	5	5	5
Valyl-tRNA synthetase;Valine--tRNA ligase;Prote	VARS;DAAP-2	B0V043;P2664	0,75691	0,7679032	1,31E+07	18	18	18
NADH dehydrogenase [ubiquinone] 1 alpha subc	NDUFA8	P51970;B1AM	0,80093	0,7695262	1193000	5	5	5
Methylcrotonoyl-CoA carboxylase beta chain, m	MCCC2;MCCB	Q9HCC0-1;Q9	0,75494	0,7697505	1,02E+07	12	12	12
Transcription factor A, mitochondrial;mtTFA;Mit	TFAM;TCF6L2	Q00059;A9QX	0,79987	0,7705226	349239,8	2	2	2
Condensin complex subunit 1;Non-SMC condens	NCAPD2;CAPD	Q15021;B3KM	0,79976	0,7706259	2320499	9	9	9
Squamous cell carcinoma antigen recognized by	SART3;KIAA01	Q15020-1;Q1!	0,79835	0,7719494	1861799	6	6	6
Dolichyl-diphosphooligosaccharide--protein glyc	DDOST;KIAA0	P39656;B2RD	0,75212	0,7723886	8748002	10	10	10
AP-2 complex subunit mu-1;Adaptin-mu2;AP-2 r	AP2M1;CLAPN	Q96CW1-1;Q9	0,79712	0,7731023	1382900	4	4	4
Creatine kinase, ubiquitous mitochondrial;U-Mt(	CKMT1A;CKM	P12532;B4DIT	0,75048	0,7739196	3,69E+07	13	13	10
Condensin complex subunit 3;Non-SMC condens	NCAPG;CAPG;	Q9BPX3;Q6NU	0,79526	0,7748428	1855001	5	5	5
Small nuclear ribonucleoprotein Sm D1;snRNP cc	SNRPD1	P62314;B5BTZ	0,74864	0,7756342	3423301	4	4	4
Proteasome subunit beta type-4;Macropain beta	PSMB4	P28070;B2R9I	0,74789	0,7763322	2922700	7	7	7
Nuclease-sensitive element-binding protein 1;Y-	YBX1;NSEP1;Y	P67809;A0JLU	0,74754	0,7766578	3674602	5	5	5
CD2 antigen cytoplasmic tail-binding protein 2;C	CD2BP2;KIAA1	O95400;B2RD	0,7932	0,7767664	506089,8	2	2	2
Eukaryotic translation initiation factor 3 subunit	EIF3C;EIF3S8;I	B5ME19;Q996	0,74637	0,7777451	1,68E+07	18	18	18
Isoleucyl-tRNA synthetase, cytoplasmic;Isoleucir	IARS;hCG_311	P41252;B4DV	0,74519	0,7788404	6756101	18	18	18
WD repeat-containing protein 61;Meiotic recom	WDR61	Q9GZS3	0,79021	0,7795504	1047300	4	4	4
Cell differentiation protein RCD1 homolog	RQCD1;RCD1	Q92600;B2RE	0,78935	0,7803495	328449,8	3	3	3
Fatty acid-binding protein, epidermal;E-FABP;Fat	FABP5	Q01469;B2R4	0,78821	0,7814074	81281,02	3	3	3
Apoptosis regulator BAX;Bax-sigma (BCL2-associ	BAX;hCG_181	Q07812-2;Q07	0,78759	0,7819821	381650,1	2	2	2
Catenin alpha-1;Cadherin-associated protein;Alp	CTNNA1	P35221-2;P35	0,78589	0,783556	2292500	7	7	7

Small nuclear ribonucleoprotein Sm D2;snRNP c	SNRPD2;SNRP	P62316;A8K7	0,73708	0,7863308	6218102	8	8	8
Peptidyl-prolyl cis-trans isomerase H;U-snRNP-a	PPIH;CYP20;C	O43447;Q6FH	0,78265	0,7865465	393110,1	3	3	3
Ribonuclease P (30kD) (RPP30);Ribonuclease P p	RP11-320F15.	Q5VU11;P783	0,78265	0,7865465	282859,8	3	3	3
Serine/arginine repetitive matrix protein 2;Serin	SRRM2;KIAA0	Q9UQ35-1;Q9	0,78232	0,7868504	722199,6	7	7	7
Cytochrome b-c1 complex subunit 8;Ubiquinol-c	UQCRQ	O14949	0,7817	0,7874211	921320,1	2	2	2
Synaptosomal-associated protein 23;Vesicle-me	SNAP23	O00161-1;O0	0,73516	0,7880941	3442499	8	8	8
Splicing factor, proline- and glutamine-rich;Poly	SFPQ;PSF	P23246-1;P23	0,73512	0,7881308	1,09E+08	26	26	25
Vacuolar ATP synthase subunit B, brain isoform;	ATP6V1B2;AT	P21281;B2R5	0,78082	0,7882304	547579,8	2	2	2
Inositol-3-phosphate synthase;Myo-inositol-1-p	INO1;ISYNA1	Q9NPH2-1;Q9	0,7806	0,7884325	806150,3	2	2	2
ATP synthase protein 8;A6L	MT-ATP8;ATP	P03928;A0S2	0,77978	0,7891856	143340	2	2	2
DNA primase large subunit;DNA primase 58 kDa	PRIM2;PRIM2	P49643-1;P49	0,77638	0,7922996	431540,2	3	3	3
Splicing factor 3B subunit 2;Pre-mRNA-splicing f	SF3B2;SAP14	Q13435;A8K4	0,72846	0,7942158	5789701	9	9	9
Sorting nexin-9;SH3 and PX domain-containing p	SNX9;SH3PX1	Q9Y5X1;B2RA	0,77273	0,7956271	476439,9	2	2	2
26S proteasome non-ATPase regulatory subunit	PSMD11	O00231;A8K3	0,72597	0,796478	2,18E+07	15	15	15
Alpha-taxilin	TXLNA;TXLN	P40222	0,77165	0,7966085	913389,6	3	3	3
Histone-binding protein RBBP4;Retinoblastoma-	RBBP4;RBAP4	Q09028;B2R6	0,72553	0,796877	9992094	10	5	5
Centromere/kinetochore protein zw10 homolog	ZW10	O43264;A1A5	0,77054	0,7976157	1656401	4	4	4
Aspartyl-tRNA synthetase, cytoplasmic;Aspartat	DARS;PIG40	P14868;A8K3	0,76892	0,7990829	2261001	6	6	6
Uncharacterized protein TLE4;Transducin-like en	TLE4;KIAA126	A6NIY7;Q5JTT	0,76876	0,7992276	1796299	3	3	3
U6 snRNA-associated Sm-like protein LSm4;Glyc	LSM4	Q9Y4Z0	0,76719	0,8006461	716869,6	3	3	3
Pre-mRNA-processing factor 19;PRP19/PSO4 ho	PRPF19;NMP	Q9UMS4	0,76481	0,8027902	827840,2	2	2	2
Coatomer subunit gamma;Gamma-coat protein	COPG;COPG1	Q9Y678;A8K6	0,7161	0,8053728	3,33E+07	26	26	22
UPF0027 protein C22orf28;cDNA FLJ58027	C22orf28;HSP	Q9Y3I0;B2R6	0,71412	0,8071428	3,14E+07	17	17	17
Eukaryotic translation initiation factor 3 subunit	EIF3G;EIF3S4	O75821;A8K5	0,71375	0,8074729	6474798	7	7	7
ATP synthase subunit gamma, mitochondrial	ATP5C1;ATP5	P36542-1;P36	0,71327	0,807901	7505604	7	7	7
Pre-mRNA-processing factor 40 homolog A;Form	PRPF40A;FBP	O75400-1;O7	0,71318	0,8079813	2,37E+07	17	17	17
Small nuclear ribonucleoprotein Sm D3;snRNP c	SNRPD3	P62318;B5BU	0,71287	0,8082575	6762097	3	3	3
Signal recognition particle 54 kDa protein	SRP54	P61011;B2R7	0,71084	0,8100637	6465201	13	13	13
Protein enabled homolog	ENAH;MENA	Q8N8S7-1;Q8	0,75461	0,8118943	1590200	5	5	5
Myosin light polypeptide 6;Smooth muscle and	MYL6	P60660-1;P60	0,70857	0,812077	7837605	9	9	9
Cytochrome b-c1 complex subunit 9;Ubiquinol-c	UQCR10;UCR	Q9UDW1;Q9	0,75366	0,8127349	1548300	2	2	2
Cytochrome b-c1 complex subunit 7;Ubiquinol-c	UQCRB;UQBP	P14927;B2R4	0,75364	0,8127526	2299099	6	6	6
KH domain-containing, RNA-binding, signal trans	KHDRBS1;SAM	Q07666-1;Q0	0,7075	0,8130237	1,60E+07	5	5	4

YTH domain family protein 3	YTHDF3	Q7Z739;B3KX	0,75298	0,8133359	1689599	4	4	4
26S proteasome non-ATPase regulatory subunit	PSMD9	O00233-1;O0(	0,75293	0,8133801	185900	3	3	3
60S acidic ribosomal protein P2;Renal carcinoma	RPLP2;D11S22	P05387;Q6FG	0,75137	0,8147561	724839,6	3	3	3
Glucosidase 2 subunit beta;Glucosidase II subuni	PRKCSH;G19P	P14314;B4DJ(	0,70126	0,8185136	1,47E+07	12	12	12
Splicing factor 1;Zinc finger protein 162;Transcri	SF1;ZFM1;ZNF	Q15637-5;Q1!	0,69995	0,8196592	4251698	5	5	5
COMM domain-containing protein 4;cDNA FLJ58	COMMD4	Q9H0A8-1;Q9	0,74385	0,8213401	252089,9	2	2	2
Nucleoporin 50 kDa;Nuclear pore-associated pro	NUP50;NPAP6	Q9UKX7;B2RE	0,7363	0,8278651	1109100	2	2	2
Septin-11;Septin-6;Septin 6 isoform E;Septin 6 (S	SEPT11;SEPT6	Q9NVA2;Q4W	0,68838	0,8296707	1,13E+07	11	11	8
Signal recognition particle 19 kDa protein	SRP19	P09132;B2R4I	0,7327	0,8309452	441469,8	3	3	3
Reticulon-4;Neurite outgrowth inhibitor;Foocen	RTN4;KIAA088	Q9NQC3-1;Q9	0,73134	0,8321034	1866100	2	2	2
Splicing factor 3A subunit 3;Spliceosome-associa	SF3A3;SAP61	Q12874;A8K5	0,68212	0,8350038	9958306	9	9	9
cDNA, FLJ96836, highly similar to Homo sapiens	CAP1;CAP;RP1	B2RDY9;B4DI3	0,67988	0,8368972	1,77E+07	12	12	4
Kinesin light chain 1	KLC1;KLC;KNS	Q07866-9;Q0	0,72381	0,8384616	930690,4	3	3	3
WD repeat-containing protein 82;Swd2	WDR82;WDR8	Q6UXN9;A8K5	0,72344	0,8387716	320600	2	2	2
Cytochrome b-c1 complex subunit 2, mitochond	UQCRC2	P22695;B3KSI	0,67718	0,8391689	2,57E+07	13	13	13
Ataxin-2-like protein;Ataxin-2 domain protein;At	ATXN2L;A2D;A	Q8WWM7-1;(	0,67508	0,8409276	1,53E+08	32	32	32
Biogenesis of lysosome-related organelles comp	BLOC1S1;BLO:	P78537;A1L4(	0,71939	0,8421498	261860	2	2	2
NADH dehydrogenase [ubiquinone] 1 subunit C2	NDUFC2;HLC1	O95298;Q549	0,71709	0,8440557	444829,7	3	3	3
Splicing factor 3A subunit 2;Spliceosome-associa	SF3A2;SAP62	Q15428;B2RB	0,71256	0,8477827	1583401	3	3	3
U1 small nuclear ribonucleoprotein A	SNRPA	P09012;B2R8(	0,66551	0,8488494	5218000	5	5	5
Coiled-coil-helix-coiled-coil-helix domain-contair	CHCHD3	Q9NX63;A4D1	0,70842	0,8511569	391809,8	2	2	2
Coatomer subunit gamma-2;Gamma-2-coat prot	COPG2	Q9UBF2;B3KN	0,70626	0,8529051	1980900	8	4	4
Mitochondrial ribosomal protein S28 variant;28S	MRPS28;MRP	Q53G62;Q9Y2	0,7055	0,8535182	153800	3	3	3
Alpha-soluble NSF attachment protein;N-ethylm	NAPA;SNAPA	P54920;A8K8	0,69913	0,8586148	391620,3	3	3	3
Vesicle-associated membrane protein-associate	VAPB;UNQ484	O95292-1;O9!	0,69709	0,8602309	468790,3	4	3	3
Eukaryotic translation initiation factor 3 subunit	EIF3F;EIF3S5	O00303;A8K0	0,64971	0,8615788	7584195	6	6	6
Coatomer subunit epsilon;Epsilon-coat protein;F	COPE;hCG_20	O14579;Q53H	0,64919	0,86199	5010202	8	8	8
Uncharacterized protein EIF3D;Eukaryotic transl	EIF3D;EIF3S7;	A8MWD3;O15	0,6486	0,862456	1,08E+07	8	8	8
Multisynthetase complex auxiliary component p	JTV1;PRO0992	Q13155;B2R7	0,69361	0,8629694	231880	3	3	3
Cleavage and polyadenylation specificity factor s	NUDT21;CFIM	O43809;B2R6	0,64632	0,8642505	1,05E+07	10	10	10
UPF0160 protein MYG1;MSTP024 (C12orf10 pro	C12orf10;MST	Q9HB07;Q86U	0,69087	0,8651092	177490	2	2	2
Eukaryotic translation initiation factor 3 subunit	EIF3M;HFLB5;	Q7L2H7;A8K7	0,64495	0,8653241	4435700	8	8	8
Cleavage and polyadenylation specificity factor s	CPSF6;CFIM68	Q16630-2;Q1(	0,64454	0,8656446	1,83E+07	10	10	10

Eukaryotic translation initiation factor 3 subunit	EIF3K;EIF3S12	Q9UBQ5;A8K(	0,68796	0,8673657	1600400	5	5	5
BetaCstF-64 variant 2;Cleavage stimulation facto	CSTF2;CSTF2T	B3V096;P332	0,64208	0,8675614	2590700	4	4	4
Sjoegren syndrome/scleroderma autoantigen 1;.	SSSCA1	O60232	0,68622	0,868707	878029,5	2	2	2
ATP synthase subunit epsilon, mitochondrial;ATI	ATP5E;ATP5EI	P56381;B2RD	0,68113	0,8725957	700169,7	2	2	2
ATP synthase subunit e, mitochondrial	ATP5I;ATP5K	P56385	0,6804	0,8731491	2217800	4	4	4
Eukaryotic translation initiation factor 3 subunit	EIF3H;EIF3S3	O15372;B3KS	0,67994	0,8734974	1978401	5	5	5
Eukaryotic translation initiation factor 3 subunit	EIF3E;EIF3S6;I	P60228;B2R8	0,63318	0,8743976	1,50E+07	18	18	18
Proteasome subunit beta type-3;Proteasome th	PSMB3	P49720	0,67821	0,8748029	1630901	7	7	7
Septin-7;CDC10 protein homolog;cDNA FLJ5257	SEPT7;CDC10;	Q16181-1;Q1	0,63252	0,8748983	9787606	8	8	8
Wiskott-Aldrich syndrome protein family memb	WASF1;KIAA0	Q92558;Q5SZ	0,67361	0,8782445	591170,2	2	2	2
ATP synthase subunit O, mitochondrial;Oligomy	ATP5O;ATPO	P48047;B2R4I	0,62454	0,8808823	8092096	7	7	7
AP-3 complex subunit sigma-1;Adapter-related p	AP3S1;CLAPS3	Q92572;B2R4	0,66784	0,882499	931520,1	5	5	5
UPF0568 protein C14orf166;CLE	C14orf166;CG	Q9Y224;Q549	0,62014	0,8841255	5047500	11	11	11
cDNA FLJ55467, highly similar to Septin-2;Septin	NEDD5;SEPT2	B4DGE8;Q53C	0,61971	0,8844403	7768405	8	8	8
ATP synthase subunit g, mitochondrial	ATP5L	O75964;A8K0	0,61816	0,8855718	4075700	6	6	6
Ubiquitin-like 4A;Ubiquitin-like protein 4A;Ubiqu	UBL4A;XX-FW	Q5HY81;P114	0,65999	0,8881731	295780,2	2	2	2
Replication protein A 32 kDa subunit;RP-A p34;R	RPA2;REPA2;F	P15927-3;P15	0,61398	0,8885975	3002698	5	5	5
39S ribosomal protein L12, mitochondrial;MRP-I	MRPL12;RPM	P52815;Q96Q	0,65854	0,8892066	479570	3	3	3
COP9 signalosome complex subunit 7a;JAB1-con	COPS7A;CSN7	Q9UBW8;A8K	0,65623	0,8908436	208969,9	2	2	2
Uncharacterized protein ABI1;Abl interactor 1;A	ABI1;SSH3BP1	A6NFN2;Q8IZ	0,60973	0,8916353	8246102	7	7	7
Leucine-rich repeat flightless-interacting protein	LRRFIP1;GCF2	Q32MZ4-1;Q3	0,65483	0,8918299	223660,1	2	2	2
Transmembrane protein 85;Cell proliferation-in	TMEM85;HSP	Q5J8M3-1;Q5	0,65407	0,8923635	349400	2	2	2
Desmoplakin;250/210 kDa paraneoplastic pemp	DSP	P15924-1;P15	0,65391	0,8924757	190930	6	6	6
Propionyl-CoA carboxylase alpha chain, mitocho	PCCA;RP11-15	P05165;B2RD	0,60841	0,8925708	8,21E+07	32	32	32
Uncharacterized protein MRPS36;28S ribosomal	MRPS36;DC47	A8MWD6;P82	0,6516	0,8940889	255810,2	2	2	2
Eukaryotic translation initiation factor 3, subunit	EIF3EIP;AL022	B0QY89;B3KP	0,60559	0,8945565	1,39E+07	17	17	17
Dynactin subunit 2;Dynactin complex 50 kDa sul	DCTN2;DCTN5	Q13561;B2RB	0,60162	0,8973221	5185197	9	9	9
Non-POU domain-containing octamer-binding p	NONO;NRB54	Q15233;A8K5	0,59952	0,8987708	1,11E+08	22	21	21
Probable ATP-dependent RNA helicase DDX5;DE	DDX5;G17P1;I	P17844;B4DN	0,59886	0,8992241	1,30E+07	16	10	10
Nuclear fragile X mental retardation-interacting	NUFIP2;KIAA1	Q7Z417;A1L3	0,59847	0,8994915	6027900	8	8	8
6-phosphofructokinase, liver type;Phosphofruct	PFKL;DKFZp68	P17858-2;P17	0,6431	0,8999218	814089,9	4	2	2
Pyrroline-5-carboxylate reductase 1, mitochond	PYCR1;hCG_3	P32322;B4DN	0,64285	0,9000909	1450300	5	4	4
Transmembrane protein 93	TMEM93	Q9BV81	0,64279	0,9001315	336059,8	2	2	2

Dynein, light chain, roadblock-type 1;cDNA FLJ59	DYNLRB1;RP5	B1AKR6;B4DF	0,64276	0,9001518	481109,9	2	2	2
Myosin regulatory light chain MRCL3 variant;My	MYLC2B;MRL(	Q53HL1;O149	0,59625	0,9010068	3619501	4	4	4
Glutaminyl-tRNA synthetase;Glutamine--tRNA li	QARS;hCG_96	P47897;A8K3	0,6411	0,9012704	913319,6	4	4	4
ATP synthase subunit f, mitochondrial;Uncharac	ATP5J2;ATP5J	P56134-1;P56	0,63988	0,9020885	513430,2	3	3	3
Small nuclear ribonucleoprotein polypeptide C v	SNRPC;RP3-37	Q53G33;Q5TA	0,63811	0,9032693	545410,2	2	2	2
UPF0569 transmembrane protein;Uncharacterized protein ENS		Q3ZAQ7;B3KU	0,63805	0,9033092	593860,2	2	2	2
cDNA FLJ77033, highly similar to Homo sapiens	HAGH;GLO2;H	A8K290;B4DR	0,59278	0,9033529	3873100	5	5	5
28S ribosomal protein S22, mitochondrial;MRP-S	MRPS22;C3or	P82650;A8K9Y	0,58827	0,9063608	7908498	6	6	6
DNA-directed RNA polymerases I, II, and III subu	POLR2E	P19388;B2R6I	0,63337	0,9063957	397920,2	2	2	2
Replication protein A 14 kDa subunit;Replication	RPA3;REPA3;F	P35244;A4D1	0,62906	0,9091929	983070,1	6	6	6
Angiomotin;Uncharacterized protein AMOT	AMOT;KIAA1C	Q4VCS5-1;Q4	0,58319	0,909692	6723800	11	11	11
40S ribosomal protein S6;Phosphoprotein NP33;	RPS6;OK/SW-	P62753;A2A3	0,62734	0,9102968	1228500	2	2	2
Coatomer subunit zeta-1;Zeta-1-coat protein	COPZ1;COPZ;(	P61923;Q53F	0,62695	0,9105462	723859,9	2	2	2
Pyruvate carboxylase, mitochondrial;Pyruvic car	PC	P11498	0,58025	0,9115922	3,02E+08	51	51	51
Tyrosine-protein kinase receptor;Protein TFG;TR	TFG/ALK fusio	Q8TDJ5;Q927	0,57924	0,9122403	6970101	4	4	4
Protein-L-isoaspartate(D-aspartate) O-methyltra	PCMT1	P22061-2;P22	0,57897	0,9124131	2,75E+08	15	15	15
AP-2 complex subunit alpha-1;Adapter-related p	AP2A1;ADTAA	O95782-1;O9	0,62139	0,9140617	578540,1	6	2	2
Cell division cycle and apoptosis regulator prote	CCAR1;CARP1	Q8IX12;A0JLT	0,61765	0,9163847	1361800	3	3	3
28S ribosomal protein S24, mitochondrial;MRP-S	MRPS24;HSPC	Q96EL2;A4D1	0,6132	0,9191046	227640	2	2	2
ATP synthase subunit d, mitochondrial;ATP5H pr	ATP5H;My032	O75947-1;O7	0,61306	0,9191894	1192199	7	7	7
ATP synthase subunit b, mitochondrial;ATP synt	ATP5F1;RP11-	P24539;A8K4	0,56742	0,9196425	2694000	5	5	5
Replication protein A 70 kDa DNA-binding subun	RPA1;REPA1;F	P27694;A8K0	0,56457	0,9213765	8417404	13	13	13
Alpha-centractin;Centrosome-associated actin h	ACTR1A;CTRN	P61163;B2R6	0,56417	0,9216183	3150998	6	6	6
Probable protein BRICK1	C3orf10;HSPC	Q8WUW1-2;C	0,60865	0,9218357	244579,9	2	2	2
Propionyl-CoA carboxylase beta chain, mitochon	PCCB	P05166;Q59G	0,56273	0,9224855	6,42E+07	26	26	26
B-cell receptor-associated protein 31;BCR-associ	BCAP31;BAP3	P51572;B3KQ	0,60334	0,9249588	2114899	3	3	3
Mitochondrial inner membrane protein;Mitofilir	IMMT;HMP;P	Q16891-1;Q1	0,59928	0,9272996	1009200	4	4	4
Similar to cytoskeleton-associated protein 4;Cyt	CKAP4	Q8TB01;Q070	0,59673	0,9287489	2303800	4	4	4
Methylcrotonoyl-CoA carboxylase subunit alpha	MCCC1;MCCA	Q96RQ3;B4D	0,59651	0,9288732	1897300	6	6	6
Splicing factor 3B subunit 5;Pre-mRNA-splicing f	SF3B5;SF3B10	Q9BWJ5;B2R5	0,58514	0,935131	404219,8	2	2	2
Prohibitin;Prohibitin variant;Uncharacterized pr	PHB	P35232;A8K4	0,54014	0,935412	8059297	12	12	12
Prohibitin-2;B-cell receptor-associated protein B	PHB2;BAP;RE	Q99623;Q9BX	0,53933	0,9358516	1,38E+07	10	10	10
28S ribosomal protein S29, mitochondrial;MRP-S	DAP3;MRPS2	P51398;B4DP	0,58271	0,9364262	589030,2	2	2	2

Protein FAM98A;Family with sequence similarity	FAM98A;hCG_	Q8NCA5;B5M	0,58174	0,936939	1965901	3	3	3
NADH-ubiquinone oxidoreductase 75 kDa subun	NDUFS1	P28331;B4DIN	0,58146	0,9370867	1512900	2	2	2
ATP synthase subunit alpha, mitochondrial;cDNA	ATP5A1;ATP5.	P25705;A8K09	0,53579	0,937753	1,14E+08	26	26	26
Paraspeckle component 1;Paraspeckle protein 1	PSPC1;PSP1	Q8WXF1-1;Q8	0,53285	0,9393078	7909900	8	8	8
Protein LSM12 homolog;cDNA FLJ51308	LSM12	Q3MHD2-2;Q	0,52321	0,9442499	1,03E+07	6	6	6
RNA-binding protein FUS;Oncogene FUS;Oncoge	FUS;TLS	P35637-1;P35	0,51668	0,9474611	2,36E+07	5	5	3
U1 small nuclear ribonucleoprotein 70 kDa	SNRP70;RNPU	P08621-1;P08	0,55834	0,9485869	1676500	2	2	2
Eukaryotic translation elongation factor 1 epsilo	EEF1E1;P18	O43324	0,55122	0,9518543	854300,4	4	4	4
ATP synthase subunit beta, mitochondrial	ATP5B;ATPMB	P06576;A8K4X	0,50618	0,9523928	1,49E+08	23	23	23
cDNA FLJ55422, highly similar to Septin-9;cDNA	Ov/Br septin;S	B4DTL7;Q96Q	0,54975	0,9525128	1262501	3	3	3
Dynein light chain Tctex-type 1;T-complex testis-	DYNLT1;TCTEI	P63172;Q5VT	0,54704	0,9537123	188780	2	2	2
28S ribosomal protein S7, mitochondrial;MRP-S7	MRPS7	Q9Y2R9;B2R9	0,53991	0,9567792	681860	3	3	3
NADH dehydrogenase [ubiquinone] 1 beta subco	NDUFB11;UN(	Q9NX14-2;Q9	0,53902	0,9571529	604369,9	2	2	2
ATP synthase subunit delta, mitochondrial	ATP5D	P30049;Q6FG	0,5376	0,9577451	1717701	2	2	2
Uncharacterized protein DCTN1;Dynactin subuni	DCTN1	A8MY36;A8M	0,52806	0,9615918	1393000	4	4	4
Ras GTPase-activating protein-binding protein 2;	G3BP2;KIAA0(	Q9UN86-1;Q9	0,52767	0,9617441	1111500	3	2	2
cDNA FLJ45695 fis, clone FEBRA2013570, highly	BCKDHA;DKFZ	Q6ZSA3;B4DP	0,48095	0,9630735	6575096	10	10	10
Ribonucleoprotein PTB-binding 1;Protein raver-1	RAVER1;KIAA1	Q8IY67-2;Q8IY	0,52181	0,963988	523240,3	2	2	2
			0,5078	0,9690075	1227000	3	3	3
Vimentin	VIM	P08670;B0YJC	0,46195	0,9700375	2,17E+07	18	18	17
Cytoplasmic dynein 1 light intermediate chain 1;	DYNC1LI1;DN(	Q9Y6G9;A2RR	0,49817	0,97218	1404899	3	3	3
Protease-associated domain-containing protein	PAP21;C2orf7	Q9BSG0;Q2Z1	0,49013	0,9746589	939169,7	4	4	4
Eukaryotic translation initiation factor 1A, X-chro	EIF1AX;EIF1A;	P47813;B2R5U	0,48783	0,97534	1200000	3	3	3
Probable ATP-dependent RNA helicase DDX6;DE	DDX6;HLR2;R(	P26196;B2R85	0,44113	0,9766323	9,61E+07	19	19	19
NADH dehydrogenase [ubiquinone] iron-sulfur p	NDUFS5	O43920;Q6IBA	0,47883	0,9778877	171230	3	3	3
2-oxoisovalerate dehydrogenase subunit beta, n	BCKDHB;RP1-:	P21953;B4E2I	0,46988	0,9802389	2273999	7	7	7
Coiled-coil domain-containing protein 124	CCDC124	Q96CT7	0,43461	0,9878404	361709,9	3	3	3
28S ribosomal protein S16, mitochondrial;MRP-S	MRPS16;RPM	Q9Y3D3;B4E0	0,39458	0,9936393	259470	2	2	2
Endothelial differentiation-related factor 1;Mult	EDF1	O60869-1;O6(	0,37183	0,9958378	451380,1	3	3	3
Lipoamide acyltransferase component of branch	DBT;BCATE2;F	P11182;B2R81	0,24078	0,9996606	3,84E+08	30	30	30
Cytochrome c;Uncharacterized protein ENSP000	CYCS;CYC	P99999;B2R4I	0,20891	0,9999037	5433101	5	5	4
cDNA FLJ37462 fis, clone BRAWH2011343, highl	CIRBP;A18HNI	B3KT17;B4E2X	0,22068	0,9999578	1173700	2	2	2
Metastasis-associated protein MTA2;Metastasis-	MTA2;MTA1L	O94776;Q68D	0,19535	0,9999885	785750,4	3	3	3

Dermcidin;Preproteolysin;Survival-promoting pe	DCD;AIDD;DSI	P81605;Q53Y	0,1711	0,9999975	1147299	2	2	2
60S ribosomal protein L9;Putative uncharacteriz	RPL9;OK/SW-	P32969;Q53Z	0,11465	1	354900,2	2	2	2
DNA-directed RNA polymerase III subunit RPC1;I	POLR3A	O14802;Q7Z7	0,1069	1	752060,3	2	2	2
Protein S100-A8;S100 calcium-binding protein A	S100A8;CAGA	P05109;A8K5	0,0894	1	2342799	3	3	3
Lysozyme C;1,4-beta-N-acetylmuramidase C	LYZ;LZM	P61626;B2R4	0,084101	1	573739,9	2	2	2
Annexin A1;Annexin-1;Annexin I;Lipocortin I;Cal	ANXA1;ANX1;	P04083;B5BU	0,047147	1	625130,4	2	2	2

ides (seq)

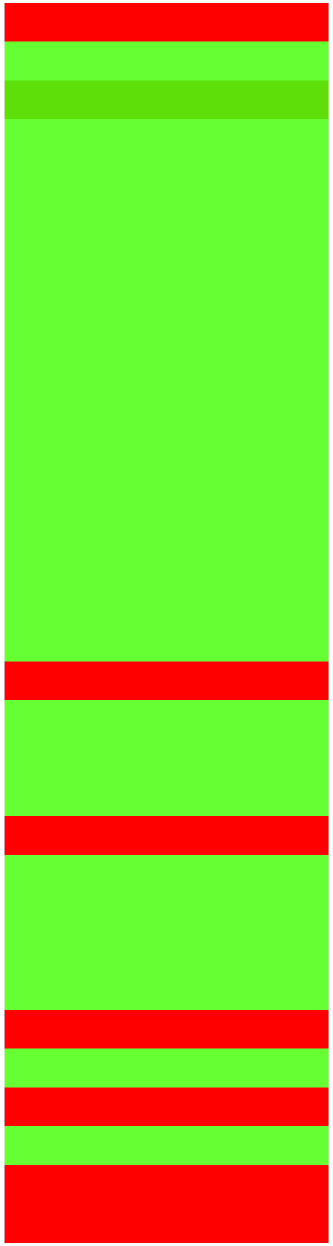

Supplement: S1 Table — For each protein group, the protein descriptions, the gene names, the uniprot identifiers as well as the sequence coverage and unique peptide counts are shown. Additionally, the mean ratio from two independent experiments for the affinity purified PIFO compared to the SF-TAP control is shown as well as the significance B for enrichment. Proteins with a significance level below 0.05 were considered to be potential PIFO interactors. Identification, quantification and significance determination was performed by using MaxQuant, including the Perseus software for statistical analysis. Protein groups significantly enriched are colored in green, those of special interest are colored in red. (PDF) [file pone.0149477.s008.pdf]
