# Supplementary material for: Pitchfork and Gprasp2 Target Smoothened to the Primary Cilium for Hedgehog Pathway Activation
Source: PLoS One. 2016 Feb 22;11(2):e0149477. doi: 10.1371/journal.pone.0149477 (PMC4763541; doi:10.1371/journal.pone.0149477)
Supplement: S2 Table — The restriction enzyme sites are indicated in red. (DOC) [file pone.0149477.s009.doc]

**Jung et al., S2 Table.**

| Primers | Sequence |
| --- | --- |
| 5’-Pifo Ex2 HR NotI | NNN GCGGCCGC CAGCGCCCTCTTGAGCTGTCAGTC |
| 5’-Pifo Ex2 HR EcoRI/BamHI | NNN GAATTC GGATCC GCCTTCCCCCACCTTGTCTTGGTTC |
| 5’-Pifo Ex3 HR EcoRI/ApaI | NNN GAATTC GGGCCC ACTCCACTTTGAAGGCCGTTC |
| 5’-Pifo Ex3 HR KpnI | NNN GGTACC GTGGAAGGAAGAGAACGAAGGC |
| 3’-Pifo after Ex6 HR1 NotI | NNN GCGGCCGC CCTGTTGGTTATCTAACCAGATTG |
| 3’-Pifo after Ex6 HR1 EcoRI/BamHI | NNN GAATTC GGATCC TATCTAAGTCCTTGATATATAGGGGC |
| 3’-Pifo after Ex6 HR2 EcoRI | NNN GAATTC GAGCTCACAAAGTATAGAAAATACTTAAATTATG |
| 3’-Pifo after Ex6 HR2 KpnI | NNN GGTACC GGCTAAGCAAGTGATGTCATGG |
| EP551 | NNN GCGGCCGC CAGCGCCCTCTTGAGCTGTCAGTC |
| EP554 | NNN GGTACC GTGGAAGGAAGAGAACGAAGGC |
| EP485 | ATGCCCAAGAAGAAGAGGAAGGT |
| EP486 | GAAATCAGTGCGTTCGAACGCTAGA |
| EP558 | NNN GGTACC GGCTAAGCAAGTGATGTCATGG |
| EP555 | NNN GCGGCCGC CCTGTTGGTTATCTAACCAGATTG |
| EP212 | TCCATTGCTCAGCGGTGCTGTCC |
| Gli1-for for qPCR | AGCTGCACTGAAGGATCTC |
| Gli1-Rev for qPCR | TACAGCGAGAGTTGATGAAAG |
| Gli2-For for qPCR | GATGCCAACCAGAACAAG |
| Gli2-Rev for qPCR | TTGCTCCGCTTATGAATG |
| Pifo-For for qPCR | ACATCACTCATGGGAAGGTG |
| Pifo-Rev for qPCR | AAACCATTCCAATCTGTTGC |
| Gprasp2-For for qPCR | AGGCGACTGTCTCAAATCTC |
| Gprasp2-Rev for qPCR | TTAAAGCAGGGCTCCAAG |
| GAPDH-For for qPCR | TGGATGCAGGGATGATGT |
| GAPDH-Rev for qPCR | ATTCAACGGCACAGTCAA |
| Ptch1-for for RT-PCR | GAAGGCGCTAATGTTCTGAC |
| Ptch1-Rev for RT-PCR | TACCTAGGAGGTATGCTGTC |
| Gli1-for for RT-PCR | TGCCAGATATGCTTCAGCCA |
| Gli1-Rev for RT-PCR | TGCCAGATATGCTTCAGCCA |
| Gli2-for for RT-PCR | ATCAAGAGGGAGCTACACGCAACA |
| Gli2-Rev for RT-PCR | TTGGGCATAGCTTCTGCTGTCTCT |
| Smo-for for RT-PCR | TTCCGGGACTATGTGCTATGCCAA |
| Smo-Rev for RT-PCR | AGACAGTGTGCATGCTGAAGGAGA |
| Pifo-for for RT-PCR | AGAAAGCATCGTAGCCGTGT |
| Pifo-Rev for RT-PCR | GGGTGACAATGAGACCCTGT |
| β-actin-for for RT-PCR | GACGAGGCCCAGAGCAAGAG |
| β-actin-Rev for RT-PCR | ATCTCCTTCTGCATCCTGTC |
